# Efficacy of prehabilitation modalities in total knee arthroplasty: *A systematic review and network meta-analysis*

**DOI:** 10.1016/j.ocarto.2026.100863

**Published:** 2026-08-21

**Authors:** Monique A.T. van der Vorst, Geeske Peeters, Gerjon Hannink, Rik Dijkman, Marcel G.M. Olde Rikkert

**Affiliations:** a Department of Geriatrics, Radboud University Medical Centre, Geert Grooteplein Zuid 10, 6525 GA, Nijmegen, the Netherlands; b Department of Orthopedics, Jeroen Bosch Hospital, Henri Dunantstraat 1, 5223 GZ ‘s-Hertogenbosch, the Netherlands; c Department of Medical Imaging, Radboud University Medical Centre, Geert Grooteplein Zuid 10, 6525 GA, Nijmegen, the Netherlands; d Department of Medical BioSciences, Radboud University Medical Centre, Geert Grooteplein Zuid 10, 6525 GA, Nijmegen, the Netherlands

**Keywords:** Prehabilitation, Total knee arthroplasty, Osteoarthritis, Physical function, Network meta-analysis, Preoperative intervention

## Abstract

**Objective:**

Up to 30% of patients with osteoarthritis experience suboptimal recovery after total knee arthroplasty (TKA), increasing interest in prehabilitation as a potential strategy to improve postoperative outcomes. This study compared the efficacy of individual prehabilitation modalities versus standard care for physical function, quadriceps strength, pain, and health-related quality of life (HR-QoL) at 3 months after TKA, with additional analyses at 4–8 weeks.

**Design:**

We conducted a systematic review and network meta-analysis (NMA) of randomized controlled trials (RCTs) following PRISMA guidelines. Eligible studies included adults undergoing TKA for osteoarthritis who received preoperative prehabilitation targeting physical, educational, nutritional, or behavioral domains. Comparators were standard care or alternative prehabilitation interventions. Confidence in the evidence was assessed using the CINeMA framework.

**Results:**

20 RCTs (1095 patients) comparing eight prehabilitation modalities were included. At 3 months after TKA, no prehabilitation modality demonstrated statistically significant improvements in physical function, quadriceps strength, pain, or HR-QoL compared with standard care. Similar findings were observed at 4–8 weeks after TKA, except that lower-extremity strength training significantly improved quadriceps strength compared with standard care. Confidence in the evidence was predominantly low to very low, primarily because of imprecision, although three treatment comparisons were supported by moderate confidence.

**Conclusion:**

Current evidence does not support recommending one prehabilitation modality over another before TKA. Although lower-extremity strength training improved short-term quadriceps strength, this finding was supported by low-confidence evidence. Larger, adequately powered head-to-head trials are needed to determine whether specific prehabilitation modalities provide clinically meaningful postoperative benefits.

**Registration:**

PROSPERO (CRD42024490615).

## 1 Introduction

Total knee arthroplasty (TKA) is an effective treatment for individuals with end-stage knee osteoarthritis (OA), aiming to relieve pain, restore physical function, and improve quality of life [1]. Although most patients experience favorable outcomes, 15–30% experience delayed or incomplete recovery [[2], [3], [4], [5], [6]]. As the prevalence of OA continues to increase due to population aging and rising obesity rates, the demand for TKA is projected to grow substantially [7]. This increasing demand, together with the invasive and costly nature of TKA, highlights the need to optimize postoperative recovery [7,8]. In this context, prehabilitation has emerged as a promising strategy to improve postoperative outcomes [9,10].

Prehabilitation comprises structured preoperative interventions, including physical conditioning, education, and psychosocial support, aimed at enhancing patients’ physical and mental readiness for surgery. It is based on the hypothesis that targeted interventions can mitigate inactivity-related decline, optimize physiological function, and ultimately enhance postoperative recovery [11]. Several systematic reviews have evaluated the effects of prehabilitation in patients undergoing TKA; however, their findings remain inconclusive [10,[12], [13], [14], [15], [16], [17], [18], [19], [20], [21], [22], [23]]. Most reviews compared prehabilitation with standard care and pooled diverse prehabilitation interventions, including exercise, education, and multimodal programs, as a single intervention, thereby obscuring potential modality-specific effects [10,[12], [13], [14],^16^,^17^,^20^,^21^,^23^]. Consequently, the relative effectiveness of individual prehabilitation modalities remains unclear.

Because direct head-to-head comparisons between prehabilitation modalities are scarce, a network meta-analysis (NMA) provides an analytical framework for integrating direct and indirect evidence to compare multiple modalities [24]. Therefore, we conducted a systematic review and NMA comparing the relative efficacy of individual prehabilitation modalities versus standard care for physical function, quadriceps strength, pain, and HR-QoL at three months after TKA, with additional analyses at 4–8 weeks after TKA to assess early postoperative outcomes.

## 2 Methods

This systematic review and NMA is registered in PROSPERO (CRD42024490615) [25] and conducted following the Preferred Reporting Items for Systematic Reviews and Meta-Analyses guidelines [24].

### 2.1 Data sources and search strategy

A systematic search was conducted in PubMed, Cochrane, EMBASE, Web of Science, MEDLINE, PsycINFO, CINAHL, and PEDro from inception to October 16, 2023. The search strategy, developed in consultation with a medical librarian, included relevant synonyms and MeSH terms for prehabilitation, physical function, pain, quality of life, and total knee arthroplasty. The complete search strategy is presented in Supplement 1. Only full-text articles published in English, German, or Dutch were included.

### 2.2 Eligibility criteria

Randomized controlled trials (RCTs) were eligible if they included adults undergoing primary unilateral total knee arthroplasty for osteoarthritis. Studies were excluded when they involved unicompartmental or bilateral procedures, mixed TKA–THA cohorts without joint-specific data, or populations selected for specific mental or physical health conditions. Eligible interventions comprised preoperative prehabilitation programs targeting physical, educational, nutritional, or behavioral domains, alone or in combination; exercise-based interventions were required to have a minimum duration of two weeks. Comparators consisted of standard care or an alternative prehabilitation modality, with comparable postoperative rehabilitation between groups. Trials were required to report at least one prespecified outcome at either 4–8 or 12–14 weeks postoperatively. Prespecified outcomes were physical function, quadriceps strength, pain, or HR-QoL, based on OMERACT–OARSI Core Domain Set, with quadriceps strength additionally included because of its prognostic relevance for functional recovery [[25], [26], [27], [28]]. Eligible outcome measures are listed in Supplement S2. Prespecified performance-based outcomes and postoperative complications were not analyzed because reporting at the selected time points was limited and inconsistent.

### 2.3 Study selection, data extraction, and intervention classification

Search results were imported into Covidence, and duplicates were removed. Two reviewers (MV and RD) independently screened all records using ASReview [29], which prioritizes studies by predicted relevance. Disagreements were resolved by a third reviewer (GP). MV and resolved with a third reviewer independently conducted full-text screening and data extraction using a Covidence-based form capturing (1) 4–8 and 12–14 weeks post-TKA outcomes, (2) study design details (blinding, randomization), (3) participant demographics (age, sex, BMI), (4) intervention details, including exercise frequency, intensity, session duration, total duration, supervision, and adherence, and (5) statistical methods. Graphical data were extracted using PlotDigitizer [30,31].

Interventions were grouped into modality categories based on their primary therapeutic focus using a pragmatic classification of the dominant intervention component. Multidomain exercise was defined as interventions that combined two or more exercise components (e.g., strength, aerobic, balance, flexibility, or neuromuscular). Education-only interventions consisted of programs focused on information provision, behavioral guidance, or self-management, whereas combined exercise–education programs were classified as a separate modality. Blood flow restriction (BFR) exercise was defined as low-load resistance or cycling training performed under partial limb occlusion to enhance muscular adaptations.

### 2.4 risk of bias (RoB) and confidence in the evidence

Two reviewers (MV and GP) independently assessed the RoB at the outcome level using the Cochrane RoB 2.0 tool [32], classifying each domain as ‘high risk’, ‘some concerns’, or ‘low risk.’ Discrepancies were resolved with a third reviewer. Potential publication bias was examined using funnel plots and Egger’s test [33,34]. The certainty of evidence for each treatment comparison was evaluated using the Confidence in Network Meta-Analysis framework [35,36], and rated as high, moderate, low, or very low.

### 2.5 Data synthesis

Due to variation in outcome measures across trials, treatment effects were pooled as standardized mean differences (SMDs; Cohen’s d) with 95% confidence intervals (CIs), using absolute follow-up scores [37,38]. Positive Standardized mean differences (SMDs) favored the prehabilitation modality over standard care. SMDs of 0.2, 0.5, and 0.8 were interpreted as small, moderate, and large effects, respectively [39]. An effect was considered clinically relevant when the point estimate exceeded the minimal important difference (|SMD|>0.5) and the 95% CI excluded trivial effects (|SMD|≤0.2).

When multiple instruments assessed the same outcome within a trial, the most reliable and clinically relevant measure was selected according to a predefined hierarchy [40]. Reverse-scaled PROMs were multiplied by −1 in accordance with Cochrane Handbook [37,41]. Medians were converted to means and missing standard deviations (SDs) were derived from reported summary statistics for four studies [[42], [43], [44], [45]] using established methods [37,46]. Authors were contacted to obtain missing group-level SDs or follow-up data. SDs were not reconstructed from CIs derived from adjusted mixed-effects models because these intervals reflect model-based uncertainty rather than within-group variability. These studies were excluded from the primary analysis, and their inclusion was evaluated in a sensitivity analysis. When follow-up data were unavailable, change scores were used where reported and evaluated in separate sensitivity analyses.

### 2.6 Statistical analysis

We conducted frequentist random-effects NMAs to estimate the efficacy of prehabilitation modalities at 4–8 weeks and 12–14 weeks postoperatively. Network geometry was visualized using network plots, with node size proportional to modality sample size and edge thickness to the number of direct comparisons. Networks were assessed for imbalance and potential bias. Network robustness was assessed using the direct evidence proportion (0–1), mean path length (values > 2 indicating greater indirectness), and minimal aggregated parallelism, with higher values indicating more robust estimates [47]. Network heterogeneity was assessed using Q-statistic, I^2^, and between-study variance (τ^2^), estimated with the DerSimonian-Laird method [48]. 95% Prediction intervals (PIs) were reported to reflect the expected range of effects in future studies. Global inconsistency was assessed using a design-by-treatment interaction model, and local inconsistency was assessed using node-splitting via back-calculation when direct and direct evidence were available [[49], [50], [51], [52], [53], [54]]. Transitivity was assessed qualitatively by comparing key clinical and methodological characteristics (including age, BMI, sex, and postoperative rehabilitation) across treatment nodes and visually using bar plots of potential effect modifiers [[55], [56], [57]]. Analyses were performed in R. Additional methodological details are provided in Supplementary Material S2.

### 2.7 Summary measures

Forest plots summarized treatment effects versus standard care, and league tables presented pairwise comparisons. Treatments were ranked using P-scores (0–1), with higher values indicating a greater likelihood of being the most effective [34,58].

### 2.8 Sensitivity analyses

Where applicable, four sensitivity analyses were performed: (1) excluding studies at high risk of bias; (2) excluding change score-derived SMDs; (3) including studies excluded from the primary analysis because group-level SDs could not be derived from adjusted mixed-effects models; and (4) excluding modalities with fewer than 20 participants.

## 3 Results

### 3.1 Search results & study selection

**Fig. 1:**
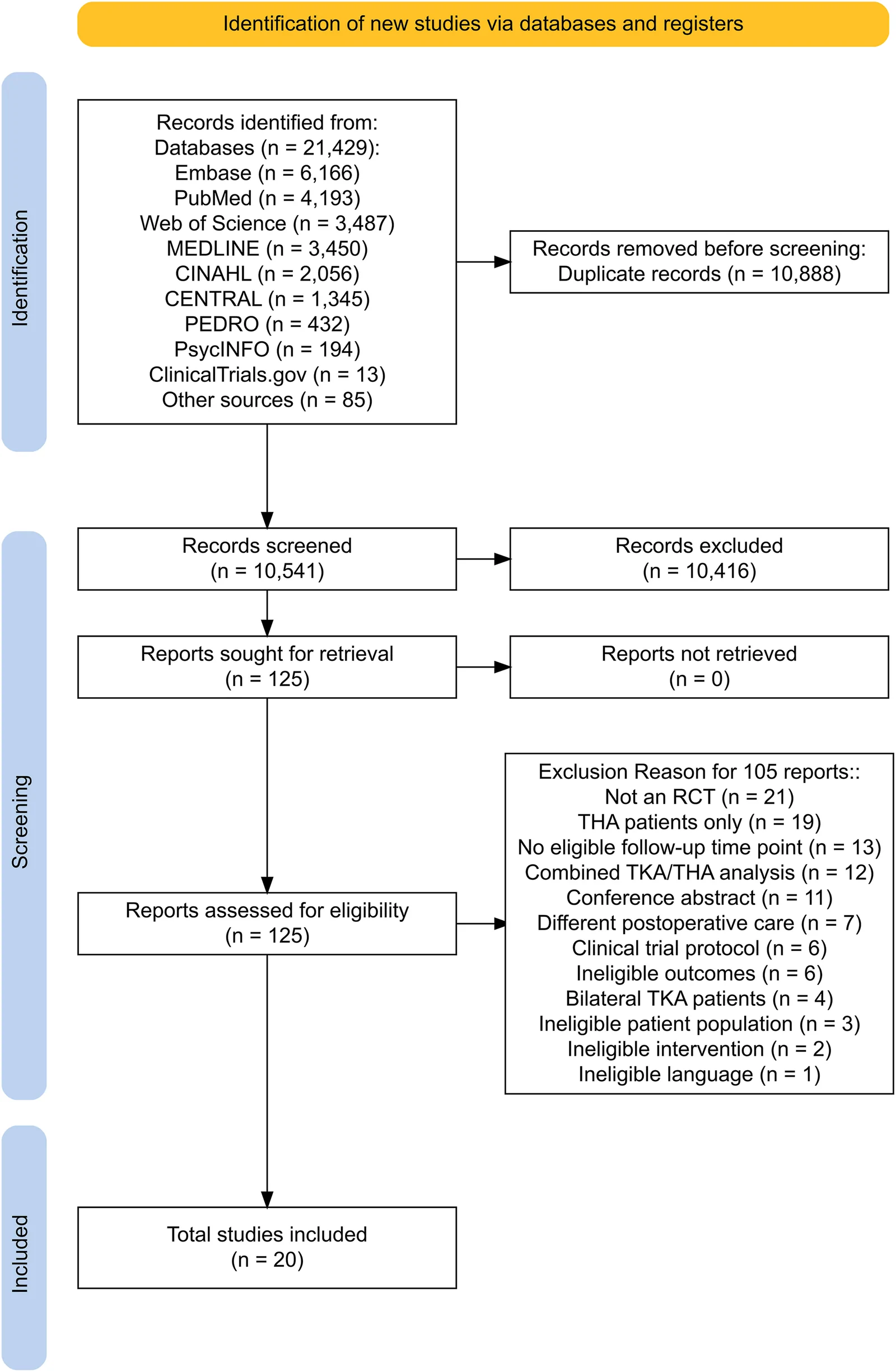
Preferred Reporting Items for Systematic Reviews and Meta-Analyses flowchart of the study selection process.

The systematic literature search identified 10,541 unique records, of which 20 RCTs involving 1095 TKA patients met the inclusion criteria [11,30,31,[42], [43], [44], [45],[59], [60], [61], [62], [63], [64], [65], [66], [67], [68], [69], [70], [71]]. Four trials contributed only to the 4-8-week analyses. Study selection is illustrated in Fig. 1, with excluded full-text articles and reasons provided in Supplement S3 [72].

### 3.2 Study characteristics

Table 1 summarizes the characteristics of the 20 included trials. Across all studies, 1095 patients reported a mean age of 68.0 years (range: 60.6–72.3), a mean BMI of 29.7 (range: 22.6–38.8), and 63% were female. Sample size ranged from 14 to 164 patients (median: 48). Baseline characteristics and scores were broadly comparable within and between trials (Suppl. S4, S5).

**Table 1:** Study characteristics.

Study	Country	Participants (N)				Age, years; mean (SD)			BMI, kg/m^2;^ mean (SD)			Female, %		
		Total	CON	INT 1	INT 2	CON	INT 1	INT 2	CON	INT 1	INT 2	CON	INT 1	INT 2
Beaupre et al. (2004)	Canada	109	58	51	–	67 (6)	67 (7)	–	31 (5)	32 (6)	–	50	60	–
Blasco et al. (2020)	Spain	77	26	25	26	70.9 (9.5)	70.2 (7.2)	72.3 (4.5)	31.2 (4.6)	32.5 (4.9)	30.8 (5.7)	58	76	73
Brown et al. (2012)	United States	18	7	11	–	NR	NR	–	34.6 (7.6)	38.8 (8.8)	–	NR	NR	–
Calatayud et al. (2017)	Spain	44	22	22	–	66.7 (3.1)	66.8 (4.8)	–	31 (3.8)	32 (4.2)	–	NR	NR	–
Dominguez-Navarro et al. (2021)	Spain	65	21	24	20	70.2 (5.6)	70.8 (5.4)	70.4 (6.4)	29.8 (6.5)	29.2 (4.6)	29.4 (4.9)	67	58	65
Franz et al. (2022)	Germany	30	10	10	10	66.3 (7.1)	64.2 (7.7)	61.5 (8.8)	29.4 (NR)	27.7 (NR)	26.8 (NR)	40	30	30
Gstoettner et al. (2011)	Austria	35	20	15	–	66.9 (61–75)^a^	72.8 (65–78)^a^	–	28.2 (NR)	27.4 (NR)	–	70	89	–
Huber et al. (2015)	Switzerland	41	◇	20	21	◇	71.9 (8.1)	68.8 (8.0)	◇	29.9 (5.5)	30.8 (4.9)	◇	44	50
Jorgensen et al. (2024)	Denmark	65	34	31	–	66.1 (7.9)	67.1 (8.3)	–	30.5 (6.2)	32.5 (10.3)	–	52	62	–
Van Leeuwen et al. (2014)	Netherlands	16	7	9	–	69.5 (7.1)	71.8 (7.5)	–	27.9 (3.1)	27.9 (4.6)	–	50	70	–
McKay et al. (2012)	Canada	17	◇	10	7	◇	60.6 (8.1)	63.5 (4.9)	◇	33.8 (7.0)	35 (6.1)	◇	67	50
Nam et al. (2023)	Korea	164	83	81	–	72.0 (6.1)	71.6 (6.8)	–	26.4 (3.2)	27.8 (4.2)	–	77	86	–
Rooks et al. (2006)	United States	29	◇	15	14	◇	69 (8)	65 (8)	◇	33.9 (6.5)	35.7 (9)	◇	57	50
Savkin et al. (2021)	Turkey	40	20	20	–	64.2 (5.5)	64.1 (5.1)	–	31.9 (5.7)	32.0 (4.2)	–	5	10	–
Skoffer et al. (2016)	Denmark	50	21	29	–	70.1 (6.4)	70.7 (7.3)	–	31.8 (24.3–42.2)^b^	30.0 (22.6–42.5)^b^	–	59	63	–
Sun et al. (2023)	China	81	41	40	–	68.5 (7.9)	66.4 (8.3)	–	23.6 (2.5)	22.6 (3.3)	–	66	72	–
Topp et al. (2009)	United States	54	28	26	–	63.5 (6.7)	64.1 (7)	–	32 (6.1)	32.2 (5.9)	–	64	73	–
Tungtrongjit et al. (2012)	Thailand	60	30	30	–	65.9 (7.2)	63 (7.6)	–	25.3 (3.8)	24.3 (2.4)	–	80	87	–
Villadsen et al. (2014)	Denmark	81	40	41	–	65.1 (9.0)	67.1 (8.8)	–	33.4 (5.8)	30.8 (4.9)	–	60	61	–
Walls et al. (2010)	Ireland	14	5	9	–	63.2 (11.4)	63.2 (11.4)	–	32.8 (6.3)	32.8 (6.3)	–	80	67	–

Study	CON	Prehabilitation modality		Outcome measures				Outcome Assesment	
		INT 1	INT 2	Physical function	Quadriceps strength	Pain	HR-QoL	4–8 weeks	12–14 weeks
Beaupre et al. (2004)	SC	Exercise & education	–	WOMAC function	Knee extension (isometric)	WOMAC pain	SF-36 PCS	–	3 Months
Blasco et al. (2020)	SC	Multidomain exercise	Home-based multidomain	KOOS ADL	Knee extension (isometric)	KOOS pain	KOOS QoL	6 Weeks	–
Brown et al. (2012)	SC	Multidomain exercise	–	SF-36 Phys. Funct.	–	SF-36 pain	–	–	3 Months
Calatayud et al. (2017)	SC	Lower-extremity strength	–	WOMAC function	Knee extension (isometric)	WOMAC pain	–	1 Month	3 Months
Dominguez-Navarro et al. (2021)	SC	Lower-extremity strength	Multidomain exercise	KOOS ADL	Knee extension	KOOS pain	KOOS QoL	6 Weeks	–
Franz et al. (2022)	SC	Aerobic exercise	BFR-exercise	KOOS ADL	Knee extension	KOOS pain	KOOS QoL	–	3 Months
Gstoettner et al. (2011)	SC	Multidomain exercise	–	WOMAC function	–	WOMAC pain	–	6 Weeks	–
Huber et al. (2015)	◇	Education	Exercise & education	KOOS ADL	Knee extension (isometric)	KOOS pain	KOOS QoL^c^	6 Weeks	3 Months
Jorgensen et al. (2024)	SC	BFR-exercise	–	KOOS ADL	Knee extension (isometric)	KOOS pain	KOOS QoL	–	3 Months
Van Leeuwen et al. (2014)	SC	Lower-extremity strength	–	–	Knee extension (isometric)	–	–	6 Weeks	12 Weeks
McKay et al. (2012)	◇	Upper-body strength	Lower-extremity strength	WOMAC function	Knee extension (isometric)	WOMAC pain	SF-36 PCS	6 Weeks	12 Weeks
Nam et al. (2023)	SC	Education	–	WOMAC function	–	WOMAC pain	SF-36 PCS	–	3 Months
Rooks et al. (2006)	◇	Education	Multidomain exercise	WOMAC function	–	WOMAC pain	–	8 Weeks	–
Savkin et al. (2021)	SC	NMES	–	KOOS ADL	Knee extension	KOOS pain	KOOS QoL	4 Weeks	12 Weeks
Skoffer et al. (2016)	SC	Lower-extremity strength	–	KOOS ADL	Knee extension (isometric)	KOOS pain	KOOS QoL	6 Weeks	12 Weeks
Sun et al. (2023)	SC	Multidomain exercise	–	–	Knee extension (isometric)	–	–	–	3 Months
Topp et al. (2009)	SC	Multidomain exercise	–	–	Knee extension	–	–	1 Month	3 Months
Tungtrongjit et al. (2012)	SC	Lower-extremity strength	–	WOMAC function	Knee extension	WOMAC pain	–	1 Month	3 Months
Villadsen et al. (2014)	SC	Multidomain exercise	–	KOOS ADL	Knee extension	KOOS pain	KOOS QoL	6 Weeks	3 Months
Walls et al. (2010)	SC	NMES	–	WOMAC function	Knee extension (isometric)	WOMAC pain	SF-36 PCS	6 Weeks	12 Weeks

ADL, activities of daily living; BFR, blood flow restriction; BMI, body mass index; CON, standard care group; HR-QoL, health-related quality of life; INT, intervention group; KOOS, Knee Injury and Osteoarthritis Outcome Score; N, number of participants assessed after prehabilitation; NMES, neuromuscular electrical stimulation; PCS, Physical Component Summary; Phys. Func, physical function; QoL, quality of life; SC, standard care; SD, standard deviation; SF-36, 36-Item Short Form Survey; WOMAC, Western Ontario and McMaster Universities Osteoarthritis Index.

◇ No Standard care group in study; - not applicable; NR, not reported.

a Mean (range).

b Median (range).

c HR-QoL data from Huber et al., 2015 were excluded in 4–8-week NMA because they formed a disconnected subnetwork.

Prehabilitation interventions were classified into eight prehabilitation modalities: 1) Lower-extremity strength training [61,62,[65], [66], [67],^69^], 2) Multidomain exercise [11,42,43,60,68,70,71], 3) BFR exercise [30,45], 4) Neuromuscular electrostimulation [31,64], 5) Aerobic exercise [30], 6) Upper-body strength training [62], 7) Exercise combined with education [44,59], 8) Education [44,63,71]. Detailed intervention content and the FITT characteristics (frequency, intensity, type, and time) are presented in Table 2 and Supplement S6. Three trials evaluated education-only prehabilitation without an exercise component, delivered as structured group-based knee education, individualized expectation management, or mailed educational booklets with telephone support. Most exercise-based interventions were supervised and delivered in clinical settings (n = 16), while others used hybrid clinic–home delivery with partial supervision (n = 4) or were unsupervised home-based programs (n = 6). Intervention dose varied substantially across modalities. The median exercise duration was 6 weeks (range: 3–12), with clinic-based programs typically delivered 1–3 days per week and home-based programs prescribed 2–7 days per week. Across exercise modalities, the total number of sessions ranged from 10 to 37. Session duration (30–60 min) was inconsistently reported and missing in nearly half of the trials, and exercise intensity was variably described, limiting cross-study comparability. Adherence was reported in 13 trials, ranging from 74% to 100%.

**Table 2:** Prehabilitation intervention characteristics.

Study	Standard	Intervention		Location	Supervision	Length	Planned sessions^a^			Frequency (x/wk)		Duration (min)		Adherence^f^
	Care	Int	Modality				Total	Clinic	Home	Clinic	Home	Clinic	Home	(%)
Franz et al. (2022)	SC	1	Aerobic exercise	Clinic	Supervised	6 wk	12	12	–	2	–	50	–	100
Franz et al. (2022)	SC	2	BFR exercise	Clinic	Supervised	6 wk	12	12	–	2	–	50	–	100
Jorgensen et al. (2024)	SC	1	BFR exercise	Clinic	Supervised	8 wk	24	24	–	3	–	NR	–	91^g^
Huber et al. (2015)	◇	1	Education	Clinic	Supervised	3 wk	3	3	–	1	–	NR	–	NR
Nam et al. (2023)	SC	1	Education	Clinic	Supervised	NR	NR	NR	–	NR	–	NR	–	NR
Rooks et al. (2006)	◇	1	Education	Home	Unsupervised	6 wk	5	–	5	–	0.8		NR	NR
Beaupre et al. (2004)	SC	1	Exercise & education	Clinic	Supervised	4 wk	12	12	–	3	–	30	–	100
Huber et al. (2015)	◇	2	Exercise & education	Clinic	Supervised	4–12 wk	10^b^	10	–	1–3	–	60	–	76^h^
Blasco et al. (2020)	SC	2	Home-based multidomain	Home	Unsupervised	4 wk	13	1	12	–	3	–	NR	97
Calatayud et al. (2017)	SC	1	Lower-extremity strength	Clinic	Supervised	8 wk	24	24	–	3	–	NR	–	NR
Dominguez-Navarro et al. (2021)	SC	1	Lower-extremity strength	Clinic	Supervised	4 wk	12	12	–	3	–	30–40	–	93
Van Leeuwen et al. (2014)	SC	1	Lower-extremity strength	Clinic & home	Hybrid	6 wk	24–35^c^	12	12–18	2–3	2–3	NR	NR	NR
McKay et al. (2012)	◇	2	Lower-extremity strength	Clinic	Supervised	6 wk	18	18	–	3	–	30	–	98
Skoffer et al. (2016)	SC	1	Lower-extremity strength	Clinic	Supervised	4 wk	12	12	–	3	–	60	–	94
Tungtrongjit et al. (2012)	SC	1	Lower-extremity strength	Home	Unsupervised	3 wk	21	–	21	–	7	–	NR	NR
Blasco et al. (2020)	SC	1	Multidomain exercise	Clinic	Supervised	4 wk	12	12	–	3	–	NR	–	93
Brown et al. (2012)	SC	1	Multidomain exercise	Clinic & home	Hybrid	8 wk	24^d^	8	16	1	2	45	45	89
Dominguez-Navarro et al. (2021)	SC	2	Multidomain exercise	Clinic	Supervised	4 wk	12	12	–	3	–	45–60	–	93
Gstoettner et al. (2011)	SC	1	Multidomain exercise	Clinic & home	Hybrid	6 wk	42	6	36	1	6	45	NR	NR
Rooks et al. (2006)	◇	2	Multidomain exercise	Clinic	Supervised	6 wk	18	18	–	3	–	30–60	–	89
Sun et al. (2023)	SC	1	Multidomain exercise	Home	Unsupervised	4 wk	21	–	20	1	5	–	60	NR
Topp et al. (2009)	SC	1	Multidomain exercise	Clinic & home	Hybrid	NR	13^e^	NR	NR	1	2	NR	NR	NR
Villadsen et al. (2014)	SC	1	Multidomain exercise	Clinic	Supervised	8 wk	16	16	–	2	–	60	–	74^i^
Savkin et al. (2021)	SC	1	NMES	Home	Unsupervised	6 wk	30	–	30	–	5	–	20	NR
Walls et al. (2010)	SC	1	NMES	Home	Unsupervised	8 wk	37	–	37	–	3–5	–	20	99
McKay et al. (2012)	◇	1	Upper -body strength	Clinic	Supervised	6 wk	18	18	–	3	–	30	–	93

◇ No Standard care group in study; - not applicable; BFR, blood flow restriction; Int, intervention group; min, minutes; x/wk: sessions per week; NMES, neuromuscular electrical stimulation; NR, Not reported; SC, standard care; wk, weeks. Supervision was categorized as ‘Supervised’ (intervention delivered under the direct supervision of a healthcare professional); ‘Unsupervised’ (self-directed), or ‘Hybrid’ (combination of supervised and unsupervised components.

a Number of planned sessions. When unavailable, the mean number of completed sessions is reported instead.

b Huber et al. (2015): median (IQR) 10 (8–14).

c Van Leeuwen et al. (2014): mean ± SD (range]) 12 ± 2 (11–17).

d Brown et al. (2012): mean ± SD 16.3 ± 6.0.

e Topp et al. (2009): mean ± SD (range) 13.04 ± 7.5 (4–23).

f Mean adherence among participants who completed the intervention.

g 80% of prescribed sessions attended.

h 8 sessions; and.

i 12 sessions.

In 17 trials, prehabilitation was compared with standard care, typically defined as continuation of usual activities or a single educational session (Suppl. S7). Postoperative rehabilitation was similar between groups within individual trials but varied widely across studies, ranging from standard mobilization routines or unspecified physiotherapy to structured inpatient programs, outpatient strength training, or home-based rehabilitation (Suppl. S7). Several studies did not report postoperative care, potentially affecting the transitivity assumption for indirect comparisons. Study-level pairwise comparisons and corresponding forest plots are also provided in Supplement S5 and S8.

### 3.3 RoB and confidence in the evidence

Of the 20 trials, 12 (60%) were judged to have some concerns regarding RoB, and 8 (40%) were rated as high risk (Suppl. S9). High RoB was primarily driven by the exclusion of patients with postoperative complications [60,61,[66], [67], [68], [69]]. Because most outcomes were patient-reported, the outcome measurement domain frequently raised some concerns due to a lack of blinding. Many studies also lacked a pre-registered protocol. Most trials were judged to be at low RoB for randomization procedures and missing outcome data.

At the treatment-comparison level, integrating risk-of-bias assessments with study contributions revealed major concerns about within-study bias for comparisons of lower-extremity strength training versus standard care across all outcomes. Overall confidence in the evidence, assessed using the Confidence in Network Meta-Analysis framework, was predominantly rated as low to very low, primarily because of imprecision, as reflected by wide CIs and limited contributing evidence, with additional downgrading due to indirectness arising from variation and incomplete reporting of prehabilitation intervention characteristics and postoperative rehabilitation protocols across studies (Suppl. S10). Only three treatment comparisons were rated as having moderate confidence. Contour-enhanced funnel plots showed no asymmetry, indicating no evidence of publication bias. In addition, Egger’s test showed no evidence of small-study effects for any outcome (Suppl. S11).

### 3.4 Primary NMAs at 3 months after TKA

#### 3.4.1 Physical function

**Fig. 2:**
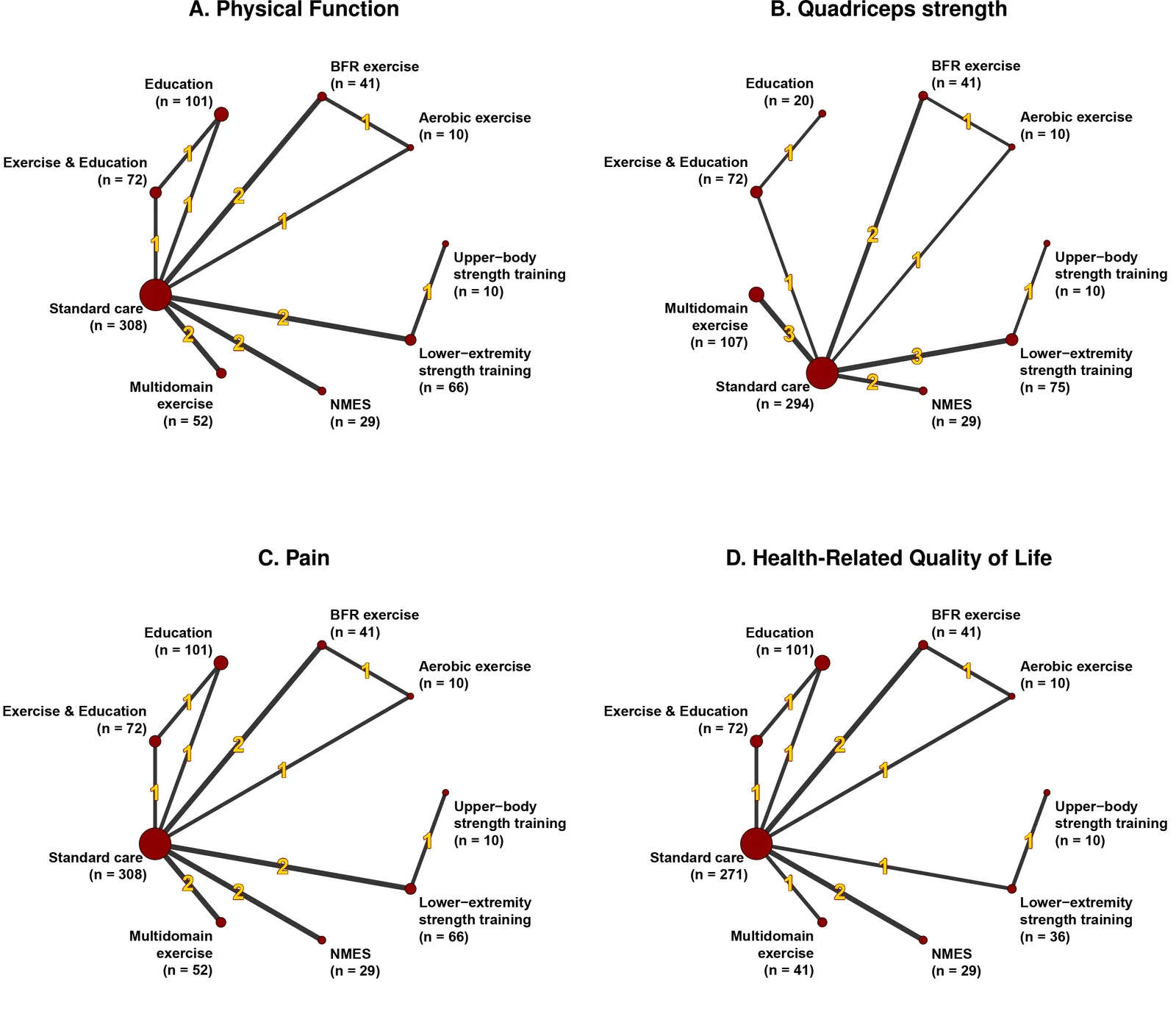
**Network plots of prehabilitation modalities at 3 months after TKA.** Nodes represent prehabilitation modalities, with node sizes proportional to the total sample size (n). Edges represent direct treatment comparisons, with edge thickness proportional to the number of studies contributing to each comparison. Separate network plots are shown for: (A) Physical function, (B) Quadriceps strength, (C) Pain, and (D) Health-related quality of life. BFR, blood flow restriction; NMES, neuromuscular electrical stimulation.

**Fig. 3:**
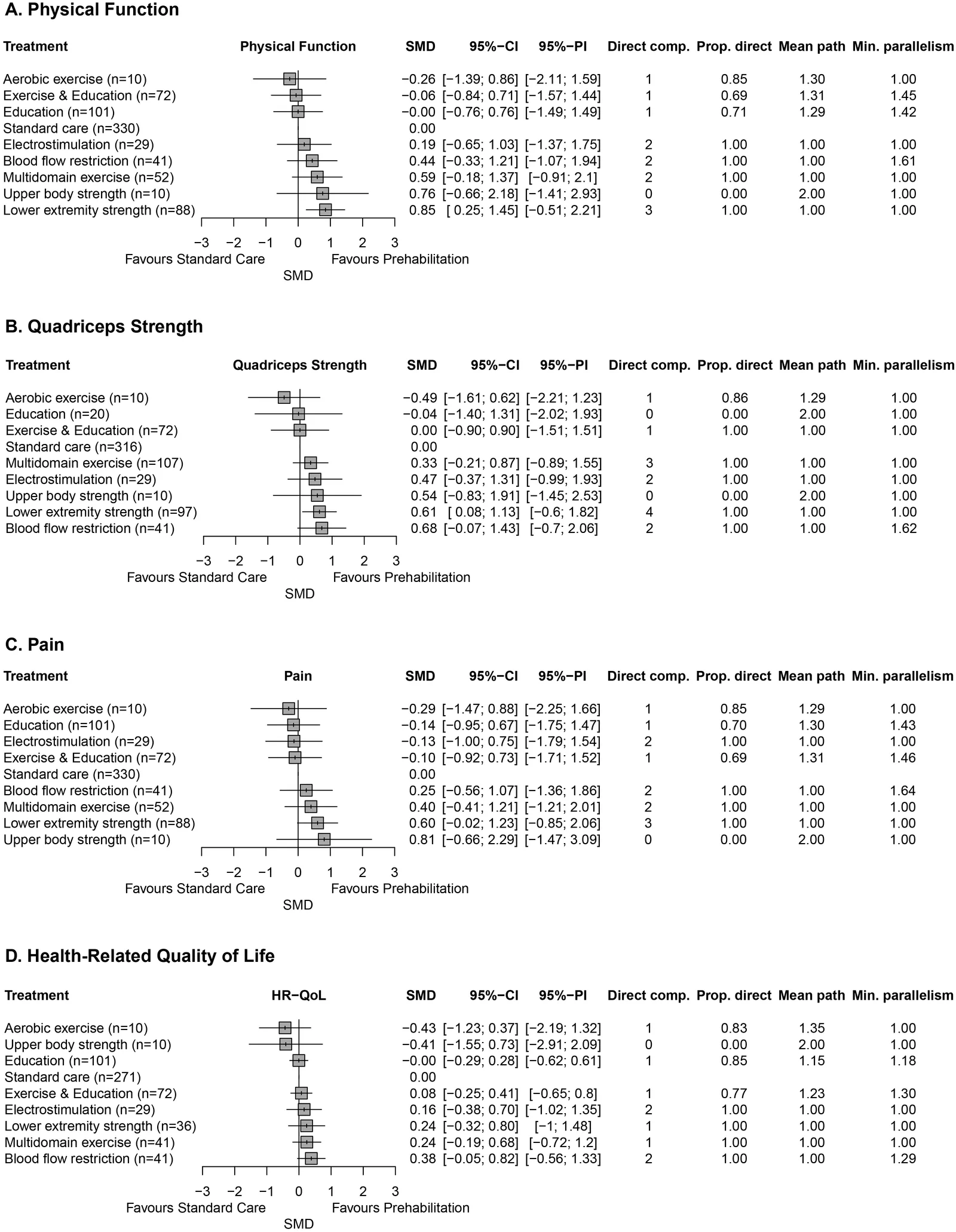
**NMA forest plots at 3 months after TKA.** SMDs with 95∖% CIs and prediction intervals (PIs) for all modalities versus standard care. Positive SMDs favor the prehabilitation modality. Outcome measures: (A) Physical function, (B) Quadriceps strength, (C) Pain, and (D) Health-related quality of life (HR-QoL). BFR, blood flow restriction; Direct comp, number of direct comparisons; Mean path, mean path length, indicates indirectness (shorter = more direct evidence); Min. Parallelism, the minimum number of independent evidence paths; n, number of participants per modality; NMES, neuromuscular electrical stimulation; Prop. direct, proportion of direct evidence.

**Fig. 4:**
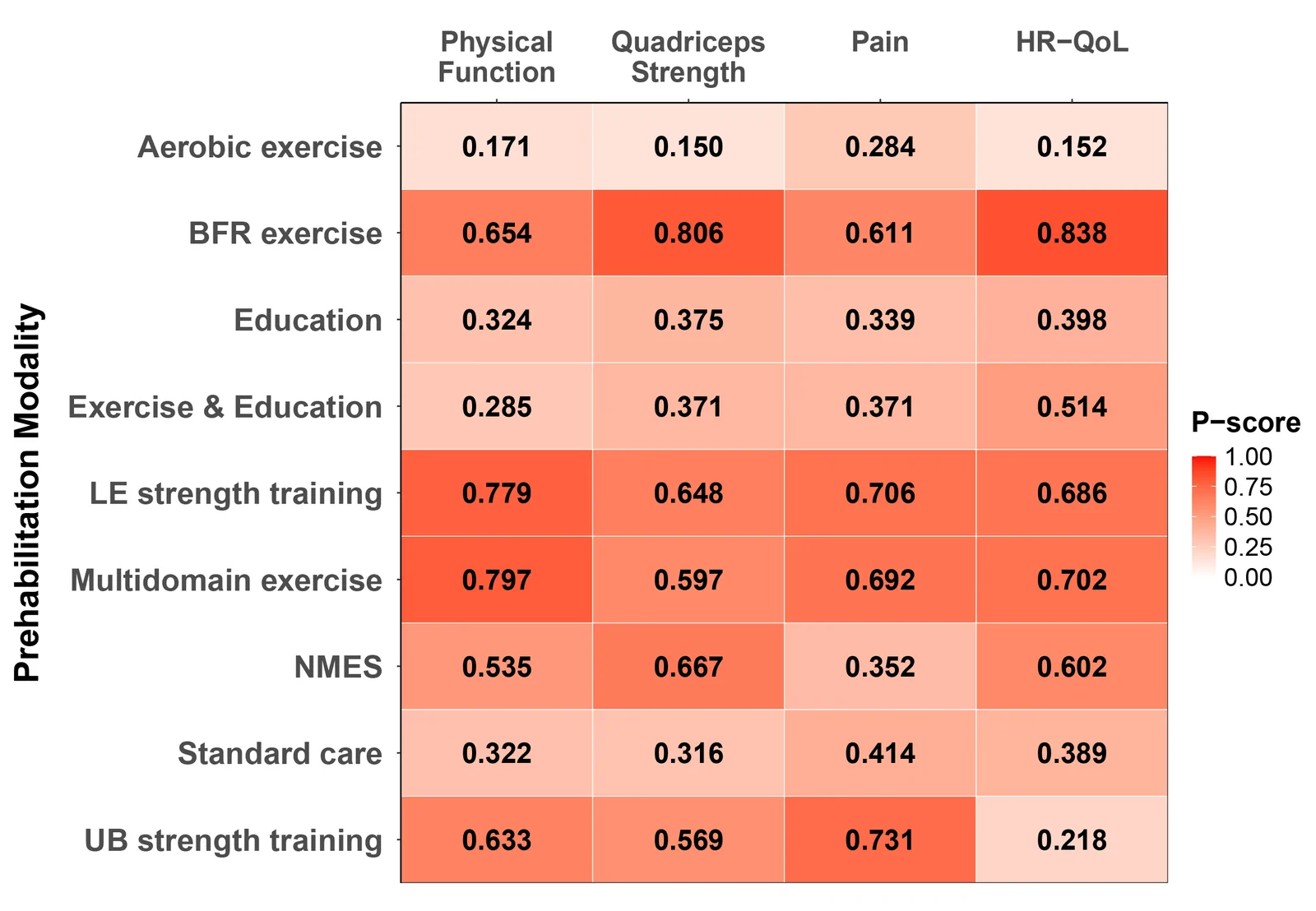
**Probability Ranking Heatmap of Prehabilitation Modalities at 3 Months After TKA.** Heatmap showing the relative ranking of prehabilitation modalities across physical function, quadriceps strength, pain, and health-related quality of life (HR-QoL). Cells represent P-scores ranging from 0 to 1, with higher values indicating a more favorable relative ranking for the corresponding outcome. BFR, blood flow restriction; LE, lower-extremity; NMES, neuromuscular electrical stimulation; UB, upper body.

The NMA for physical function included 12 trials (689 patients) comparing eight prehabilitation modalities, with standard care as reference (308 patients across 11 trials; Fig. 2A). Physical function was assessed using WOMAC Function [31,59,62,63,66], KOOS ADL [30,42,44,45,64,65], and SF-36 Physical Functioning [60]. No prehabilitation modality showed a statistically significant improvement in physical function compared with standard care (Fig. 3A). The largest estimated effects were observed for multidomain exercise (SMD 0.50, 95% CI –0.01–1.00; P-score = 0.80), and lower-extremity strength training (SMD 0.46, 95% CI 0.00–0.92; P-score = 0.78; Fig. 4A). Pairwise treatment comparisons showed no statistically significant differences between modalities (Suppl. 12A). Heterogeneity was low (τ^2^ = 0.034; I^2^ = 26%). There was no evidence of global inconsistency (Q = 4.70, df = 2, p = 0.10). Local inconsistency could not be assessed because of limited direct–indirect overlap (Suppl. S13A).

#### 3.4.2 Quadriceps strength

The NMA for quadriceps strength included 13 trials (658 patients) comparing eight prehabilitation modalities, with standard care as reference (294 patients across 12 trials; Fig. 2B) [11,30,31,[42], [43], [44], [45],^59^,^61^,^62^,^64^,^65^]. No prehabilitation modality showed a statistically significant improvement in quadriceps strength compared with standard care (Fig. 3B). BFR exercise showed the largest estimated effect (SMD 0.68, 95% CI –0.06–1.42) and ranked highest among the evaluated modalities (P-score = 0.81), followed by Neuromuscular electrostimulation (SMD 0.46, 95% CI –0.37–1.29; P-score = 0.67) and lower-extremity strength training (SMD 0.41, 95% CI –0.20–1.01; P-score = 0.65; Fig. 4B). Pairwise treatment comparisons showed no statistically significant differences among prehabilitation modalities (Suppl. S12B). Heterogeneity was moderate (τ^2^ = 0.164; I^2^ = 64%). There was no evidence of global inconsistency (Q = 0.05, df = 1, p = 0.82). Local inconsistency could not be assessed (Suppl. S13B).

#### 3.4.3 Pain

The NMA for pain included 12 trials (689 patients) comparing eight prehabilitation modalities, with standard care as reference (308 patients across 11 trials; Fig. 2C). Pain was assessed using WOMAC Pain [31,59,62,63,66], KOOS Pain [30,42,44,45,64,65], and SF-36 Pain subscales [60]. No prehabilitation modality showed a statistically significant reduction in pain compared with standard care (Fig. 3C), and pairwise treatment comparisons provided no evidence of differences between modalities (Suppl. S12C). Heterogeneity was moderate (τ^2^ = 0.243; I^2^ = 71%). There was no evidence of global inconsistency (Q = 3.82, df = 1, p = 0.15). Local inconsistency could not be assessed (Suppl. S13C).

#### 3.4.4 Health-related quality of life

The NMA for HR-QoL included 10 trials (611 patients) comparing eight prehabilitation modalities, with standard care as reference (271 patients across 8 trials; Fig. 2D). HR-QoL was assessed using KOOS QoL [30,42,44,45,64,65] and SF-36 Physical Component Score [31,59,62,63]. No prehabilitation modality showed a statistically significant improvement in HR-QoL compared with standard care (Fig. 3D). Pairwise treatment comparisons provided no evidence of differences between modalities (Suppl. S12D). Heterogeneity was low (τ^2^ = 0; I^2^ = 0%). There was no evidence of global inconsistency (Q = 2.73, df = 2, p = 0.26). Local inconsistency could not be assessed (Suppl. S13D).

### 3.5 Sensitivity analysis

#### 3.5.1 Exclusion of high-risk-of-bias studies

Excluding high-risk-of-bias studies did not materially affect the overall findings (Suppl. S14). For physical function, heterogeneity increased slightly (τ^2^ = 0.056; I^2^ = 37%), and the estimated effect of lower-extremity strength training was modestly attenuated (Suppl. S14A). For quadriceps strength, BFR exercise remained the highest-ranked modality, with only minor changes in effect estimates and treatment rankings (Suppl. S14B). For pain, estimated effects for the higher-ranked interventions were slightly attenuated, with modest changes in treatment rankings (Suppl. S14C).

#### 3.5.2 Exclusion of studies reporting SMDs calculated from change scores

Excluding studies reporting SMDs calculated from change scores increased estimated effects for BFR exercise across all outcomes (Suppl. S15). For physical function, heterogeneity was eliminated (τ^2^ = 0; I^2^ = 0%), and estimated effects increased for BFR exercise (SMD from 0.32 to 1.13) and multidomain exercise (SMD from 0.50 to 1.10), both becoming statistically significant, and BFR exercise became the highest-ranked modality (Suppl. S15A). For quadriceps strength, heterogeneity and treatment rankings remained largely unchanged, whereas estimated effects modestly increased for BFR exercise and multidomain exercise (Suppl. S15B). For pain, the estimated effect of BFR exercise increased (SMD from 0.26 to 1.18) and became the highest-ranked modality, although the effect remained non-significant (Suppl. S15C). For HR-QoL, the estimated effect of BFR exercise increased (SMD from 0.38 to 0.97) and became statistically significant, while multidomain exercise was no longer represented in the network (Suppl. S15D).

#### 3.5.3 Inclusion of model-based CIs

Including the previously excluded study by Calatayud et al. [67], using SDs derived from model-based CIs, increased estimated effects for lower-extremity strength training across outcomes, while heterogeneity, network consistency, and estimates for the remaining modalities remained largely unchanged (Suppl. S16). For physical function, the effect estimate increased from 0.46 to 0.85, making lower-extremity strength training the highest-ranked and only statistically significant modality (Suppl. S16A). For quadriceps strength, the effect estimate increased from 0.41 to 0.61 and became statistically significant (Suppl. S16B). For pain, the effect estimate increased from 0.42 to 0.60, with minor changes in treatment rankings, although it remained non-significant (Suppl. S16C).

#### 3.5.4 Exclusion of small modalities with <20 participants

Excluding modalities with fewer than 20 participants (aerobic exercise and upper-body strength; n = 10 each) had minimal impact on the overall findings (Suppl. S17). Estimated effects, treatment rankings, heterogeneity, and network consistency remained largely unchanged across all outcomes (Suppl. S17A–D).

### 3.6 Secondary NMA at 4–8 weeks after TKA

At 4–8 weeks after TKA, the physical function and pain networks each included 11 trials (514 patients), the quadriceps strength network included 9 trials (398 patients), and the HR-QoL network included 7 trials (348 patients). Compared with the 12-14-week time point, the 4–8-week networks included an additional home-based multidomain exercise node to accommodate one three-arm trial comparing home-based and supervised multidomain exercise [68]. Lower-extremity strength training was the only modality associated with a statistically significant improvement in quadriceps strength compared with standard care (SMD 0.50, 95% CI 0.16–0.83). No significant effects were observed for physical function, pain, or HR-QoL. Heterogeneity ranged from low to moderate (I^2^ 18.6%–60.5%), and global inconsistency was detected only for physical function. Detailed NMA results are provided in Supplement S18–S30.

## 4 Discussion

To our knowledge, this is the first modality-specific NMA comparing the relative efficacy of eight prehabilitation modalities before TKA. Overall, we found no evidence that any prehabilitation modality was superior to standard care for physical function, quadriceps strength, pain, or HR-QoL at 3 months after TKA. A similar pattern was observed at 4–8 weeks after surgery, except that lower-extremity strength training was associated with improved quadriceps strength compared with standard care. Although multidomain exercise, lower-extremity strength training, and BFR exercise ranked highest for selected outcomes, the corresponding estimates remained imprecise and were supported by low-to-moderate confidence in the evidence. In contrast to previous systematic reviews, which primarily evaluated exercise-based prehabilitation as a single intervention, this NMA demonstrates the value of evaluating individual prehabilitation modalities separately, even though no modality showed clear superiority.

Several factors likely contributed to the uncertainty surrounding these findings. First, substantial clinical heterogeneity existed across trials. Although interventions were classified into eight modalities, they varied considerably in frequency, duration, intensity, supervision, and exercise dose. Incomplete reporting, particularly of exercise intensity and session duration, limited assessment of intervention dose and reproducibility, potentially contributing to residual clinical heterogeneity. Postoperative rehabilitation protocols also varied widely and were frequently reported incompletely or not at all. As postoperative outcomes likely reflect combined effects of pre- and postoperative rehabilitation, differences in postoperative care may have confounded estimated treatment effects and complicated indirect comparisons. Although statistical inconsistency was generally low, variation and incomplete reporting of potential effect modifiers raise concerns regarding the transitivity assumption. Finally, the outcome networks comprised relatively few studies distributed across multiple intervention nodes, resulting in sparse evidence and imprecise estimates. Consequently, treatment rankings should be interpreted with caution, particularly for intervention modalities supported by limited evidence. Given the small number of studies per intervention node, subgroup analyses and network meta-regression were not performed because they were unlikely to yield reliable estimates.

### 4.1 Strengths & limitations

A key strength of this review is its modality-specific NMA framework, which enabled comparison of the relative effectiveness of individual prehabilitation modalities rather than treating exercise-based prehabilitation as a single intervention. Several limitations should be considered when interpreting these findings. First, intervention reporting was frequently incomplete. Second, because most outcomes were patient-reported, the lack of participant blinding across all included trials may have introduced measurement bias, potentially leading to overestimation of treatment effects, as subjective outcomes are particularly susceptible to bias in unblinded trials [73,74]. Third, treatment effect estimates were sensitive to certain analytical decisions. One study [67] reported only adjusted model-based 95% CIs without group-level SDs and was therefore excluded from the primary analyses. Including this study increased the estimated effects and treatment rankings of lower-extremity strength training, likely because the derived SDs were implausibly small, thereby inflating the SMD estimates. Likewise, excluding studies reporting change scores strengthened the estimated effects of BFR- and multidomain exercise, as each comparison was informed by only two studies and the pooled estimate shifted toward the larger effect observed in the remaining study. These findings illustrate how treatment effect estimates in sparse-evidence networks can be sensitive to assumptions underlying variance estimation and effect-size calculation. In contrast, excluding high-risk-of-bias studies or modalities with fewer than 20 participants had little impact on the findings, supporting the robustness of the primary analyses. Fourth, reporting of postoperative complications was inadequate and inconsistent, and several studies excluded participants who developed postoperative complications [60,61,[66], [67], [68], [69]], precluding safety evaluation and potentially limiting generalizability. Fifth, most evidence was derived from comparisons with standard care, whereas direct head-to-head comparisons between prehabilitation modalities were scarce. Consequently, conclusions about the relative effectiveness of individual modalities were largely based on indirect comparisons. Finally, although funnel plots and Egger’s regression tests showed no evidence of publication bias, both methods have limited power when relatively few studies are available. Therefore, publication bias cannot be excluded, and the exclusion of grey literature may have further increased this risk.

### 4.2 Clinical implications and future research

Current evidence does not support recommending one prehabilitation modality over another in routine clinical practice. Nevertheless, lower-extremity strength training may be considered, because it demonstrated short-term improvements in quadriceps strength. However, this finding should be interpreted cautiously given the imprecision of the estimate and the low confidence in the evidence supporting this comparison.

Future research should prioritize adequately powered head-to-head RCTs comparing individual prehabilitation modalities rather than additional small trials comparing single modalities with standard care. Such studies are needed to determine whether specific approaches provide clinically meaningful advantages over others. To detect a moderate treatment effect (SMD = 0.5), approximately 150 participants would be required for a two-group comparison (allowing for 10–15% attrition) to achieve 80% statistical power, highlighting the need for substantially larger trials than those currently available. Larger studies would also improve the precision of treatment effect estimates and reduce the influence of small-study effects in future NMAs. Future exercise intervention trials should report interventions according to the Template for Intervention Description and Replication (TIDieR) checklist, including exercise dose, intensity, supervision, adherence, and intervention fidelity, to improve reproducibility and facilitate implementation [75]. Standardized reporting of postoperative rehabilitation is equally important, as postoperative outcomes likely reflect the combined effects of pre- and postoperative care. Future studies should also use standardized outcome measures and assessment time points, including early and late follow-up, to better distinguish the effects of prehabilitation from postoperative rehabilitation. Finally, adverse events and postoperative complications should be reported in accordance with the CONSORT Harms recommendations to enable balanced evaluation of both intervention effectiveness and harms [76]. Addressing these methodological limitations will strengthen the evidence base and improve our ability to determine whether specific prehabilitation modalities provide clinically meaningful short- and long-term benefits after TKA.

## 5 Conclusion

This modality-specific NMA found no evidence that one prehabilitation modality was superior to standard care for improving physical function, pain, quadriceps strength, or HR-QoL at 3 months after TKA. Lower-extremity strength training demonstrated short-term improvements in quadriceps strength, although this finding was supported by low-confidence evidence. Larger, well-designed head-to-head RCTs are needed to determine whether specific prehabilitation modalities provide clinically meaningful benefits after TKA.

## Contributions

***Monique van der Vorst:*** Conception and design; Collection and assembly of data; Analysis and interpretation of data; Drafting of the article; Critical revision of the article for important intellectual content; Statistical expertise; Final approval of the article.

***Geeske Peeters:*** Conception and design; Collection and assembly of data; Analysis and interpretation of data; Drafting of the article; Critical revision of the article for important intellectual content; Obtaining of funding; Final approval of the article.

***Gerjon Hannink:*** Analysis and interpretation of data; Drafting of the article; Critical revision of the article for important intellectual content; Statistical expertise; Final approval of the article.

***Rik Dijkman:*** Collection and assembly of data; Critical revision of the article for important intellectual content; Final approval of the article.

***Marcel Olde Rikkert:*** Conception and design; Analysis and interpretation of data; Critical revision of the article for important intellectual content; Obtaining of funding; Final approval of the article.

## Data availability statement

All data used in this study were extracted from published articles. The dataset of study characteristics and effect sizes is available from the corresponding author upon reasonable request.

## PROSPERO registration

The review protocol was registered in PROSPERO (CRD42024490615).

## Declaration of generative AI in scientific writing

The authors declare that no generative AI or AI-assisted technologies were used in the writing of this manuscript. An AI-assisted prioritization tool (ASReview, version 1.3rc1) was used exclusively to support and prioritize the title and abstract screening process by ranking records according to predicted relevance. All records were independently screened by two reviewers, and the AI tool did not replace dual human screening or decision-making.

## Funding

This work was supported by the “Academisch Medisch Netwerk” (Academic Medical Network), a regional collaboration of hospitals, including the Jeroen Bosch Hospital and the Radboud University Medical Centre in the Netherlands.

## Competing interests

The author(s) declared no potential conflicts of interest with respect to the research, authorship, and/or publication of this article.
