# Supplementary material for: Efficacy of prehabilitation modalities in total knee arthroplasty: A systematic review and network meta-analysis
Source: Osteoarthr Cartil Open. 2026 Aug 21;8(4):100863. doi: 10.1016/j.ocarto.2026.100863 (PMC13545374; doi:10.1016/j.ocarto.2026.100863)
Supplement: Supplementary file 1 [file mmc1.pdf]

Efficacy of Prehabilitation Modalities in Total Knee Arthroplasty:

A Systematic Review and Network Meta-Analysis

Supplementary Material **page**

S0. PRISMA Checklist 2

S1. Search Strategy 6

S2. Additional methodological details 17

S3. Excluded Full-Text Citations 18

S4. Assessment of Transitivity Assumption (3 Months) 23

S5. Study-Level Outcome Data and Effect Estimates (3 Months) 24

S6. Study-Level Description of Prehabilitation Interventions 26

S7. Standard Preoperative and Postoperative Care Across Individual Studies 28

S8. Forest Plots of Study-Level Pairwise Comparisons (3 Months) 29

S9. Risk of Bias Assessments at Outcome Level 33

S10. CINeMA Assessment of Network Confidence (3 Months) 35

S11. Comparison-Adjusted Funnel Plots (3 Months) 39

S12. League Tables Comparing Prehabilitation Modalities (3 Months) 40

S13. Local Inconsistency by Treatment Comparison (3 Months) 41

S14. Sensitivity Analysis: Excluding High Risk of Bias Studies (3 Months) 45

S15. Sensitivity Analysis: Excluding Change Score Studies (3 Months) 48

S16. Sensitivity Analysis: Including Model-Based Confidence Intervals (3 Months) 52

S17. Sensitivity Analysis: Excluding Small Modalities (n < 20) (3 Months) 55

NMA Outcome Assessment at 4–8 Weeks

S18. Assessment of Transitivity Assumption (4–8 Weeks) 59

S19. Network plot of prehabilitation modalities (4–8 Weeks) 60

S20. Network Forest Plots (4–8 Weeks) 61

S21. Forest Plots of Study-Level Pairwise Comparisons (4–8 Weeks) 62

S22. Probability Ranking Heatmap of Prehabilitation Modalities (4–8 Weeks) 66

S23 CINeMA Assessment of Network Confidence (4–8 Weeks) 67

S24. Comparison-Adjusted Funnel Plots (4–8 Weeks) 71

S25. League Tables Comparing Prehabilitation Modalities (4–8 Weeks) 72

S26. Local Inconsistency by Treatment Comparison (4–8 Weeks) 73

S27. Sensitivity Analysis: Excluding High Risk of Bias Studies (4–8 Weeks) 77

S28. Sensitivity Analysis: Excluding Change Score Studies (4–8 Weeks) 81

S29. Sensitivity Analysis: Including Model-Based Confidence Intervals (4–8 Weeks) 85

S30. Sensitivity Analysis: Excluding Small Modalities (n < 20) (4–8 Weeks) 88

PRISMA NMA Checklist of Items to Include When Reporting A Systematic Review Involving a Network Meta-analysis

Section/Topic	Item #	Checklist Item	Reported on Page #
**TITLE**			
Title	1	Identify the report as a systematic review *incorporating a network meta-analysis (or related form of meta-analysis).*	1
**ABSTRACT**			
Structured summary	2	Provide a structured summary including, as applicable:<br>**Background:** main objectives<br>**Methods:** data sources; study eligibility criteria, participants, and interventions; study appraisal; and *synthesis methods, such as network meta-analysis.*<br>**Results:** number of studies and participants identified; summary estimates with corresponding confidence/credible intervals; *treatment rankings may also be discussed. Authors may choose to summarize pairwise comparisons against a chosen treatment included in their analyses for brevity.*<br>**Discussion/Conclusions:** limitations; conclusions and implications of findings.<br>**Other:** primary source of funding; systematic review registration number with registry name.	2
**INTRODUCTION**			
Rationale	3	Describe the rationale for the review in the context of what is already known*, including mention of why a network meta-analysis has been conducted.*	3
Objectives	4	Provide an explicit statement of questions being addressed, with reference to participants, interventions, comparisons, outcomes, and study design (PICOS).	3
**METHODS**			
Protocol and registration	5	Indicate whether a review protocol exists and if and where it can be accessed (e.g., Web address); and, if available, provide registration information, including registration number.	4
Eligibility criteria	6	Specify study characteristics (e.g., PICOS, length of follow-up) and report characteristics (e.g., years considered, language, publication status) used as criteria for eligibility, giving rationale. *Clearly describe eligible treatments included in the treatment network, and note whether any have been clustered or merged into the same node (with justification).*	4-5
Information sources	7	Describe all information sources (e.g., databases with dates of coverage, contact with study authors to identify additional studies) in the search and date last searched.	4
Search	8	Present full electronic search strategy for at least one database, including any limits used, such that it could be repeated.	4
Study selection	9	State the process for selecting studies (i.e., screening, eligibility, included in systematic review, and, if applicable,	5

		included in the meta-analysis).	
Data collection process	10	Describe method of data extraction from reports (e.g., piloted forms, independently, in duplicate) and any processes for obtaining and confirming data from investigators.	5
Data items	11	List and define all variables for which data were sought (e.g., PICOS, funding sources) and any assumptions and simplifications made.	5
**Geometry of the network**	**S1**	Describe methods used to explore the geometry of the treatment network under study and potential biases related to it. This should include how the evidence base has been graphically summarized for presentation, and what characteristics were compiled and used to describe the evidence base to readers.	7
Risk of bias within individual studies	12	Describe methods used for assessing risk of bias of individual studies (including specification of whether this was done at the study or outcome level), and how this information is to be used in any data synthesis.	5-6
Summary measures	13	State the principal summary measures (e.g., risk ratio, difference in means). *Also describe the use of additional summary measures assessed, such as treatment rankings and surface under the cumulative ranking curve (SUCRA) values, as well as modified approaches used to present summary findings from meta-analyses.*	7
Planned methods of analysis	14	Describe the methods of handling data and combining results of studies for each network meta-analysis. This should include, but not be limited to:<br>• *Handling of multi-arm trials;*<br>• *Selection of variance structure;*<br>• *Selection of prior distributions in Bayesian analyses; and*<br>• *Assessment of model fit.*	7
**Assessment of Inconsistency**	**S2**	Describe the statistical methods used to evaluate the agreement of direct and indirect evidence in the treatment network(s) studied. Describe efforts taken to address its presence when found.	7
Risk of bias across studies	15	Specify any assessment of risk of bias that may affect the cumulative evidence (e.g., publication bias, selective reporting within studies).	5-6
Additional analyses	16	Describe methods of additional analyses if done, indicating which were pre-specified. This may include, but not be limited to, the following:<br>• Sensitivity or subgroup analyses;<br>• Meta-regression analyses;<br>• *Alternative formulations of the treatment network; and*<br>• *Use of alternative prior distributions for Bayesian analyses (if applicable).*	7

RESULTS†

Study selection	17	Give numbers of studies screened, assessed for eligibility, and included in the review, with reasons for exclusions at each stage, ideally with a flow diagram.	8
**Presentation of network structure**	**S3**	Provide a network graph of the included studies to enable visualization of the geometry of the treatment network.	Fig. 2
**Summary of network geometry**	**S4**	Provide a brief overview of characteristics of the treatment network. This may include commentary on the abundance of trials and randomized patients for the different interventions and pairwise comparisons in the network, gaps of evidence in the treatment network, and potential biases reflected by the network structure.	10-11
Study characteristics	18	For each study, present characteristics for which data were extracted (e.g., study size, PICOS, follow-up period) and provide the citations.	8-9
Risk of bias within studies	19	Present data on risk of bias of each study and, if available, any outcome level assessment.	9-10
Results of individual studies	20	For all outcomes considered (benefits or harms), present, for each study: 1) simple summary data for each intervention group, and 2) effect estimates and confidence intervals. *Modified approaches may be needed to deal with information from larger networks.*	10-11
Synthesis of results	21	Present results of each meta-analysis done, including confidence/credible intervals. *In larger networks, authors may focus on comparisons versus a particular comparator (e.g. placebo or standard care), with full findings presented in an appendix. League tables and forest plots may be considered to summarize pairwise comparisons.* If additional summary measures were explored (such as treatment rankings), these should also be presented.	10-11
**Exploration for inconsistency**	**S5**	Describe results from investigations of inconsistency. This may include such information as measures of model fit to compare consistency and inconsistency models, *P* values from statistical tests, or summary of inconsistency estimates from different parts of the treatment network.	10-11
Risk of bias across studies	22	Present results of any assessment of risk of bias across studies for the evidence base being studied.	9-10
Results of additional analyses	23	Give results of additional analyses, if done (e.g., sensitivity or subgroup analyses, meta-regression analyses, *alternative network geometries studied, alternative choice of prior distributions for Bayesian analyses,* and so forth).	12-13

DISCUSSION

Summary of evidence	24	Summarize the main findings, including the strength of evidence for each main outcome; consider their relevance to key groups (e.g., healthcare providers, users, and policy-makers).	14
Limitations	25	Discuss limitations at study and outcome level (e.g., risk of bias), and at review level (e.g., incomplete retrieval of identified research, reporting bias). *Comment on the validity of the assumptions, such as transitivity and consistency. Comment*	15-16

		on any concerns regarding network geometry (e.g., avoidance of certain comparisons).	
Conclusions	26	Provide a general interpretation of the results in the context of other evidence, and implications for future research.	17
**FUNDING**			
Funding	27	Describe sources of funding for the systematic review and other support (e.g., supply of data); role of funders for the systematic review. This should also include information regarding whether funding has been received from manufacturers of treatments in the network and/or whether some of the authors are content experts with professional conflicts of interest that could affect use of treatments in the network.	18

PICOS = population, intervention, comparators, outcomes, study design.
* Text in italics indicateS wording specific to reporting of network meta-analyses that has been added to guidance from the PRISMA statement.
† Authors may wish to plan for use of appendices to present all relevant information in full detail for items in this section.

S1. Search Strategy

Search Date: October 16th 2023

Records identified per database

Search Number	Building Block	Pubmed	CENTRAL	Embase	Web of Science	PsycINFO	MEDLINE	CINAHL	Clinical-Trials.gov	PEDRO	Other sources	Total
#1	Building block 1	134,559	19,719	175,251	133,887	1,763	132,621	48,194				
#2	Building block 2	472,505	55,598	801,723	335,512	5,733	471,925	123,296				
#3	Building block 3	2,046,022	327,263	2,956,593	5,459,830	852,670	1,874,414	873,498				
#4	Building block 4	1,123,959	155,785	3,160,141	1,445,301	138,330	1,216,165	349,704				
#5	Building block 5	926,955	113,653	1,215,064	1,572,442	498,074	925,998	506,181				
#6	Building block 6	892,171	139,053	1,022,950	904,454	652,835	890,659	376,234				
#7	(Animals[MeSH] NOT Humans[MeSH])	5,159,339										
#8	#1 AND #2	18,223	3,563	27,020	15,153	221	17,871	8,250				
#9	#3 OR #4 OR #5 OR #6	4,261,629	560,071	7,085,795	8,532,422	1,761,264	4,219,370	1,750,116				
#10	#8 AND #9	4,195	1,348	8,281	**3,487**	199	3,933	**2,056**	**13**	**432**		
#11	#11 NOT #7	**4,193**		8,101		**194**	**3,450**					
#12	CENTRAL: limt to trails		**1,345**									
#13	Embase: limit no conference abstracts and humans			**6,166**								
**Total Number of Identified Records**		**4,193**	**1,345**	**6,166**	**3,487**	**194**	**3,450**	**2,056**	**13**	**432**	**85**	**21,429**

PUBMED

Block 1: Patient population

"Arthroplasty, Replacement, Hip"[mesh] OR "Hip Prosthesis"[mesh] OR "Arthroplasty, Replacement, Knee"[mesh] OR "Knee Prosthesis"[mesh] OR "Hip implantation"[tiab] OR "Hip joint prosthes*"[tiab] OR "Hip joint replacement*"[tiab] OR "Total hip"[tiab] OR "Hipprosthes*"[tiab] OR "Femoral Head Prosthes*"[tiab] OR "Knee joint prosthes*"[tiab] OR "Knee implantation"[tiab] OR "Knee joint arthroplast*"[tiab] OR "Knee joint replacement*"[tiab] OR "Total knee"[tiab] OR "THA"[tiab] OR "THR"[tiab] OR "TKA"[tiab] OR "TKR"[tiab] OR "Hip Prosthesis"[tiab:~2] OR "Hip Replacement"[tiab:~2] OR "Hip arthroplasty"[tiab:~2] OR "Hip Prostheses"[tiab:~2] OR "Hip Replacements"[tiab:~2] OR "Hip arthroplasties"[tiab:~2] OR "Knee Prosthesis"[tiab:~2] OR "Knee Arthroplasty"[tiab:~2] OR "Knee Replacement"[tiab:~2] OR "Knee Prostheses"[tiab:~2] OR "Knee Arthroplasties"[tiab:~2] OR "Knee Replacements"[tiab:~2]

Block 2: Prehabilitation

"Preoperative Care"[mesh] OR "Preoperative Period"[mesh] OR "Preoperative Exercise"[mesh] OR "Perioperative Care"[mesh] OR "Preoperative"[tiab] OR "Pre-operative"[tiab] OR "Pre operative"[tiab] OR "presurgery"[tiab] OR "pre-surgery"[tiab] OR "pre surgery"[tiab] OR "perioperative care"[tiab] OR "prehabilitat*"[tiab] OR "prehab"[tiab] OR "pre-hab"[tiab] OR "prerehab"[tiab] OR "prerehabilitat*"[tiab] OR "prior hip surgery"[tiab:~2] OR "prior hip operation"[tiab:~2] OR "prior total hip"[tiab:~2] OR "prior hip arthroplasty"[tiab:~2] OR "prior hip arthroplasties"[tiab:~2] OR "prior hip replacement"[tiab:~2] OR "prior hip replacements"[tiab:~2] OR "prior hip prosthesis"[tiab:~2] OR "prior hip prostheses"[tiab:~2] OR "prior knee surgery"[tiab:~2] OR "prior knee operation"[tiab:~2] OR "prior total knee"[tiab:~2] OR "prior knee arthroplasty"[tiab:~2] OR "prior knee arthroplasties"[tiab:~2] OR "prior knee replacement"[tiab:~2] OR "prior knee replacements"[tiab:~2] OR "prior knee prosthesis"[tiab:~2] OR "prior knee prostheses"[tiab:~2] OR "scheduled hip operation"[tiab:~2] OR "scheduled total hip"[tiab:~2] OR "scheduled hip arthroplasty"[tiab:~2] OR "scheduled hip arthroplasties"[tiab:~2] OR "scheduled hip replacement"[tiab:~2] OR "scheduled hip replacements"[tiab:~2] OR "scheduled hip prosthesis"[tiab:~2] OR "scheduled hip prostheses"[tiab:~2] OR "scheduled knee surgery"[tiab:~2] OR "scheduled knee operation"[tiab:~2] OR "scheduled total knee"[tiab:~2] OR "scheduled knee arthroplasty"[tiab:~2] OR "scheduled knee arthroplasties"[tiab:~2] OR "scheduled knee replacement"[tiab:~2] OR "scheduled knee replacements"[tiab:~2] OR "scheduled knee prosthesis"[tiab:~2] OR "scheduled knee prostheses"[tiab:~2]

Block 3: Intervention - Exerciese
"Exercise"[mesh] OR "Muscle Strength"[mesh] OR "Exercise Therapy"[mesh] OR "Hydrotherapy"[mesh] OR "Physical Therapy Specialty"[mesh] OR "Exercise Movement Techniques"[mesh] OR "Resistance Training"[mesh] OR "Rehabilitation"[mesh] OR "Physical fitness"[mesh] OR "Gymnastics"[mesh] OR "Telerehabilitation"[mesh] OR "Musculoskeletal Manipulations"[mesh] OR "Physical Therapy Modalities"[mesh] OR "Exercise*"[tiab] OR "Training"[tiab] OR "Rehabilitation"[tiab] OR "physiotherap*"[tiab] OR "mobilisation"[tiab] OR "mobilization"[tiab] OR "kinesiotherap*"[tiab] OR "hydrotherap*"[tiab] OR "sensorimotor*"[tiab] OR "balance"[tiab] OR "proprioception"[tiab] OR "aquatic"[tiab] OR "swim"[tiab] OR "swimming"[tiab] OR "cycling"[tiab] OR "biking"[tiab] OR "bike"[tiab] OR "fitness"[tiab] OR "Gymnastic*"[tiab] OR "weight lifting"[tiab] OR "muscle strengthening"[tiab] OR "therapeutic activit*"[tiab] OR "water therap*"[tiab] OR "graded activit*"[tiab] OR "Physical therap*"[tiab] OR "Physical Activit*"[tiab] OR "Manual Therap*"[tiab]

Block 4: Intervention - Nutrition
"Diet"[mesh] OR "Diet Therapy"[mesh] OR "Nutrition Assessment"[mesh] OR "Nutritional Status"[mesh] OR "Nutrition Therapy"[mesh] OR "Feeding Behavior"[mesh] OR "Food Preferences"[mesh] OR "Weight Reduction Programs"[mesh] OR "Diet"[tiab] OR "Diets"[tiab] OR "Nutrition*"[tiab] OR "Dietary"[tiab]

Block 5: Intervention - Education
"Health Education"[mesh] OR "Self Care"[mesh] OR "Telemedicine"[mesh] OR "health promotion program*"[tiab] OR "health promotion campaign*"[tiab] OR "Education"[tiab] OR "Telemedicine"[tiab] OR "Telehealth"[tiab] OR "telemonitor*"[tiab] OR "eHealth"[tiab] OR "e-health"[tiab] OR "e-learning"[tiab] OR "m-health"[tiab] OR "telerehabilitation"[tiab] OR "instructional film*"[tiab] OR "instruction film*"[tiab] OR "interactive tutorial"[tiab]

Block 6: Intervention - Psychological
"Health Behavior"[mesh] OR "Motivation"[mesh] OR "Behavior Control"[mesh] OR "Behavior Therapy"[mesh] OR "Cognitive Behavioral Therapy"[mesh] OR "Acceptance and Commitment Therapy"[mesh] OR "Behavior Change Technique*"[tiab] OR "Behaviour Change Technique*"[tiab] OR "cognitive behavioral therap*"[tiab] OR "cognitive behavioural therap*"[tiab] OR "psychotherapy"[tiab] OR "Expectation*"[tiab] OR "Motivation*"[tiab] OR "stress reduction"[tiab] OR "psychological therap*"[tiab] OR "cognitive training"[tiab] OR "skills training"[tiab] OR "acceptance and commitment"[tiab] OR "graded exposure"[tiab] OR "sleep training"[tiab] OR "cognitive therapy"[tiab] OR "Health Behavior"[tiab] OR "Health Behaviour"[tiab] OR "Healthy Behaviour"[tiab] OR "Behavior Therap*"[tiab] OR "Behaviour Therap*"[tiab] OR "Behavioral therap*"[tiab] OR "Behavior Modification"[tiab] OR "Behaviour Modification"[tiab] OR "Conditioning Therap*"[tiab] OR "cognitive behavioral treatment*"[tiab] OR "cognitive behavioural treatment*"[tiab] OR "psychological treatment*"[tiab] OR "cognitive treatment*"[tiab] OR "Behavior treatment*"[tiab] OR "Behaviour treatment*"[tiab] OR "Behavioral treatment*"[tiab] OR "Conditioning treatment*"[tiab] OR "smoking cessation"[tiab:~2] OR "alcohol cessation"[tiab:~2] OR "quiting smoking"[tiab:~2] OR "quit smoking"[tiab:~2]

Cochrane (CENTRAL)
Block 1: Patient population
[mh "Arthroplasty, Replacement, Hip"] OR [mh "Hip Prosthesis"] OR [mh "Arthroplasty, Replacement, Knee"] OR [mh "Knee Prosthesis"] OR (Hip NEXT implantation):ti,ab,kw OR (Hip NEXT joint prosthes*):ti,ab,kw OR (Hip NEXT joint replacement*):ti,ab,kw OR (Total NEXT hip):ti,ab,kw OR (Hipprosthes*):ti,ab,kw OR (Femoral NEXT Head Prosthes*):ti,ab,kw OR (Knee NEXT joint prosthes*):ti,ab,kw OR (Knee NEXT implantation):ti,ab,kw OR (Knee NEXT joint arthroplast*):ti,ab,kw OR (Knee NEXT joint replacement*):ti,ab,kw OR (Total NEXT knee):ti,ab,kw OR (THA):ti,ab,kw OR (THR):ti,ab,kw OR (TKA):ti,ab,kw OR (TKR):ti,ab,kw OR (Hip NEAR/2 Prosthesis):ti,ab,kw OR (Hip NEAR/2 Replacement):ti,ab,kw OR (Hip NEAR/2 arthroplasty):ti,ab,kw OR (Hip NEAR/2 Prostheses):ti,ab,kw OR (Hip NEAR/2 Replacements):ti,ab,kw OR (Hip NEAR/2 arthroplasties):ti,ab,kw OR (Knee NEAR/2

Prosthesis):ti,ab,kw OR (Knee NEAR/2 Arthroplasty):ti,ab,kw OR (Knee NEAR/2 Replacement):ti,ab,kw OR (Knee NEAR/2 Prostheses):ti,ab,kw OR (Knee NEAR/2 Arthroplasties):ti,ab,kw OR (Knee NEAR/2 Replacements):ti,ab,kw OR (Hipprostheses):ti,ab,kw

Block 2: Prehabilitation
[mh "Preoperative Care"] OR [mh "Preoperative Period"] OR [mh "Preoperative Exercise"] OR [mh "Perioperative Care"] OR (Preoperative):ti,ab,kw OR ("Pre-operative"):ti,ab,kw OR ("Pre operative"):ti,ab,kw OR (presurgery):ti,ab,kw OR ("pre-surgery"):ti,ab,kw OR ("pre surgery"):ti,ab,kw OR ("perioperative care"):ti,ab,kw OR (prehabilitat*):ti,ab,kw OR (prehab):ti,ab,kw OR ("pre-hab"):ti,ab,kw OR (prerehab):ti,ab,kw OR (prerehabilitat*):ti,ab,kw OR (prior NEAR/2 hip surgery):ti,ab,kw OR (prior NEAR/2 hip operation):ti,ab,kw OR (prior NEAR/2 total hip):ti,ab,kw OR (prior NEAR/2 hip arthroplasty):ti,ab,kw OR (prior NEAR/2 hip arthroplasties):ti,ab,kw OR (prior NEAR/2 hip replacement):ti,ab,kw OR (prior NEAR/2 hip replacements):ti,ab,kw OR (prior NEAR/2 hip prosthesis):ti,ab,kw OR (prior NEAR/2 hip prostheses):ti,ab,kw OR (prior NEAR/2 knee surgery):ti,ab,kw OR (prior NEAR/2 knee operation):ti,ab,kw OR (prior NEAR/2 total knee):ti,ab,kw OR (prior NEAR/2 knee arthroplasty):ti,ab,kw OR (prior NEAR/2 knee arthroplasties):ti,ab,kw OR (prior NEAR/2 knee replacement):ti,ab,kw OR (prior NEAR/2 knee replacements):ti,ab,kw OR (prior NEAR/2 knee prosthesis):ti,ab,kw OR (prior NEAR/2 knee prostheses):ti,ab,kw OR (scheduled NEAR/2 hip operation):ti,ab,kw OR (scheduled NEAR/2 total hip):ti,ab,kw OR (scheduled NEAR/2 hip arthroplasty):ti,ab,kw OR (scheduled NEAR/2 hip arthroplasties):ti,ab,kw OR (scheduled NEAR/2 hip replacement):ti,ab,kw OR (scheduled NEAR/2 hip replacements):ti,ab,kw OR (scheduled NEAR/2 hip prosthesis):ti,ab,kw OR (scheduled NEAR/2 hip prostheses):ti,ab,kw OR (scheduled NEAR/2 knee surgery):ti,ab,kw OR (scheduled NEAR/2 knee operation):ti,ab,kw OR (scheduled NEAR/2 total knee):ti,ab,kw OR (scheduled NEAR/2 knee arthroplasty):ti,ab,kw OR (scheduled NEAR/2 knee arthroplasties):ti,ab,kw OR (scheduled NEAR/2 knee replacement):ti,ab,kw OR (scheduled NEAR/2 knee replacements):ti,ab,kw OR (scheduled NEAR/2 knee prosthesis):ti,ab,kw OR (scheduled NEAR/2 knee prostheses):ti,ab,kw

Block 3: Intervention - Exerciese
[mh "Exercise"] OR [mh "Muscle Strength"] OR [mh "Exercise Therapy"] OR [mh "Hydrotherapy"] OR [mh "Physical Therapy Specialty"] OR [mh "Exercise Movement Techniques"] OR [mh "Resistance Training"] OR [mh "Rehabilitation"] OR [mh "Physical fitness"] OR [mh "Gymnastics"] OR [mh "Telerehabilitation"] OR [mh "Musculoskeletal Manipulations"] OR [mh "Physical Therapy Modalities"] OR (Exercise*):ti,ab,kw OR (Training):ti,ab,kw OR (Rehabilitation):ti,ab,kw OR (physiotherap*):ti,ab,kw OR (mobilisation):ti,ab,kw OR (mobilization):ti,ab,kw OR (kinesiotherap*):ti,ab,kw OR (hydrotherap*):ti,ab,kw OR (sensorimotor*):ti,ab,kw OR (balance):ti,ab,kw OR (proprioception):ti,ab,kw OR (aquatic):ti,ab,kw OR (swim):ti,ab,kw OR (swimming):ti,ab,kw OR (cycling):ti,ab,kw OR (biking):ti,ab,kw OR (bike):ti,ab,kw OR (fitness):ti,ab,kw OR (Gymnastic*):ti,ab,kw OR (weight NEXT lifting):ti,ab,kw OR (muscle NEXT strengthening):ti,ab,kw OR (therapeutic NEXT activit*):ti,ab,kw OR (water NEXT therap*):ti,ab,kw OR (graded NEXT activit*):ti,ab,kw OR (Physical NEXT therap*):ti,ab,kw OR (Physical NEXT Activit*):ti,ab,kw OR (Manual Therap*):ti,ab,kw

Block 4: Intervention - Nutrition
[mh "Diet"] OR [mh "Diet Therapy"] OR [mh "Nutrition Assessment"] OR [mh "Nutritional Status"] OR [mh "Nutrition Therapy"] OR [mh "Feeding Behavior"] OR [mh "Food Preferences"] OR [mh "Weight Reduction Programs"] OR (Diet):ti,ab,kw OR (Diets):ti,ab,kw OR (Nutrition*):ti,ab,kw OR (Dietary):ti,ab,kw OR (weight NEAR/1 reduction):ti,ab,kw OR (weight NEAR/1 loss):ti,ab,kw

Block 5: Intervention - Education
[mh "Health Education"] OR [mh "Self Care"] OR [mh "Telemedicine"] OR (health NEXT promotion program*):ti,ab,kw OR (health NEXT promotion campaign*):ti,ab,kw OR (Education):ti,ab,kw OR (Telemedicine):ti,ab,kw OR (Telehealth):ti,ab,kw OR (telemonitor*):ti,ab,kw OR (eHealth):ti,ab,kw OR (e-health):ti,ab,kw OR (e-learning):ti,ab,kw OR (m-health):ti,ab,kw OR (telerehabilitation):ti,ab,kw OR (instructional film*):ti,ab,kw OR (instruction film*):ti,ab,kw OR (interactive tutorial):ti,ab,kw

Block 6: Intervention - Psychological
[mh "Health Behavior"] OR [mh "Motivation"] OR [mh "Behavior Control"] OR [mh "Behavior Therapy"] OR [mh "Cognitive Behavioral Therapy"] OR [mh "Acceptance and Commitment Therapy"] OR (Behavior NEXT Change Technique*):ti,ab,kw OR (Behaviour NEXT Change Technique*):ti,ab,kw OR (cognitive NEXT behavioral therap*):ti,ab,kw OR (cognitive NEXT behavioural therap*):ti,ab,kw OR (psychotherapy):ti,ab,kw OR (Expectation*):ti,ab,kw OR (Motivation*):ti,ab,kw OR (stress NEXT reduction):ti,ab,kw OR (psychological NEXT therapy):ti,ab,kw OR (psychological NEXT therapies):ti,ab,kw OR (cognitive NEXT training):ti,ab,kw OR (skills NEXT training):ti,ab,kw OR (acceptance NEAR/1 commitment):ti,ab,kw OR (graded NEXT exposure):ti,ab,kw OR (sleep NEXT training):ti,ab,kw OR (cognitive NEXT therapy):ti,ab,kw OR (Health NEXT Behavior):ti,ab,kw OR (Health NEXT Behaviour):ti,ab,kw OR (Healthy NEXT Behaviour):ti,ab,kw OR (Behavior NEXT Therap*):ti,ab,kw OR (Behaviour NEXT Therap*):ti,ab,kw OR (Behavioral NEXT therap*):ti,ab,kw OR (Behavior NEXT Modification):ti,ab,kw OR (Behaviour NEXT Modification):ti,ab,kw OR (Conditioning NEXT Therap*):ti,ab,kw OR (smoking NEAR/1 cessation):ti,ab,kw OR (alcohol NEAR/1 cessation):ti,ab,kw OR (cognitive NEXT behavioral treatment*):ti,ab,kw OR (cognitive NEXT behavioural treatment*):ti,ab,kw OR (psychological NEXT treatment*):ti,ab,kw OR (cognitive NEXT treatment*):ti,ab,kw OR (Behavior NEXT treatment*):ti,ab,kw OR (Behaviour NEXT treatment*):ti,ab,kw OR (Behavioral NEXT treatment*):ti,ab,kw OR (Conditioning NEXT treatment*):ti,ab,kw OR (quiting NEAR/1 smoking):ti,ab,kw OR (quit NEAR/1 smoking):ti,ab,kw

EMBASE
Block 1: Patient population
exp hip arthroplasty/ OR exp hip prosthesis/ OR exp knee arthroplasty/ OR exp knee prosthesis/ OR (Hip ADJ1 implantation).ti,ab,kf OR (Hip ADJ1 joint prosthes*).ti,ab,kf OR (Hip ADJ1 joint replacement*).ti,ab,kf OR (Total ADJ1 hip).ti,ab,kf OR (Hipprosthes*).ti,ab,kf OR (Femoral Head Prosthes*).ti,ab,kf OR (Knee ADJ1 joint prosthes*).ti,ab,kf OR (Knee ADJ1 implantation).ti,ab,kf OR (Knee ADJ1 joint arthroplast*).ti,ab,kf OR (Knee ADJ1 joint replacement*).ti,ab,kf OR (Total ADJ1 knee).ti,ab,kf OR (THA).ti,ab,kf OR (THR).ti,ab,kf OR (TKA).ti,ab,kf OR (TKR).ti,ab,kf OR (Hip ADJ2 Prosthesis).ti,ab,kf OR (Hip ADJ2 Replacement).ti,ab,kf OR (Hip ADJ2 arthroplasty).ti,ab,kf OR (Hip ADJ2 Prostheses).ti,ab,kf OR (Hip ADJ2 Replacements).ti,ab,kf OR (Hip ADJ2 arthroplasties).ti,ab,kf OR (Knee ADJ2 Prosthesis).ti,ab,kf OR (Knee ADJ2 Arthroplasty).ti,ab,kf OR (Knee ADJ2 Replacement).ti,ab,kf OR (Knee ADJ2 Prostheses).ti,ab,kf OR (Knee ADJ2 Arthroplasties).ti,ab,kf OR (Knee ADJ2 Replacements).ti,ab,kf

Block 2: Prehabilitation
exp Preoperative care/ OR exp preoperative period/ OR exp preoperative exercise/ OR exp perioperative care/ OR (Preoperative).ti,ab,kf OR (Pre-operative).ti,ab,kf OR (Pre ADJ1 operative).ti,ab,kf OR (presurgery).ti,ab,kf OR (pre-surgery).ti,ab,kf OR (pre AJD1 surgery).ti,ab,kf OR (perioperative ADJ1 care).ti,ab,kf OR (prehabilitat*).ti,ab,kf OR (prehab).ti,ab,kf OR (pre-hab).ti,ab,kf OR (prerehab).ti,ab,kf OR (prerehabilitat*).ti,ab,kf OR (prior ADJ2 hip surgery).ti,ab,kf OR (prior ADJ2 hip operation).ti,ab,kf OR (prior ADJ2 total hip).ti,ab,kf OR (prior ADJ2 hip arthroplasty).ti,ab,kf OR (prior ADJ2 hip arthroplasties).ti,ab,kf OR (prior ADJ2 hip replacement).ti,ab,kf OR (prior ADJ2 hip replacements).ti,ab,kf OR (prior ADJ2 hip prosthesis).ti,ab,kf OR (prior ADJ2 hip prostheses).ti,ab,kf OR (prior ADJ2 knee surgery).ti,ab,kf OR (prior ADJ2 knee operation).ti,ab,kf OR (prior ADJ2 total knee).ti,ab,kf OR (prior ADJ2 knee arthroplasty).ti,ab,kf OR (prior ADJ2 knee arthroplasties).ti,ab,kf OR (prior ADJ2 knee replacement).ti,ab,kf OR (prior ADJ2 knee replacements).ti,ab,kf OR (prior ADJ2 knee prosthesis).ti,ab,kf OR (prior ADJ2 knee prostheses).ti,ab,kf OR (scheduled ADJ2 hip operation).ti,ab,kf OR (scheduled ADJ2 total hip).ti,ab,kf OR (scheduled ADJ2 hip arthroplasty).ti,ab,kf OR (scheduled ADJ2 hip arthroplasties).ti,ab,kf OR (scheduled ADJ2 hip replacement).ti,ab,kf OR (scheduled ADJ2 hip replacements).ti,ab,kf OR (scheduled ADJ2 hip prosthesis).ti,ab,kf OR (scheduled ADJ2 hip prostheses).ti,ab,kf OR (scheduled ADJ2 knee surgery).ti,ab,kf OR (scheduled ADJ2 knee operation).ti,ab,kf OR (scheduled ADJ2 total knee).ti,ab,kf OR (scheduled ADJ2 knee arthroplasty).ti,ab,kf OR (scheduled ADJ2 knee arthroplasties).ti,ab,kf OR (scheduled ADJ2 knee replacement).ti,ab,kf OR (scheduled ADJ2 knee replacements).ti,ab,kf OR (scheduled ADJ2 knee prosthesis).ti,ab,kf OR (scheduled ADJ2 knee prostheses).ti,ab,kf

Block 3: Intervention - Exerciese
exp Exercise/ OR exp muscle strength/ OR exp kinesiotherapy/ OR exp hydrotherapy/ OR exp physiotherapy/ OR exp Physical activity/ OR exp resistance training/ OR exp Rehabilitation/ OR exp fitness/ OR exp gymnastics/ OR exp telerehabilitation/ OR exp musculoskeletal manipulation/ OR (Exercise*).ti,ab,kf OR (Training).ti,ab,kf OR (Rehabilitation).ti,ab,kf OR (physiotherap*).ti,ab,kf OR (mobilisation).ti,ab,kf OR (mobilization).ti,ab,kf OR (kinesiotherap*).ti,ab,kf OR (hydrotherap*).ti,ab,kf OR (sensorimotor*).ti,ab,kf OR (balance).ti,ab,kf OR (proprioception).ti,ab,kf OR (aquatic).ti,ab,kf OR (swim).ti,ab,kf OR (swimming).ti,ab,kf OR (cycling).ti,ab,kf OR (biking).ti,ab,kf OR (bike).ti,ab,kf OR (fitness).ti,ab,kf OR (Gymnastic*).ti,ab,kf OR (weight lifting).ti,ab,kf OR (muscle strengthening).ti,ab,kf OR (therapeutic activit*).ti,ab,kf OR (water therap*).ti,ab,kf OR (graded activit*).ti,ab,kf OR (Physical therap*).ti,ab,kf OR (Physical Activit*).ti,ab,kf OR (Manual Therap*).ti,ab,kf

Block 4: Intervention - Nutrition
exp diet/ OR exp diet therapy/ OR exp nutritional assessment/ OR exp nutritional status/ OR exp nutrition/ OR exp food preference/ OR exp feeding behavior/ OR exp caloric intake/ OR exp weight loss program/ OR (Diet).ti,ab,kf OR (Diets).ti,ab,kf OR (Nutrition*).ti,ab,kf OR (Dietary).ti,ab,kf OR (weight ADJ1 reduction).ti,ab,kf OR (weight ADJ1 loss).ti,ab,kf

Block 5: Intervention - Education
exp health education/ OR exp nutrition education/ OR exp self care/ OR exp telemedicine/ OR (health promotion program*).ti,ab,kf OR (health promotion campaign*).ti,ab,kf OR (Education).ti,ab,kf OR (Telemedicine).ti,ab,kf OR (Telehealth).ti,ab,kf OR (telemonitor*).ti,ab,kf OR (eHealth).ti,ab,kf OR (e-health).ti,ab,kf OR (e-learning).ti,ab,kf OR (m-health).ti,ab,kf OR (telerehabilitation).ti,ab,kf OR (instructional film*).ti,ab,kf OR (instruction film*).ti,ab,kf OR (interactive tutorial).ti,ab,kf

Block 6: Intervention - Psychological
exp health behavior/ OR exp motivation/ OR exp behavior control/ OR exp behavior therapy/ OR exp cognitive behavioral therapy/ OR (Behavior Change Technique*).ti,ab,kf OR (Behaviour Change Technique*).ti,ab,kf OR (cognitive behavioral therap*).ti,ab,kf OR (cognitive behavioural therap*).ti,ab,kf OR (psychotherapy).ti,ab,kf OR (Expectation*).ti,ab,kf OR (Motivation*).ti,ab,kf OR (stress reduction).ti,ab,kf OR (psychological therap*).ti,ab,kf OR (cognitive training).ti,ab,kf OR (skills training).ti,ab,kf OR (acceptance ADJ1 commitment).ti,ab,kf OR (graded exposure).ti,ab,kf OR (sleep training).ti,ab,kf OR (cognitive therapy).ti,ab,kf OR (Health Behavior).ti,ab,kf OR (Health Behaviour).ti,ab,kf OR (Healthy Behaviour).ti,ab,kf OR (Behavior Therap*).ti,ab,kf OR (Behaviour Therap*).ti,ab,kf OR (Behavioral therap*).ti,ab,kf OR (Behavior Modification).ti,ab,kf OR (Behaviour Modification).ti,ab,kf OR (Conditioning Therap*).ti,ab,kf OR (smoking ADJ1 cessation).ti,ab,kf OR (alcohol ADJ1 cessation).ti,ab,kf OR (cognitive behavioral treatment*).ti,ab,kf OR (cognitive behavioural treatment*).ti,ab,kf OR (psychological treatment*).ti,ab,kf OR (cognitive treatment*).ti,ab,kf OR (Behavior treatment*).ti,ab,kf OR (Behaviour treatment*).ti,ab,kf OR (Behavioral treatment*).ti,ab,kf OR (Conditioning treatment*).ti,ab,kf OR (quiting ADJ1 smoking).ti,ab,kf OR (quit ADJ1 smoking).ti,ab,kf

WEB OF SCIENCE
Block 1: Patient population
TS = ("Hip implantation") OR TS = ("Hip joint prosthes*") OR TS = ("Hip joint replacement*") OR TS = ("Total hip") OR TS = (Hipprosthes*) OR TS = ("Femoral Head Prosthes*") OR TS = ("Knee joint prosthes*") OR TS = ("Knee implantation") OR TS = ("Knee joint arthroplast*") OR TS = ("Knee joint replacement*") OR TS = ("Total knee") OR TS = (THA) OR TS = (THR) OR TS = (TKA) OR TS = (TKR) OR TS = (Hip NEAR/2 Prosthesis) OR TS = (Hip NEAR/2 Replacement) OR TS = (Hip NEAR/2 arthroplasty) OR TS = (Hip NEAR/2 Prostheses) OR TS = (Hip NEAR/2 Replacements) OR TS = (Hip NEAR/2 arthroplasties) OR TS = (Knee NEAR/2 Prosthesis) OR TS = (Knee NEAR/2 Arthroplasty) OR TS = (Knee NEAR/2 Replacement) OR TS = (Knee NEAR/2 Prostheses) OR TS = (Knee NEAR/2 Arthroplasties) OR TS = (Knee NEAR/2 Replacements)

Block 2: Prehabilitation

TS = (Preoperative) OR TS = ("Pre-operative") OR TS = ("Pre operative") OR TS = (presurgery) OR TS = ("pre-surgery") OR TS = ("pre surgery") OR TS = ("perioperative care") OR TS = (prehabilitat*) OR TS = (prehab) OR TS = ("pre-hab") OR TS = (prerehab) OR TS = (prerehabilitat*) OR TS = (prior NEAR/2 hip surgery) OR TS = (prior NEAR/2 hip operation) OR TS = (prior NEAR/2 total hip) OR TS = (prior NEAR/2 hip arthroplasty) OR TS = (prior NEAR/2 hip arthroplasties) OR TS = (prior NEAR/2 hip replacement) OR TS = (prior NEAR/2 hip replacements) OR TS = (prior NEAR/2 hip prosthesis) OR TS = (prior NEAR/2 hip prostheses) OR TS = (prior NEAR/2 knee surgery) OR TS = (prior NEAR/2 knee operation) OR TS = (prior NEAR/2 total knee) OR TS = (prior NEAR/2 knee arthroplasty) OR TS = (prior NEAR/2 knee arthroplasties) OR TS = (prior NEAR/2 knee replacement) OR TS = (prior NEAR/2 knee replacements) OR TS = (prior NEAR/2 knee prosthesis) OR TS = (prior NEAR/2 knee prostheses) OR TS = (scheduled NEAR/2 hip operation) OR TS = (scheduled NEAR/2 total hip) OR TS = (scheduled NEAR/2 hip arthroplasty) OR TS = (scheduled NEAR/2 hip arthroplasties) OR TS = (scheduled NEAR/2 hip replacement) OR TS = (scheduled NEAR/2 hip replacements) OR TS = (scheduled NEAR/2 hip prosthesis) OR TS = (scheduled NEAR/2 hip prostheses) OR TS = (scheduled NEAR/2 knee surgery) OR TS = (scheduled NEAR/2 knee operation) OR TS = (scheduled NEAR/2 total knee) OR TS = (scheduled NEAR/2 knee arthroplasty) OR TS = (scheduled NEAR/2 knee arthroplasties) OR TS = (scheduled NEAR/2 knee replacement) OR TS = (scheduled NEAR/2 knee replacements) OR TS = (scheduled NEAR/2 knee prosthesis) OR TS = (scheduled NEAR/2 knee prostheses)

Block 3: Intervention - Exerciese

TS = (Exercise*) OR TS = (Training) OR TS = (Rehabilitation) OR TS = (physiotherap*) OR TS = (mobilisation) OR TS = (mobilization) OR TS = (kinesiotherap*) OR TS = (hydrotherap*) OR TS = (sensorimotor*) OR TS = (balance) OR TS = (proprioception) OR TS = (aquatic) OR TS = (swim) OR TS = (swimming) OR TS = (cycling) OR TS = (biking) OR TS = (bike) OR TS = (fitness) OR TS = (Gymnastic*) OR TS = ("weight lifting") OR TS = ("muscle strengthening") OR TS = ("therapeutic activit*") OR TS = ("water therap*") OR TS = ("graded activit*") OR TS = ("Physical therap*") OR TS = ("Physical Activit*") OR TS = ("Manual Therap*")

Block 4: Intervention - Nutrition

TS = (Diet) OR TS = (Diets) OR TS = (Nutrition*) OR TS = (Dietary) OR TS = (weight NEAR/1 reduction) OR TS = (weight NEAR/1 loss)

Block 5: Intervention - Education

TS = ("health promotion program*") OR TS = ("health promotion campaign*") OR TS = (Education) OR TS = (Telemedicine) OR TS = (Telehealth) OR TS = (telemonitor*) OR TS = (eHealth) OR TS = ("e-health") OR TS = ("e-learning") OR TS = ("m-health") OR TS = (telerehabilitation) OR TS = ("instructional film*") OR TS = ("instruction film*") OR TS = ("interactive tutorial")

Block 6: Intervention - Psychological

TS = ("Behavior Change Technique*") OR TS = ("Behaviour Change Technique*") OR TS = ("cognitive behavioral therap*") OR TS = ("cognitive behavioural therap*") OR TS = (psychotherapy) OR TS = (Expectation*) OR TS = (Motivation*) OR TS = ("stress reduction") OR TS = ("psychological therap*") OR TS = ("cognitive training") OR TS = ("skills training") OR TS = (acceptance NEAR/1 commitment) OR TS = ("graded exposure") OR TS = ("sleep training") OR TS = ("cognitive therapy") OR TS = ("Health Behavior") OR TS = ("Health Behaviour") OR TS = ("Healthy Behaviour") OR TS = ("Behavior Therap*") OR TS = ("Behaviour Therap*") OR TS = ("Behavioral therap*") OR TS = ("Behavior Modification") OR TS = ("Behaviour Modification") OR TS = ("Conditioning Therap*") OR TS = (smoking NEAR/1 cessation) OR TS = (alcohol NEAR/1 cessation) OR TS = ("cognitive behavioral treatment*") OR TS = ("cognitive behavioural treatment*") OR TS = ("psychological treatment*") OR TS = ("cognitive treatment*") OR TS = ("Behavior treatment*") OR TS = ("Behaviour treatment*") OR TS = ("Behavioral treatment*") OR TS = ("Conditioning treatment*") OR TS = (quiting NEAR/1 smoking) OR TS = (quit NEAR/1 smoking)

PSYCINFO

Block 1: Patient population

(Hip implantation).tw. OR (Hip joint prosthes*).tw. OR (Hip joint replacement*).tw. OR (Total hip).tw. OR (Hipprosthes*).tw. OR (Femoral Head Prosthes*).tw. OR (Knee joint prosthes*).tw. OR (Knee implantation).tw. OR (Knee joint arthroplast*).tw. OR (Knee joint replacement*).tw. OR (Total knee).tw. OR (THA).tw. OR (THR).tw. OR (TKA).tw. OR (TKR).tw. OR (Hip Prosthesis).tw. OR (Hip Replacement).tw. OR (Hip arthroplasty).tw. OR (Hip Prostheses).tw. OR (Hip Replacements).tw. OR (Hip arthroplasties).tw. OR (Knee Prosthesis).tw. OR (Knee Arthroplasty).tw. OR (Knee Replacement).tw. OR (Knee Prostheses).tw. OR (Knee Arthroplasties).tw. OR (Knee Replacements).tw.

Block 2: Prehabilitation

(Preoperative).tw. OR (Pre-operative).tw. OR (Pre operative).tw. OR (presurgery).tw. OR (pre-surgery).tw. OR (pre surgery).tw. OR (perioperative care).tw. OR (prehabilitat*).tw. OR (prehab).tw. OR (pre-hab).tw. OR (prerehab).tw. OR (prerehabilitat*).tw. OR (prior to hip surgery).tw. OR (prior to hip operation).tw. OR (prior to total hip).tw. OR (prior to hip arthroplasty).tw. OR (prior to hip arthroplasties).tw. OR (prior to hip replacement).tw. OR (prior to hip replacements).tw. OR (prior to hip prosthesis).tw. OR (prior to hip prostheses).tw. OR (prior to knee surgery).tw. OR (prior to knee operation).tw. OR (prior to total knee).tw. OR (prior to knee arthroplasty).tw. OR (prior to knee arthroplasties).tw. OR (prior to knee replacement).tw. OR (prior to knee replacements).tw. OR (prior to knee prosthesis).tw. OR (prior to knee prostheses).tw.

Block 3: Intervention - Exerciese

exp Exercise/ OR exp Aerobic Exercise/ OR exp physical strength/ OR exp physical treatment methods/ OR exp physical therapy/ OR exp athletic training/ OR exp rehabilitation/ OR exp physical fitness/ OR exp telerehabilitation/ OR (Exercise*).tw. OR (Training).tw. OR (Rehabilitation).tw. OR (physiotherap*).tw. OR (mobilisation).tw. OR (mobilization).tw. OR (kinesiotherap*).tw. OR (hydrotherap*).tw. OR (sensorimotor*).tw. OR (balance).tw. OR (proprioception).tw. OR (aquatic).tw. OR (swim).tw. OR (swimming).tw. OR (cycling).tw. OR (biking).tw. OR (bike).tw. OR (fitness).tw. OR (Gymnastic*).tw. OR (weight lifting).tw. OR (muscle strengthening).tw. OR (therapeutic activit*).tw. OR (water therap*).tw. OR (graded activit*).tw. OR (Physical therap*).tw. OR (Physical Activit*).tw. OR (Manual Therap*).tw.

Block 4: Intervention - Nutrition

exp Diets/ OR exp nutrition/ OR exp weight control/ OR exp eating behavior/ OR exp food preferences/ OR exp weight loss/ OR (Diet).tw. OR (Diets).tw. OR (Nutrition*).tw. OR (Dietary).tw. OR (weight reduction).tw. OR (weight loss).tw. OR (loss of weight).tw.

Block 5: Intervention - Education

exp health behavior/ OR exp Self-Care/ OR exp Telemedicine/ OR (health promotion program*).tw. OR (health promotion campaign*).tw. OR (Education).tw. OR (Telemedicine).tw. OR (Telehealth).tw. OR (telemonitor*).tw. OR (eHealth).tw. OR (e-health).tw. OR (e-learning).tw. OR (m-health).tw. OR (telerehabilitation).tw. OR (instructional film*).tw. OR (instruction film*).tw. OR (interactive tutorial).tw.

Block 6: Intervention - Psychological

exp health behavior/ OR exp motivation/ OR exp behavior therapy/ OR exp cognitive behavior therapy/ OR exp "acceptance and commitment therapy"/ OR exp smoking cessation/ OR (Behavior Change Technique*).tw. OR (Behaviour Change Technique*).tw. OR (cognitive behavioral therap*).tw. OR (cognitive behavioural therap*).tw. OR (psychotherapy).tw. OR (Expectation*).tw. OR (Motivation*).tw. OR (stress reduction).tw. OR (psychological therap*).tw. OR (cognitive training).tw. OR (skills training).tw. OR (acceptance and commitment).tw. OR (graded exposure).tw. OR (sleep training).tw. OR (cognitive therapy).tw. OR (Health Behavior).tw. OR (Health Behaviour).tw. OR (Healthy Behaviour).tw. OR (Behavior Therap*).tw. OR (Behaviour Therap*).tw. OR (Behavioral therap*).tw. OR (Behavior Modification).tw. OR (Behaviour Modification).tw. OR (Conditioning Therap*).tw. OR (smoking cessation).tw. OR (alcohol cessation).tw. OR (cognitive behavioral treatment*).tw. OR (cognitive behavioural treatment*).tw. OR (psychological

treatment*).tw. OR (cognitive treatment*).tw. OR (Behavior treatment*).tw. OR (Behaviour treatment*).tw. OR (Behavioral treatment*).tw. OR (Conditioning treatment*).tw. OR (quiting smoking).tw. OR (quit smoking).tw.

MEDLINE

Block 1: Patient population

exp Arthroplasty, Replacement, Hip/ OR exp Arthroplasty, Replacement, Knee/ OR exp Hip Prosthesis/ OR exp Knee Prosthesis/ OR (Hip implantation).ti,ab,kf OR (Hip joint prosthes*).ti,ab,kf OR (Hip joint replacement*).ti,ab,kf OR (Total hip).ti,ab,kf OR (Hipprosthes*).ti,ab,kf OR (Femoral Head Prosthes*).ti,ab,kf OR (Knee joint prosthes*).ti,ab,kf OR (Knee implantation).ti,ab,kf OR (Knee joint arthroplast*).ti,ab,kf OR (Knee joint replacement*).ti,ab,kf OR (Total knee).ti,ab,kf OR (THA).ti,ab,kf OR (THR).ti,ab,kf OR (TKA).ti,ab,kf OR (TKR).ti,ab,kf OR (Hip ADJ2 Prosthesis).ti,ab,kf OR (Hip ADJ2 Replacement).ti,ab,kf OR (Hip ADJ2 arthroplasty).ti,ab,kf OR (Hip ADJ2 Prostheses).ti,ab,kf OR (Hip ADJ2 Replacements).ti,ab,kf OR (Hip ADJ2 arthroplasties).ti,ab,kf OR (Knee ADJ2 Prosthesis).ti,ab,kf OR (Knee ADJ2 Arthroplasty).ti,ab,kf OR (Knee ADJ2 Replacement).ti,ab,kf OR (Knee ADJ2 Prostheses).ti,ab,kf OR (Knee ADJ2 Arthroplasties).ti,ab,kf OR (Knee ADJ2 Replacements).ti,ab,kf

Block 2: Prehabilitation

exp Preoperative care/ OR exp preoperative period/ OR exp preoperative exercise/ OR exp perioperative care/ OR (Preoperative).ti,ab,kf OR (Pre-operative).ti,ab,kf OR (Pre operative).ti,ab,kf OR (presurgery).ti,ab,kf OR (pre-surgery).ti,ab,kf OR (pre surgery).ti,ab,kf OR (perioperative care).ti,ab,kf OR (prehabilitat*).ti,ab,kf OR (prehab).ti,ab,kf OR (pre-hab).ti,ab,kf OR (prerehab).ti,ab,kf OR (prerehabilitat*).ti,ab,kf OR (prior ADJ2 hip surgery).ti,ab,kf OR (prior ADJ2 hip operation).ti,ab,kf OR (prior ADJ2 total hip).ti,ab,kf OR (prior ADJ2 hip arthroplasty).ti,ab,kf OR (prior ADJ2 hip arthroplasties).ti,ab,kf OR (prior ADJ2 hip replacement).ti,ab,kf OR (prior ADJ2 hip replacements).ti,ab,kf OR (prior ADJ2 hip prosthesis).ti,ab,kf OR (prior ADJ2 hip prostheses).ti,ab,kf OR (prior ADJ2 knee surgery).ti,ab,kf OR (prior ADJ2 knee operation).ti,ab,kf OR (prior ADJ2 total knee).ti,ab,kf OR (prior ADJ2 knee arthroplasty).ti,ab,kf OR (prior ADJ2 knee arthroplasties).ti,ab,kf OR (prior ADJ2 knee replacement).ti,ab,kf OR (prior ADJ2 knee replacements).ti,ab,kf OR (prior ADJ2 knee prosthesis).ti,ab,kf OR (prior ADJ2 knee prostheses).ti,ab,kf OR (scheduled ADJ2 hip operation).ti,ab,kf OR (scheduled ADJ2 total hip).ti,ab,kf OR (scheduled ADJ2 hip arthroplasty).ti,ab,kf OR (scheduled ADJ2 hip arthroplasties).ti,ab,kf OR (scheduled ADJ2 hip replacement).ti,ab,kf OR (scheduled ADJ2 hip replacements).ti,ab,kf OR (scheduled ADJ2 hip prosthesis).ti,ab,kf OR (scheduled ADJ2 hip prostheses).ti,ab,kf OR (scheduled ADJ2 knee surgery).ti,ab,kf OR (scheduled ADJ2 knee operation).ti,ab,kf OR (scheduled ADJ2 total knee).ti,ab,kf OR (scheduled ADJ2 knee arthroplasty).ti,ab,kf OR (scheduled ADJ2 knee arthroplasties).ti,ab,kf OR (scheduled ADJ2 knee replacement).ti,ab,kf OR (scheduled ADJ2 knee replacements).ti,ab,kf OR (scheduled ADJ2 knee prosthesis).ti,ab,kf OR (scheduled ADJ2 knee prostheses).ti,ab,kf

Block 3: Intervention - Exerciese

exp Exercise/ OR exp muscle strength/ OR exp hydrotherapy/ OR exp resistance training/ OR exp resistance training/ OR exp physical fitness/ OR exp Cardiorespiratory Fitness/ OR exp gymnastics/ OR exp Telerehabilitation/ OR exp Musculoskeletal Manipulations/ OR (Exercise*).ti,ab,kf OR (Training).ti,ab,kf OR (Rehabilitation).ti,ab,kf OR (physiotherap*).ti,ab,kf OR (mobilisation).ti,ab,kf OR (mobilization).ti,ab,kf OR (kinesiotherap*).ti,ab,kf OR (hydrotherap*).ti,ab,kf OR (sensorimotor*).ti,ab,kf OR (balance).ti,ab,kf OR (proprioception).ti,ab,kf OR (aquatic).ti,ab,kf OR (swim).ti,ab,kf OR (swimming).ti,ab,kf OR (cycling).ti,ab,kf OR (biking).ti,ab,kf OR (bike).ti,ab,kf OR (fitness).ti,ab,kf OR (Gymnastic*).ti,ab,kf OR (weight lifting).ti,ab,kf OR (muscle strengthening).ti,ab,kf OR (therapeutic activit*).ti,ab,kf OR (water therap*).ti,ab,kf OR (graded activit*).ti,ab,kf OR (Physical therap*).ti,ab,kf OR (Physical Activit*).ti,ab,kf OR (Manual Therap*).ti,ab,kf

Block 4: Intervention - Nutrition

exp Diet/ OR exp nutrition assessment/ OR exp Nutrition Therapy/ OR exp feeding behavior/ OR exp food preference/ OR exp nutritional status/ OR exp Energy Intake/ OR exp Weight Reduction Programs/ OR exp

weight loss/ OR (Diet).ti,ab,kf OR (Diets).ti,ab,kf OR (Nutrition*).ti,ab,kf OR (Dietary).ti,ab,kf OR (weight reduction).ti,ab,kf OR (weight loss).ti,ab,kf OR (loss of weight).ti,ab,kf

Block 5: Intervention - Education
exp health education/ OR exp self care/ OR exp telemedicine/ OR (health promotion program*).ti,ab,kf OR (health promotion campaign*).ti,ab,kf OR (Education).ti,ab,kf OR (Telemedicine).ti,ab,kf OR (Telehealth).ti,ab,kf OR (telemonitor*).ti,ab,kf OR (eHealth).ti,ab,kf OR (e-health).ti,ab,kf OR (e-learning).ti,ab,kf OR (m-health).ti,ab,kf OR (telerehabilitation).ti,ab,kf OR (instructional film*).ti,ab,kf OR (instruction film*).ti,ab,kf OR (interactive tutorial).ti,ab,kf

Block 6: Intervention - Psychological
exp health behavior/ OR exp motivation/ OR exp behavior control/ OR exp behavior therapy/ OR exp cognitive behavioral therapy/ OR (Behavior Change Technique*).ti,ab,kf OR (Behaviour Change Technique*).ti,ab,kf OR (cognitive behavioral therap*).ti,ab,kf OR (cognitive behavioural therap*).ti,ab,kf OR (psychotherapy).ti,ab,kf OR (Expectation*).ti,ab,kf OR (Motivation*).ti,ab,kf OR (stress reduction).ti,ab,kf OR (psychological therap*).ti,ab,kf OR (cognitive training).ti,ab,kf OR (skills training).ti,ab,kf OR (acceptance ADJ1 commitment).ti,ab,kf OR (graded exposure).ti,ab,kf OR (sleep training).ti,ab,kf OR (cognitive therapy).ti,ab,kf OR (Health Behavior).ti,ab,kf OR (Health Behaviour).ti,ab,kf OR (Healthy Behaviour).ti,ab,kf OR (Behavior Therap*).ti,ab,kf OR (Behaviour Therap*).ti,ab,kf OR (Behavioral therap*).ti,ab,kf OR (Behavior Modification).ti,ab,kf OR (Behaviour Modification).ti,ab,kf OR (Conditioning Therap*).ti,ab,kf OR (smoking ADJ1 cessation).ti,ab,kf OR (alcohol ADJ1 cessation).ti,ab,kf OR (cognitive behavioral treatment*).ti,ab,kf OR (cognitive behavioural treatment*).ti,ab,kf OR (psychological treatment*).ti,ab,kf OR (cognitive treatment*).ti,ab,kf OR (Behavior treatment*).ti,ab,kf OR (Behaviour treatment*).ti,ab,kf OR (Behavioral treatment*).ti,ab,kf OR (Conditioning treatment*).ti,ab,kf OR (quiting ADJ1 smoking).ti,ab,kf OR (quit ADJ1 smoking).ti,ab,kf

CINAHL
Block 1: Patient population
(MH "Arthroplasty, Replacement, Hip+") OR (MH "Arthroplasty, Replacement, Knee+") OR (TI "Hip implantation" OR AB "Hip implantation") OR (TI "Hip joint prosthes*" OR AB "Hip joint prosthes*") OR (TI "Hip joint replacement*" OR AB "Hip joint replacement*") OR (TI "Total hip" OR AB "Total hip") OR (TI "Hipprosthes*" OR AB "Hipprosthes*") OR (TI "Femoral Head Prosthes*" OR AB "Femoral Head Prosthes*") OR (TI "Knee joint prosthes*" OR AB "Knee joint prosthes*") OR (TI "Knee implantation" OR AB "Knee implantation") OR (TI "Knee joint arthroplast*" OR AB "Knee joint arthroplast*") OR (TI "Knee joint replacement*" OR AB "Knee joint replacement*") OR (TI "Total knee" OR AB "Total knee") OR (TI "THA" OR AB "THA") OR (TI "THR" OR AB "THR") OR (TI "TKA" OR AB "TKA") OR (TI "TKR" OR AB "TKR") OR (TI "Hip Prosthes*" OR AB "Hip Prosthes*") OR (TI "Hip Replacement*" OR AB "Hip Replacement*") OR (TI "Hip arthroplast*" OR AB "Hip arthroplast*") OR (TI "Knee Prosthes*" OR AB "Knee Prosthes*") OR (TI "Knee Arthroplast*" OR AB "Knee Arthroplast*") OR (TI "Knee Replacement*" OR AB "Knee Replacement*")

Block 2: Prehabilitation
(MH "Preoperative Care+") OR (MH "Prehabilitation+") OR (MH "Perioperative Care+") OR (TI "Preoperative" OR AB "Preoperative") OR (TI "Pre-operative" OR AB "Pre-operative") OR (TI "Pre operative" OR AB "Pre operative") OR (TI "presurgery" OR AB "presurgery") OR (TI "pre-surgery" OR AB "pre-surgery") OR (TI "pre surgery" OR AB "pre surgery") OR (TI "perioperative care" OR AB "perioperative care") OR (TI "prehabilitat*" OR AB "prehabilitat*") OR (TI "prehab" OR AB "prehab") OR (TI "pre-hab" OR AB "pre-hab") OR (TI "prerehab" OR AB "prerehab") OR (TI "prerehabilitat*" OR AB "prerehabilitat*") OR (TI "prior hip surgery" OR AB "prior hip surgery") OR (TI "prior hip operation" OR AB "prior hip operation") OR (TI "prior total hip" OR AB "prior total hip") OR (TI "prior hip arthroplasty" OR AB "prior hip arthroplasty") OR (TI "prior hip arthroplasties" OR AB "prior hip arthroplasties") OR (TI "prior hip replacement" OR AB "prior hip replacement") OR (TI "prior hip replacements" OR AB "prior hip replacements") OR (TI "prior hip prosthesis"

OR AB "prior hip prosthesis") OR (TI "prior hip prostheses" OR AB "prior hip prostheses") OR (TI "prior knee surgery" OR AB "prior knee surgery") OR (TI "prior knee operation" OR AB "prior knee operation") OR (TI "prior total knee" OR AB "prior total knee") OR (TI "prior knee arthroplasty" OR AB "prior knee arthroplasty") OR (TI "prior knee arthroplasties" OR AB "prior knee arthroplasties") OR (TI "prior knee replacement" OR AB "prior knee replacement") OR (TI "prior knee replacements" OR AB "prior knee replacements") OR (TI "prior knee prosthesis" OR AB "prior knee prosthesis") OR (TI "prior knee prostheses" OR AB "prior knee prostheses") OR (TI "scheduled hip operation" OR AB "scheduled hip operation") OR (TI "scheduled total hip" OR AB "scheduled total hip") OR (TI "scheduled hip arthroplasty" OR AB "scheduled hip arthroplasty") OR (TI "scheduled hip arthroplasties" OR AB "scheduled hip arthroplasties") OR (TI "scheduled hip replacement" OR AB "scheduled hip replacement") OR (TI "scheduled hip replacements" OR AB "scheduled hip replacements") OR (TI "scheduled hip prosthesis" OR AB "scheduled hip prosthesis") OR (TI "scheduled hip prostheses" OR AB "scheduled hip prostheses") OR (TI "scheduled knee surgery" OR AB "scheduled knee surgery") OR (TI "scheduled knee operation" OR AB "scheduled knee operation") OR (TI "scheduled total knee" OR AB "scheduled total knee") OR (TI "scheduled knee arthroplasty" OR AB "scheduled knee arthroplasty") OR (TI "scheduled knee arthroplasties" OR AB "scheduled knee arthroplasties") OR (TI "scheduled knee replacement" OR AB "scheduled knee replacement") OR (TI "scheduled knee replacements" OR AB "scheduled knee replacements") OR (TI "scheduled knee prosthesis" OR AB "scheduled knee prosthesis") OR (TI "scheduled knee prostheses" OR AB "scheduled knee prostheses")

Block 3: Intervention - Exerciese
(MH "Exercise+") OR (MH "Muscle Strength+") OR (MH "Physical Therapy+") OR (MH "Therapeutic Exercise+") OR (MH "Hydrotherapy+") OR (MH "Resistance Training+") OR (MH "Rehabilitation+") OR (MH "Physical fitness+") OR (MH "Gymnastics+") OR (MH "Telerehabilitation+") OR (TI "Exercise*" OR AB "Exercise*") OR (TI "Training" OR AB "Training") OR (TI "Rehabilitation" OR AB "Rehabilitation") OR (TI "physiotherap*" OR AB "physiotherap*") OR (TI "mobilisation" OR AB "mobilisation") OR (TI "mobilization" OR AB "mobilization") OR (TI "kinesiotherap*" OR AB "kinesiotherap*") OR (TI "hydrotherap*" OR AB "hydrotherap*") OR (TI "sensorimotor*" OR AB "sensorimotor*") OR (TI "balance" OR AB "balance") OR (TI "proprioception" OR AB "proprioception") OR (TI "aquatic" OR AB "aquatic") OR (TI "swim" OR AB "swim") OR (TI "swimming" OR AB "swimming") OR (TI "cycling" OR AB "cycling") OR (TI "biking" OR AB "biking") OR (TI "bike" OR AB "bike") OR (TI "fitness" OR AB "fitness") OR (TI "Gymnastic*" OR AB "Gymnastic*") OR (TI "weight lifting" OR AB "weight lifting") OR (TI "muscle strengthening" OR AB "muscle strengthening") OR (TI "therapeutic activit*" OR AB "therapeutic activit*") OR (TI "water therap*" OR AB "water therap*") OR (TI "graded activit*" OR AB "graded activit*") OR (TI "Physical therap*" OR AB "Physical therap*") OR (TI "Physical Activit*" OR AB "Physical Activit*") OR (TI "Manual Therap*" OR AB "Manual Therap*")

Block 4: Intervention - Nutrition
(MH "Diet+") OR (MH "Diet Therapy+") OR (MH "Nutritional Assessment+") OR (MH "Nutritional Status+") OR (MH "Diet Therapy+") OR (MH "Eating Behavior+") OR (MH "Food Preferences+") OR (MH "Weight Reduction Programs+") OR (TI "Diet" OR AB "Diet") OR (TI "Diets" OR AB "Diets") OR (TI "Nutrition*" OR AB "Nutrition*") OR (TI "Dietary" OR AB "Dietary") OR (TI "weight reduction" OR AB "weight reduction") OR (TI "weight loss" OR AB "weight loss") OR (TI "loss of weight" OR AB "loss of weight")

Block 5: Intervention - Education
(MH "Preoperative Education+") OR (MH "Health Education+") OR (MH "Self Care+") OR (MH "Telemedicine+") OR (TI "health promotion program*" OR AB "health promotion program*") OR (TI "health promotion campaign*" OR AB "health promotion campaign*") OR (TI "Education" OR AB "Education") OR (TI "Telemedicine" OR AB "Telemedicine") OR (TI "Telehealth" OR AB "Telehealth") OR (TI "telemonitor*" OR AB "telemonitor*") OR (TI "eHealth" OR AB "eHealth") OR (TI "e-health" OR AB "e-health") OR (TI "e-learning" OR AB "e-learning") OR (TI "m-health" OR AB "m-health") OR (TI "telerehabilitation" OR AB "telerehabilitation") OR (TI "instructional film*" OR AB "instructional film*") OR (TI "instruction film*" OR AB "instruction film*") OR (TI "interactive tutorial" OR AB "interactive tutorial")

Block 6: Intervention - Psychological

(MH "Health Behavior+") OR (MH "Motivation+") OR (MH "Behavioral Changes+") OR (MH "Behavior Therapy+") OR (MH "Cognitie Therapy+") OR (MH "Acceptance and Commitment Therapy+") OR (TI "Behavior Change Technique*" OR AB "Behavior Change Technique*") OR (TI "Behaviour Change Technique*" OR AB "Behaviour Change Technique*") OR (TI "cognitive behavioral therap*" OR AB "cognitive behavioral therap*") OR (TI "cognitive behavioural therap*" OR AB "cognitive behavioural therap*") OR (TI "psychotherapy" OR AB "psychotherapy") OR (TI "Expectation*" OR AB "Expectation*") OR (TI "Motivation*" OR AB "Motivation*") OR (TI "stress reduction" OR AB "stress reduction") OR (TI "psychological therap*" OR AB "psychological therap*") OR (TI "cognitive training" OR AB "cognitive training") OR (TI "skills training" OR AB "skills training") OR (TI "acceptance and commitment" OR AB "acceptance and commitment") OR (TI "graded exposure" OR AB "graded exposure") OR (TI "sleep training" OR AB "sleep training") OR (TI "cognitive therapy" OR AB "cognitive therapy") OR (TI "Health Behavior" OR AB "Health Behavior") OR (TI "Health Behaviour" OR AB "Health Behaviour") OR (TI "Healthy Behaviour" OR AB "Healthy Behaviour") OR (TI "Behavior Therap*" OR AB "Behavior Therap*") OR (TI "Behaviour Therap*" OR AB "Behaviour Therap*") OR (TI "Behavioral therap*" OR AB "Behavioral therap*") OR (TI "Behavior Modification" OR AB "Behavior Modification") OR (TI "Behaviour Modification" OR AB "Behaviour Modification") OR (TI "Conditioning Therap*" OR AB "Conditioning Therap*") OR (TI "smoking cessation" OR AB "smoking cessation") OR (TI "alcohol cessation" OR AB "alcohol cessation") OR (TI "cognitive behavioral treatment*" OR AB "cognitive behavioral treatment*") OR (TI "cognitive behavioural treatment*" OR AB "cognitive behavioural treatment*") OR (TI "psychological treatment*" OR AB "psychological treatment*") OR (TI "cognitive treatment*" OR AB "cognitive treatment*") OR (TI "Behavior treatment*" OR AB "Behavior treatment*") OR (TI "Behaviour treatment*" OR AB "Behaviour treatment*") OR (TI "Behavioral treatment*" OR AB "Behavioral treatment*") OR (TI "Conditioning treatment*" OR AB "Conditioning treatment*") OR (TI "quiting smoking" OR AB "quiting smoking") OR (TI "quit smoking" OR AB "quit smoking")

S2. Additional methodological details

S2.1 Eligible outcome measures

Outcome	Eligible outcome measure
Physical function	WOMAC Function, KOOS ADL, or SF-36 Physical Functioning.
Quadriceps strength	Any validated assessment of quadriceps muscle strength.
Pain	WOMAC Pain, KOOS Pain, or SF-36 Bodily Pain.
Health-related quality of life	KOOS Quality of Life (QoL) or SF-36 Physical Component Score (PCS).

Abbreviations: ADL, Activities of Daily Living; KOOS, Knee Injury and Osteoarthritis Outcome Score; PCS, Physical Component Summary; SF-36, 36-Item Short Form Health Survey; WOMAC, Western Ontario and McMaster Universities Osteoarthritis Index.

S2.2 Software used for study selection and data extraction

The following software was used during the review process:

- *Covidence* (Veritas Health Innovation, Melbourne, Australia) for study selection and data extraction.
- *ASReview* (version 1.3rc1) to prioritize records based on predicted relevance during title and abstract screening.
- *WebPlotDigitizer* (https://plotdigitizer.com/app) to extract numerical outcome data from published figures when required.

S2.3 Risk of bias and confidence in the evidence

Confidence in network estimates was assessed using the CINeMA framework, which evaluates six domains:

- Within-study bias
- Reporting bias
- Indirectness
- Imprecision
- Heterogeneity
- Incoherence

S2.4 Statistical Analysis

All statistical analyses were performed in *R* (version 4.4.1; R Foundation for Statistical Computing, Vienna, Austria) using the netmeta package.

S3. Excluded Full-Text Citations

Study	Exclusion Reason
**Al-Heizan MO**, Shoman A, Tawffeq A, et al. The Efficacy of a Preoperative Occupational Therapy Educational Session for Saudi Patients Undergoing a Lower Extremity Joint Replacement. *J Multidiscip Healthc*. 2023;16:31-38. doi:10.2147/JMDH.S394761	Not an RCT
**Alghadir A**, Iqbal ZA, Anwer S. Comparison of the effect of pre- and post-operative physical therapy versus post-operative physical therapy alone on pain and recovery of function after total knee arthroplasty. *J Phys Ther Sci*. 2016;28(10):2754-2758. doi:10.1589/jpts.28.2754	Ineligible intervention
**Aytekin E**, Sukur, et al. The effect of a 12 week prehabilitation program on pain and function for patients undergoing total knee arthroplasty: A prospective controlled study. *J Clin Orthop Trauma*. 2019;10(2):345-349. doi:10.1016/j.jcot.2018.04.006	Not an RCT
**Berge JD**, Dolin, et al. Pre-operative and post-operative effect of a pain management programme prior to total hip replacement: a randomized controlled trial. *Pain*. 2004;110(1-2):33-9.	THA patients only
**Biau JD**, Porcher, et al. Neither pre-operative education or a minimally invasive procedure have any influence on the recovery time after total hip replacement. *International orthopaedics*. 2015;39(8):1475-81. doi:https://dx.doi.org/10.1007/s00264-015-2802-y	Not an RCT
**Bitterli R**, Sieben, et al. Pre-surgical sensorimotor training for patients undergoing total hip replacement: a randomised controlled trial. *International journal of sports medicine*. 2011;32(9):725-32. doi:https://dx.doi.org/10.1055/s-0031-1271696	THA patients only
**Brosky T**, Topp R, Finley M, et al. Effects of prehabilitation on early rehabilitation outcomes following total knee arthroplasty in patients with knee osteoarthritis. 2011;97:eS160. doi:10.1016/j.physio.2011.04.002	Conference abstract
**Brown AK,** Comparing pre- and post-surgical self-efficacy behavior changes by introducing prehabilitation exercise. University of Louisville; 2010.	Not an RCT
**Börjesson M**, Robertson, et al. Physiotherapy in knee osteoarthrosis: effect on pain and walking. *Physiother Res Int*. 1996;1(2):89-97. doi:10.1002/pri.6120010205	Ineligible patient population
**Cavill S**, McKenzie K, Munro A, et al. The effect of prehabilitation on the range of motion and functional outcomes in patients following the total knee or hip arthroplasty: A pilot randomized trial. *Physiotherapy theory and practice*. 2016;32(4):262-70. doi:https://dx.doi.org/10.3109/09593985.2016.1138174	Different postoperative care
**Chen**, Su-Ru, Chen, Chyang-Shiong, Lin, Pi-Chu. The effect of educational intervention on the pain and rehabilitation performance of patients who undergo a total knee replacement. *Journal of clinical nursing*. 2014;23(1-2):279-87. doi:https://dx.doi.org/10.1111/jocn.12466	Not an RCT
**Chen W**, Sun, et al. Cognitive behavioral therapy cannot relieve postoperative pain and improve joint function after total knee arthroplasty in patients aged 70 years and older. *Aging clinical and experimental research*. 2021;33(12):3293-3302. doi:https://dx.doi.org/10.1007/s40520-021-01870-7	Different postoperative care
**Clode NJ**, Perry MA, Wulff L. Does physiotherapy prehabilitation improve pre-surgical outcomes and influence patient expectations prior to knee and hip joint arthroplasty? *International journal of orthopaedic and trauma nursing.* 2018;30(101528681):14-19. doi:https://dx.doi.org/10.1016/j.ijotn.2018.05.004	Not an RCT
**Cooke M**, Walker R, Aitken LM, Freeman A, Pavey S, Cantrill R. Pre-operative self-efficacy education vs. usual care for patients undergoing joint replacement surgery: a pilot randomised controlled trial. *Scandinavian journal of caring sciences*. 2016;30(1):74-82. doi:https://dx.doi.org/10.1111/scs.12223	Combined TKA/THA analysis
**Crotty M**, Prendergast, et al. Self-management and peer support among people with arthritis on a hospital joint replacement waiting list: a randomised controlled trial. *Osteoarthritis Cartilage*. 2009;17(11):1428-33. doi:10.1016/j.joca.2009.05.010	Combined TKA/THA analysis
**Dlima DD**, Colwell, et al. The effect of preoperative exercise on total knee replacement outcomes. *Clinical orthopaedics and related research.* 1996; (326):174-82.	Ineligible outcomes
**Daltroy LH**, Morlino CI, Eaton HM, Poss R, Liang MH. Preoperative education for total hip and knee replacement patients. *Arthritis care and research : the official journal of the Arthritis Health Professions Association.* 1998;11(6):469-78.	Combined TKA/THA analysis
**Doering S**, Katzlberger, et al. Videotape preparation of patients before hip replacement surgery reduces stress. *Psychosomatic medicine.* 2000;62(3):365-73.	THA patients only
**Doiron-Cadrin P**, Kairy, et al. Feasibility and preliminary effects of a tele-prehabilitation program and an in-person prehablitation program compared to usual care for total hip or knee arthroplasty candidates: a pilot randomized controlled trial. *Disability and rehabilitation*. 2020;42(7):989-998. doi:https://dx.doi.org/10.1080/09638288.2018.1515992	No eligible follow-up time point
**Du D**, Li H, Xu Y, et al. Study on the Effect of Pain Programmed Care Based on the Concept of Prehabilitation on the Recovery of Joint Function and WHOQOL-BREF Score in Elderly Patients after Total Hip Arthroplasty *Altern Ther Health Med.* 2023;	Different postoperative care
**Evgeniadis G**, Beneka, et al. Effects of pre- or postoperative therapeutic exercise on the quality of life, before and after total knee arthroplasty for osteoarthritis. *Journal of Back and Musculoskeletal Rehabilitation.* 2008;21(3):161-169. doi:https://dx.doi.org/10.3233/BMR-2008-21303	Different postoperative care

Study	Exclusion Reason
**Faurschou P**, Villadsen B. Effects of patient training. *Danish Journal of Nursing / Sygeplejersken*. 107(6):50-55.	Not an RCT
**Fernandes L**, Roos, et al. Supervised neuromuscular exercise prior to hip and knee replacement: 12-month clinical effect and cost-utility analysis alongside a randomised controlled trial. *BMC musculoskeletal disorders*. 2017;18(1):5. doi:https://dx.doi.org/10.1186/s12891-016-1369-0	Combined TKA/THA analysis
**Ferrara EP**, Rabini, et al. Effect of pre-operative physiotherapy in patients with end-stage osteoarthritis undergoing hip arthroplasty. Clinical Rehabilitation. 2008;22(10-11):977-986. doi:10.1177/0269215508094714	THA patients only
**Fu S**, Wang, et al. The efficacy of nursing intervention to reduce preoperative anxiety in patients with total knee arthroplasty: A protocol of prospective randomized trial. *Medicine*. 2020;99(38):e22213. doi:https://dx.doi.org/10.1097/MD.0000000000022213	Clinical trial protocol
**Gilbey HJ,** Ackland TR, Tapper J, Wang AW. Perioperative exercise improves function following total hip arthroplasty: A randomized controlled trial. *Journal of Musculoskeletal Research*. 2003;7(2):111-123. doi:https://dx.doi.org/10.1142/S0218957703001046	THA patients only
**Gilbey HJ**, Ackland TR, Wang AW, Morton AR, Trouchet T, Tapper J. Exercise improves early functional recovery after total hip arthroplasty. *Clinical orthopaedics and related research.* 2003;(408):193-200.	THA patients only
**Gill DS**, McBurney H, Schulz, LD. Land-based versus pool-based exercise for people awaiting joint replacement surgery of the hip or knee: results of a randomized controlled trial. *Archives of physical medicine and rehabilitation*. 2009;90(3):388-94. doi:https://dx.doi.org/10.1016/j.apmr.2008.09.561	Combined TKA/THA analysis
**Giraudet-Le Quintrec J-S**, Coste J, Vastel L, et al. Positive effect of patient education for hip surgery: a randomized trial. *Clinical orthopaedics and related research.* 2003;(414):112-20.	THA patients only
**Gocen Z**, Sen A, Unver B, Karatosun V, Gunal I. The effect of preoperative physiotherapy and education on the outcome of total hip replacement: a prospective randomized controlled trial. *Clinical rehabilitation.* 2004;18(4):353-8.	THA patients only
**Grottanelli S,** The relevance to the pre-surgery information to the patient subjected to hip arthroplasty. *Scienza Riabilitativa*. 2007;9(1):13-22.	Not an RCT
**Gränicher P**, Stöggl, et al. Preoperative exercise in patients undergoing total knee arthroplasty: a pilot randomized controlled trial. *Arch Physiother.* 2020;10(NA):13. doi:10.1186/s40945-020-00085-9	Ineligible outcomes
**Hanley WA**, Gililland J, Garland LE. To Be Mindful of the Breath or Pain: Comparing Two Brief Preoperative Mindfulness Techniques for Total Joint Arthroplasty Patients. Journal of Consulting and Clinical Psychology. 2021;89(7):590-600. doi:10.1037/ccp0000657	Combined TKA/THA analysis
**Hanley AW,** Gililland J, Erickson J, et al. Brief preoperative mind-body therapies for total joint arthroplasty patients: a randomized controlled trial. *Pain*. 2021;162(6):1749-1757. doi:https://dx.doi.org/10.1097/j.pain.0000000000002195	Combined TKA/THA analysis
**Hansen TB**, Bredtoft KH, Larsen K. Preoperative physical optimization in fast-track hip and knee arthroplasty. *Danish medical journal.* 2012;59(2):A4381.	Not an RCT
**Hermann A.**, Holsgaard-Larsen, et al. Preoperative resistance training increases muscle function in patients diagnosed with hip osteoarthritis scheduled for total hip arthroplasty: A-randomized explorative trial. 2013;21:S149-S150.	Conference abstract
**Hermann A**, Holsgaard-Larsen A, Zerahn B, Mejdahl S, Overgaard S. Preoperative effects of progressive explosive type resistance training in patients with ostheoarthritis schedueled for total hip arthroplasty – a prospective randomized clinical trial. *Ostheoarthritis and Cartilage*. 2014;22:S454-S455. doi:10.1016/j.joca.2014.02.862	Conference abstract
**Hermann A**, Holsgaard-Larsen A, Zerahn B, Mejdahl S, Overgaard S. Preoperative progressive explosive-type resistance training is feasible and effective in patients with hip osteoarthritis scheduled for total hip arthroplasty-- a randomized controlled trial. *Osteoarthritis Cartilage*. 2016 Jan;24(1):91-8. doi: 10.1016/j.joca.2015.07.030.	THA patients only
**Holsgaard-Larsen A**, Hermann, et al. Effects of progressive resistance training prior to total HIP arthroplasty - a secondary analysis of a randomized controlled trial. *Osteoarthritis and cartilage*. 2020;28(8):1038-1045. doi:https://dx.doi.org/10.1016/j.joca.2020.04.010	THA patients only
**Hoogeboom TJ**, Dronkers JJ, van den Ende CHM, Oosting E, van Meeteren NLU. Preoperative therapeutic exercise in frail elderly scheduled for total hip replacement: a randomized pilot trial. *Clinical rehabilitation*. 2010;24(10):901-10. doi:https://dx.doi.org/10.1177/0269215510371427	THA patients only
**Huang WS**, Chen HP, Chou HY. Effects of a preoperative simplified home rehabilitation education program on length of stay of total knee arthroplasty patients. *Orthopaedics & traumatology, surgery & research: OTSR.* 2012;98(3):259-64. doi:https://dx.doi.org/10.1016/j.otsr.2011.12.004	No eligible follow-up time point
**Huber OE**, de Bie RA, et al. Effect of pre-operative neuromuscular training on functional outcome after total knee replacement: a randomized-controlled trial. *BMC musculoskeletal disorders*. 2013;14(100968565):157. doi:https://dx.doi.org/10.1186/1471-2474-14-157	Clinical trial protocol
**Husted RS**, Troelsen, et al. Efficacy of pre-operative quadriceps strength training on knee-extensor strength before and shortly following total knee arthroplasty: protocol for a randomized, dose-response trial (The QUADX-1 trial). *Trials*. 2018;19(1):47. doi:https://dx.doi.org/10.1186/s13063-017-2366-9	Clinical trial protocol

Study	Exclusion Reason
**Ikeda T**, Jinno, et al. Effect of exercise therapy combined with branched-chain amino acid supplementation on muscle strengthening in persons with osteoarthritis. Hong Kong Physiother J. 2018;38(1):23-31. doi:10.1142/S1013702518500038	THA patients only
**Irct2014111619980N**. Effect of educational program on pain intensity and range of motion in patients undergoing joint replacement. 2015	Clinical trial protocol
**Ismail ME**, Shukry M, Sharifudin, et al. Preoperative physiotherapy and short-term functional outcomes of primary total knee arthroplasty. *Singapore medical journal.* 2016;57(3):138-43. doi:https://dx.doi.org/10.11622/smedj.2016055	Bilateral TKA patients
**Jahic D**, Omerovic, et al. The Effect of Prehabilitation on Postoperative Outcome in Patients Following Primary Total Knee Arthroplasty. *Medical archives (Sarajevo, Bosnia and Herzegovina)*. 2018;72(6):439-443. doi:https://dx.doi.org/10.5455/medarh.2018.72.439-443	Ineligible outcomes
**Janardhan TP**, Ram PR, Narayan P, Karun CSS. Functional Outcome of Preoperative Exercises on Range of Movements Following TKA: A Prospective Comparative Study. *Travmatologyia / Orthopediya Rossi//*. 2023;29(1):17-24. doi:10.17816/2311-2905-2014	Ineligible outcomes
**Karihtala T**, Heinonen, et al. Effects of preoperative group-based aquatic training on health related quality of life in persons with late stage knee osteoarthritis. 2015;101:eS723. doi:10.1016/j.physio.2015.03.3580	Conference abstract
**Kaya Ç**, Bilik O. Effect of Counseling on Quality of Life and Self-Care Agency for Patients Who are Scheduled for Total Knee Replacement. *Clinical nursing research.* 2022;31(3):519-529. doi:https://dx.doi.org/10.1177/10547738211058985	Not an RCT
**Kennedy M.** A pilot randomized control trial of aerobic cycling before total knee arthroplasty. 2014;22: S460.	Conference abstract
**Kim S**, Hsu, et al. A pilot study of aquatic prehabilitation in adults with knee osteoarthritis undergoing total knee arthroplasty - short term outcome. *BMC musculoskeletal disorders.* 2021;22(1):388. doi:https://dx.doi.org/10.1186/s12891-021-04253-1	Ineligible patient population
**Kosek E**, Roos, et al. Increased pain sensitivity but normal function of exercise induced analgesia in hip and knee osteoarthritis--treatment effects of neuromuscular exercise and total joint replacement. *Osteoarthritis and cartilage.* 2013;21(9):1299-307. doi:https://dx.doi.org/10.1016/j.joca.2013.06.019	Not an RCT
**Lluch E**, Dueñas L, Falla D, et al. Preoperative Pain Neuroscience Education Combined With Knee Joint Mobilization for Knee Osteoarthritis: A Randomized Controlled Trial. *Clin J Pain*. Jan 2018;34(1):44-52. doi:10.1097/ajp.0000000000000511	Ineligible outcomes
**Louw A**, Puentedura, et al. A controlled clinical trial of preoperative pain neuroscience education for patients about to undergo total knee arthroplasty. *Clinical rehabilitation.* 2019;33(11):1722-1731. doi:https://dx.doi.org/10.1177/0269215519857782	Not an RCT
**March KM**, Harmer, et al. The KOMPACT-P study: Knee Osteoarthritis Management with Physiotherapy informed by Acceptance and Commitment Therapy-Pilot study protocol. *BMJ open.* 2020;10(6):e032675. doi:https://dx.doi.org/10.1136/bmjopen-2019-032675	Clinical trial protocol
**Matassi F**, Duerinckx, et al. Range of motion after total knee arthroplasty: the effect of a preoperative home exercise program. *Knee surgery, sports traumatology, arthroscopy.* 2014;22(3):703-9. doi:https://dx.doi.org/10.1007/s00167-012-2349-z	Ineligible outcomes
**McHugh G.** The role of perhabilitation on the outcome of total knee arthroplasty: A randomized control trial. University College Dublin (Ireland); 2011.	Not an RCT
**McKay C**, Prapavessis, H. The Effect of a Strength-training Prehabilitation Intervention on Postoperative Outcomes of Total Knee Arthroplasty. *Medicine and Science in Sports and Exercise*. 2012;44:627-627.	Conference abstract
**Moe HR**, Dagfinrud H. A neuromuscular exercise program prior to hip or knee arthroplasty does not improve recovery of function three months after surgery. *Journal of physiotherapy.* 2014;60(1):59. doi:https://dx.doi.org/10.1016/j.jphys.2013.12.009	Not an RCT
**Moller MA**, Villebro, et al. Effect of preoperative smoking intervention on postoperative complications: a randomised clinical trial. *Lancet (London, England).* 2002;359(9301):114-7.	Combined TKA/THA analysis
**Murray WE.** A preoperative smoking intervention decreased postoperative complications in elective knee or hip replacement. *Evidence Based Nursing.* 2002;5(3):84.	Not an RCT
**Nguyen C**, Boutron I, Roren A, et al. Effect of Prehabilitation Before Total Knee Replacement for Knee Osteoarthritis on Functional Outcomes: A Randomized Clinical Trial. *JAMA network open.* 2022;5(3):e221462. doi:https://dx.doi.org/10.1001/jamanetworkopen.2022.1462	No eligible follow-up time point
**Nuñez M**, Nuñez, et al. The effect of an educational program to improve health-related quality of life in patients with osteoarthritis on waiting list for total knee replacement: a randomized study. *Osteoarthritis Cartilage*. 2006;14(3):279-85. doi:10.1016/j.joca.2005.10.002	No eligible follow-up time point
**Ohno C**, Ogawa T, Taniguchi T, et al. Effect of 3-week preoperative rehabilitation on pain and daily physical activities in patients with severe osteoarthritis undergoing total knee arthroplasty. *Br J Pain.* 2022;16(5):472-480. doi:10.1177/20494637221084190	Bilateral TKA patients
**Oosting E**, Jans MP, Dronkers JJ, et al. Preoperative home-based physical therapy versus usual care to improve functional health of frail older adults scheduled for elective total hip arthroplasty: a pilot randomized controlled trial. *Archives of physical medicine and rehabilitation.* 2012;93(4):610-6. doi:https://dx.doi.org/10.1016/j.apmr.2011.11.006	THA patients only
**Parsons G**, Jester, R, Godfrey, H. A randomised controlled trial to evaluate the efficacy of a health maintenance clinic intervention for patients undergoing elective primary total hip and knee replacement surgery. *International Journal of Orthopaedic & Trauma Nursing.* 2013;17(4):171-179. doi:10.1016/j.ijotn.2013.07.004	Combined TKA/THA analysis

Study	Exclusion Reason
**Percope de Andrade MA**, Silva MdA, et al. A new methodology for patient education in total knee arthroplasty: a randomized controlled trial. *European Journal of Orthopaedic Surgery and Traumatology*. 2022;32(1):107-112. doi:https://dx.doi.org/10.1007/s00590-021-02936-y	No eligible follow-up time point
**Petersen, KM,** Madsen, et al. Efficacy of multimodal optimization of mobilization and nutrition in patients undergoing hip replacement: a randomized clinical trial. *Acta anaesthesiologica Scandinavica.* 2006;50(6):712-7.	Different postoperative care
**Pinskiy M**, Lubovsky O, Kalichman L. The effect of a preoperative physical therapy education program on short-term outcomes of patients undergoing elective total hip arthroplasty: A controlled prospective clinical trial. *Acta orthopaedica et traumatologica turcica*. 2021;55(4):306-310.	Not an RCT
**Przkora R**, Sibille K, Victor S, et al. Feasibility of Blood Flow Restriction Exercise Prehabilitation to Attenuate Postoperative Loss of Function after Total Knee Replacement: A Randomized Pilot Study. *Anesthesia and Analgesia.* 2021;132(5S_SUPPL):868-869.	Conference abstract
**Przkora, R**, Sibille, et al. Blood flow restriction exercise to attenuate postoperative loss of function after total knee replacement: a randomized pilot study. *Eur J Transl Myol.* 2021;31(3). doi:10.4081/ejtm.2021.9932	No eligible follow-up time point
**Rezzan G**, Marinelli, et al. Efficacy of action observation pre-operative training in functional recovery after hip and knee prosthesis. 2015;101:eS1502?eS1503. doi:10.1016/j.physio.2015.03.1483	Conference abstract
**Riddle LD**, Keefe, et al. Pain coping skills training for patients with elevated pain catastrophizing who are scheduled for knee arthroplasty: a quasi-experimental study. *Archives of physical medicine and rehabilitation*. 2011;92(6):859-65. doi:https://dx.doi.org/10.1016/j.apmr.2011.01.003	Not an RCT
**Rittharomya J**, Aree-ue S, Malathum P, Orathai P, Belza B, Kawinwonggowit V. The Effectiveness of Preoperative Quadriceps Exercise and Diet Control Program for Older Adults Waiting for Total Knee Arthroplasty: A Randomized Controlled Trial. *Pacific RIM International Journal of Nursing Research*. 2020;24(4):485-501	No eligible follow-up time point
**Rodgers JA,** Garvin KL, Walker CW, Morford D, Urban J, Bedard J. Preoperative physical therapy in primary total knee arthroplasty. *The Journal of arthroplasty*. 1998;13(4):414-21.	Not an RCT
**Ródenas-Martínez S**, Santos-Andrés JF, Abril-Boren C, Usabiaga-Bernal T, Abouh-Lais S, Aguilar-Naranjo JJ. Effectiveness of a pre-surgery rehabilitation program in total knee arthroplasty. *Rehabilitación.* 2008;42(1):4-12.	Different postoperative care
**Sandell, LC.** A multidisciplinary assessment and intervention for patients awaiting total hip replacement to improve their quality of life. *International Journal of Orthopaedic and Trauma Nursing.* 2008;12(1):26-34. doi:10.1016/j.joon.2007.11.002	THA patients only
**Saw MM**, Kruger-Jakins T, Edries N, Parker R. Significant improvements in pain after a six-week physiotherapist-led exercise and education intervention, in patients with osteoarthritis awaiting arthroplasty, in South Africa: a randomised controlled trial. *BMC musculoskeletal disorders*. 2016;17(100968565):236. doi:https://dx.doi.org/10.1186/s12891-016-1088-6	Combined TKA/THA analysis
**Sjoling M**, Nordahl, et al. The impact of preoperative information on state anxiety, postoperative pain and satisfaction with pain management. *Patient education and counseling*. 2003;51(2):169-76. doi: 10.1016/s0738-3991(02)00191-x	No eligible follow-up time point
**Skoffer B**, Maribo T, Mechlenburg I, Korsgaard CG, Soballe K, Dalgas U. Efficacy of preoperative progressive resistance training in patients undergoing total knee arthroplasty: 12-month follow-up data from a randomized controlled trial. *Clinical rehabilitation*. 2020;34(1):82-90.doi:https://dx.doi.org/10.1177/0269215519883420	No eligible follow-up time point
**Soeters R**, White PB, Murray-Weir M, Koltsov JCB, Alexiades MM, Ranawat AS. Preoperative Physical Therapy Education Reduces Time to Meet Functional Milestones After Total Joint Arthroplasty. *Clinical orthopaedics and related research.* 2018;476(1):40-48. doi:https://dx.doi.org/10.1007/s11999.0000000000000010	Combined TKA/THA analysis
**Soni A**, Mudge, et al. Severe knee osteoarthritis: a study of combined acupuncture and physiotherapy vs home exercise advice in patients awaiting total knee arthroplasty. 2010;49: i79?i80. doi:10.1093/rheumatology/keq722	Conference abstract
**Soni A**, Joshi A, Mudge N, Wyatt M, Williamson L. Supervised exercise plus acupuncture for moderate to severe knee osteoarthritis: a small randomised controlled trial. *Acupuncture in medicine*: journal of the British Medical Acupuncture Society. 2012;30(3):176-81. doi:https://dx.doi.org/10.1136/acupmed-2012-010128	Bilateral TKA patients
**Swank AM**, Kachelman JB, Bibeau W, et al. Prehabilitation before total knee arthroplasty increases strength and function in older adults with severe osteoarthritis. *Journal of strength and conditioning research*. 2011;25(2):318-25. doi:https://dx.doi.org/10.1519/JSC.0b013e318202e431	No eligible follow-up time point
**Temporiti F**, Ruspi A, De Leo D, et al. Action Observation and Motor Imagery administered the day before surgery enhance functional recovery in patients after total hip arthroplasty: A randomized controlled trial. *Clinical rehabilitation.* 2022;36(12):1613-1622. doi:https://dx.doi.org/10.1177/02692155221116820	THA patients only
**Terradas-Monllor M**, Ochandorena-Acha M, Beltran-Alacreu H, Garcia Oltra E, Collado Saenz F, Hernandez Hermoso J. A feasibility study of home-based preoperative multimodal physiotherapy for patients scheduled for a total knee arthroplasty who catastrophize about their pain. *Physiotherapy theory and practice*. 2023;39(8):1606-1625. doi:https://dx.doi.org/10.1080/09593985.2022.2044423	Ineligible patient population

Study	Exclusion Reason
**Tolk JJ**, Janssen RPA, Haanstra TM, Bierma-Zeinstra SMA, Reijman M. The EKSPECT study: the influence of Expectation modification in Knee arthroplasty on Satisfaction of PatiEnts: study protocol for a randomized Controlled Trial. *Trials.* 2018;19(1):437. doi:https://dx.doi.org/10.1186/s13063-018-2821-2	Clinical trial protocol
**Toume M**. Intervention and evaluation of preadmission patient education for patients underdoing [sic] total hip arthroplasty. *Journal of Japan Academy of Nursing Science*. 2004;24(2):24-32.	Ineligible language
**Turkoglu M**, Sayilan, AA. The effect of different training programs on patients' anxiety and pain levels before total hip arthroplasty surgery. *Pielegniarstwo Chirurgiczne I Angiologiczne–Surgical and Vascular Nursing*. 2021;(2):94-102.	Not an RCT
**Valtonen A**, Pöyhönen T, Manninen M, Heinonen A, Sipilä S. Effects of preoperative aquatic resistance training on knee pain, mobility limitation and muscle impairments in people with late-stage knee osteoarthritis. 2015;101:eS1568-eS1569. doi:10.1016/j.physio.2015.03.1570	Conference abstract
**Villadsen A**, Overgaard S, Holsgaard-Larsen A, Christensen R, Roos EM. Immediate efficacy of neuromuscular exercise in patients with severe osteoarthritis of the hip or knee: a secondary analysis from a randomized controlled trial. *J Rheumatol.* Jul 2014;41(7):1385-94. doi:10.3899/jrheum.130642	No eligible follow-up time point
**Villadsen A**, Roos, et al. Neuromuscular exercise improves functional performance in patients with severe hip osteoarthritis. 2011;19: S44.	Conference abstract
**Villadsen A.** Neuromuscular exercise prior to joint arthroplasty in patients with osteoarthritis of the hip or knee. *Danish medical journal.* 2016;63(4)	Not an RCT
**Vukomanovic A**, Popovic, et al. The effects of short-term preoperative physical therapy and education on early functional recovery of patients younger than 70 undergoing total hip arthroplasty. *Vojnosanitetski pregled.* 2008;65(4):291-7.	THA patients only
**Wang Q**, Ma, et al. Effects of preoperative otago exercise program on rehabilitation in total knee arthroplasty patients. *International Journal of Clinical and Experimental Medicine*. 2020;13(8):5914-5922.	No eligible follow-up time point
**Wang WA**, Gilbey JH, Ackland RT. Perioperative exercise programs improve early return of ambulatory function after total hip arthroplasty: a randomized, controlled trial. *American journal of physical medicine & rehabilitation*. 2002;81(11):801-6.	THA patients only
**Wijgman JA**, Dekkers, et al. No positive effect of preoperative physical therapy and instruction of patients to be subjected to hip arthroplasty. *Nederlands Tijdschrift voor Geneeskunde*. 1994;138(19):949-952.	THA patients only
**Williams D**. Preoperative patient education: in the home or in the hospital? *Orthopaedic Nursing.* 1986;5(1):37-41	Not an RCT
**Williams AD**, O'Brien J, Mulford J, et al. Effect of combined exercise training and behaviour change counselling versus usual care on physical activity in patients awaiting hip and knee arthroplasty: A randomised controlled trial. *Osteoarthr Cartil Open*. 2022;4(4):100308. doi:10.1016/j.ocarto.2022.100308	Combined TKA/THA analysis
**Williamson L**, Wyatt MR, Yein K, Melton JTK. Severe knee osteoarthritis: a randomized controlled trial of acupuncture, physiotherapy (supervised exercise) and standard management for patients awaiting knee replacement. *Rheumatology (Oxford, England)*. 2007;46(9):1445-9.	Bilateral TKA patients
**Wilson RA**. A Randomized Controlled Trial of an Individualized Preoperative Education Intervention for Symptom Management Following Total Knee Arthroplasty. University of Toronto (Canada); 2011.	No eligible follow-up time point
**Yu J**, Liu, X. Effect of psychological intervention care on the negative mood and hope level of elderly patients undergoing knee arthroplasty. *Am J Transl Res*. 2021;13(6):6584-6591	Different postoperative care
**Zeng** R, Lin, et al. A randomized controlled trial: preoperative home-based combined Tai Chi and Strength Training (TCST) to improve balance and aerobic capacity in patients with total hip arthroplasty (THA). *Archives of gerontology and geriatrics.* 2015;60(2):265-71. doi:https://dx.doi.org/10.1016/j.archger.2014.11.009	THA patients only
**Zheng** Y, Huang, et al. The Effect of Preoperative Rehabilitation Training on the Early Recovery of Joint Function after Artificial Total Knee Arthroplasty and Its Effect Evaluation. *Journal of healthcare engineering*. 2022;2022(101528166):3860991. doi:https://dx.doi.org/10.1155/2022/3860991	Ineligible intervention

S4. Assessment of the Transitivity Assumption (3 Months)

Age

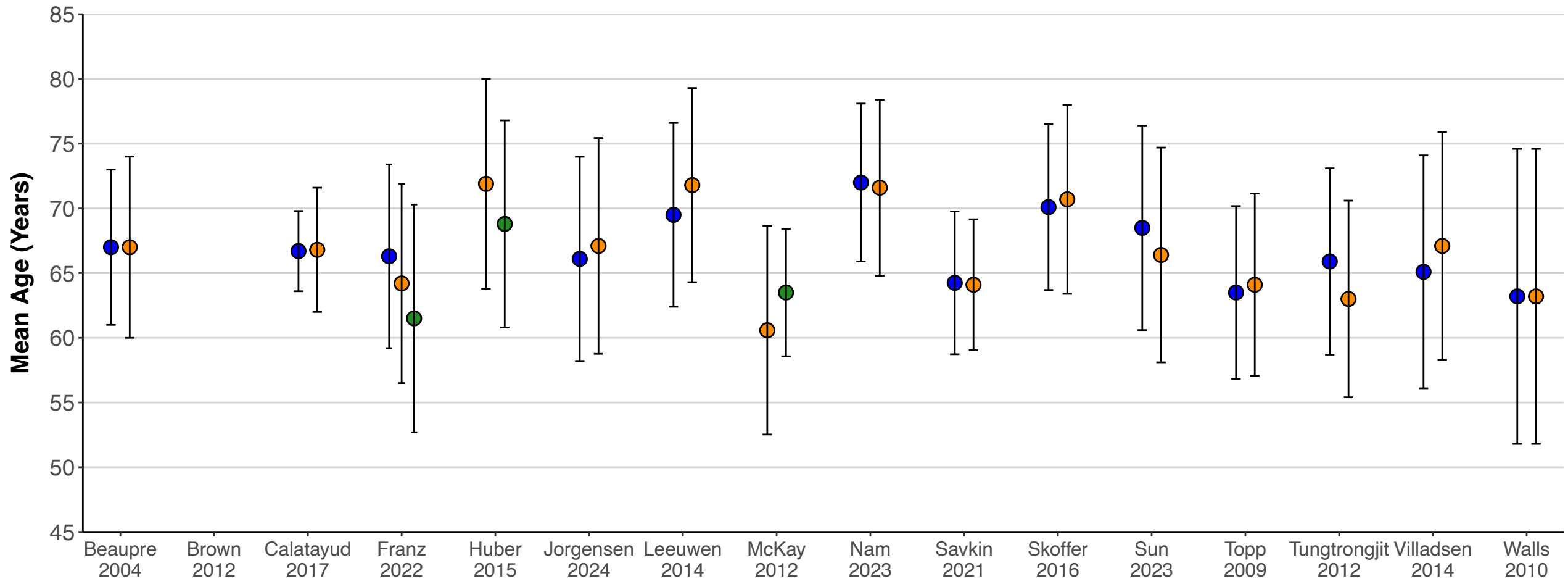

BMI

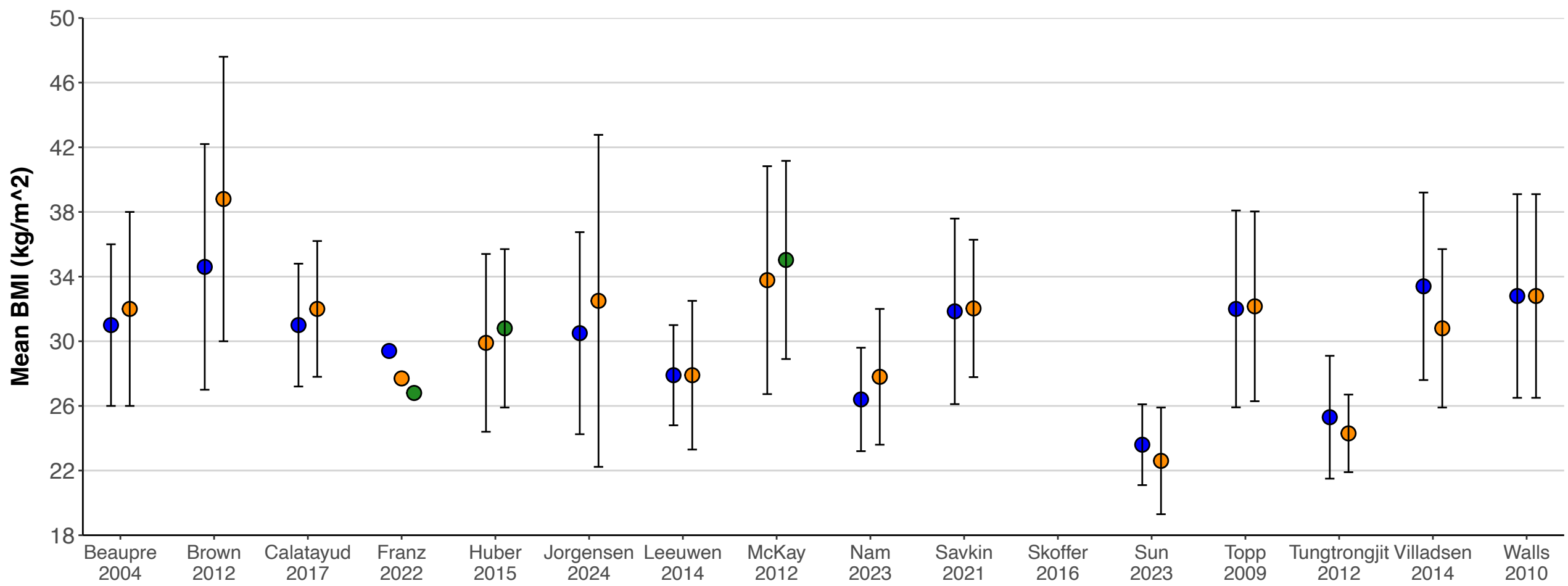

Proportion Females

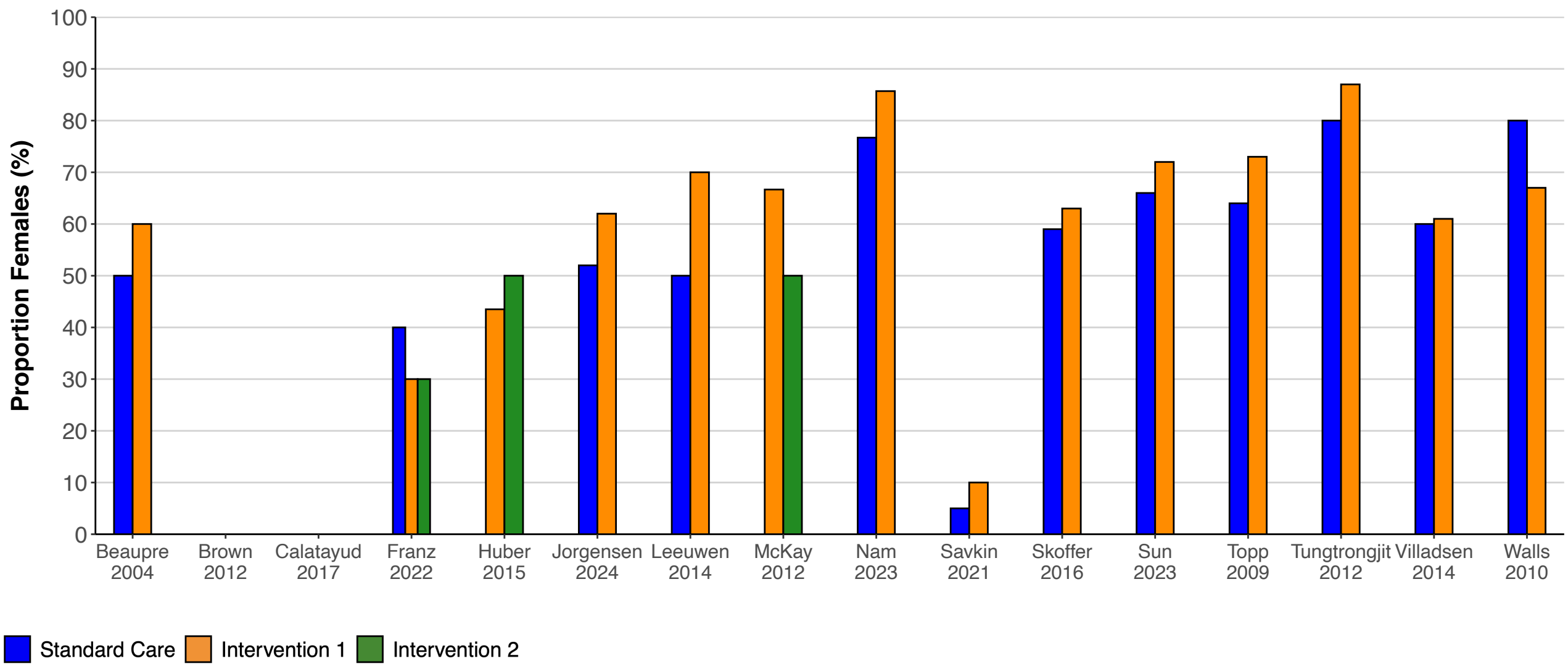

Baseline characteristics across studies included in the 3-month network meta-analysis. The distributions of mean age, body mass index (BMI), and the proportion of female participants are presented to evaluate the plausibility of the transitivity assumption.

S5. Study-Level Outcome Data and Effect Estimates (3 Months)

Author	Year	Treatment 1	Treatment 2	Sample size, N		Outcome measure	Range/ Unit	Baseline score, mean (SD)		Follow-up score, mean (SD)		Effect estimate	Follow-up	Risk of bias
				Treat 1	Treat 2			Treat 1	Treat 2	Treat 1	Treat 2	SMD (95% CI)	Time	(Overall)
**Physical Function**														
Franz	2022	Standard care	Aerobic exercise	10	10	KOOS ADL	0 - 100	49.2 (4.2)	46.7 (3.5)	58.7 (4.5)	59.0 (3.5)	0.07 (-0.81, 0.95)	3 Months	Some concerns
Franz	2022	Standard care	BFR exercise	10	10	KOOS ADL	0 - 100	49.2 (4.2)	45.5 (4.2)	58.7 (4.5)	63.7 (5.1)	1.13 (0.21, 2.05)	3 Months	Some concerns
Franz	2022	Aerobic exercise	BFR exercise	10	10	KOOS ADL	0 - 100	46.7 (3.5)	45.5 (4.2)	59.0 (3.5)	63.7 (5.1)	1.06 (0.14, 1.98)	3 Months	Some concerns
Jorgensen[1,4]	2024	Standard care	BFR exercise	34	31	KOOS ADL	0 - 100	53.0 (15.0)	54.3 (15.6)	75.7	77.2	0.01 (-0.48, 0.50)	3 Months	Some concerns
Nam[2]	2023	Standard care	Education	83	81	WOMAC Function	0 - 68	23.8 (7.2)	25.7 (7.5)	38.6 (8.6)	38.1 (7.2)	-0.06 (-0.37, 0.25)	3 Months	Some concerns
Savkin	2021	Standard care	NMES	20	20	KOOS ADL	0 - 100	42.4 (13.1)	50.5 (15.6)	87.2 (11.2)	89.9 (12.4)	0.22 (-0.41, 0.85)	12 Weeks	Some concerns
Walls[2]	2010	Standard care	NMES	5	9	WOMAC Function	0 - 68	31.0 (15.7)	31.1 (11.3)	46.4 (11.3)	48.1 (13.9)	0.13 (-0.97, 1.23)	12 Weeks	Some concerns
Beaupre	2004	Standard care	Exercise & Education	58	51	WOMAC Function	0 - 100	50.0 (17)	51.0 (18.0)	73.0 (15.0)	73.0 (17.0)	0.00 (-0.37, 0.37)	3 Months	Some concerns
Huber[4]	2015	Education	Exercise & Education	20	21	KOOS ADL	0 - 100	49.9 (19.1)	51.7 (17.8)	78.4 (20.6)	75.9 (13.3)	-0.15 (-0.76, 0.46)	3 Months	Some concerns
Calatayud[2]	2017	Standard care	LE-strength training	22	22	WOMAC Function	0 - 68	31.3 (2.4)	30.8 (2.3)	45.3 (2.3)	49.2 (2.1)	1.77 (1.06, 2.48)	3 Months	High risk
McKay[2]	2012	Upper body strength	LE-strength training	10	7	WOMAC Function	0 - 68	27.8 (5.0)	34.3 (11.8)	53.7 (15.4)	54.9 (11.6)	0.09 (-0.87, 1.05)	12 Weeks	Some concerns
Skoffer	2016	Standard care	LE-strength training	21	29	KOOS ADL	0 - 100	56.7 (14.8)	58.8 (13.9)	78.2 (12.9)	82.9 (11.7)	0.38 (-0.19, 0.95)	12 Weeks	Some concerns
Tungtrongjit[2,5]	2012	Standard care	LE-strength training	30	30	WOMAC Function	0 - 170	76.2 (24.9)	76.7 (22.7)	134.0 (19.7)	144.3 (19.1)	0.53 (0.02, 1.04)	3 Months	High risk
Brown[4]	2012	Standard care	Multidomain exercise	7	11	SF-36 Physical Function	0 - 100	NR	NR	39.3 (30.2)	66.4 (20.7)	1.10 (0.08, 2.12)	3 Months	High risk
Villadsen[1,4,5]	2014	Standard care	Multidomain exercise	40	41	KOOS ADL	0 - 100	43.9 (13.8)	53.3 (15.2)	65.6	80.5	0.33 (-0.10, 0.76)	3 Months	Some concerns
**Quadricpes Strength**														
Franz	2022	Standard care	Aerobic exercise	10	10	Knee extension	kg	20.3 (8.1)	11.8 (7.8)	15.0 (6.3)	11.1 (9.5)	-0.44 (-1.32, 0.44)	3 Months	Some concerns
Franz	2022	Standard care	BFR exercise	10	10	Knee extension	kg	20.3 (8.1)	13.8 (9.1)	15.0 (6.3)	22.1 (10.4)	0.80 (-0.10, 1.70)	3 Months	Some concerns
Franz	2022	Aerobic exercise	BFR exercise	10	10	Knee extension	kg	11.8 (7.8)	13.8 (9.1)	11.1 (9.5)	22.1 (10.4)	1.23 (0.31, 2.15)	3 Months	Some concerns
Jorgensen[1,4]	2024	Standard care	BFR exercise	34	31	Isometric knee extension	Nm/kg	2.6 (0.8)	2.3 (1.0)	2.2	2.4	0.61 (0.12, 1.10)	3 Months	Low risk
Savkin	2021	Standard care	NMES	20	20	Knee extension	Newton	94.9 (22.0)	99.0 (19.6)	98.0 (22.8)	96.3 (25.8)	-0.07 (-0.70, 0.56)	12 Weeks	Some concerns
Walls	2010	Standard care	NMES	5	9	Isometric knee extension	Nm	89.5 (12.0)	87.5 (12.0)	61.7 (12.0)	80.9 (12.0)	1.60 (0.37, 2.83)	12 Weeks	Some concerns
Beaupre	2004	Standard care	Exercise & Education	58	51	Isometric leg extension	lbs	24.0 (11.0)	22.0 (8.0)	27.0 (8.0)	27.0 (10.0)	0.00 (-0.37, 0.37)	3 Months	Some concerns
Huber[1,4]	2015	Education	Exercise & Education	20	21	Isometric knee extension	Newton	247.6 (100.4)	244.8 (91.7)	204.1	197.7	0.04 (-0.57, 0.65)	3 Months	Low risk
Calatayud	2017	Standard care	LE-strength training	22	22	Isometric knee extension	kg	23.5 (7.8)	23.5 (7.5)	14.3 (7.2)	22.8 (7.0)	1.20 (0.55, 1.85)	3 Months	High risk
Leeuwen[5]	2014	Standard care	LE-strength training	7	9	Isometric knee extension	Nm	121.0 (52.0)	106.0 (45.0)	97.0 (40.0)	76.0 (34.0)	-0.57 (-1.57, 0.43)	12 Weeks	High risk
McKay[5]	2012	Upper body strength	LE-strength training	10	7	Isometric knee extension	Nm/kg	0.8 (0.5)	1.0 (0.6)	0.7 (0.4)	0.8 (0.6)	0.07 (-0.89, 1.03)	12 Weeks	Some concerns
Skoffer	2016	Standard care	LE-strength training	21	29	Isometric knee extension	Nm/kg	1.0 (0.4)	1.0 (0.3)	0.8 (0.3)	1.0 (0.3)	0.67 (0.10, 1.24)	12 Weeks	Some concerns
Tungtrongjit	2012	Standard care	LE-strength training	30	30	Knee extension	NR	5.8 (3.9)	6.5 (3.8)	5.3 (3.4)	7.5 (2.9)	0.70 (0.17, 1.23)	3 Months	High risk
Sun	2023	Standard care	Multidomain exercise	41	40	Isometric knee extension	kg	21.7 (5.8)	19.9 (5.1)	16.3 (6.3)	21.9 (9.1)	0.72 (0.27, 1.17)	3 Months	High risk
Topp	2009	Standard care	Multidomain exercise	28	26	Quadriceps strength	PT/BW (kg)	60.2 (6.3)	53.8 (6.6)	60.7 (4.8)	62.3 (5.0)	0.31 (-0.22, 0.84)	3 Months	Some concerns
Villadsen[1,3,4]	2014	Standard care	Multidomain exercise	40	41	Knee extension	Watt	36.4	32.6	24.3	21.7	-0.03 (-0.46, 0.40)	3 Months	Some concerns

Author	Year	Treatment 1	Treatment 2	Sample size, N		Outcome measure	Range/	Baseline score, mean (SD)		Follow-up score, mean (SD)		Effect estimate	Follow-up	Risk of bias
				Treat 1	Treat 2		Unit	Treat 1	Treat 2	Treat 1	Treat 2	SMD (95% CI)	Time	(Overall)
**Pain**														
Franz	2022	Standard care	Aerobic exercise	10	10	KOOS Pain	0 - 100	48.7 (3.9)	44.2 (3.8)	60.8 (6.9)	61.7 (6.8)	0.15 (-0.73, 1.03)	3 Months	Some concerns
Franz	2022	Standard care	BFR exercise	10	10	KOOS Pain	0 - 100	48.7 (3.9)	41.1 (4.3)	60.8 (6.9)	67.8 (3.5)	1.18 (0.26, 2.10)	3 Months	Some concerns
Franz	2022	Aerobic exercise	BFR exercise	10	10	KOOS Pain	0 - 100	44.2 (3.8)	41.1 (4.3)	61.7 (6.8)	67.8 (3.5)	1.03 (0.11, 1.95)	3 Months	Some concerns
Jorgensen[1,4]	2024	Standard care	BFR exercise	34	31	KOOS Pain	0 - 100	45.0 (31.1)	50.5 (15.4)	74.4	73.6	-0.34 (-0.83, 0.15)	3 Months	Some concerns
Nam[2]	2023	Standard care	Education	83	81	WOMAC Pain	0 - 20	8.3 (3.2)	8.1 (3.1)	14.3 (2.1)	13.7 (2.0)	-0.29 (-0.60, 0.02)	3 Months	Some concerns
Savkin	2021	Standard care	NMES	20	20	KOOS Pain	0 - 100	39.6 (12.1)	43.3 (17.9)	86.4 (13.0)	83.9 (15.2)	-0.18 (-0.81, 0.45)	12 Weeks	Some concerns
Walls[2]	2010	Standard care	NMES	5	9	WOMAC Pain	0 - 20	10.0 (5.7)	8.3 (2.7)	14.8 (4.3)	14.6 (4.0)	-0.05 (-1.15, 1.05)	12 Weeks	Some concerns
Beaupre	2004	Standard care	Exercise & Education	58	51	WOMAC Pain	0 - 100	50.0 (19.0)	50.0 (16.0)	73.0 (14.0)	74.0 (18.0)	0.06 (-0.31, 0.43)	3 Months	Some concerns
Huber[4]	2015	Education	Exercise & Education	20	21	KOOS Pain	0 - 100	47.3 (16.8)	48.1 (17.6)	74.4 (17.4)	71.9 (16.0)	-0.15 (-0.76, 0.46)	3 Months	Some concerns
Calatayud[2]	2017	Standard care	LE-strength training	22	22	WOMAC Pain	0 - 20	9.4 (1.0)	9.5 (1.0)	16.2 (0.9)	17.1 (0.9)	1.00 (0.37, 1.63)	3 Months	High risk
McKay[2]	2012	Upper body strength	LE-strength training	10	7	WOMAC Pain	0 - 20	8.1 (3.6)	9.2 (2.2)	16.42 (4.4)	15.6 (3.2)	-0.21 (-1.17, 0.75)	12 Weeks	Some concerns
Skoffer	2016	Standard care	LE-strength training	21	29	KOOS Pain	0 - 100	53.4 (13.5)	53.0 (13.3)	79.9 (14.2)	78.1 (16.3)	-0.12 (-0.69, 0.45)	12 Weeks	Some concerns
Tungtrongjit[2,5]	2012	Standard care	LE-strength training	30	30	WOMAC Pain	0 - 50	22.1 (8.1)	19.7 (6.3)	39.4 (6.4)	44.6 (4.4)	0.95 (0.42, 1.48)	3 Months	High risk
Brown[4,5]	2012	Standard care	Multidomain exercise	7	11	SF-36 Pain	0 - 100	NR	NR	47.6 (22.8)	59.9 (19.2)	0.60 (-0.36, 1.56)	3 Months	High risk
Villadsen[1,4]	2014	Standard care	Multidomain exercise	40	41	KOOS Pain	0 - 100	39.7 (12.6)	47.8 (14.9)	59.1	72.7	0.29 (-0.14, 0.72)	3 Months	Some concerns
**Health-Related Quality of Life**														
Franz	2022	Standard care	Aerobic exercise	10	10	KOOS QoL	0 - 100	26.0 (9.2)	24.5 (5.3)	45.6 (9.2)	44.4 (4.6)	-0.17 (-1.05, 0.71)	3 Months	Some concerns
Franz	2022	Standard care	BFR exercise	10	10	KOOS QoL	0 - 100	26.0 (9.2)	21.9 (3.3)	45.6 (9.2)	52.5 (6.8)	0.97 (0.07, 1.87)	3 Months	Some concerns
Franz	2022	Aerobic exercise	BFR exercise	10	10	KOOS QoL	0 - 100	24.5 (5.3)	21.9 (3.3)	44.4 (4.6)	52.5 (6.8)	1.14 (0.22, 2.06)	3 Months	Some concerns
Jorgensen[1,4]	2024	Standard care	BFR exercise	34	31	KOOS QoL	0 - 100	28.3 (12.7)	31.0 (13.2)	57.2	64.8	0.22 (-0.27, 0.71)	3 Months	Some concerns
Nam	2023	Standard care	Education	83	81	SF-36 PCS	0 - 100	20.9 (11.8)	21.8 (11.7)	50.2 (14.6)	50.8 (10.7)	0.05 (-0.26, 0.36)	3 Months	Some concerns
Savkin	2021	Standard care	NMES	20	20	KOOS QoL	0 - 100	23.1 (19.7)	25.6 (20.6)	68.4 (26.4)	73.8 (23.6)	0.21 (-0.42, 0.84)	12 Weeks	Some concerns
Walls	2010	Standard care	NMES	5	9	SF-36 PCS	0 - 100	39.0 (20.8)	41.0 (22.1)	57.0 (25.2)	57.1 (18.0)	0.00 (-1.10, 1.10)	12 Weeks	Some concerns
Beaupre	2004	Standard care	Exercise & Education	58	51	SF-36 PCS	0 - 100	29.0 (6.0)	29.0 (6.0)	35.0 (8.0)	35.0 (9.0)	0.00 (-0.37, 0.37)	3 Months	Some concerns
Huber[1,4]	2015	Education	Exercise & Education	20	21	KOOS QoL	0 - 100	26.8 (15.3)	26.4 (14.5)	65.3	59.0	0.28 (-0.33, 0.89)	3 Months	Some concerns
McKay	2012	Upper body strength	LE-strength training	10	7	SF-36 PCS	0 - 100	24.2 (4.5)	26.9 (7.0)	34.8 (9.8)	41.3 (10.1)	0.65 (-0.35, 1.65)	12 Weeks	Some concerns
Skoffer	2016	Standard care	LE-strength training	21	29	KOOS QoL	0 - 100	33.8 (14.4)	39.6 (14.8)	61.9 (16.6)	66.2 (18.9)	0.24 (-0.33, 0.81)	12 Weeks	Some concerns
Villadsen[1,4]	2014	Standard care	Multidomain exercise	40	41	KOOS QoL	0 - 100	27.1 (16.8)	30.9 (13.1)	48.5	57.0	0.24 (-0.19, 0.67)	3 Months	Some concerns

Study-level baseline and follow-up outcome data for the 3-month network meta-analysis. Baseline and follow-up values are presented as mean (SD), with SMD and 95% CIs for each direct treatment comparison. Positive SMD values favor Treatment 2. Higher scores indicate better outcomes for all measures. [1] SMDs were calculated from change scores (baseline to 12–14 weeks). [2] Scores were reversed for instruments in which higher scores indicated worse outcomes. [3] Three-month mean values were approximated from the baseline median and reported change score. [4] Primary endpoint of the study. [5] Primary outcome of the study.

***Abbreviations*:** ADL, activities of daily living; BFR, blood flow restriction; CIs, confidence intervals; HR-QoL, health-related quality of life; KOOS, Knee Injury and Osteoarthritis Outcome Score; LE, lower extremity; N, number of participants; NMES, neuromuscular electrical stimulation; NR, not reported; PCS, Physical Component Summary; PT/BW, peak torque per kilogram body weight; SD,standard deviation; SF-36, 36-Item Short Form Health Survey; SMD, standardized mean difference; WOMAC, Western Ontario and McMaster Universities Osteoarthritis Index.

S6. Study-Level Descriptions of Prehabilitation Interventions

STUDY	MODALITY	INTERVENTION DESCRIPTION
Franz et al. (2022)	Aerobic exercise	6-week cycling ergometer training protocol, performed twice weekly for approximately 50 minutes at individualised intensity, using sham blood flow restriction (BFR) cuffs inflated to 20 mmHg and served as the active-control group.
Franz et al. (2022)	BFR exercise	6-week cycling ergometer training protocol with blood flow restriction (BFR), performed twice weekly for approximately 50 minutes at individualised intensity. BFR was applied bilaterally using inflatable tourniquets set at 40% of each participant's limb occlusion pressure during the cycling sessions, administered three times per leg per session, with occlusion intervals increasing from 1 minute in week 1 to 6 minutes in week 6.
Jorgensen et al. (2024)	BFR exercise	8-week supervised resistance training program with three weekly sessions using blood flow restriction (60% limb occlusion pressure) on the affected leg. Only the affected leg was trained. Sessions included a 10-minute cycling warm-up, followed by leg press and knee extension (4 sets: 30, 15, 15, to failure at 30% 1RM). The load increased if >15 repetitions were completed in the final set. Cuff pressure was maintained during 30-second rest intervals between sets, with a 5-minute rest without BFR between exercises.
Huber et al. (2015)	Education	4-week knee school education program was delivered in three weekly individual or group sessions by a specially trained physiotherapist. The program covered knee anatomy, prosthesis use, postoperative pain management, and rehabilitation. Educational materials included anatomical models, handouts, visual media (photos, videos), and peer discussions.
Nam et al. (2023)	Education	A one-on-one education module focusing on realistic expectations following TKA. It addressed expected improvements (e.g., pain, function, psychological well-being), individual factors (e.g., age, comorbidities), and limitations (e.g., high-impact sports, kneeling, squatting). Cultural adaptations included guidance on avoiding culturally specific postures (e.g., kneeling on ondol-floor living), and information on crepitus, thigh/calf pain, and contractures was emphasised.
Rooks et al. (2006)	Education	6-week education program with two mailed booklets on home modification, fall prevention, and surgery preparation, plus three follow-up support calls.
Beaupre et al. (2004)	Exercise & education	4-week program with three sessions per week combining education and exercise. Education addressed crutch walking, bed mobility, transfers, and postoperative range of motion (ROM). Exercises focused on knee mobility and simple strengthening exercises with progressive resistance added to patient tolerance.
Huber et al. (2015)	Exercise & education	4–12-week program combining weekly education (knee school) and supervised neuromuscular training (1–3 sessions/week). Knee school (3 sessions) covered joint anatomy, prosthesis use, pain management, and postoperative rehabilitation. Neuromuscular training (NEMEX-TJR) included aerobic warm-up, circuit-based exercises (2–3 cycles of 10–15 reps), and cooldown, targeting postural stability, alignment, strength, and function, with individualised progression based on performance and tolerance.
Blasco et al. (2020)	Home-based multidomain	4-week unsupervised home-based lower-extremity strengthening and balance exercise program.
Calatayud et al. (2017)	Lower-extremity strength	8-week supervised high-intensity lower-extremity strengthening program (3 sessions/week), including resistance training, balance exercises, and stretching.
Dominguez-Navarro et al. (2021)	Lower-extremity strength	4-week supervised progressive lower-extremity strengthening program (12 sessions).
Van Leeuwen et al. (2014)	Lower-extremity strength	6-week progressive strength program targeting both lower limbs, performed 2–3 times per week plus a 2–3 times weekly home program (step-ups, squats). Exercises included single-leg leg press, step-ups, squats, and knee extensions. Load was adjusted per set using a ±3% laod adjustment rule, with repetitions decreasing and weight increasing over time. Modifications for pain were made while maintaining high intensity.
McKay et al. (2012)	Lower-extremity strength	6-week supervised strength training program with three 30-minute sessions per week. Each session included a 10-minute aerobic warm-up (participant's choice of equipment), followed by two sets of 8 reps of bilateral lower-body exercises (calf raise, leg press, leg curl, knee extension). Exercises started at 60% of 1RM and progressed by 1–2 kg weekly
Skoffer et al. (2016)	Lower-extremity strength	4-week supervised progressive resistance training (PRT) program performed three times per week. Each 60-minute session included a 10-minute cycling warm-up, followed by three sets of six unilateral lower-limb exercises (leg press, knee extension/flexion, hip extension/abduction/adduction) at 12RM progressing to 8RM, with 2-minute rest intervals. Sessions concluded with stretching of key lower-limb muscle groups.
Tungtrongjit et al. (2012)	Lower-extremity strength	3-week home-based quadriceps program was performed three times daily. Each session included 10 seated knee extensions, held for≥6 seconds, with 20-second rests. Patients received weekly phone guidance on exercise technique.
Blasco et al. (2020)	Multidomain exercise	4-week supervised lower-extremity strengthening and balance training, including gait, agility, proprioception, and stability exercises.
Brown et al. (2012)	Multidomain exercise	8-week program performed three times weekly (one supervised, two home-based), combining warm-up, progressive TheraBand® resistance, flexibility, step training, and cool-down. Intensity and volume increased over time, supported by an exercise booklet, log, and individualised adjustments.
Dominguez-Navarro et al. (2021)	Multidomain exercise	4-week supervised multidomain exercise program (12 sessions), combining progressive lower-extremity strengthening, balance, and proprioceptive training.
Gstoettner et al. (2011)	Multidomain exercise	6-week supervised proprioceptive training program, including weekly supervised sessions and daily home balance exercises.
Rooks et al. (2006)	Multidomain exercise	6-week supervised water- and land-based exercise program (3 sessions/week), progressing from joint-specific aquatic exercises to cardiovascular, strength, and flexibility training.

STUDY	MODALITY	INTERVENTION DESCRIPTION
Sun et al. (2023)	Multidomain exercise	4-week home-based program with five 1-hour sessions per week, combining strength and balance training targeting peri-knee muscles. Exercises (5×10 reps at 10RM, with 60s rest) progressed from 0 to 2–3 kg. Sessions included warm-up, proprioceptive training, manual therapy, and ice compression, preceded by a supervised familiarisation session and guided by an online module.
Topp et al. (2009)	Multidomain exercise	Program duration varied depending on the time until surgery. Prehabilitation was performed at least 3 times weekly (1 supervised, 2 home-based), with each session including a 5-minute warm-up, nine TheraBand resistance exercises (e.g., squats, hip/knee flexion and extension, ankle plantar/dorsiflexion), step training on an 8-inch step, and a cool-down with light static stretching.
Villadsen et al. (2014)	Multidomain exercise	8-week neuromuscular exercise (NEMEX-TJR) program delivered twice weekly in 1-hour group sessions, including a 10-minute cycling warm-up and a circuit targeting core stability/postural control, postural orientation, lower extremity muscle strength and functional exercises. Progression was based on sensorimotor control and movement quality, monitored by a physiotherapist.
Savkin et al. (2021)	NMES	6-week NMES intervention applied high-intensity, visible quadriceps contractions using a portable stimulator for 20 minutes per session, 5 days per week. Electrodes were placed proximally and distally on the rectus femoris and vastus medialis. Stimulation was delivered in a seated position to elicit isometric contractions using biphasic, symmetrical square waveforms (50Hz, 400 µs pulse duration; 2s ramp-up, 10s contraction, 2s ramp-down, 30s rest).
Walls et al. (2010)	NMES	8-week home-based NMES program targeting the quadriceps of the affected leg, using a portable stimulator for 20 minutes per session. Stimulation was applied in a seated position with the knee flexed to 60°, starting on alternate days for 2 weeks, followed by 5 sessions per week for 6 weeks. Parameters included biphasic square waveform (50 Hz, pulse width 100–400 µs), 5s contraction, 10s rest, with ramp-up/down times of 1s and 0.5s. Stimulation intensity was set to the participant's maximum tolerated level.
McKay et al. (2012)	Upper body strength	6-week supervised upper-body control program, conducted three times per week for 30 minutes, included a 10-minute warm-up followed by two sets of 8 repetitions of exercises (lat pull, chest press, elbow flexion, and extension) starting at 60% of one-rep max, with gradual weight increments.

Abbreviations: BFR, blood flow restriction; NMES, neuromuscular electrical stimulation.

S7. Standard Preoperative and Postoperative Care Across Individual Studies

STUDY	STANDARD CARE	INTERVENTION 1	INTERVENTION 2	PREOPERATIVE STANDARD CARE	POSTOPERATIVE STANDARD CARE
Beaupre et al. (2004)	Standard Care	Exercise & Education	-	Continue normal activities.	Standard postoperative mobilization routines.
Blasco et al. (2020)	Standard Care	Multidomain exercise	Home-based multidomain	Continue normal activities.	In-hospital rehabilitation (mean stay 3.6 days), followed by 12 supervised outpatient sessions (45–60 min, 3x/week) during postoperative weeks 2–6.
Brown et al. (2012)	Standard Care	Multidomain exercise	-	Standard care.	Not reported.
Calatayud et al. (2017)	Standard Care	Lower extremity strength	-	Not reported.	Daily physiotherapy (60 min, Mon-Fr) for one month.
Dominguez-Navarro et al. (2021)	Standard Care	Lower extremity strength	Multidomain exercise	Standard care.	Postoperative rehabilitation included 12 outpatient physiotherapy sessions starting 10–12 days after surgery.
Franz et al. (2022)	Standard Care	Aerobic exercise	BFR exercise	Standard care.	Daily in-hospital physiotherapy (7 days), followed by 3-week inpatient rehabilitation.
Gstoettner et al. (2011)	Standard Care	Multidomain exercise	-	Standard care.	Not reported.
Huber et al. (2015)	◇	Education	Exercise & Education	◇	In-hospital physiotherapy until discharge (7–10 days), followed by outpatient (9–18 sessions) or inpatient rehabilitation (2–3 weeks, 10–15 treatment days).
Jorgensen et al. (2024)	Standard Care	BFR exercise	-	Education session and continue normal activities.	Home-based rehabilitation, with supervised rehabilitation if needed (CON: 70%; INT: 55%).
Van Leeuwen et al. (2014)	Standard Care	Lower extremity strength	-	Standard care, including education and exercises.	Strength training provided at various physiotherapy centers.
McKay et al. (2012)	◇	Upper body strength	Lower extremity strength	◇	Physiotherapy.
Nam et al. (2023)	Standard Care	Education	-	Standard care, including education.	Not reported.
Rooks et al. (2006)	◇	Education	Multidomain exercise	◇	Not reported.
Savkin et al. (2021)	Standard Care	NMES	-	Standard care.	Physiotherapy during hospitalization, followed by a 12-week home exercise program.
Skoffer et al. (2016)	Standard Care	Lower extremity strength	-	Continue normal activities.	Postoperative strength training, 3x/week for 4 weeks.
Sun et al. (2023)	Standard Care	Multidomain exercise	-	Standard care, including education.	Active and passive motion exercises to improve leg strength and knee mobility.
Topp et al. (2009)	Standard Care	Multidomain exercise	-	Continue normal activities.	Physiotherapy targeting functional tasks, strength, and flexibility, with up to nine home sessions until 0–100° knee ROM was achieved.
Tungtrongjit et al. (2012)	Standard Care	Lower extremity strength	-	Continue normal activities.	Physiotherapy focusing on functional tasks, strength and flexibility exercises.
Villadsen et al. (2014)	Standard Care	Multidomain exercise	-	3-hour education session with an exercise leaflet.	Rehabilitation provided within the patient's municipality.
Walls et al. (2010)	Standard Care	NMES	-	Standard care, including home exercises.	Physiotherapy (twice daily) from day three until discharge.

◇ No Standard care group in study; - not applicable; BFR, blood flow restriction; NMES, neuromuscular electrical stimulation; NR,Not reported.

S8A. Forest Plots of Study-Level Pairwise Comparisons (3 Months)

Physical Function

Study	N Prehab.	N Control	SMD	95% CI	SE
**Aerobic exercise vs BFR exercise**					
Franz 2022	10	10	−1.06	[−1.98; −0.15]	0.47
**Aerobic exercise vs Standard care**					
Franz 2022	10	10	0.07	[−0.81; 0.94]	0.45
**BFR exercise vs Standard care**					
Franz 2022	10	10	1.13	[0.21; 2.05]	0.47
Jorgensen 2024	31	34	0.01	[−0.47; 0.50]	0.25
**Random effects model**	**41**	**44**	**0.50**	**[−0.59; 1.59]**	
Heterogeneity: $I^2 = 77.4\%$, $\tau^2 = 0.4855$, $p = 0.0353$					
**Education vs Exercise & Education**					
Huber 2015	20	21	0.15	[−0.47; 0.76]	0.31
**Education vs Standard care**					
Nam 2023	81	83	−0.06	[−0.37; 0.24]	0.16
**Exercise & Education vs Standard care**					
Beaupre 2004	51	58	−0.00	[−0.38; 0.38]	0.19
**Lower−extremity strength vs Standard care**					
Tungtrongjit 2012	30	30	0.53	[0.02; 1.05]	0.26
Skoffer 2016	29	21	0.38	[−0.18; 0.95]	0.29
**Random effects model**	**59**	**51**	**0.46**	**[0.08; 0.85]**	
Heterogeneity: $I^2 = 0\%$, $\tau^2 = 0$, $p = 0.7085$					
**Lower−extremity strength vs Upper−body strength**					
McKay 2012	7	10	0.09	[−0.88; 1.05]	0.49
**Multidomain exercise vs Standard care**					
Villadsen 2014	41	40	0.33	[−0.11; 0.77]	0.22
Brown 2012	11	7	1.10	[0.08; 2.11]	0.52
**Random effects model**	**52**	**47**	**0.57**	**[−0.13; 1.27]**	
Heterogeneity: $I^2 = 46.2\%$, $\tau^2 = 0.1361$, $p = 0.1729$					
**NMES vs Standard care**					
Walls 2010	9	5	0.13	[−0.96; 1.22]	0.56
Savkin 2021	20	20	0.22	[−0.40; 0.85]	0.32
**Random effects model**	**29**	**25**	**0.20**	**[−0.34; 0.74]**	
Heterogeneity: $I^2 = 0\%$, $\tau^2 = 0$, $p = 0.8834$					

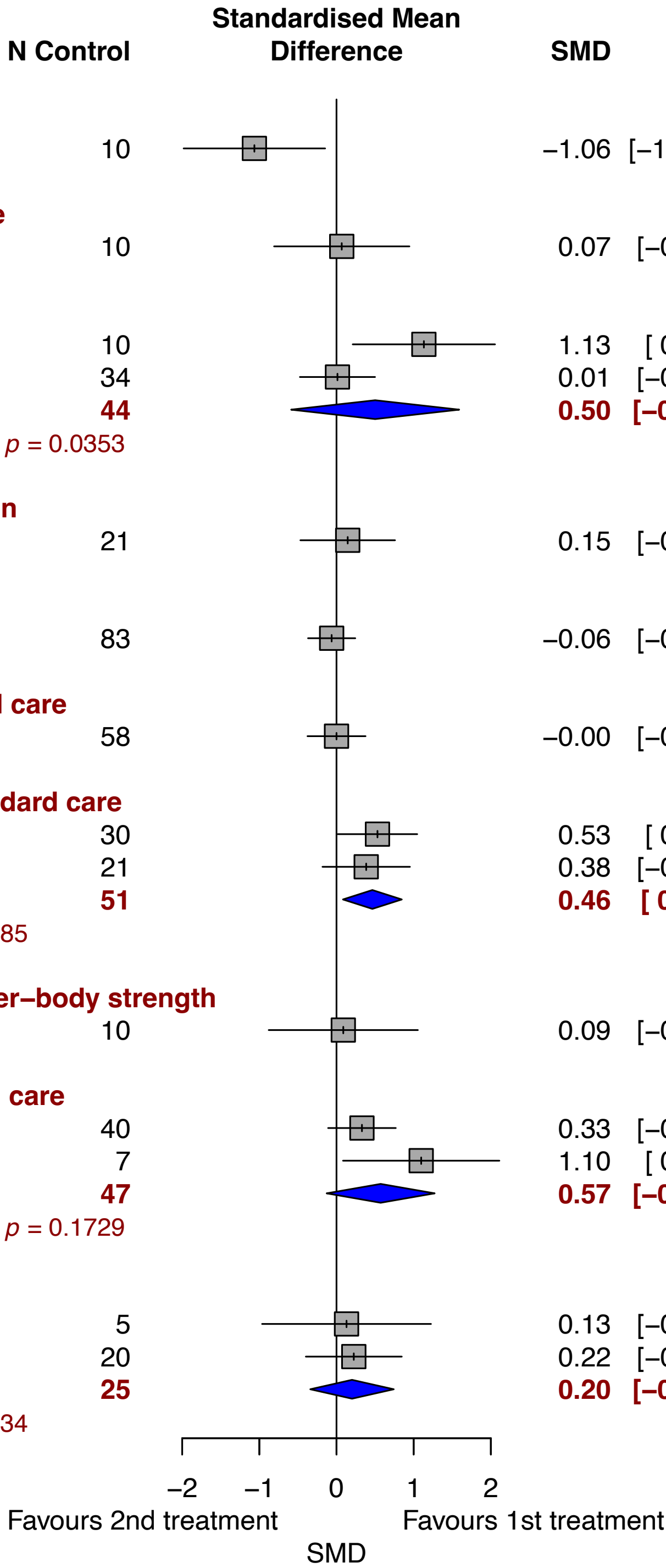

Study-level pairwise treatment comparisons for physical function at 3 months after TKA. Results are presented as standardized mean differences (SMD) with 95% confidence intervals (CI). Positive SMDs favor the first treatment listed in each comparison; negative SMDs favor the second treatment. BFR, blood flow restriction; NMES, neuromuscular electrical stimulation.

S8B. Forest Plots of Study-Level Pairwise Comparisons (3 Months)

Quadriceps Strength

Study	N Prehab.	N Control	SMD	95% CI	SE
**Aerobic exercise vs BFR exercise**					
Franz 2022	10	10	−1.23	[−2.17; −0.30]	0.47
**Aerobic exercise vs Standard care**					
Franz 2022	10	10	−0.44	[−1.32; 0.45]	0.45
**BFR exercise vs Standard care**					
Franz 2022	10	10	0.80	[−0.10; 1.70]	0.46
Jorgensen 2024	31	34	0.61	[0.11; 1.11]	0.25
**Random effects model**	**41**	**44**	**0.65**	**[0.22; 1.09]**	
Heterogeneity: $I^2 = 0\%$, $\tau^2 = 0$, $p = 0.7192$					
**Education vs Exercise & Education**					
Huber 2015	20	21	−0.04	[−0.66; 0.57]	0.31
**Exercise & Education vs Standard care**					
Beaupre 2004	51	58	−0.00	[−0.38; 0.38]	0.19
**Lower−extremity strength vs Standard care**					
Leeuwen 2014	9	7	−0.57	[−1.58; 0.44]	0.51
Skoffer 2016	29	21	0.67	[0.09; 1.24]	0.29
Tungtrongjit 2012	30	30	0.70	[0.18; 1.22]	0.27
**Random effects model**	**68**	**58**	**0.40**	**[−0.22; 1.02]**	
Heterogeneity: $I^2 = 61.6\%$, $\tau^2 = 0.1812$, $p = 0.0741$					
**Lower−extremity strength vs Upper−body strength**					
McKay 2012	7	10	0.07	[−0.90; 1.03]	0.49
**Multidomain exercise vs Standard care**					
Sun 2023	40	41	0.72	[0.27; 1.17]	0.23
Topp 2009	26	28	0.31	[−0.22; 0.85]	0.27
Villadsen 2014	41	40	−0.03	[−0.47; 0.40]	0.22
**Random effects model**	**107**	**109**	**0.33**	**[−0.12; 0.78]**	
Heterogeneity: $I^2 = 63.7\%$, $\tau^2 = 0.1016$, $p = 0.0636$					
**NMES vs Standard care**					
Savkin 2021	20	20	−0.07	[−0.69; 0.55]	0.32
Walls 2010	9	5	1.60	[0.36; 2.84]	0.63
**Random effects model**	**29**	**25**	**0.68**	**[−0.95; 2.30]**	
Heterogeneity: $I^2 = 82\%$, $\tau^2 = 1.1409$, $p = 0.0186$					

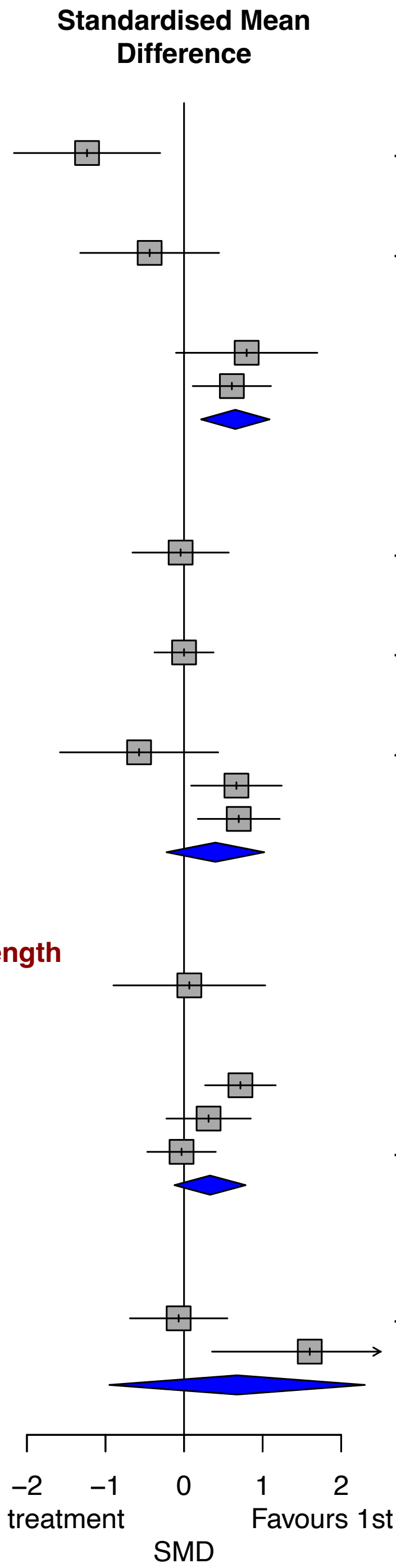

Study-level pairwise treatment comparisons for quadriceps strength outcomes at 3 months after TKA. Results are presented as standardized mean differences (SMD) with 95% confidence intervals (CI). Positive SMDs favor the first treatment listed in each comparison; negative SMDs favor the second treatment. BFR, blood flow restriction; NMES, neuromuscular electrical stimulation.

S8C. Forest Plots of Study-Level Pairwise Comparisons (3 Months)

Pain

Study	N Prehab.	N Control	SMD	95% CI	SE
**Aerobic exercise vs BFR exercise**					
Franz 2022	10	10	−1.03	[−1.94; −0.11]	0.47
**Aerobic exercise vs Standard care**					
Franz 2022	10	10	0.15	[−0.73; 1.03]	0.45
**BFR exercise vs Standard care**					
Franz 2022	10	10	1.18	[0.25; 2.10]	0.47
Jorgensen 2024	31	34	−0.34	[−0.83; 0.15]	0.25
**Random effects model**	**41**	**44**	**0.37**	**[−1.11; 1.85]**	
Heterogeneity: I^2 = 87.5%, τ^2 = 1.0033, p = 0.0046					
**Education vs Exercise & Education**					
Huber 2015	20	21	0.15	[−0.46; 0.76]	0.31
**Education vs Standard care**					
Nam 2023	81	83	−0.29	[−0.60; 0.02]	0.16
**Exercise & Education vs Standard care**					
Beaupre 2004	51	58	0.06	[−0.31; 0.44]	0.19
**Lower−extremity strength vs Standard care**					
Tungtrongjit 2012	30	30	0.95	[0.41; 1.48]	0.27
Skoffer 2016	29	21	−0.12	[−0.68; 0.45]	0.29
**Random effects model**	**59**	**51**	**0.42**	**[−0.62; 1.46]**	
Heterogeneity: I^2 = 86.2%, τ^2 = 0.4871, p = 0.0072					
**Lower−extremity strength vs Upper−body strength**					
McKay 2012	7	10	−0.21	[−1.18; 0.76]	0.49
**Multidomain exercise vs Standard care**					
Villadsen 2014	41	40	0.29	[−0.15; 0.72]	0.22
Brown 2012	11	7	0.60	[−0.37; 1.56]	0.49
**Random effects model**	**52**	**47**	**0.34**	**[−0.06; 0.74]**	
Heterogeneity: I^2 = 0%, τ^2 = 0, p = 0.5657					
**NMES vs Standard care**					
Walls 2010	9	5	−0.05	[−1.14; 1.04]	0.56
Savkin 2021	20	20	−0.18	[−0.80; 0.44]	0.32
**Random effects model**	**29**	**25**	**−0.15**	**[−0.69; 0.39]**	
Heterogeneity: I^2 = 0%, τ^2 = 0, p = 0.8416					

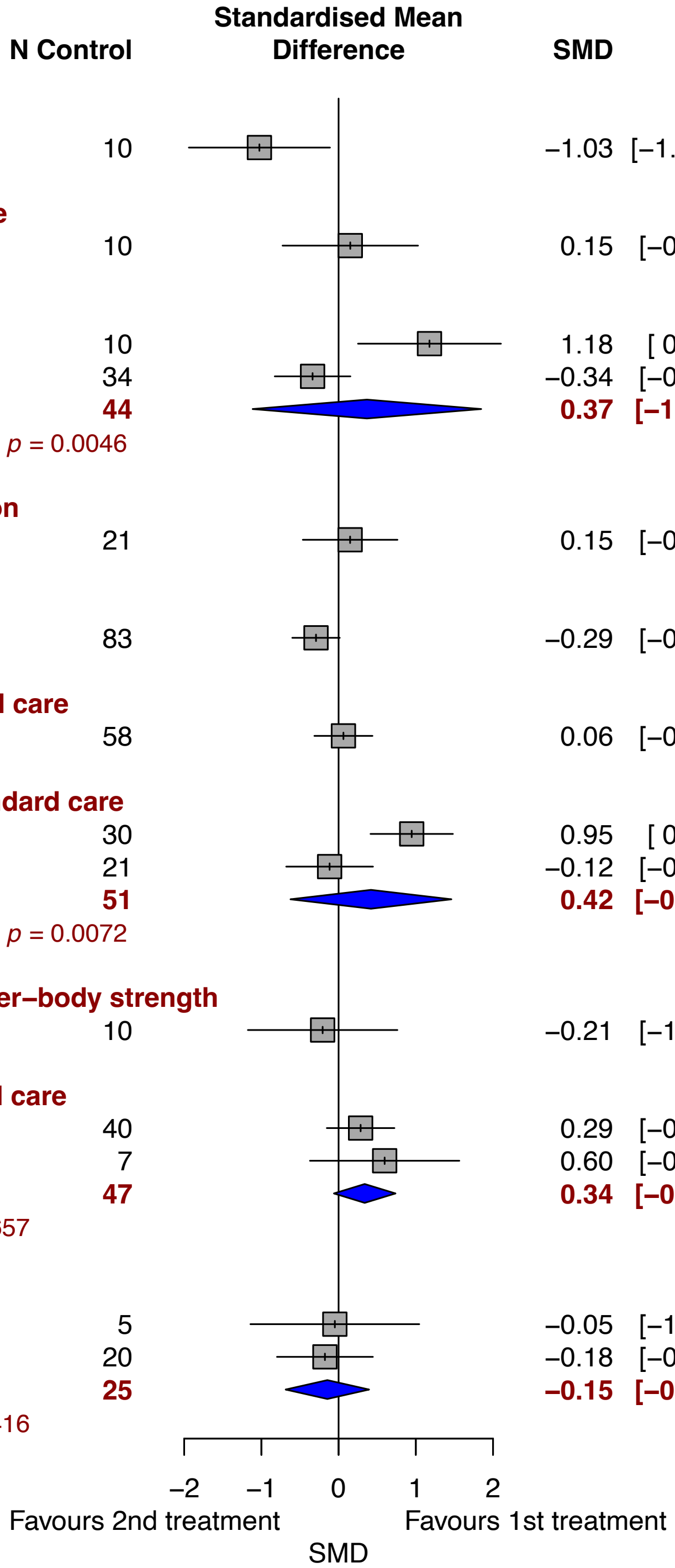

Study-level pairwise treatment comparisons for pain outcomes at 3 months after TKA. Results are presented as standardized mean differences (SMD) with 95% confidence intervals (CI). Positive SMDs favor the first treatment listed in each comparison; negative SMDs favor the second treatment. BFR, blood flow restriction; NMES, neuromuscular electrical stimulation.

S8D. Forest Plots of Study-Level Pairwise Comparisons (3 Months)

Health-Related Quality of Life

Study	N Prehab.	N Control	SMD	95% CI	SE
**Aerobic exercise vs BFR exercise**					
Franz 2022	10	10	−1.14	[−2.06; −0.22]	0.47
**Aerobic exercise vs Standard care**					
Franz 2022	10	10	−0.17	[−1.05; 0.71]	0.45
**BFR exercise vs Standard care**					
Franz 2022	10	10	0.97	[0.06; 1.88]	0.46
Jorgensen 2024	31	34	0.22	[−0.27; 0.70]	0.25
**Random effects model**	**41**	**44**	**0.49**	**[−0.22; 1.20]**	
Heterogeneity: $I^2 = 51\%$, $\tau^2 = 0.1442$, $p = 0.1533$					
**Education vs Exercise & Education**					
Huber 2015	20	21	−0.28	[−0.90; 0.33]	0.31
**Education vs Standard care**					
Nam 2023	81	83	0.05	[−0.26; 0.35]	0.16
**Exercise & Education vs Standard care**					
Beaupre 2004	51	58	−0.00	[−0.38; 0.38]	0.19
**Lower−extremity strength vs Standard care**					
Skoffer 2016	29	21	0.24	[−0.32; 0.80]	0.29
**Lower−extremity strength vs Upper−body strength**					
McKay 2012	7	10	0.65	[−0.34; 1.64]	0.51
**Multidomain exercise vs Standard care**					
Villadsen 2014	41	40	0.24	[−0.19; 0.68]	0.22
**NMES vs Standard care**					
Savkin 2021	20	20	0.21	[−0.41; 0.83]	0.32
Walls 2010	9	5	0.00	[−1.09; 1.10]	0.56
**Random effects model**	**29**	**25**	**0.16**	**[−0.38; 0.70]**	
Heterogeneity: $I^2 = 0\%$, $\tau^2 = 0$, $p = 0.7468$					

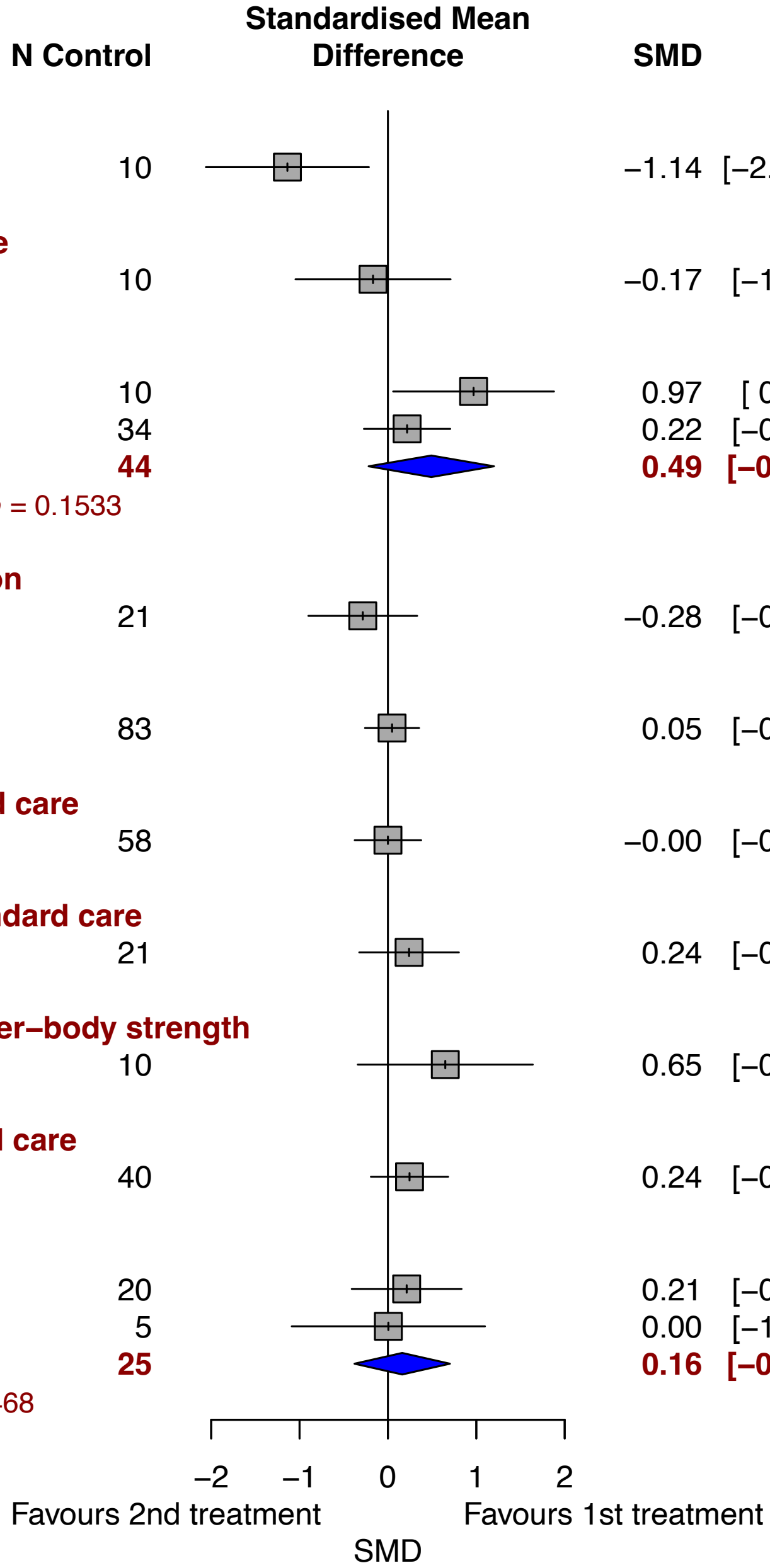

Study-level pairwise treatment comparisons for health-related quality of life outcomes at 3 months after TKA. Results are presented as standardized mean differences (SMD) with 95% confidence intervals (CI). Positive SMDs favor the first treatment listed in each comparison; negative SMDs favor the second treatment. BFR, blood flow restriction; NMES, neuromuscular electrical stimulation.

S9. Risk of Bias Assessments at the Outcome Level

Physical Function

STUDY	TREATMENT 2	TREATMENT 1	OUTCOME MEASURE	ASSESSMENT TIME 4–8 wk	ASSESSMENT TIME 12–14 wk	D1	D2	D3	D4	D5	OVERALL
Franz et al. (2022)	Aerobic exercise	Standard care	KOOS ADL	-	3 months	+	+	+	!	!	!
Franz et al. (2022)	BFR exercise	Aerobic exercise	KOOS ADL	-	3 months	+	+	+	!	!	!
Franz et al. (2022)	BFR exercise	Standard care	KOOS ADL	-	3 months	+	+	+	!	!	!
Jorgensen et al. (2024)	BFR exercise	Standard care	KOOS ADL	-	3 months	+	+	+	!	+	!
Nam et al. (2023)	Education	Standard care	WOMAC Function	-	3 months	+	!	+	+	+	!
Beaupre et al. (2004)	Exercise & Education	Standard care	WOMAC Function	-	3 months	+	!	+	!	!	!
Huber et al. (2015)	Exercise & Education	Education	KOOS ADL	6 wk	3 months	+	+	+	!	+	!
Blasco et al. (2020)[✦]	Home-based multidomain	Standard care	KOOS ADL	6 wk	-	+	-	!	!	+	-
Blasco et al. (2020)[✦]	Home-based multidomain	Multidomain exercise	KOOS ADL	6 wk	-	+	-	!	!	+	-
McKay et al. (2012)	Lower-extremity strength	Upper-body strength	WOMAC Function	6 wk	12 wk	+	!	+	!	!	!
Calatayud et al. (2017)[✦]	Lower-extremity strength	Standard care	WOMAC Function	1 month	3 months	+	-	!	!	!	-
Dominguez-Navarro et al. (2021)[✦]	Lower-extremity strength	Standard care	KOOS Pain	6 wk	-	+	-	!	!	+	-
Skoffer et al. (2016)	Lower-extremity strength	Standard care	KOOS ADL	6 wk	12 wk	+	!	+	!	+	!
Tungtrongjit et al. (2012)[✦]	Lower-extremity strength	Standard care	WOMAC Function	1 month	3 months	+	-	!	!	!	-
Rooks et al. (2006)	Multidomain exercise	Education	WOMAC Function	8 wk	-	+	!	+	!	!	!
Dominguez-Navarro et al. (2021)[✦]	Multidomain exercise	Lower-extremity strength	KOOS ADL	6 wk	-	+	-	!	!	+	-
Blasco et al. (2020)[✦]	Multidomain exercise	Standard care	KOOS ADL	6 wk	-	+	-	!	!	+	-
Brown et al. (2012)[✦]	Multidomain exercise	Standard care	SF-36 Phys. funct.	-	3 months	+	-	!	!	!	-
Dominguez-Navarro et al. (2021)[✦]	Multidomain exercise	Standard care	KOOS ADL	6 wk	-	+	-	!	!	+	-
Gstoettner et al. (2011)	Multidomain exercise	Standard care	WOMAC Function	6 wk	-	+	+	+	!	!	!
Villadsen et al. (2014)	Multidomain exercise	Standard care	KOOS ADL	6 wk	3 months	+	!	+	!	+	!
Savkin et al. (2021)	NMES	Standard care	KOOS ADL	4 wk	12 wk	+	!	+	!	!	!
Walls et al. (2010)	NMES	Standard care	WOMAC Function	6 wk	12 wk	+	!	+	!	!	!

Quadriceps Strength

STUDY	TREATMENT 2	TREATMENT 1	OUTCOME MEASURE	ASSESSMENT TIME 4–8 wk	ASSESSMENT TIME 12–14 wk	D1	D2	D3	D4	D5	OVERALL
Franz et al. (2022)	Aerobic exercise	Standard care	Knee ext.	-	3 months	+	+	+	+	!	!
Franz et al. (2022)	BFR exercise	Aerobic exercise	Knee ext.	-	3 months	+	+	+	+	!	!
Franz et al. (2022)	BFR exercise	Standard care	Knee ext.	-	3 months	+	+	+	+	!	!
Jorgensen et al. (2024)	BFR exercise	Standard care	Knee ext. (isom.)	-	3 months	+	+	+	+	+	+
Beaupre et al. (2004)	Exercise & Education	Standard care	Knee ext. (isom.)	-	3 months	+	!	+	+	!	!
Huber et al. (2015)	Exercise & Education	Education	Knee ext. (isom.)	6 wk	3 months	+	+	+	+	+	+
McKay et al. (2012)	Lower-extremity strength	Upper-body strength	Knee ext. (isom.)	6 wk	12 wk	+	!	+	!	!	!
Blasco et al. (2020)[✦]	Home-based multidomain	Standard care	Knee ext. (isom.)	6 wk	-	+	-	!	+	+	-
Blasco et al. (2020)[✦]	Home-based multidomain	Multidomain exercise	Knee ext. (isom.)	6 wk	-	+	-	!	+	+	-
Calatayud et al. (2017)[✦]	Lower-extremity strength	Standard care	Knee ext. (isom.)	1 month	3 months	+	-	!	+	!	-
Dominguez-Navarro et al. (2021)[✦]	Lower-extremity strength	Standard care	Knee ext.	6 wk	-	+	-	!	+	+	-
van Leeuwen et al. (2014)	Lower-extremity strength	Standard care	Knee ext. (isom.)	6 wk	12 wk	-	-	+	!	!	-
Skoffer et al. (2016)	Lower-extremity strength	Standard care	Knee ext. (isom.)	6 wk	12 wk	+	!	+	+	+	!
Tungtrongjit et al. (2012)[✦]	Lower-extremity strength	Standard care	Knee ext.	1 month	3 months	+	-	!	+	!	-
Dominguez-Navarro et al. (2021)[✦]	Multidomain exercise	Lower-extremity strength	Knee ext.	6 wk	-	+	-	!	+	+	-
Blasco et al. (2020)[✦]	Multidomain exercise	Standard care	Knee ext. (isom.)	6 wk	-	+	-	!	+	+	-
Dominguez-Navarro et al. (2021)[✦]	Multidomain exercise	Standard care	Knee ext.	6 wk	-	+	-	!	+	+	-
Sun et al. (2023)	Multidomain exercise	Standard care	Knee ext. (isom.)	-	3 months	+	!	-	+	+	-
Topp et al. (2009)	Multidomain exercise	Standard care	Knee ext.	1 month	3 months	+	+	+	+	!	!
Villadsen et al. (2014)	Multidomain exercise	Standard care	Knee ext.	6 wk	3 months	+	!	+	+	+	!
Savkin et al. (2021)	NMES	Standard care	Knee ext.	4 wk	12 wk	+	!	+	+	!	!
Walls et al. (2010)	NMES	Standard care	Knee ext. (isom.)	6 wk	12 wk	+	!	+	+	!	!

Pain

STUDY	TREATMENT 2	TREATMENT 1	OUTCOME MEASURE	ASSESSMENT TIME 4–8 wk	ASSESSMENT TIME 12–14 wk	D1	D2	D3	D4	D5	OVERALL
Franz et al. (2022)	Aerobic exercise	Standard care	KOOS Pain	-	3 months	+	+	+	!	!	!
Franz et al. (2022)	BFR exercise	Aerobic exercise	KOOS Pain	-	3 months	+	+	+	!	!	!
Franz et al. (2022)	BFR exercise	Standard care	KOOS Pain	-	3 months	+	+	+	!	!	!
Jorgensen et al. (2024)	BFR exercise	Standard care	KOOS Pain	-	3 months	+	+	+	!	+	!
Nam et al. (2023)	Education	Standard care	WOMAC Pain	-	3 months	+	!	+	+	+	!
Beaupre et al. (2004)	Exercise & Education	Standard care	WOMAC Pain	-	3 months	+	!	+	!	!	!
Huber et al. (2015)	Exercise & Education	Education	KOOS Pain	6 wk	3 months	+	+	+	!	+	!
Blasco et al. (2020)✦	Home-based multidomain	Standard care	KOOS Pain	6 wk	-	+	-	!	!	+	-
Blasco et al. (2020)✦	Home-based multidomain	Multidomain exercise	KOOS Pain	6 wk	-	+	-	!	!	+	-
McKay et al. (2012)	Lower-extremity strength	Upper-body strength	WOMAC Pain	6 wk	12 wk	+	!	+	!	!	!
Calatayud et al. (2017)✦	Lower-extremity strength	Standard care	WOMAC Pain	1 month	3 months	+	-	!	!	!	-
Dominguez-Navarro et al. (2021)✦	Lower-extremity strength	Standard care	KOOS Pain	6 wk	-	+	-	!	!	+	-
Skoffer et al. (2016)	Lower-extremity strength	Standard care	KOOS Pain	6 wk	12 wk	+	!	+	!	+	!
Tungtrongjit et al. (2012)✦	Lower-extremity strength	Standard care	WOMAC Pain	1 month	3 months	+	-	!	!	!	-
Rooks et al. (2006)	Multidomain exercise	Education	WOMAC Pain	8 wk	12 wk	+	!	+	!	!	!
Dominguez-Navarro et al. (2021)✦	Multidomain exercise	Lower-extremity strength	KOOS Pain	6 wk	-	+	-	!	!	+	-
Blasco et al. (2020)✦	Multidomain exercise	Standard care	KOOS Pain	6 wk	-	+	-	!	!	+	-
Brown et al. (2012)✦	Multidomain exercise	Standard care	SF-36 Pain	-	3 months	+	-	!	!	!	-
Dominguez-Navarro et al. (2021)✦	Multidomain exercise	Standard care	KOOS Pain	6 wk	-	+	-	!	!	+	-
Gstoettner et al. (2011)	Multidomain exercise	Standard care	WOMAC Pain	6 wk	-	+	+	+	!	!	!
Villadsen et al. (2014)	Multidomain exercise	Standard care	KOOS Pain	6 wk	3 months	+	!	+	!	+	!
Savkin et al. (2021)	NMES	Standard care	KOOS Pain	4 wk	12 wk	+	!	+	!	!	!
Walls et al. (2010)	NMES	Standard care	WOMAC Pain	6 wk	12 wk	+	!	+	!	!	!

Health-Related Quality of Life

STUDY	TREATMENT 2	TREATMENT 1	OUTCOME MEASURE	ASSESSMENT TIME 4–8 wk	ASSESSMENT TIME 12–14 wk	D1	D2	D3	D4	D5	OVERALL
Franz et al. (2022)	Aerobic exercise	Standard care	KOOS QoL	-	3 months	+	+	+	!	!	!
Franz et al. (2022)	BFR exercise	Aerobic exercise	KOOS QoL	-	3 months	+	+	+	!	!	!
Franz et al. (2022)	BFR exercise	Standard care	KOOS QoL	-	3 months	+	+	+	!	!	!
Jorgensen et al. (2024)	BFR exercise	Standard care	KOOS QoL	-	3 months	+	+	+	!	+	!
Nam et al. (2023)	Education	Standard care	SF-36 PCS	-	3 months	+	!	+	+	+	!
Beaupre et al. (2004)	Exercise & Education	Standard care	SF-36 PCS	-	3 months	+	!	+	!	!	!
Huber et al. (2015)	Exercise & Education	Education	KOOS QoL	6 wk	3 months	+	+	+	!	+	!
Blasco et al. (2020)✦	Home-based multidomain	Standard care	KOOS QoL	6 wk	-	+	-	!	!	+	-
Blasco et al. (2020)✦	Home-based multidomain	Multidomain exercise	KOOS QoL	6 wk	-	+	-	!	!	+	-
McKay et al. (2012)	Lower-extremity strength	Upper-body strength	SF-36 PCS	6 wk	12 wk	+	!	+	!	!	!
Dominguez-Navarro et al. (2021)✦	Lower-extremity strength	Standard care	KOOS QoL	6 wk	-	+	-	!	!	+	-
Skoffer et al. (2016)	Lower-extremity strength	Standard care	KOOS QoL	6 wk	12 wk	+	!	+	!	+	!
Dominguez-Navarro et al. (2021)✦	Multidomain exercise	Lower-extremity strength	KOOS QoL	6 wk	-	+	-	!	!	+	-
Blasco et al. (2020)✦	Multidomain exercise	Standard care	KOOS QoL	6 wk	-	+	-	!	!	+	-
Dominguez-Navarro et al. (2021)✦	Multidomain exercise	Standard care	KOOS QoL	6 wk	-	+	-	!	!	+	-
Villadsen et al. (2014)	Multidomain exercise	Standard care	KOOS QoL	6 wk	3 months	+	+	+	!	+	!
Savkin et al. (2021)	NMES	Standard care	KOOS QoL	4 wk	12 wk	+	!	+	!	!	!
Walls et al. (2010)	NMES	Standard care	SF-36 PCS	6 wk	12 wk	+	!	+	!	!	!

D1: Randomization process, D2: Deviations from the intended interventions; D3: Missing outcome data; D4: Measurement of the outcome; D5: Selection of the reported result.

✦Studies excluded patients with postoperative complications, contributing to high risk of bias in Domain 2. ADL, Activities of Daily Living; BFR, blood flood restriction; ext, extension; isom, isometric; KOOS, Knee Injury and Osteoarthritis Outcome Score; NMES, neuromuscular electrical stimulation; PCS, Physical Component Summary; Phys. funct, physical functioning, QoL, Quality of Life; SF-36, 36-Item Short Form Survey;wk, week; WOMAC: Western Ontario and McMaster Universities Osteoarthritis Index.

Low risk + Some concerns ! High risk -

S10A. CINeMA Assessment of Network Confidence (3 Months)

Physical Function

TREATMENT COMPARISON	NUMBER of STUDIES	WITHIN-STUDY BIAS	REPORTING BIAS	INDIRECTNESS	IMPRECISION	HETEROGENEITY	INCOHERENCE	CONFIDENCE RATING
MIXED ESTIMATES								
Aerobic exercise : BFR exercise	1	Some concerns	Low risk	Some concerns	Some concerns	Some concerns	Some concerns	**Low**
Aerobic exercise : Standard care	1	Some concerns	Low risk	Some concerns	Major concerns	No concerns	Some concerns	**Low**
BFR exercise : Standard care	2	Some concerns	Low risk	Some concerns	Some concerns	Some concerns	No concerns	**Low**
Education : Exercise & Education	1	Some concerns	Low risk	Some concerns	Some concerns	Some concerns	No concerns	**Low**
Education : Standard care	1	Some concerns	Low risk	Some concerns	No concerns	Major concerns	No concerns	**Low**
Exercise & Education : Standard care	1	Some concerns	Low risk	Some concerns	Some concerns	Some concerns	No concerns	**Low**
Lower-extremity strength : Standard care	2	Major concerns	Low risk	Some concerns	No concerns	Some concerns	No concerns	**Low**
Lower-extremity strength : Upper-body strength	1	Some concerns	Low risk	Some concerns	Major concerns	No concerns	No concerns	**Low**
Multidomain exercise : Standard care	2	Some concerns	Low risk	Some concerns	Some concerns	No concerns	No concerns	**Moderate**
NMES : Standard care	2	Some concerns	Low risk	Some concerns	Some concerns	Some concerns	No concerns	**Low**
INDIRECT ESTIMATES								
Aerobic exercise : Education	0	Some concerns	Low risk	Some concerns	Major concerns	No concerns	No concerns	**Very Low**
Aerobic exercise : Exercise & Education	0	Some concerns	Low risk	Some concerns	Major concerns	No concerns	No concerns	**Very Low**
Aerobic exercise : Lower-extremity strength	0	Some concerns	Low risk	Some concerns	Some concerns	Some concerns	No concerns	**Low**
Aerobic exercise : Multidomain exercise	0	Some concerns	Low risk	Some concerns	Some concerns	Some concerns	No concerns	**Low**
Aerobic exercise : NMES	0	Some concerns	Low risk	Some concerns	Major concerns	No concerns	No concerns	**Very Low**
Aerobic exercise : Upper-body strength	0	Some concerns	Low risk	Some concerns	Major concerns	No concerns	No concerns	**Very Low**
BFR exercise : Education	0	Some concerns	Low risk	Some concerns	Some concerns	Some concerns	No concerns	**Low**
BFR exercise : Exercise & Education	0	Some concerns	Low risk	Some concerns	Some concerns	Some concerns	No concerns	**Low**
BFR exercise : Lower-extremity strength	0	Some concerns	Low risk	Some concerns	Major concerns	No concerns	No concerns	**Very Low**
BFR exercise : Multidomain exercise	0	Some concerns	Low risk	Some concerns	Major concerns	No concerns	No concerns	**Very Low**
BFR exercise : NMES	0	Some concerns	Low risk	Some concerns	Major concerns	No concerns	No concerns	**Very Low**
BFR exercise : Upper-body strength	0	Some concerns	Low risk	Some concerns	Major concerns	No concerns	No concerns	**Very Low**
Education : Lower-extremity strength	0	Some concerns	Low risk	Some concerns	Some concerns	No concerns	No concerns	**Low**
Education : Multidomain exercise	0	Some concerns	Low risk	Some concerns	Some concerns	No concerns	No concerns	**Low**
Education : NMES	0	Some concerns	Low risk	Some concerns	Major concerns	No concerns	No concerns	**Very Low**
Education : Upper-body strength	0	Some concerns	Low risk	Some concerns	Major concerns	No concerns	No concerns	**Very Low**
Exercise & Education : Lower-extremity strength	0	Some concerns	Low risk	Some concerns	Some concerns	No concerns	No concerns	**Low**
Exercise & Education : Multidomain exercise	0	Some concerns	Low risk	Some concerns	Some concerns	No concerns	No concerns	**Low**
Exercise & Education : Upper-body strength	0	Some concerns	Low risk	Some concerns	Major concerns	No concerns	No concerns	**Very Low**
Lower-extremity strength : Multidomain exercise	0	Some concerns	Low risk	Some concerns	Major concerns	No concerns	No concerns	**Very Low**
Multidomain exercise : Upper-body strength	0	Some concerns	Low risk	Some concerns	Major concerns	No concerns	No concerns	**Very Low**
NMES : Exercise & Education	0	Some concerns	Low risk	Some concerns	Major concerns	No concerns	No concerns	**Very Low**
NMES : Lower-extremity strength	0	Some concerns	Low risk	Some concerns	Some concerns	Some concerns	No concerns	**Low**
NMES : Multidomain exercise	0	Some concerns	Low risk	Some concerns	Some concerns	Some concerns	No concerns	**Low**
NMES : Upper-body strength	0	Some concerns	Low risk	Some concerns	Major concerns	No concerns	No concerns	**Very Low**
Standard care : Upper-body strength	0	Some concerns	Low risk	Some concerns	Major concerns	No concerns	No concerns	**Very Low**

CINeMA: Confidence in Network Meta-Analysis for physical function at 3 Months after TKA. Six domains influencing confidence in network meta-analysis results are assessed: (a) within-study bias, (b) reporting bias, (c) indirectness, (d) imprecision, (e) heterogeneity, and (f) incoherence. Confidence levels are categorized as high, moderate, low, or very low. Imprecision, heterogeneity, and incoherence were assessed using a clinically important SMD threshold of ±0.5, considering the position of confidence intervals, prediction intervals, and direct–indirect disagreement relative to this threshold. BFR, blood flow restriction; NMES, neuromuscular electrical stimulation.

S10B. CINeMA Assessment of Network Confidence (3 Months)

Quadriceps Strength

TREATMENT COMPARISON	NUMBER of STUDIES	WITHIN-STUDY BIAS	REPORTING BIAS	INDIRECTNESS	IMPRECISION	HETEROGENEITY	INCOHERENCE	CONFIDENCE RATING
MIXED ESTIMATES								
Aerobic exercise : BFR exercise	1	Some concerns	Low risk	Some concerns	No concerns	Major concerns	No concerns	**Low**
Aerobic exercise : Standard care	1	Some concerns	Low risk	Some concerns	Major concerns	No concerns	No concerns	**Low**
BFR exercise : Standard care	2	No concerns	Low risk	Some concerns	Some concerns	Some concerns	No concerns	**Moderate**
Education : Exercise & Education	1	No concerns	Low risk	Some concerns	Major concerns	No concerns	No concerns	**Low**
Exercise & Education : Standard care	1	Some concerns	Low risk	Some concerns	Major concerns	No concerns	No concerns	**Low**
Lower-extremity strength : Standard care	3	Major concerns	Low risk	Some concerns	Some concerns	Some concerns	No concerns	**Low**
Lower-extremity strength : Upper-body strength	1	Some concerns	Low risk	Some concerns	Major concerns	No concerns	No concerns	**Low**
Multidomain exercise : Standard care	3	Some concerns	Low risk	Some concerns	Some concerns	Some concerns	No concerns	**Low**
NMES : Standard care	2	Some concerns	Low risk	Some concerns	Some concerns	Some concerns	No concerns	**Low**
INDIRECT ESTIMATES								
Aerobic exercise : Education	0	Some concerns	Low risk	Some concerns	Major concerns	No concerns	No concerns	**Very Low**
Aerobic exercise : Exercise & Education	0	Some concerns	Low risk	Some concerns	Major concerns	No concerns	No concerns	**Very Low**
Aerobic exercise : Lower-extremity strength	0	Some concerns	Low risk	Some concerns	Some concerns	Some concerns	No concerns	**Low**
Aerobic exercise : Multidomain exercise	0	Some concerns	Low risk	Some concerns	Some concerns	Some concerns	No concerns	**Low**
Aerobic exercise : NMES	0	Some concerns	Low risk	Some concerns	Some concerns	Some concerns	No concerns	**Low**
Aerobic exercise : Upper-body strength	0	Some concerns	Low risk	Some concerns	Major concerns	No concerns	No concerns	**Very Low**
BFR exercise : Education	0	No concerns	Low risk	Some concerns	Major concerns	No concerns	No concerns	**Low**
BFR exercise : Exercise & Education	0	Some concerns	Low risk	Some concerns	Some concerns	Some concerns	No concerns	**Low**
BFR exercise : Lower-extremity strength	0	Some concerns	Low risk	Some concerns	Major concerns	No concerns	No concerns	**Very Low**
BFR exercise : Multidomain exercise	0	Some concerns	Low risk	Some concerns	Major concerns	No concerns	No concerns	**Very Low**
BFR exercise : NMES	0	Some concerns	Low risk	Some concerns	Major concerns	No concerns	No concerns	**Very Low**
BFR exercise : Upper-body strength	0	Some concerns	Low risk	Some concerns	Major concerns	No concerns	No concerns	**Very Low**
Education : Lower-extremity strength	0	Some concerns	Low risk	Some concerns	Major concerns	No concerns	No concerns	**Very Low**
Education : Multidomain exercise	0	Some concerns	Low risk	Some concerns	Major concerns	No concerns	No concerns	**Very Low**
Education : NMES	0	Some concerns	Low risk	Some concerns	Major concerns	No concerns	No concerns	**Very Low**
Education : Standard care	0	Some concerns	Low risk	Some concerns	Major concerns	No concerns	No concerns	**Very Low**
Education : Upper-body strength	0	Some concerns	Low risk	Some concerns	Major concerns	No concerns	No concerns	**Very Low**
Exercise & Education : Lower-extremity strength	0	Some concerns	Low risk	Some concerns	Major concerns	No concerns	No concerns	**Very Low**
Exercise & Education : Multidomain exercise	0	Some concerns	Low risk	Some concerns	Major concerns	No concerns	No concerns	**Very Low**
Exercise & Education : Upper-body strength	0	Some concerns	Low risk	Some concerns	Major concerns	No concerns	No concerns	**Very Low**
Lower-extremity strength : Multidomain exercise	0	Some concerns	Low risk	Some concerns	Major concerns	No concerns	No concerns	**Very Low**
Multidomain exercise : Upper-body strength	0	Some concerns	Low risk	Some concerns	Major concerns	No concerns	No concerns	**Very Low**
NMES : Exercise & Education	0	Some concerns	Low risk	Some concerns	Major concerns	No concerns	No concerns	**Very Low**
NMES : Lower-extremity strength	0	Some concerns	Low risk	Some concerns	Major concerns	No concerns	No concerns	**Very Low**
NMES : Multidomain exercise	0	Some concerns	Low risk	Some concerns	Major concerns	No concerns	No concerns	**Very Low**
NMES : Upper-body strength	0	Some concerns	Low risk	Some concerns	Major concerns	No concerns	No concerns	**Very Low**
Standard care : Upper-body strength	0	Some concerns	Low risk	Some concerns	Major concerns	No concerns	No concerns	**Very Low**

CINeMA: Confidence in Network Meta-Analysis for quadriceps strength at 3 Months after TKA. Six domains influencing confidence in network meta-analysis results are assessed: (a) within-study bias, (b) reporting bias, (c) indirectness, (d) imprecision, (e) heterogeneity, and (f) incoherence. Confidence levels are categorized as high, moderate, low, or very low. Imprecision, heterogeneity, and incoherence were assessed using a clinically important SMD threshold of ±0.5, considering the position of confidence intervals, prediction intervals, and direct–indirect disagreement relative to this threshold. BFR, blood flow restriction; NMES, neuromuscular electrical stimulation.

S10C. CINeMA Assessment of Network Confidence (3 Months)

Pain

TREATMENT COMPARISON	NUMBER of STUDIES	WITHIN-STUDY BIAS	REPORTING BIAS	INDIRECTNESS	IMPRECISION	HETEROGENEITY	INCOHERENCE	CONFIDENCE RATING
MIXED ESTIMATES								
Aerobic exercise : BFR exercise	1	Some concerns	Low risk	Some concerns	Major concerns	No concerns	Some concerns	**Low**
Aerobic exercise : Standard care	1	Some concerns	Low risk	Some concerns	Major concerns	No concerns	Some concerns	**Low**
BFR exercise : Standard care	2	Some concerns	Low risk	Some concerns	Major concerns	No concerns	No concerns	**Low**
Education : Exercise & Education	1	Some concerns	Low risk	Some concerns	Major concerns	No concerns	No concerns	**Low**
Education : Standard care	1	Some concerns	Low risk	Some concerns	Major concerns	No concerns	No concerns	**Low**
Exercise & Education : Standard care	1	Some concerns	Low risk	Some concerns	Major concerns	No concerns	No concerns	**Low**
Lower-extremity strength : Standard care	2	Major concerns	Low risk	Some concerns	Some concerns	Some concerns	No concerns	**Low**
Lower-extremity strength : Upper-body strength	1	Some concerns	Low risk	Some concerns	Major concerns	No concerns	No concerns	**Low**
Multidomain exercise : Standard care	2	Some concerns	Low risk	Some concerns	Some concerns	Some concerns	No concerns	**Low**
NMES : Standard care	2	Some concerns	Low risk	Some concerns	Major concerns	No concerns	No concerns	**Low**
INDIRECT ESTIMATES								
Aerobic exercise : Education	0	Some concerns	Low risk	Some concerns	Major concerns	No concerns	No concerns	**Very Low**
Aerobic exercise : Exercise & Education	0	Some concerns	Low risk	Some concerns	Major concerns	No concerns	No concerns	**Very Low**
Aerobic exercise : Lower-extremity strength	0	Some concerns	Low risk	Some concerns	Major concerns	No concerns	No concerns	**Very Low**
Aerobic exercise : Multidomain exercise	0	Some concerns	Low risk	Some concerns	Major concerns	No concerns	No concerns	**Very Low**
Aerobic exercise : NMES	0	Some concerns	Low risk	Some concerns	Major concerns	No concerns	No concerns	**Very Low**
Aerobic exercise : Upper-body strength	0	Some concerns	Low risk	Some concerns	Major concerns	No concerns	No concerns	**Very Low**
BFR exercise : Education	0	Some concerns	Low risk	Some concerns	Major concerns	No concerns	No concerns	**Very Low**
BFR exercise : Exercise & Education	0	Some concerns	Low risk	Some concerns	Major concerns	No concerns	No concerns	**Very Low**
BFR exercise : Lower-extremity strength	0	Some concerns	Low risk	Some concerns	Major concerns	No concerns	No concerns	**Very Low**
BFR exercise : Multidomain exercise	0	Some concerns	Low risk	Some concerns	Major concerns	No concerns	No concerns	**Very Low**
BFR exercise : NMES	0	Some concerns	Low risk	Some concerns	Major concerns	No concerns	No concerns	**Very Low**
BFR exercise : Upper-body strength	0	Some concerns	Low risk	Some concerns	Major concerns	No concerns	No concerns	**Very Low**
Education : Lower-extremity strength	0	Some concerns	Low risk	Some concerns	Major concerns	No concerns	No concerns	**Very Low**
Education : Multidomain exercise	0	Some concerns	Low risk	Some concerns	Major concerns	No concerns	No concerns	**Very Low**
Education : NMES	0	Some concerns	Low risk	Some concerns	Major concerns	No concerns	No concerns	**Very Low**
Education : Upper-body strength	0	Some concerns	Low risk	Some concerns	Major concerns	No concerns	No concerns	**Very Low**
Exercise & Education : Lower-extremity strength	0	Some concerns	Low risk	Some concerns	Major concerns	No concerns	No concerns	**Very Low**
Exercise & Education : Multidomain exercise	0	Some concerns	Low risk	Some concerns	Major concerns	No concerns	No concerns	**Very Low**
Exercise & Education : Upper-body strength	0	Some concerns	Low risk	Some concerns	Major concerns	No concerns	No concerns	**Very Low**
Lower-extremity strength : Multidomain exercise	0	Some concerns	Low risk	Some concerns	Major concerns	No concerns	No concerns	**Very Low**
Multidomain exercise : Upper-body strength	0	Some concerns	Low risk	Some concerns	Major concerns	No concerns	No concerns	**Very Low**
NMES : Exercise & Education	0	Some concerns	Low risk	Some concerns	Major concerns	No concerns	No concerns	**Very Low**
NMES : Lower-extremity strength	0	Some concerns	Low risk	Some concerns	Major concerns	No concerns	No concerns	**Very Low**
NMES : Multidomain exercise	0	Some concerns	Low risk	Some concerns	Major concerns	No concerns	No concerns	**Very Low**
NMES : Upper-body strength	0	Some concerns	Low risk	Some concerns	Major concerns	No concerns	No concerns	**Very Low**
Standard care : Upper-body strength	0	Some concerns	Low risk	Some concerns	Major concerns	No concerns	No concerns	**Very Low**

CINeMA: Confidence in Network Meta-Analysis for pain at 3 Months after TKA. Six domains influencing confidence in network meta-analysis results are assessed: (a) within-study bias, (b) reporting bias, (c) indirectness, (d) imprecision, (e) heterogeneity, and (f) incoherence. Confidence levels are categorized as high, moderate, low, or very low. Imprecision, heterogeneity, and incoherence were assessed using a clinically important SMD threshold of ±0.5, considering the position of confidence intervals, prediction intervals, and direct–indirect disagreement relative to this threshold. BFR, blood flow restriction; NMES, neuromuscular electrical stimulation.

S10D. CINeMA Assessment of Network Confidence (3 Months)

Health-Related Quality of Life

TREATMENT COMPARISON	NUMBER of STUDIES	WITHIN-STUDY BIAS	REPORTING BIAS	INDIRECTNESS	IMPRECISION	HETEROGENEITY	INCOHERENCE	CONFIDENCE RATING
MIXED ESTIMATES								
Aerobic exercise : BFR exercise	1	Some concerns	Low risk	Some concerns	No concerns	Some concerns	No concerns	**Low**
Aerobic exercise : Standard care	1	Some concerns	Low risk	Some concerns	Some concerns	Some concerns	No concerns	**Low**
BFR exercise : Standard care	2	Some concerns	Low risk	Some concerns	Some concerns	No concerns	No concerns	**Low**
Education : Exercise & Education	1	Some concerns	Low risk	Some concerns	No concerns	Major concerns	No concerns	**Low**
Education : Standard care	1	Some concerns	Low risk	Some concerns	No concerns	No concerns	No concerns	**Moderate**
Exercise & Education : Standard care	1	Some concerns	Low risk	Some concerns	No concerns	Some concerns	No concerns	**Low**
Lower-extremity strength : Standard care	1	Some concerns	Low risk	Some concerns	Some concerns	Some concerns	No concerns	**Low**
Lower-extremity strength : Upper-body strength	1	Some concerns	Low risk	Some concerns	Some concerns	Some concerns	No concerns	**Low**
Multidomain exercise : Standard care	1	Some concerns	Low risk	Some concerns	Some concerns	No concerns	No concerns	**Low**
NMES : Standard care	2	Some concerns	Low risk	Some concerns	Some concerns	Some concerns	No concerns	**Low**
INDIRECT ESTIMATES								
Aerobic exercise : Education	0	Some concerns	Low risk	Some concerns	Some concerns	Some concerns	No concerns	**Low**
Aerobic exercise : Exercise & Education	0	Some concerns	Low risk	Some concerns	Some concerns	Some concerns	No concerns	**Low**
Aerobic exercise : Lower-extremity strength	0	Some concerns	Low risk	Some concerns	Some concerns	Some concerns	No concerns	**Low**
Aerobic exercise : Multidomain exercise	0	Some concerns	Low risk	Some concerns	Some concerns	Some concerns	No concerns	**Low**
Aerobic exercise : NMES	0	Some concerns	Low risk	Some concerns	Some concerns	Some concerns	No concerns	**Low**
Aerobic exercise : Upper-body strength	0	Some concerns	Low risk	Some concerns	Major concerns	No concerns	No concerns	**Very Low**
BFR exercise : Education	0	Some concerns	Low risk	Some concerns	Some concerns	No concerns	No concerns	**Low**
BFR exercise : Exercise & Education	0	Some concerns	Low risk	Some concerns	Some concerns	Some concerns	No concerns	**Low**
BFR exercise : Lower-extremity strength	0	Some concerns	Low risk	Some concerns	Major concerns	No concerns	No concerns	**Very Low**
BFR exercise : Multidomain exercise	0	Some concerns	Low risk	Some concerns	Some concerns	Some concerns	No concerns	**Low**
BFR exercise : NMES	0	Some concerns	Low risk	Some concerns	Some concerns	Some concerns	No concerns	**Low**
BFR exercise : Upper-body strength	0	Some concerns	Low risk	Some concerns	Some concerns	Some concerns	No concerns	**Low**
Education : Lower-extremity strength	0	Some concerns	Low risk	Some concerns	Some concerns	Some concerns	No concerns	**Low**
Education : Multidomain exercise	0	Some concerns	Low risk	Some concerns	Some concerns	Some concerns	No concerns	**Low**
Education : NMES	0	Some concerns	Low risk	Some concerns	Some concerns	Some concerns	No concerns	**Low**
Education : Upper-body strength	0	Some concerns	Low risk	Some concerns	Major concerns	No concerns	No concerns	**Very Low**
Exercise & Education : Lower-extremity strength	0	Some concerns	Low risk	Some concerns	Some concerns	Some concerns	No concerns	**Low**
Exercise & Education : Multidomain exercise	0	Some concerns	Low risk	Some concerns	Some concerns	Some concerns	No concerns	**Low**
Exercise & Education : Upper-body strength	0	Some concerns	Low risk	Some concerns	Major concerns	No concerns	No concerns	**Very Low**
Lower-extremity strength : Multidomain exercise	0	Some concerns	Low risk	Some concerns	Major concerns	No concerns	No concerns	**Very Low**
Multidomain exercise : Upper-body strength	0	Some concerns	Low risk	Some concerns	Major concerns	No concerns	No concerns	**Very Low**
NMES : Exercise & Education	0	Some concerns	Low risk	Some concerns	Major concerns	No concerns	No concerns	**Very Low**
NMES : Lower-extremity strength	0	Some concerns	Low risk	Some concerns	Major concerns	No concerns	No concerns	**Very Low**
NMES : Multidomain exercise	0	Some concerns	Low risk	Some concerns	Major concerns	No concerns	No concerns	**Very Low**
NMES : Upper-body strength	0	Some concerns	Low risk	Some concerns	Major concerns	No concerns	No concerns	**Very Low**
Standard care : Upper-body strength	0	Some concerns	Low risk	Some concerns	Major concerns	No concerns	No concerns	**Very Low**

CINeMA: Confidence in Network Meta-Analysis for health-related quality of life at 3 Months after TKA. Six domains influencing confidence in network meta-analysis results are assessed: (a) within-study bias, (b) reporting bias, (c) indirectness, (d) imprecision, (e) heterogeneity, and (f) incoherence. Confidence levels are categorized as high, moderate, low, or very low. Imprecision, heterogeneity, and incoherence were assessed using a clinically important SMD threshold of ±0.5, considering the position of confidence intervals, prediction intervals, and direct–indirect disagreement relative to this threshold. BFR, blood flow restriction; NMES, neuromuscular electrical stimulation.

S11. Comparison-Adjusted Funnel Plots (3 Months)

Physical Function

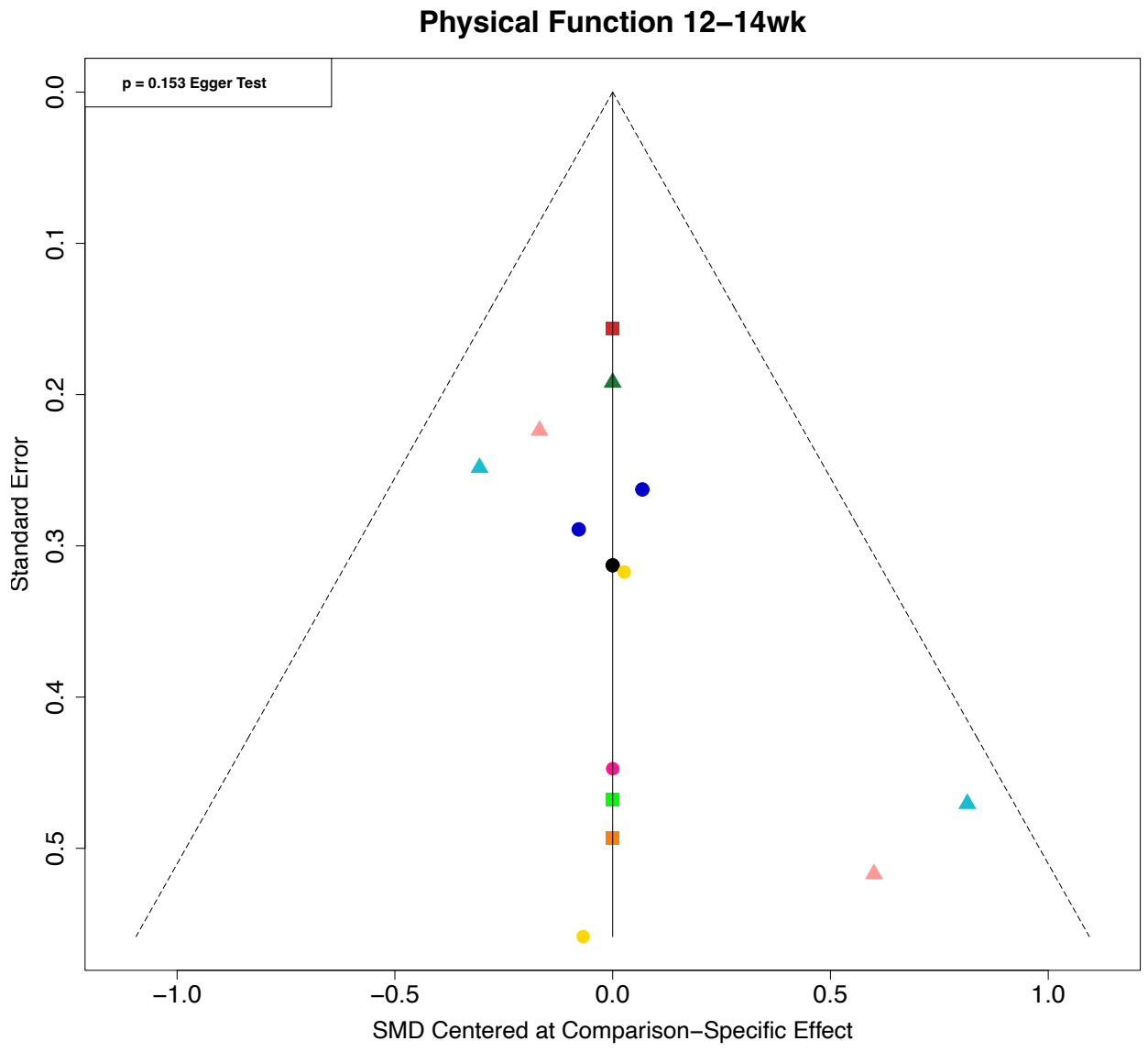

Quadriceps Strength

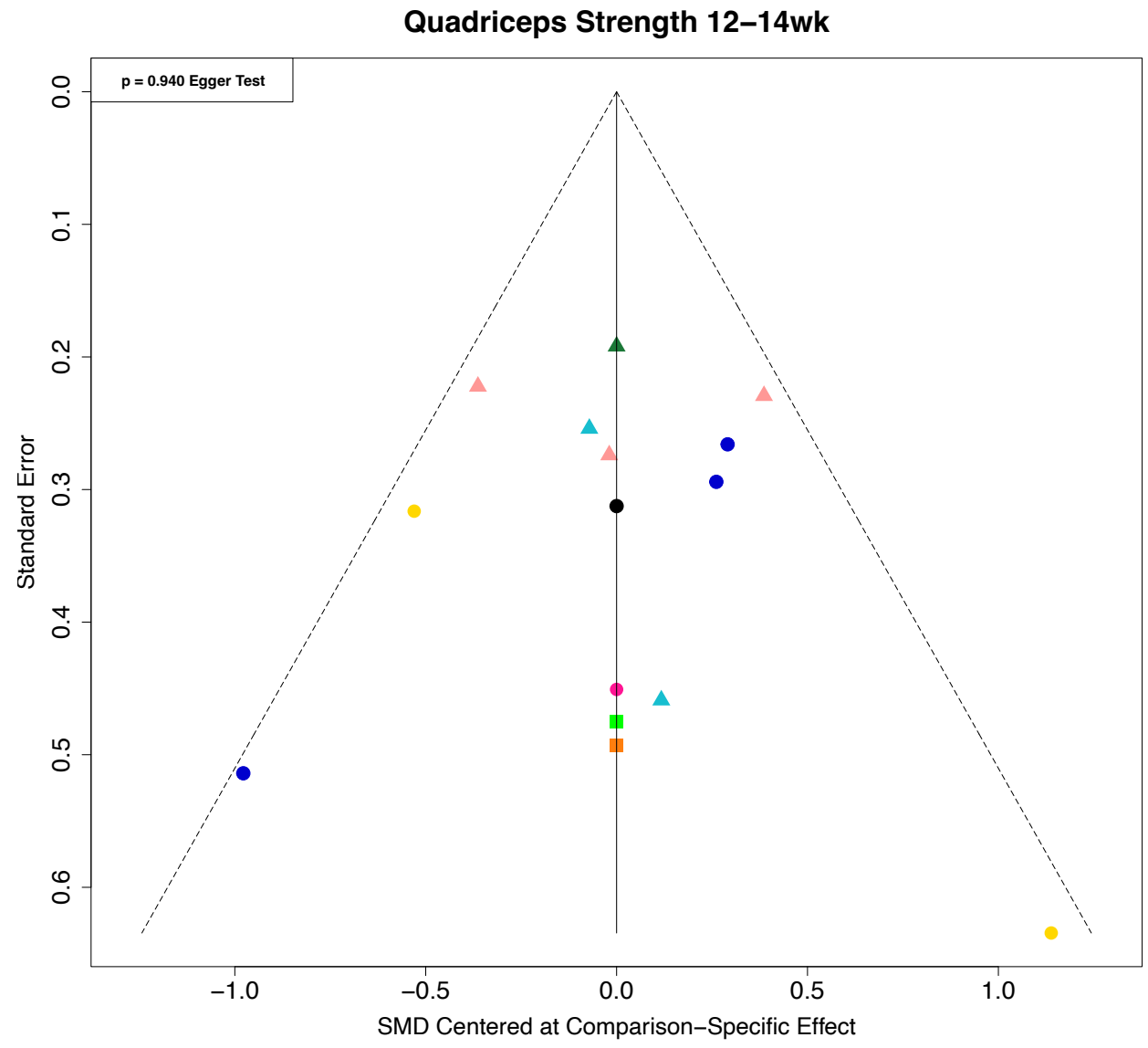

Pain

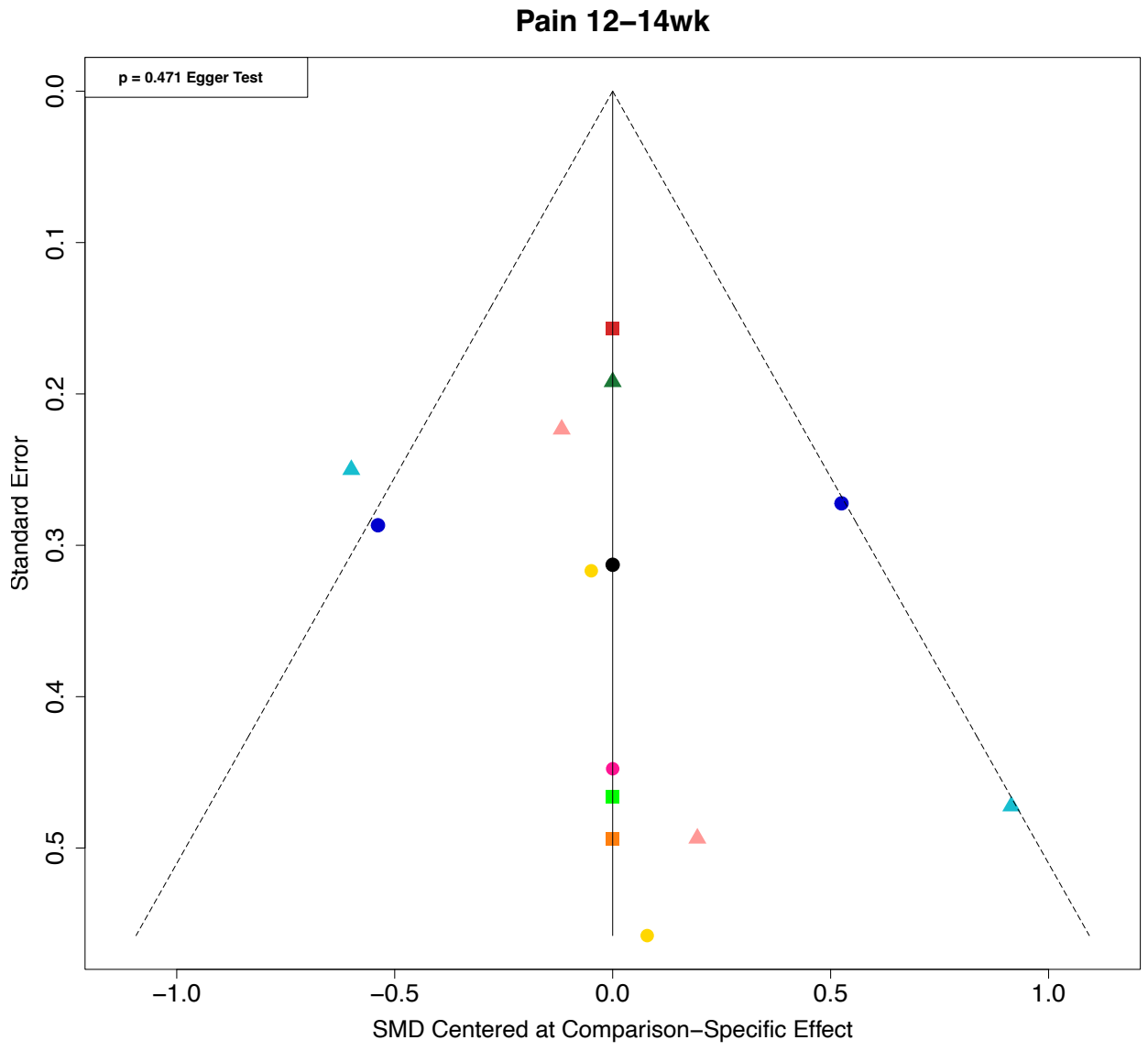

Health-Related Quality of Life

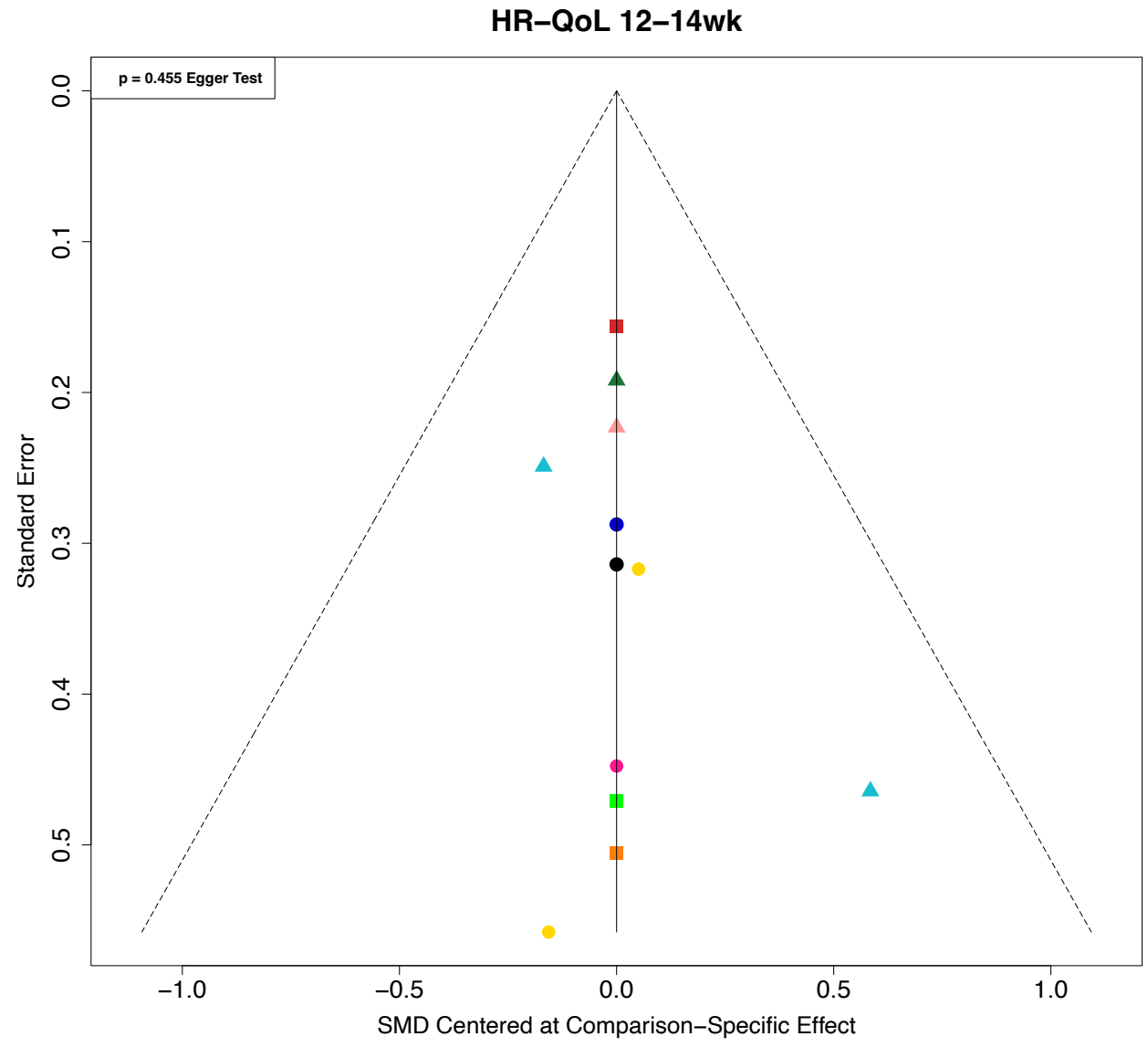

- ■ Aerobic exercise vs. BFR exercise
- ● Aerobic exercise vs. Standard care
- ▲ BFR exercise vs. Standard care
- ● Education vs. Exercise & Education
- ■ Education vs. Standard care
- ● NMES vs. Standard care
- ▲ Exercise & Education vs. Standard care
- ● Lower-extremity strength vs. Standard care
- ■ Lower-extremity strength vs. Upper-body training
- ▲ Multidomain exercise vs. standard care

Comparison-adjusted funnel plots of standardized mean differences (SMDs) centered at the comparison-specific effect. Coloured symbols indicate treatment comparisons. P-values from Egger's regression test are presented. BFR, blood flow restriction; NMES, neuromuscular electrical stimulation.

S12. League Tables Comparing Prehabilitation Modalities (3 Months)

Physical Function

**Aerobic exercise**	-1.06 (-2.05; -0.08)	.	.	.	.	.	0.07 (-0.88; 1.02)	.
-0.63 (-1.50; 0.25)	**BFR exercise**	.	.	.	.	.	0.32 (-0.20; 0.84)	.
-0.29 (-1.25; 0.67)	0.33 (-0.33; 1.00)	**Education**	.	0.15 (-0.57; 0.86)	.	.	-0.06 (-0.54; 0.41)	.
-0.51 (-1.57; 0.55)	0.12 (-0.68; 0.92)	-0.21 (-0.95; 0.53)	**NMES**	.	.	.	0.20 (-0.41; 0.81)	.
-0.25 (-1.23; 0.72)	0.37 (-0.31; 1.06)	0.04 (-0.46; 0.54)	0.25 (-0.50; 1.01)	**Exercise & Education**	.	.	0.00 (-0.52; 0.52)	.
-0.77 (-1.75; 0.21)	-0.14 (-0.84; 0.55)	-0.48 (-1.10; 0.14)	-0.27 (-1.03; 0.50)	-0.52 (-1.16; 0.12)	**LE-strength**	.	0.46 (0.00; 0.92)	0.09 (-0.94; 1.12)
-0.81 (-1.81; 0.19)	-0.18 (-0.90; 0.54)	-0.51 (-1.17; 0.14)	-0.30 (-1.09; 0.49)	-0.55 (-1.23; 0.12)	-0.03 (-0.72; 0.65)	**Multidomain exercise**	0.50 (-0.01; 1.00)	.
-0.31 (-1.17; 0.56)	0.32 (-0.20; 0.84)	-0.02 (-0.43; 0.40)	0.20 (-0.41; 0.81)	-0.06 (-0.50; 0.39)	0.46 (0.00; 0.92)	0.50 (-0.01; 1.00)	**Standard care**	.
-0.68 (-2.11; 0.74)	-0.06 (-1.30; 1.19)	-0.39 (-1.60; 0.81)	-0.18 (-1.46; 1.11)	-0.43 (-1.65; 0.78)	0.09 (-0.94; 1.12)	0.12 (-1.11; 1.36)	-0.37 (-1.50; 0.76)	**Upper-body strength**

Quadriceps Strength

**Aerobic exercise**	-1.23 (-2.46; -0.01)	.	.	.	.	.	-0.44 (-1.62; 0.75)	.
-1.17 (-2.29; -0.06)	**BFR exercise**	.	.	.	.	.	0.68 (-0.06; 1.42)	.
-0.45 (-2.18; 1.28)	0.72 (-0.80; 2.25)	**Education**	.	-0.04 (-1.05; 0.96)	.	.	.	.
-0.95 (-2.33; 0.42)	0.22 (-0.89; 1.33)	-0.51 (-2.08; 1.06)	**NMES**	.	.	.	0.46 (-0.37; 1.29)	.
-0.49 (-1.90; 0.91)	0.68 (-0.47; 1.83)	-0.04 (-1.05; 0.96)	0.46 (-0.75; 1.67)	**Exercise & Education**	.	.	0.00 (-0.88; 0.88)	.
-0.90 (-2.15; 0.36)	0.27 (-0.68; 1.23)	-0.45 (-1.91; 1.01)	0.06 (-0.97; 1.08)	-0.41 (-1.47; 0.66)	**LE-strength**	.	0.41 (-0.20; 1.01)	0.07 (-1.18; 1.32)
-0.82 (-2.05; 0.40)	0.35 (-0.56; 1.26)	-0.38 (-1.81; 1.06)	0.13 (-0.86; 1.12)	-0.33 (-1.36; 0.70)	0.07 (-0.73; 0.88)	**Multidomain exercise**	0.33 (-0.20; 0.86)	.
-0.49 (-1.59; 0.61)	0.68 (-0.06; 1.42)	-0.04 (-1.38; 1.29)	0.46 (-0.37; 1.29)	-0.00 (-0.88; 0.88)	0.41 (-0.20; 1.01)	0.33 (-0.20; 0.86)	**Standard care**	.
-0.83 (-2.60; 0.94)	0.34 (-1.23; 1.91)	-0.38 (-2.30; 1.54)	0.12 (-1.49; 1.74)	-0.34 (-1.98; 1.30)	0.07 (-1.18; 1.32)	-0.01 (-1.49; 1.48)	-0.34 (-1.73; 1.05)	**Upper-body strength**

Pain

**Aerobic exercise**	-1.03 (-2.36; 0.30)	.	.	.	.	.	0.15 (-1.15; 1.46)	.
-0.55 (-1.77; 0.66)	**BFR exercise**	.	.	.	.	.	0.26 (-0.58; 1.10)	.
-0.15 (-1.62; 1.32)	0.40 (-0.79; 1.60)	**Education**	.	0.15 (-1.00; 1.29)	.	.	-0.29 (-1.31; 0.72)	.
-0.16 (-1.67; 1.34)	0.39 (-0.84; 1.62)	-0.01 (-1.25; 1.23)	**NMES**	.	.	.	-0.13 (-1.03; 0.77)	.
-0.19 (-1.67; 1.29)	0.36 (-0.84; 1.56)	-0.04 (-0.94; 0.85)	-0.03 (-1.28; 1.21)	**Exercise & Education**	.	.	0.06 (-0.97; 1.10)	.
-0.71 (-2.15; 0.73)	-0.16 (-1.31; 0.99)	-0.56 (-1.72; 0.59)	-0.55 (-1.75; 0.65)	-0.52 (-1.68; 0.65)	**LE-strength**	.	0.42 (-0.36; 1.21)	-0.21 (-1.57; 1.16)
-0.69 (-2.16; 0.78)	-0.14 (-1.33; 1.05)	-0.54 (-1.73; 0.65)	-0.53 (-1.76; 0.70)	-0.50 (-1.70; 0.70)	0.02 (-1.13; 1.17)	**Multidomain exercise**	0.40 (-0.44; 1.24)	.
-0.29 (-1.49; 0.92)	0.26 (-0.58; 1.10)	-0.14 (-0.99; 0.71)	-0.13 (-1.03; 0.77)	-0.10 (-0.95; 0.76)	0.42 (-0.36; 1.21)	0.40 (-0.44; 1.24)	**Standard care**	.
-0.92 (-2.90; 1.07)	-0.37 (-2.15; 1.42)	-0.77 (-2.56; 1.02)	-0.76 (-2.57; 1.06)	-0.73 (-2.52; 1.07)	-0.21 (-1.57; 1.16)	-0.23 (-2.01; 1.56)	-0.63 (-2.21; 0.95)	**Upper-body strength**

Health-Related Quality of Life

**Aerobic exercise**	-1.14 (-2.06; -0.22)	.	.	.	.	.	-0.17 (-1.05; 0.71)	.
-0.82 (-1.63; -0.01)	**BFR exercise**	.	.	.	.	.	0.38 (-0.05; 0.82)	.
-0.43 (-1.28; 0.42)	0.39 (-0.13; 0.90)	**Education**	.	-0.28 (-0.90; 0.33)	.	.	0.05 (-0.26; 0.35)	.
-0.59 (-1.56; 0.37)	0.22 (-0.47; 0.91)	-0.17 (-0.77; 0.44)	**NMES**	.	.	.	0.16 (-0.38; 0.70)	.
-0.51 (-1.37; 0.36)	0.31 (-0.23; 0.85)	-0.08 (-0.46; 0.30)	0.09 (-0.55; 0.72)	**Exercise & Education**	.	.	0.00 (-0.38; 0.38)	.
-0.67 (-1.65; 0.31)	0.15 (-0.56; 0.85)	-0.24 (-0.87; 0.39)	-0.08 (-0.86; 0.70)	-0.16 (-0.82; 0.49)	**LE-strength**	.	0.24 (-0.32; 0.80)	0.65 (-0.34; 1.64)
-0.68 (-1.59; 0.23)	0.14 (-0.47; 0.75)	-0.25 (-0.77; 0.27)	-0.08 (-0.78; 0.61)	-0.17 (-0.72; 0.38)	-0.01 (-0.72; 0.71)	**Multidomain exercise**	0.24 (-0.19; 0.68)	.
-0.43 (-1.23; 0.37)	0.38 (-0.05; 0.82)	-0.00 (-0.29; 0.28)	0.16 (-0.38; 0.70)	0.08 (-0.25; 0.41)	0.24 (-0.32; 0.80)	0.24 (-0.19; 0.68)	**Standard care**	.
-0.02 (-1.41; 1.37)	0.79 (-0.42; 2.01)	0.41 (-0.77; 1.58)	0.57 (-0.69; 1.83)	0.49 (-0.70; 1.67)	0.65 (-0.34; 1.64)	0.65 (-0.57; 1.87)	0.41 (-0.73; 1.55)	**Upper-body strength**

League tables presenting standardized mean differences (SMDs) with 95% confidence intervals (CIs) for Physical function, Quadriceps strength, Pain, and Health-related quality of life across all prehabilitation modality comparisons. The upper triangle displays direct treatment effects, whereas the lower triangle presents indirect network estimates. Interpretation differs between triangles: in the upper triangle, positive SMDs favor the row-defined treatment and negative SMDs favor the column-defined treatment; in the lower triangle, positive SMDs favor the column-defined treatment and negative SMDs favor the row-defined treatment. Statistically significant comparisons ($p < 0.05$) are highlighted in red. BFR, blood flow restriction; LE, lower extremity; NMES, neuromuscular electrical stimulation.

S13A. Local Inconsistency by Treatment Comparison (3 Months)

Physical Function

Comparison	Number of Studies	Direct Evidence	I2	SMD	95% CI
**Aerobic exercise vs Standard care**					
Direct estimate	1	0.83		0.07	[−0.88; 1.02]
Indirect estimate				−2.15	[−4.24; −0.05]
Network estimate				−0.31	[−1.17; 0.56]
95% prediction interval				−0.31	[−1.54; 0.92]
**Exercise & Education vs Standard care**					
Direct estimate	1	0.73		0.00	[−0.52; 0.52]
Indirect estimate				−0.21	[−1.06; 0.65]
Network estimate				−0.06	[−0.50; 0.39]
95% prediction interval				−0.06	[−0.81; 0.70]
**Education vs Standard care**					
Direct estimate	1	0.78		−0.06	[−0.54; 0.41]
Indirect estimate				0.15	[−0.74; 1.03]
Network estimate				−0.02	[−0.43; 0.40]
95% prediction interval				−0.02	[−0.74; 0.71]
**NMES vs Standard care**					
Direct estimate	2	1.00	0.0%	0.20	[−0.41; 0.81]
Indirect estimate					
Network estimate				0.20	[−0.41; 0.81]
95% prediction interval				0.20	[−0.73; 1.13]
**BFR exercise vs Standard care**					
Direct estimate	2	1.00	77.4%	0.32	[−0.20; 0.84]
Indirect estimate					
Network estimate				0.32	[−0.20; 0.84]
95% prediction interval				0.32	[−0.51; 1.15]
**Upper−body strength vs Standard care**					
Direct estimate	0	0			
Indirect estimate				0.37	[−0.76; 1.50]
Network estimate				0.37	[−0.76; 1.50]
95% prediction interval				0.37	[−1.18; 1.93]
**Lower−extremity strength vs Standard care**					
Direct estimate	2	1.00	0.0%	0.46	[0.00; 0.92]
Indirect estimate					
Network estimate				0.46	[0.00; 0.92]
95% prediction interval				0.46	[−0.31; 1.23]
**Multidomain exercise vs Standard care**					
Direct estimate	2	1.00	46.2%	0.50	[−0.01; 1.00]
Indirect estimate					
Network estimate				0.50	[−0.01; 1.00]
95% prediction interval				0.50	[−0.32; 1.31]

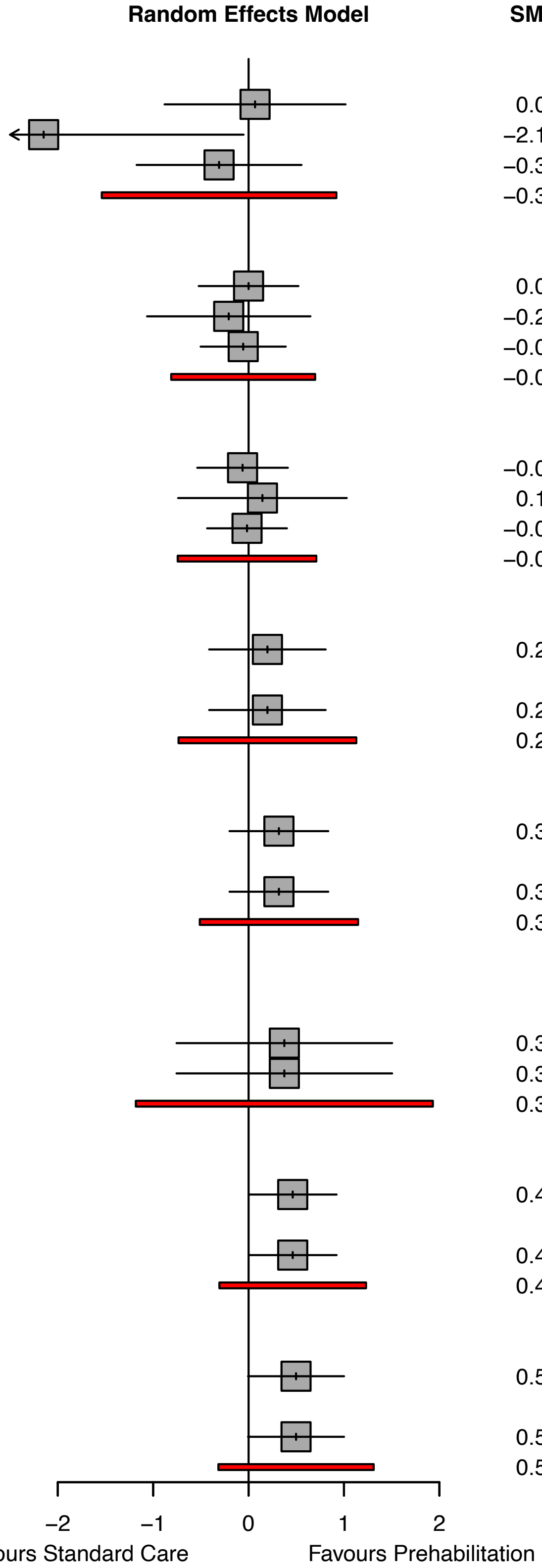

Local inconsistency assessment for physical function at 3 months after TKA. Direct, indirect, and network estimates versus standard care are presented as standardized mean differences (SMDs) with 95% confidence intervals (CIs). Agreement between the direct and indirect estimates indicates little evidence of local inconsistency. Red horizontal lines indicate the 95% prediction intervals. Positive SMDs favor the prehabilitation modality, whereas negative SMDs favor standard care. BFR, blood flow restriction; NMES, neuromuscular electrical stimulation.

S13B. Local Inconsistency by Treatment Comparison (3 Months)

Quadriceps Strength

Comparison	Number of Studies	Direct Evidence	I2	SMD	95% CI
**Aerobic exercise vs Standard care**					
Direct estimate	1	0.86		−0.44	[−1.62; 0.75]
Indirect estimate				−0.83	[−3.77; 2.11]
Network estimate				−0.49	[−1.59; 0.61]
95% prediction interval				−0.49	[−2.19; 1.20]
**Education vs Standard care**					
Direct estimate	0	0			
Indirect estimate				−0.04	[−1.38; 1.29]
Network estimate				−0.04	[−1.38; 1.29]
95% prediction interval				−0.04	[−1.98; 1.89]
**Exercise & Education vs Standard care**					
Direct estimate	1	1.00		0.00	[−0.88; 0.88]
Indirect estimate					
Network estimate				0.00	[−0.88; 0.88]
95% prediction interval				0.00	[−1.48; 1.48]
**Multidomain exercise vs Standard care**					
Direct estimate	3	1.00	63.7%	0.33	[−0.20; 0.86]
Indirect estimate					
Network estimate				0.33	[−0.20; 0.86]
95% prediction interval				0.33	[−0.86; 1.52]
**Upper−body strength vs Standard care**					
Direct estimate	0	0			
Indirect estimate				0.34	[−1.05; 1.73]
Network estimate				0.34	[−1.05; 1.73]
95% prediction interval				0.34	[−1.66; 2.33]
**Lower−extremity strength vs Standard care**					
Direct estimate	3	1.00	61.6%	0.41	[−0.20; 1.01]
Indirect estimate					
Network estimate				0.41	[−0.20; 1.01]
95% prediction interval				0.41	[−0.84; 1.65]
**NMES vs Standard care**					
Direct estimate	2	1.00	82.0%	0.46	[−0.37; 1.29]
Indirect estimate					
Network estimate				0.46	[−0.37; 1.29]
95% prediction interval				0.46	[−0.97; 1.90]
**BFR exercise vs Standard care**					
Direct estimate	2	1.00	0.0%	0.68	[−0.06; 1.42]
Indirect estimate					
Network estimate				0.68	[−0.06; 1.42]
95% prediction interval				0.68	[−0.67; 2.03]

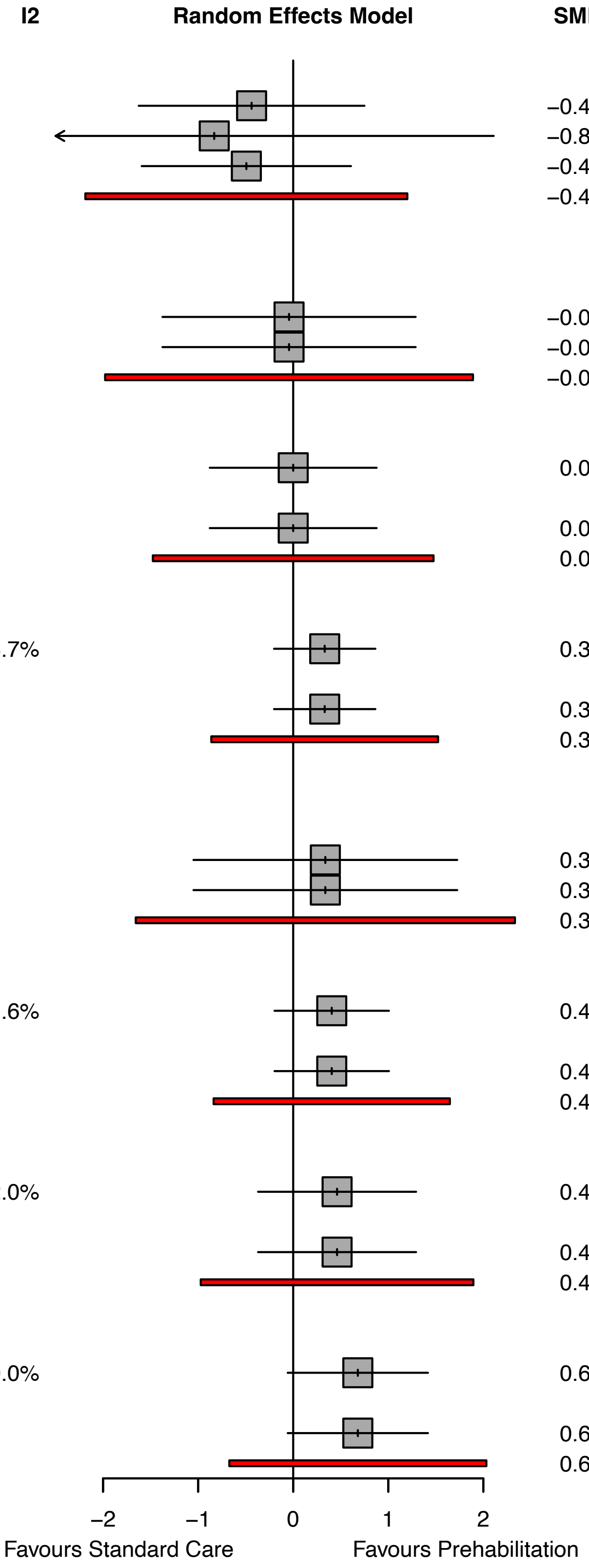

Local inconsistency assessment for quadriceps strength at 3 months after TKA. Direct, indirect, and network estimates versus standard care are presented as standardized mean differences (SMDs) with 95% confidence intervals (CIs). Agreement between the direct and indirect estimates indicates little evidence of local inconsistency. Red horizontal lines indicate the 95% prediction intervals. Positive SMDs favor the prehabilitation modality, whereas negative SMDs favor standard care. BFR, blood flow restriction; NMES, neuromuscular electrical stimulation.

S13C. Local Inconsistency by Treatment Comparison (3 Months)

Pain

Comparison	Number of Studies	Direct Evidence	I2	SMD	95% CI
**Aerobic exercise vs Standard care**					
Direct estimate	1	0.85		0.15	[−1.15; 1.46]
Indirect estimate				−2.84	[−5.98; 0.30]
Network estimate				−0.29	[−1.49; 0.92]
95% prediction interval				−0.29	[−2.32; 1.74]
**Education vs Standard care**					
Direct estimate	1	0.70		−0.29	[−1.31; 0.72]
Indirect estimate				0.21	[−1.33; 1.76]
Network estimate				−0.14	[−0.99; 0.71]
95% prediction interval				−0.14	[−1.83; 1.55]
**NMES vs Standard care**					
Direct estimate	2	1.00	0.0%	−0.13	[−1.03; 0.77]
Indirect estimate					
Network estimate				−0.13	[−1.03; 0.77]
95% prediction interval				−0.13	[−1.86; 1.61]
**Exercise & Education vs Standard care**					
Direct estimate	1	0.68		0.06	[−0.97; 1.10]
Indirect estimate				−0.44	[−1.97; 1.09]
Network estimate				−0.10	[−0.95; 0.76]
95% prediction interval				−0.10	[−1.79; 1.60]
**BFR exercise vs Standard care**					
Direct estimate	2	1.00	87.5%	0.26	[−0.58; 1.10]
Indirect estimate					
Network estimate				0.26	[−0.58; 1.10]
95% prediction interval				0.26	[−1.42; 1.94]
**Multidomain exercise vs Standard care**					
Direct estimate	2	1.00	0.0%	0.40	[−0.44; 1.24]
Indirect estimate					
Network estimate				0.40	[−0.44; 1.24]
95% prediction interval				0.40	[−1.28; 2.08]
**Lower−extremity strength vs Standard care**					
Direct estimate	2	1.00	86.2%	0.42	[−0.36; 1.21]
Indirect estimate					
Network estimate				0.42	[−0.36; 1.21]
95% prediction interval				0.42	[−1.21; 2.06]
**Upper−body strength vs Standard care**					
Direct estimate	0	0			
Indirect estimate				0.63	[−0.95; 2.21]
Network estimate				0.63	[−0.95; 2.21]
95% prediction interval				0.63	[−1.80; 3.05]

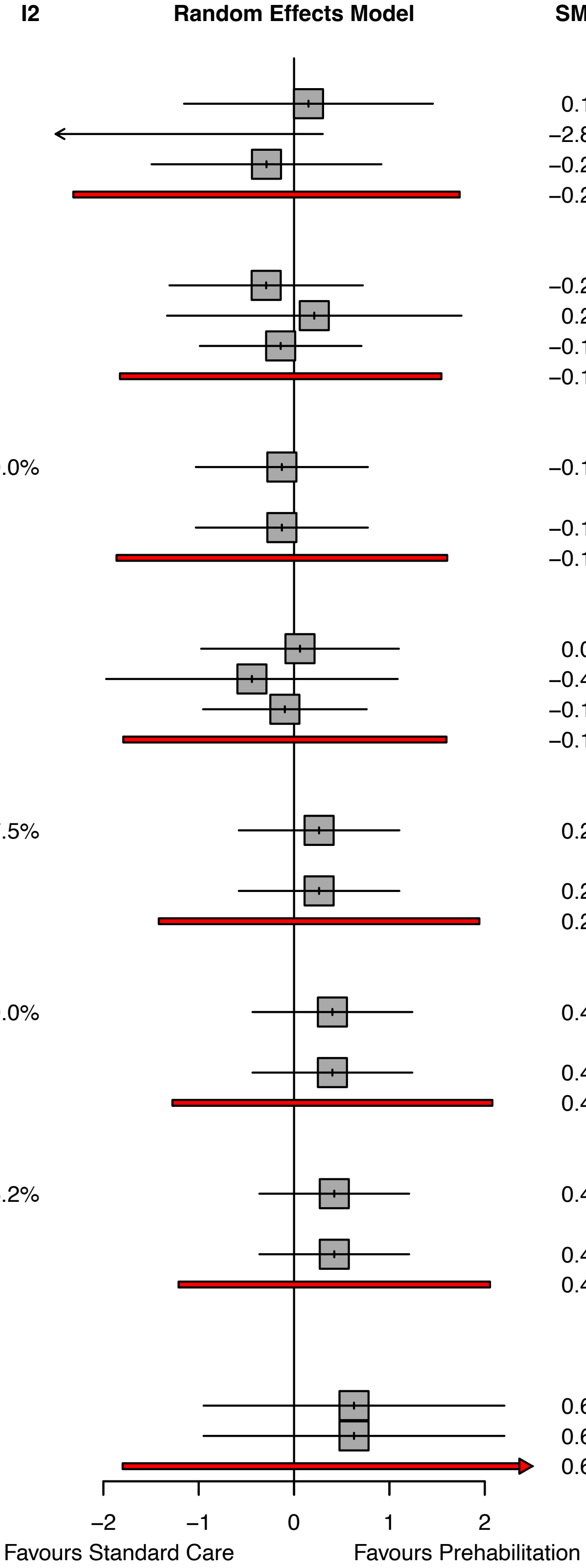

Local inconsistency assessment for pain at 3 months after TKA. Direct, indirect, and network estimates versus standard care are presented as standardized mean differences (SMDs) with 95% confidence intervals (CIs). Agreement between the direct and indirect estimates indicates little evidence of local inconsistency. Red horizontal lines indicate the 95% prediction intervals. Positive SMDs favor the prehabilitation modality, whereas negative SMDs favor standard care. BFR, blood flow restriction; NMES, neuromuscular electrical stimulation.

S13D. Local Inconsistency by Treatment Comparison (3 Months)

Health-Related Quality of Life

Comparison	Number of Studies	Direct Evidence	I2	SMD	95% CI
**Aerobic exercise vs Standard care**					
Direct estimate	1	0.83		−0.17	[−1.05; 0.71]
Indirect estimate				−1.72	[−3.66; 0.22]
Network estimate				−0.43	[−1.23; 0.37]
95% prediction interval				−0.43	[−1.73; 0.87]
**Upper−body strength vs Standard care**					
Direct estimate	0	0			
Indirect estimate				−0.41	[−1.55; 0.73]
Network estimate				−0.41	[−1.55; 0.73]
95% prediction interval				−0.41	[−2.26; 1.44]
**Education vs Standard care**					
Direct estimate	1	0.85		0.05	[−0.26; 0.35]
Indirect estimate				−0.28	[−1.01; 0.44]
Network estimate				−0.00	[−0.29; 0.28]
95% prediction interval				−0.00	[−0.46; 0.45]
**Exercise & Education vs Standard care**					
Direct estimate	1	0.77		0.00	[−0.38; 0.38]
Indirect estimate				0.33	[−0.36; 1.02]
Network estimate				0.08	[−0.25; 0.41]
95% prediction interval				0.08	[−0.46; 0.61]
**NMES vs Standard care**					
Direct estimate	2	1.00	0.0%	0.16	[−0.38; 0.70]
Indirect estimate					
Network estimate				0.16	[−0.38; 0.70]
95% prediction interval				0.16	[−0.72; 1.04]
**Lower−extremity strength vs Standard care**					
Direct estimate	1	1.00		0.24	[−0.32; 0.80]
Indirect estimate					
Network estimate				0.24	[−0.32; 0.80]
95% prediction interval				0.24	[−0.68; 1.15]
**Multidomain exercise vs Standard care**					
Direct estimate	1	1.00		0.24	[−0.19; 0.68]
Indirect estimate					
Network estimate				0.24	[−0.19; 0.68]
95% prediction interval				0.24	[−0.47; 0.95]
**BFR exercise vs Standard care**					
Direct estimate	2	1.00	51.0%	0.38	[−0.05; 0.82]
Indirect estimate					
Network estimate				0.38	[−0.05; 0.82]
95% prediction interval				0.38	[−0.31; 1.08]

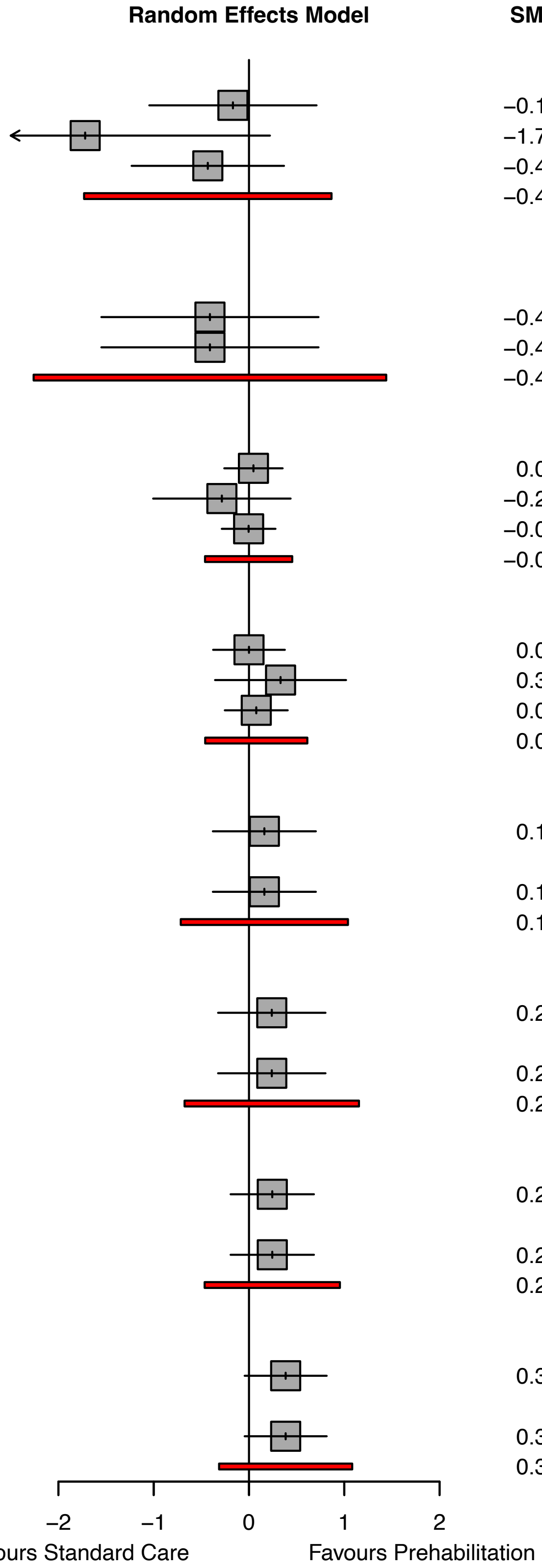

Local inconsistency assessment for health-related quality of life at 3 months after TKA. Direct, indirect, and network estimates versus standard care are presented as standardized mean differences (SMDs) with 95% confidence intervals (CIs). Agreement between the direct and indirect estimates indicates little evidence of local inconsistency. Red horizontal lines indicate the 95% prediction intervals. Positive SMDs favor the prehabilitation modality, whereas negative SMDs favor standard care. BFR, blood flow restriction; NMES, neuromuscular electrical stimulation.

S14A. Sensitivity Analysis: Excluding High Risk of Bias Studies (3 Months)

Physical Function

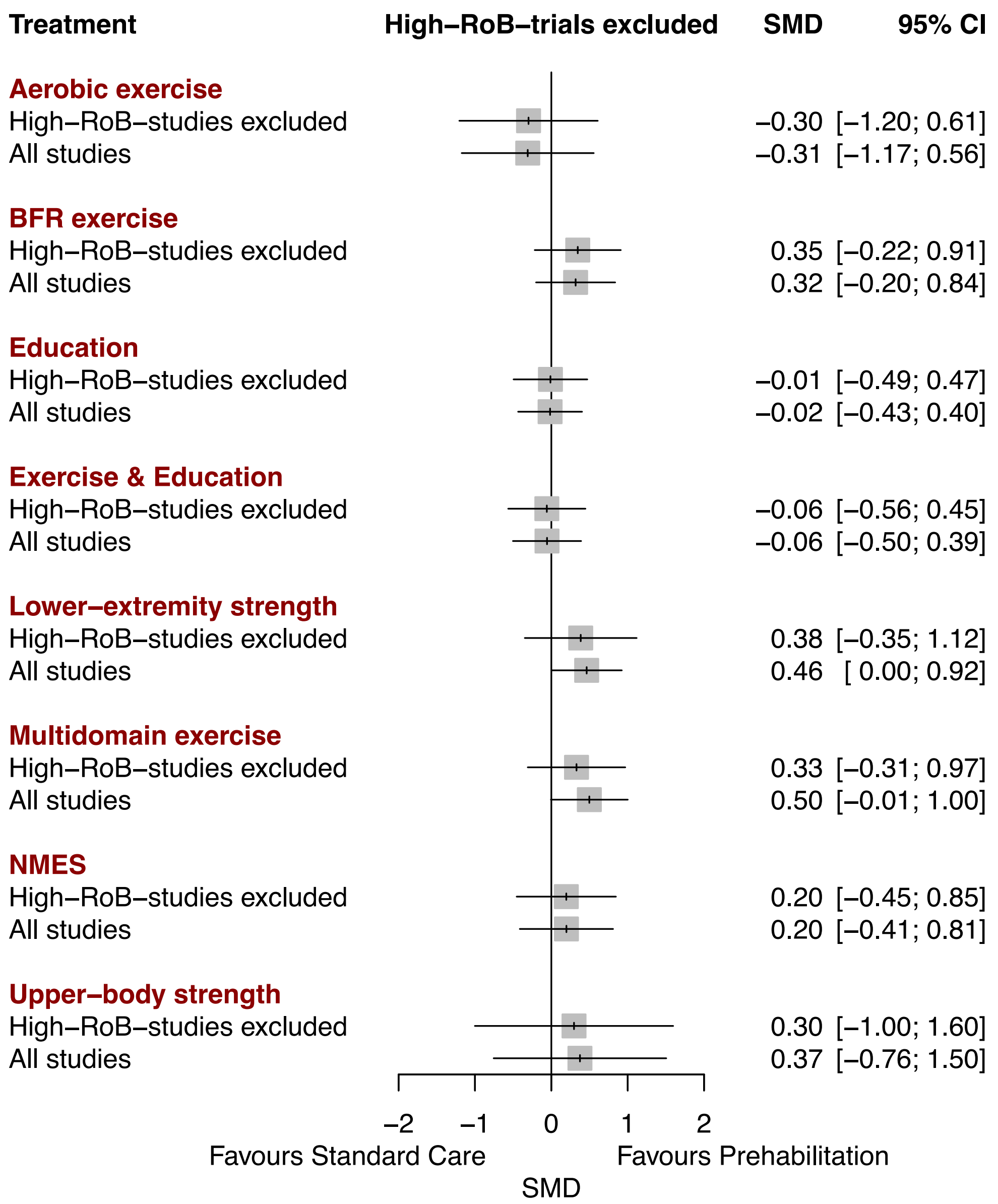

Sensitivity analysis for physical function after excluding studies at high risk of bias (Brown et al. 2012 and Tungtrongjit et al. 2012). Results are presented as standardized mean differences (SMDs) with 95% confidence intervals (CIs). Positive SMDs favor the prehabilitation modality, whereas negative SMDs favor standard care. BFR, blood flow restriction; NMES, neuromuscular electrical stimulation.

S14B. Sensitivity Analysis: Excluding High Risk of Bias Studies (3 Months)

Quadriceps Strength

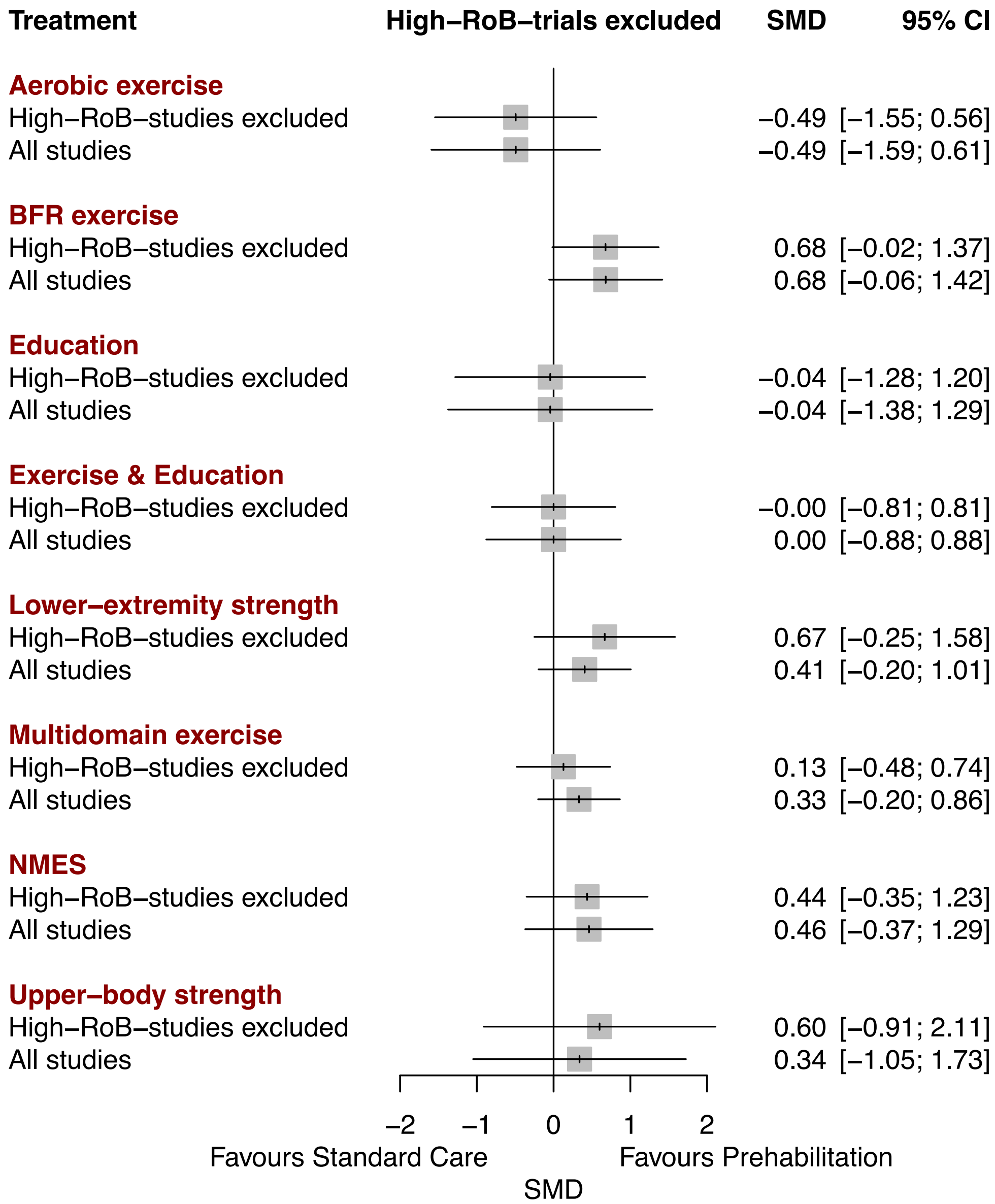

Sensitivity analysis for quadriceps strength after excluding studies at high risk of bias (Sun et al. 2023, Van Leeuwen et al. 2014, Tungtrongjit et al. 2012). Results are presented as standardized mean differences (SMDs) with 95% confidence intervals (CIs). Positive SMDs favor the prehabilitation modality, whereas negative SMDs favor standard care. BFR, blood flow restriction; NMES, neuromuscular electrical stimulation.

S14C. Sensitivity Analysis: Excluding High Risk of Bias Studies (3 Months)

Pain

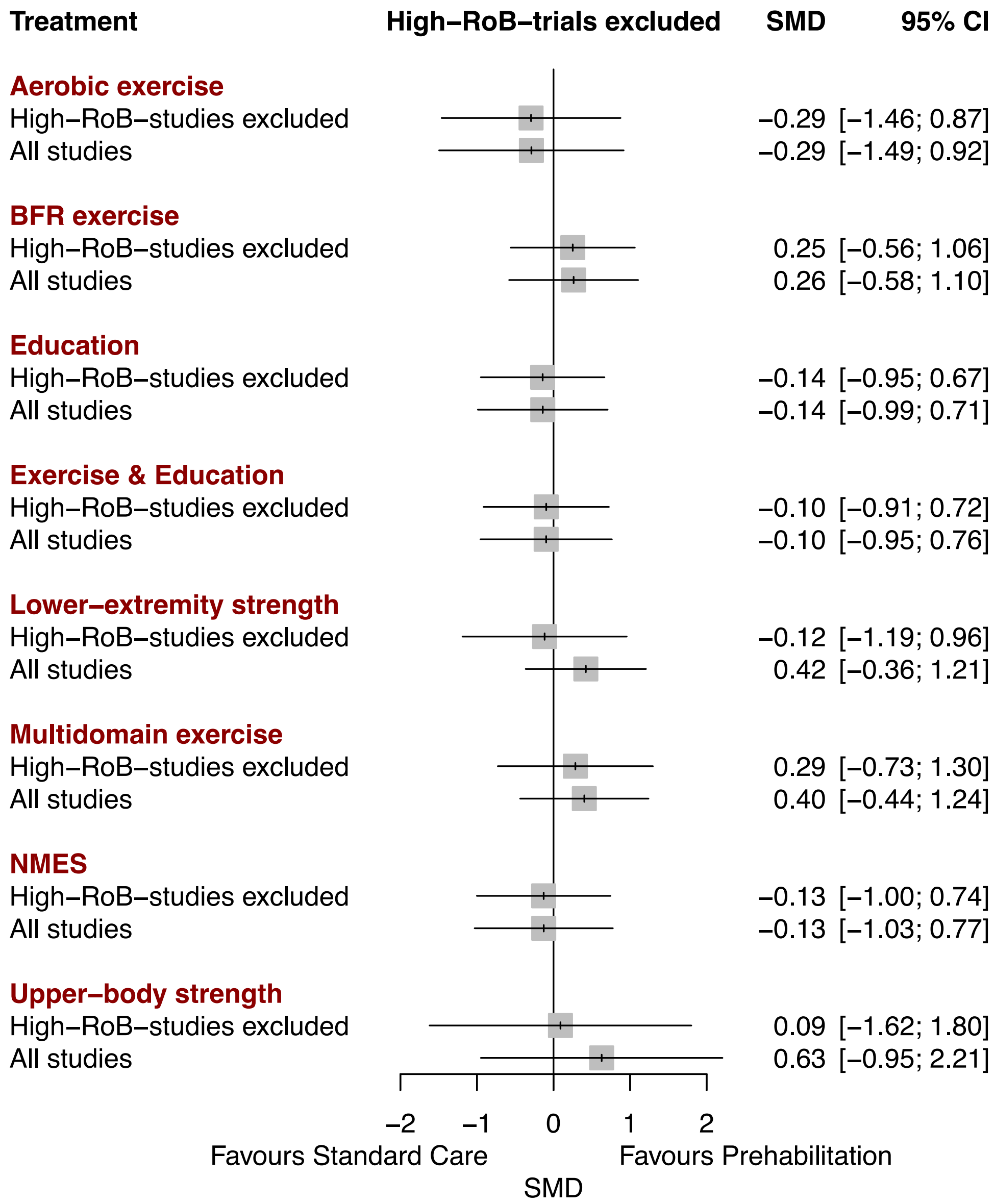

Sensitivity analysis for pain after excluding studies at high risk of bias (Brown et al. 2012 and Tungtrongjit et al. 2012). Results are presented as standardized mean differences (SMDs) with 95% confidence intervals (CIs). Positive SMDs favor the prehabilitation modality, whereas negative SMDs favor standard care. BFR, blood flow restriction; NMES, neuromuscular electrical stimulation.

S15A. Sensitivity Analysis: Excluding Change Score Studies (3 Months)

Physical Function

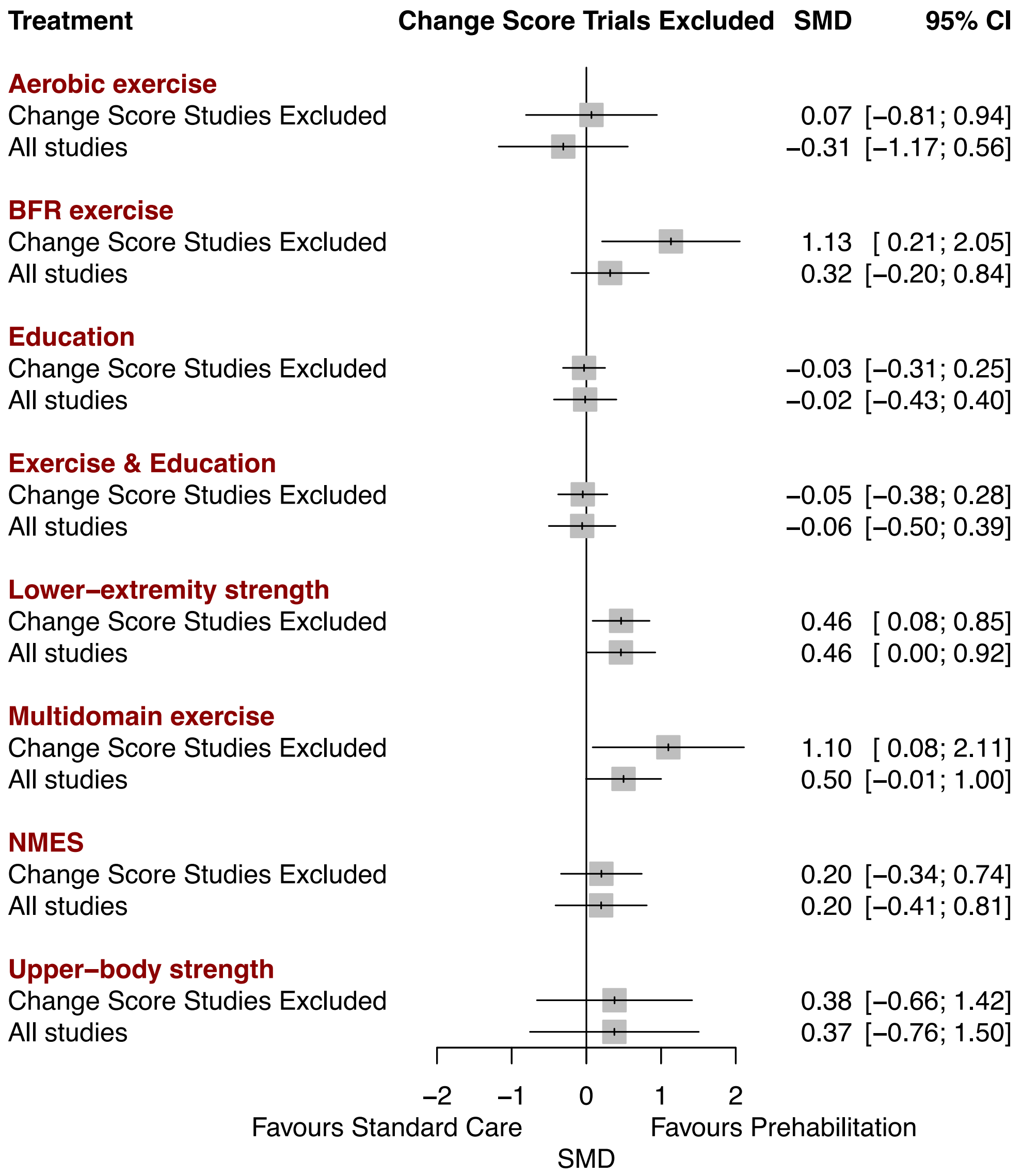

Sensitivity analysis for physical function excluding studies reporting change scores from baseline to 3 months after TKA (Jorgensen et al. 2024; Villadsen et al. 2014). Results are presented as standardized mean differences (SMDs) with 95% confidence intervals (CIs). Positive SMDs favor the prehabilitation modality, whereas negative SMDs favor standard care. BFR, blood flow restriction; NMES, neuromuscular electrical stimulation.

S15B. Sensitivity Analysis: Excluding Change Score Studies (3 Months)

Quadriceps Strength

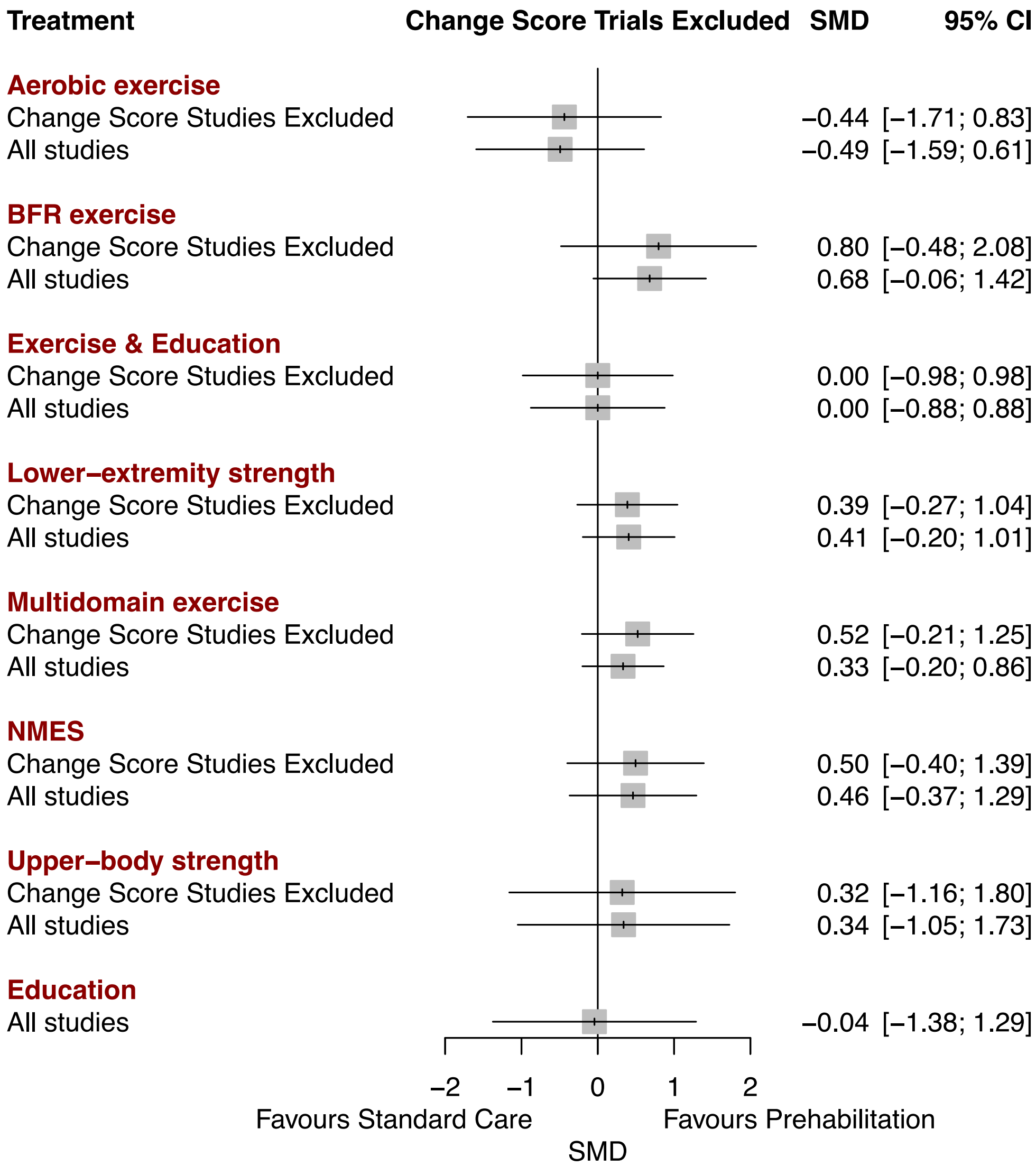

Sensitivity analysis for quadriceps strength excluding studies reporting change scores from baseline to 3 months after TKA (Huber et al. 2015; Jorgensen et al. 2024; Villadsen et al. 2014). Results are presented as standardized mean differences (SMDs) with 95% confidence intervals (CIs). Positive SMDs favor the prehabilitation modality, whereas negative SMDs favor standard care. BFR, blood flow restriction; NMES, neuromuscular electrical stimulation.

S15C. Sensitivity Analysis: Excluding Change Score Studies (3 Months)

Pain

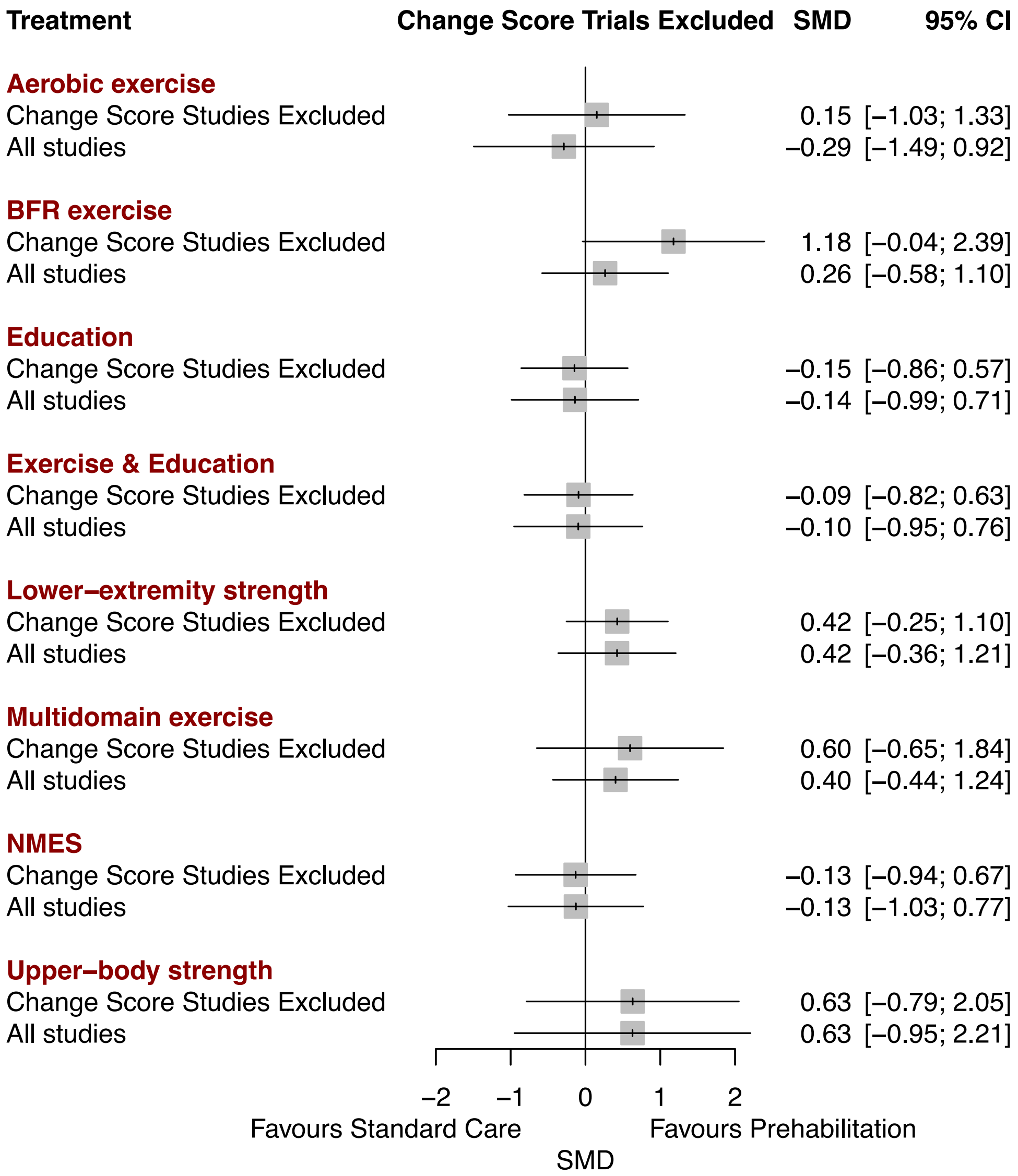

Sensitivity analysis for pain excluding studies reporting change scores from baseline to 3 months after TKA (Jorgensen et al. 2024; Villadsen et al. 2014). Results are presented as standardized mean differences (SMDs) with 95% confidence intervals (CIs). Positive SMDs favor the prehabilitation modality, whereas negative SMDs favor standard care. BFR, blood flow restriction; NMES, neuromuscular electrical stimulation.

S15D. Sensitivity Analysis: Excluding Change Score Studies (3 Months)

Health-Related Quality of Life

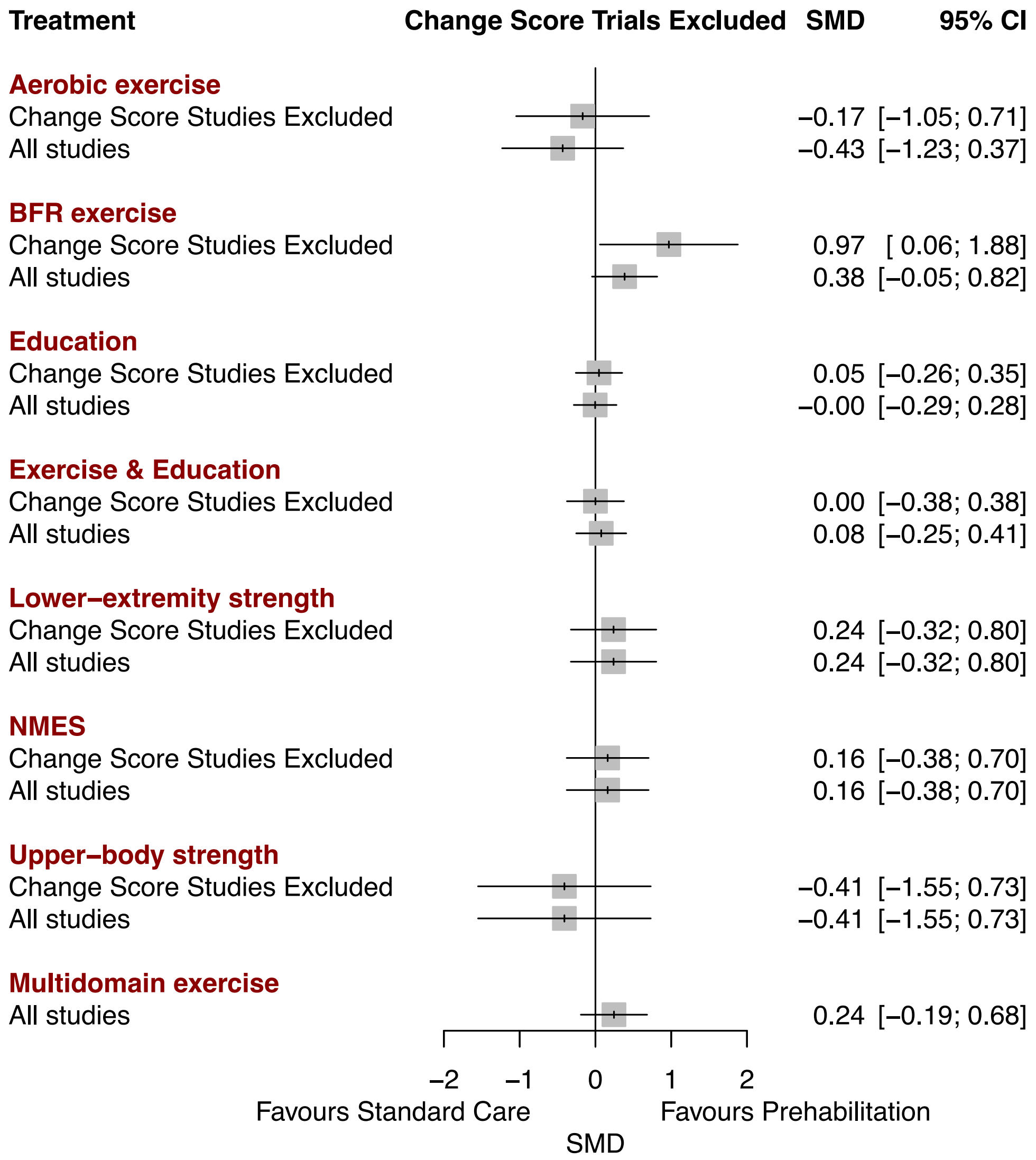

Sensitivity analysis for health-related quality of life excluding studies reporting change scores from baseline to 3 months after TKA (Huber et al. 2015; Jorgensen et al. 2024; Villadsen et al. 2014). Results are presented as standardized mean differences (SMDs) with 95% confidence intervals (CIs). Positive SMDs favor the prehabilitation modality, whereas negative SMDs favor standard care. BFR, blood flow restriction; NMES, neuromuscular electrical stimulation.

S16A. Sensitivity Analysis: Including Model-Based CIs (3 Months)

Physical Function

Treatment	SMD	95% CI
**Aerobic exercise**		
Primary analysis	−0.31	[−1.17; 0.56]
Calatayud included	−0.26	[−1.39; 0.86]
**BFR exercise**		
Primary analysis	0.32	[−0.20; 0.84]
Calatayud included	0.44	[−0.33; 1.21]
**Education**		
Primary analysis	−0.02	[−0.43; 0.40]
Calatayud included	−0.00	[−0.76; 0.76]
**Exercise & Education**		
Primary analysis	−0.06	[−0.50; 0.39]
Calatayud included	−0.06	[−0.84; 0.71]
**Lower−extremity strength**		
Primary analysis	0.46	[0.00; 0.92]
Calatayud included	0.85	[0.25; 1.45]
**Multidomain exercise**		
Primary analysis	0.50	[−0.01; 1.00]
Calatayud included	0.59	[−0.18; 1.37]
**NMES**		
Primary analysis	0.20	[−0.41; 0.81]
Calatayud included	0.19	[−0.65; 1.03]
**Upper−body strength**		
Primary analysis	0.37	[−0.76; 1.50]
Calatayud included	0.76	[−0.66; 2.18]

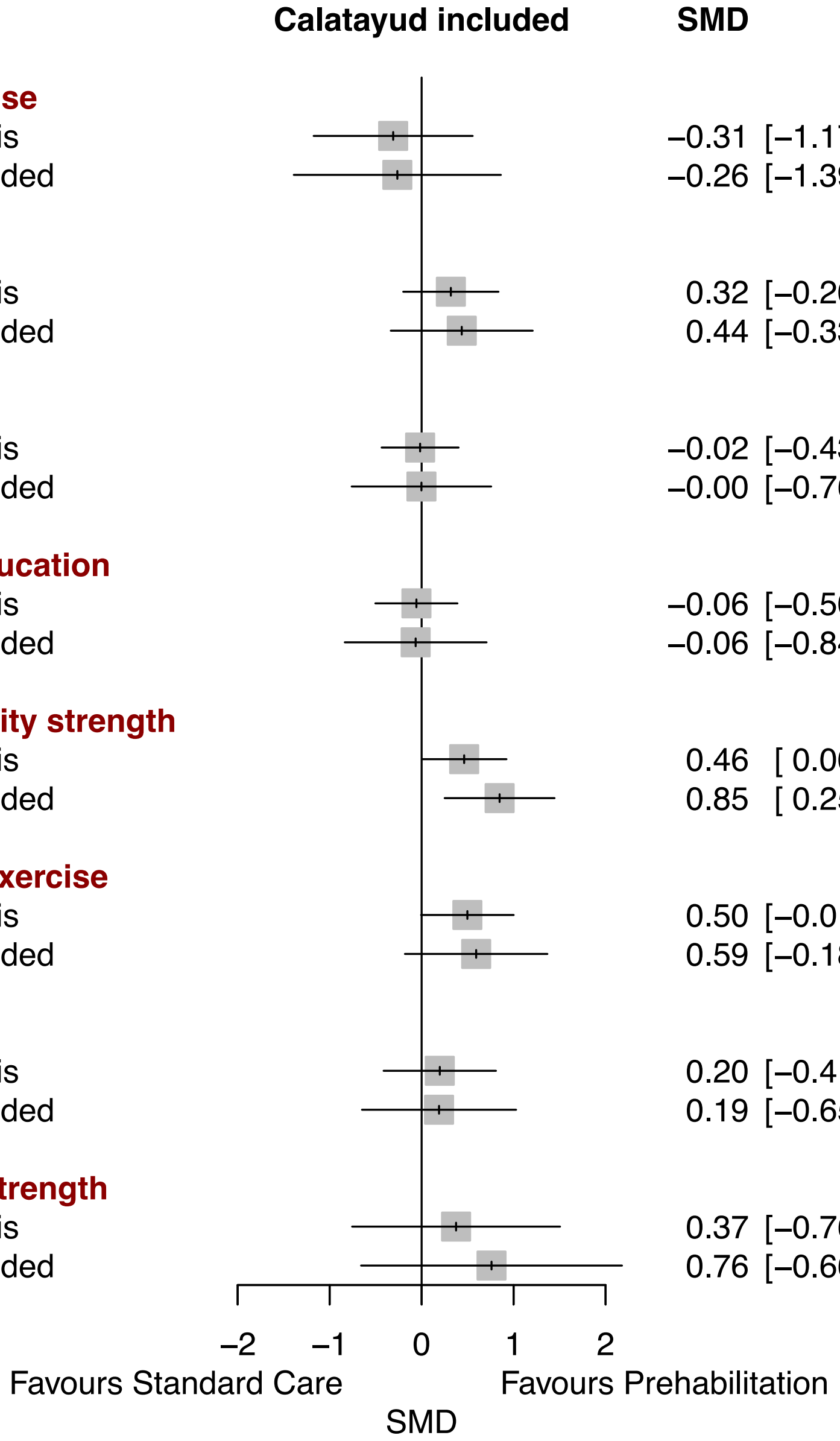

Sensitivity analysis for physical function including the previously excluded study by Calatayud et al. (2017), using SDs derived from model-based confidence intervals (CIs). Results are presented as standardized mean differences (SMDs) with 95% CIs. Positive SMDs favor the prehabilitation modality, whereas negative SMDs favor standard care. BFR, blood flow restriction; NMES, neuromuscular electrical stimulation.

S16B. Sensitivity Analysis: Including Model-Based CIs (3 Months)

Quadriceps Strength

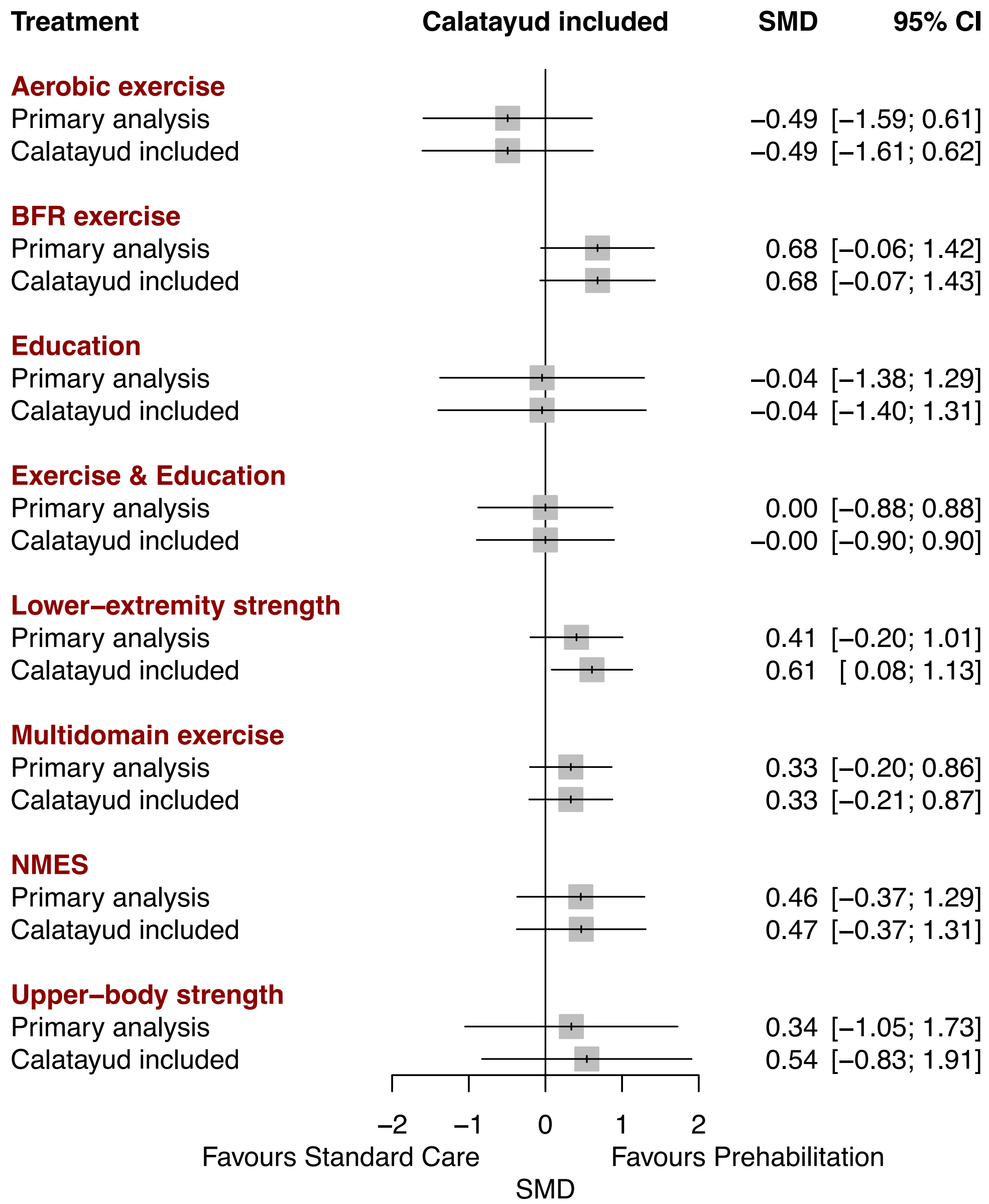

Sensitivity analysis for quadriceps strength including the previously excluded study by Calatayud et al. (2017), using SDs derived from model-based confidence intervals (CIs). Results are presented as standardized mean differences (SMDs) with 95% CIs. Positive SMDs favor the prehabilitation modality, whereas negative SMDs favor standard care. BFR, blood flow restriction; NMES, neuromuscular electrical stimulation.

S16C. Sensitivity Analysis: Including Model-Based CIs (3 Months)

Pain

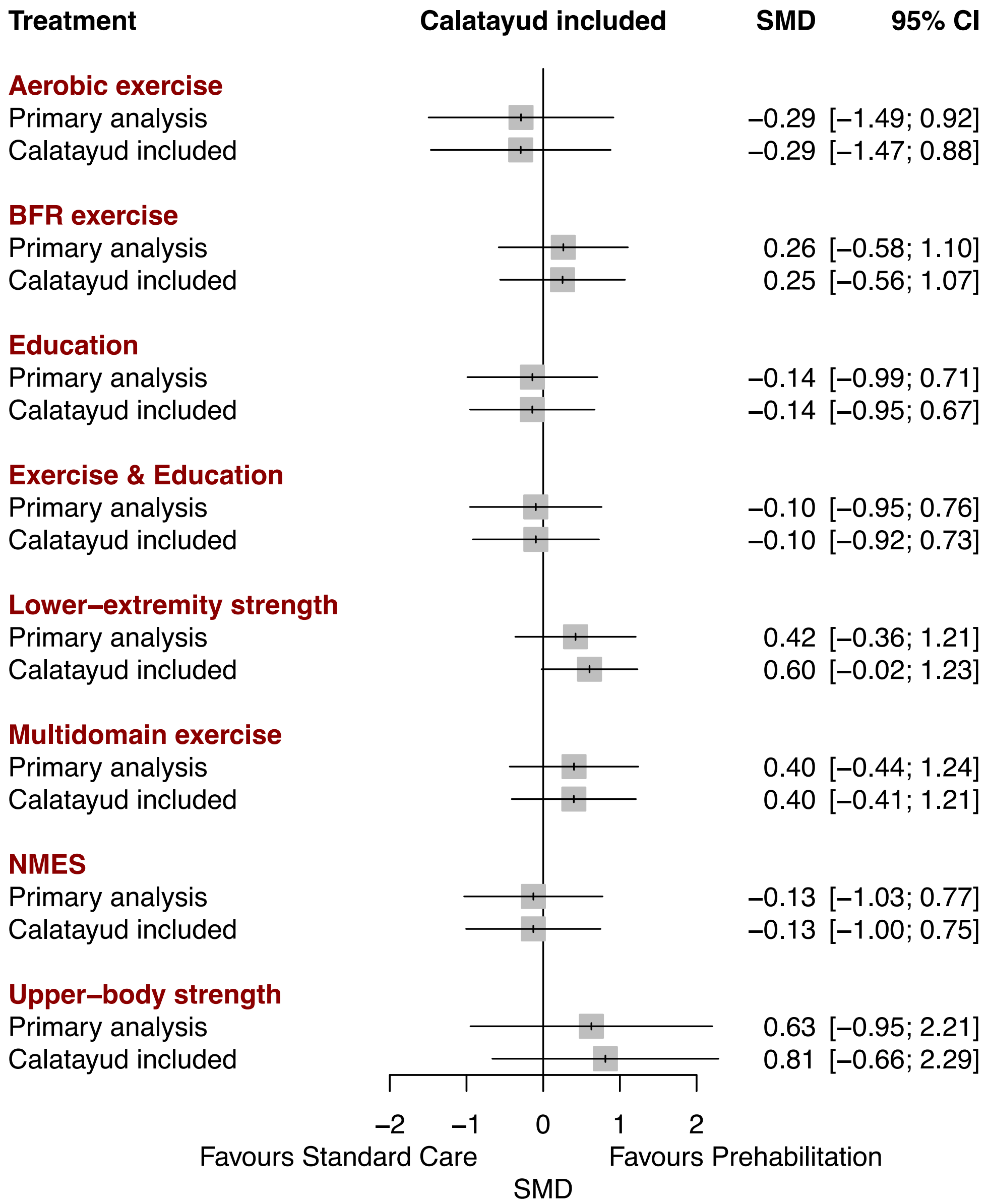

Sensitivity analysis for pain including the previously excluded study by Calatayud et al. (2017), using SDs derived from model-based confidence intervals (CIs). Results are presented as standardized mean differences (SMDs) with 95% CIs. Positive SMDs favor the prehabilitation modality, whereas negative SMDs favor standard care. BFR, blood flow restriction; NMES, neuromuscular electrical stimulation.

S17A. Sensitivity Analysis: Excluding Small Modalities (n<20) (3 Months)

Physical Function

Treatment	SMD	95% CI
**BFR exercise**		
Modalities with N<20 excluded	0.32	[−0.20; 0.84]
All studies	0.32	[−0.20; 0.84]
**Education**		
Modalities with N<20 excluded	−0.02	[−0.43; 0.40]
All studies	−0.02	[−0.43; 0.40]
**Exercise & Education**		
Modalities with N<20 excluded	−0.06	[−0.50; 0.39]
All studies	−0.06	[−0.50; 0.39]
**Lower−extremity strength**		
Modalities with N<20 excluded	0.46	[0.00; 0.92]
All studies	0.46	[0.00; 0.92]
**Multidomain exercise**		
Modalities with N<20 excluded	0.50	[−0.01; 1.00]
All studies	0.50	[−0.01; 1.00]
**NMES**		
Modalities with N<20 excluded	0.20	[−0.41; 0.81]
All studies	0.20	[−0.41; 0.81]
**Aerobic exercise**		
All studies	−0.31	[−1.17; 0.56]
**Upper−body strength**		
All studies	0.37	[−0.76; 1.50]

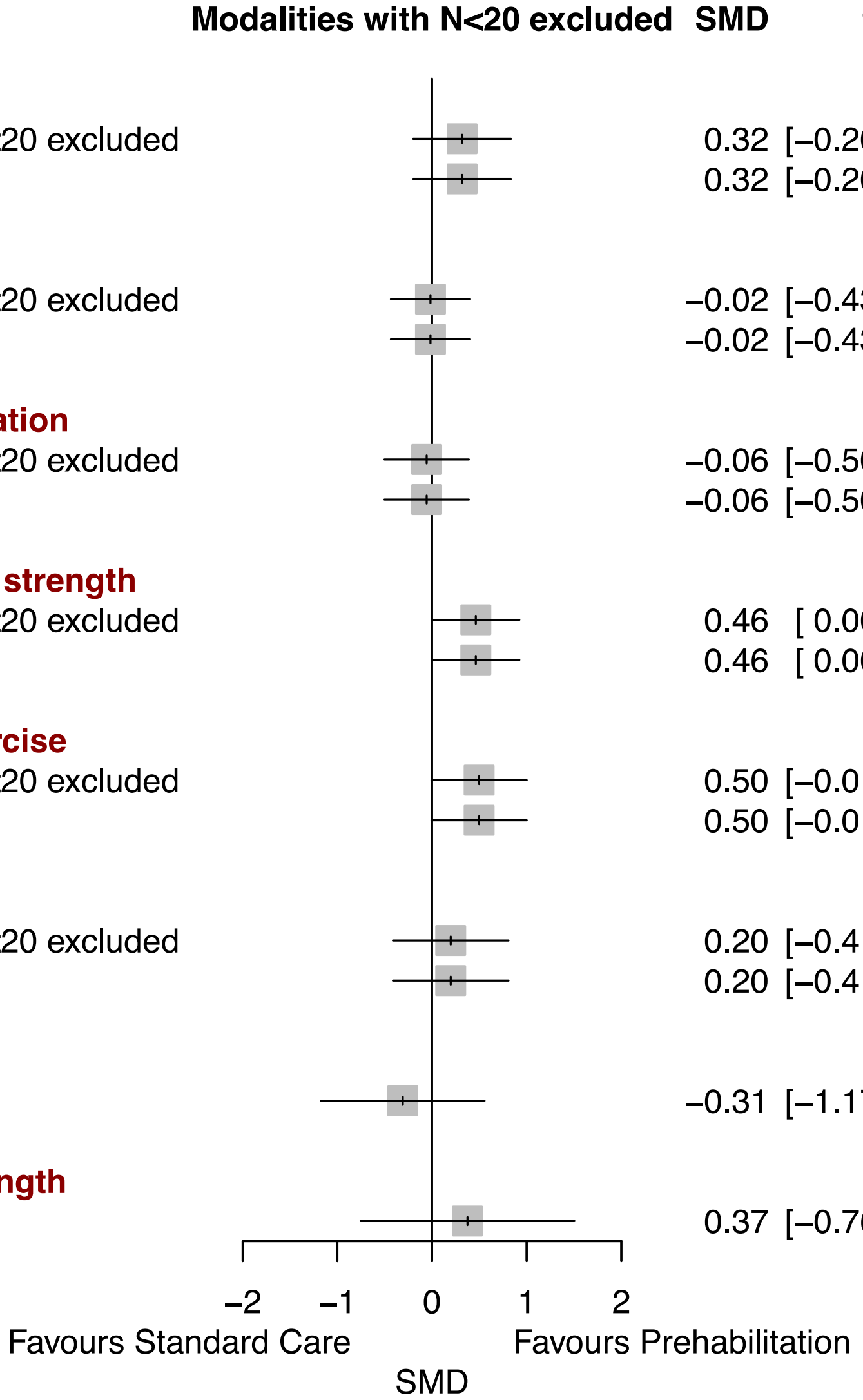

Sensitivity analysis for physical function after excluding modalities with a sample size of n<20 (aerobic exercise and upper-body strength; n = 10 each). Results are presented as standardized mean differences (SMDs) with 95% confidence intervals (CIs). Positive SMDs favor the prehabilitation modality, whereas negative SMDs favor standard care. BFR, blood flow restriction; NMES, neuromuscular electrical stimulation.

S17B. Sensitivity Analysis: Excluding Small Modalities (n<20) (3 Months)

Quadriceps Strength

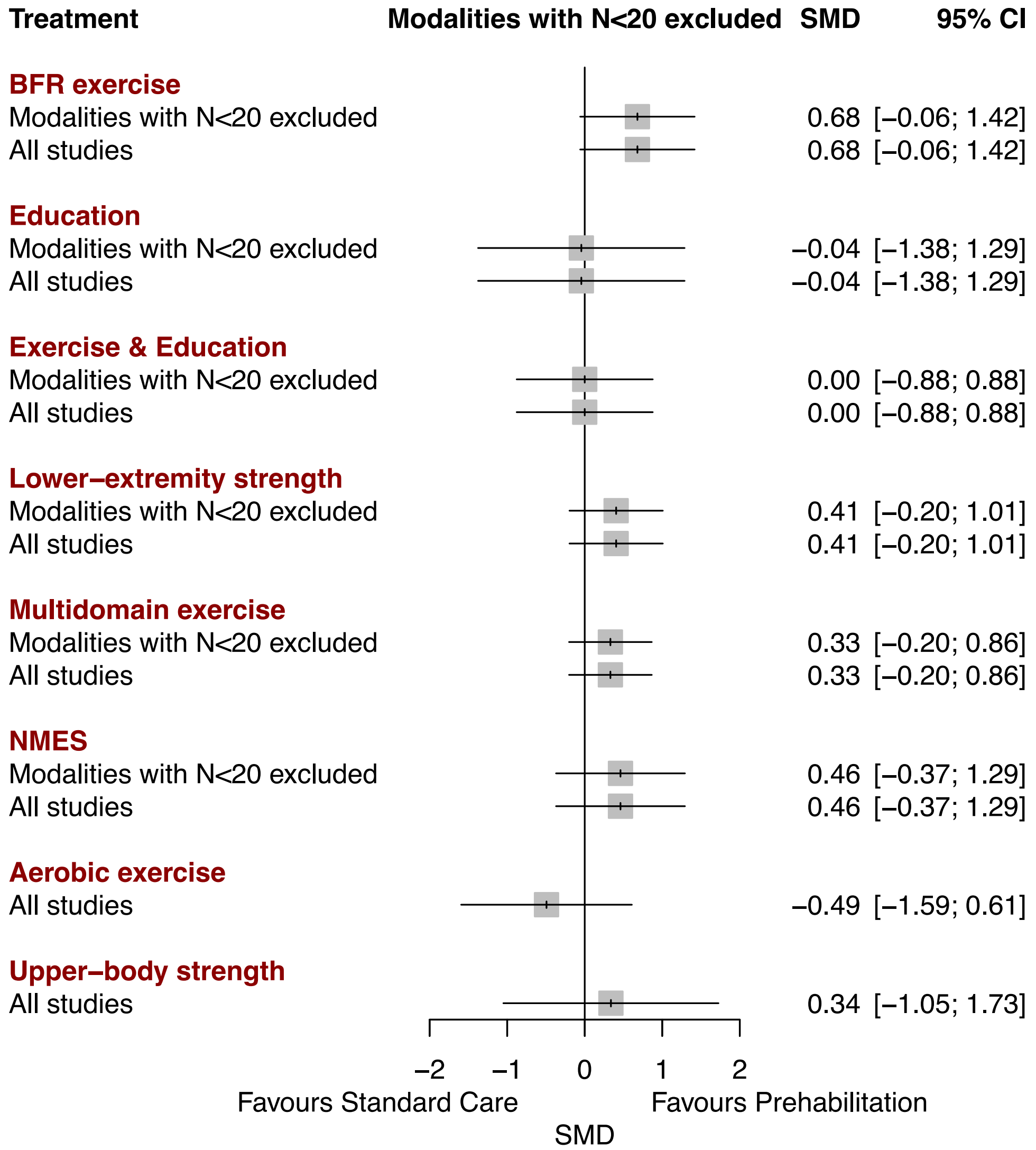

Sensitivity analysis for quadriceps strength after excluding modalities with a sample size of n<20 (aerobic exercise and upper-body strength; n = 10 each). Results are presented as standardized mean differences (SMDs) with 95% confidence intervals (CIs). Positive SMDs favor the prehabilitation modality, whereas negative SMDs favor standard care. BFR, blood flow restriction; NMES, neuromuscular electrical stimulation.

S17C. Sensitivity Analysis: Excluding Small Modalities (n<20) (3 Months)

Pain

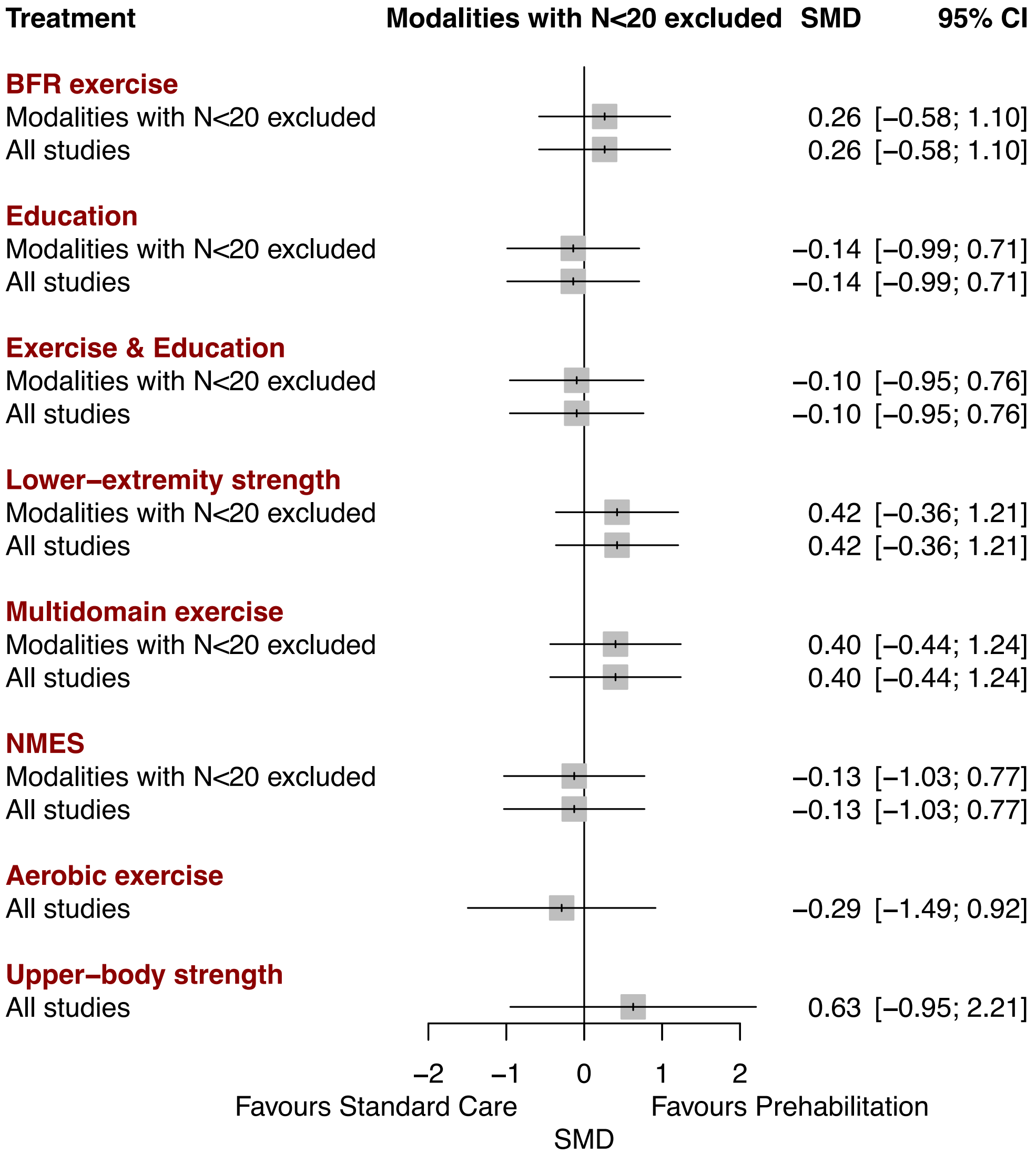

Sensitivity analysis for pain after excluding modalities with a sample size of n<20 (aerobic exercise and upper-body strength; n = 10 each). Results are presented as standardized mean differences (SMDs) with 95% confidence intervals (CIs). Positive SMDs favor the prehabilitation modality, whereas negative SMDs favor standard care. BFR, blood flow restriction; NMES, neuromuscular electrical stimulation.

S17D. Sensitivity Analysis: Excluding Small Modalities (n<20) (3 Months)

Health-related quality of life

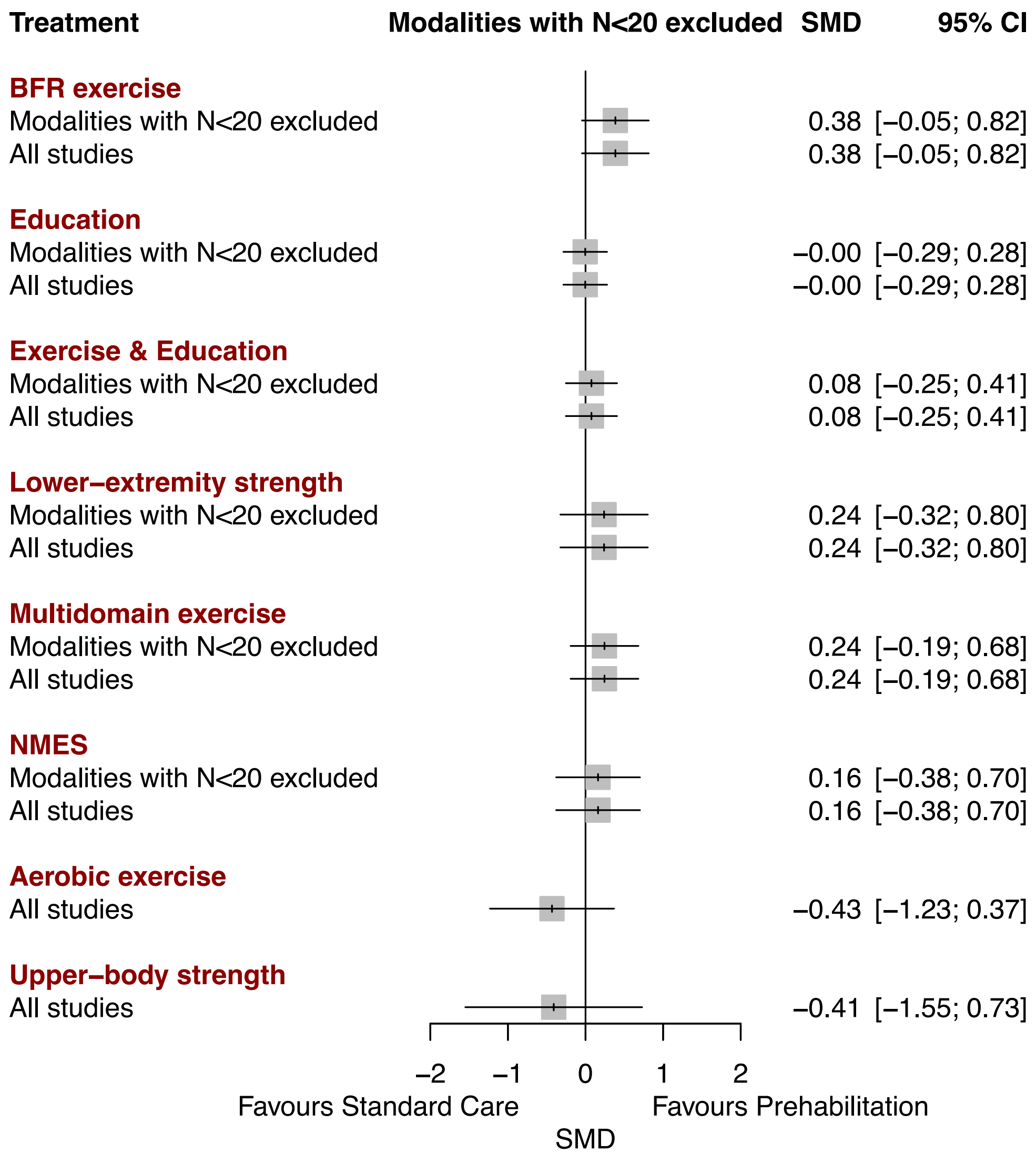

Sensitivity analysis for health-related quality of life after excluding modalities with a sample size of n<20 (aerobic exercise and upper-body strength; n = 10 each). Results are presented as standardized mean differences (SMDs) with 95% confidence intervals (CIs). Positive SMDs favor the prehabilitation modality, whereas negative SMDs favor standard care. BFR, blood flow restriction; NMES, neuromuscular electrical stimulation.

S18. Assessment of the Transitivity Assumption (4–8 Weeks)

Age

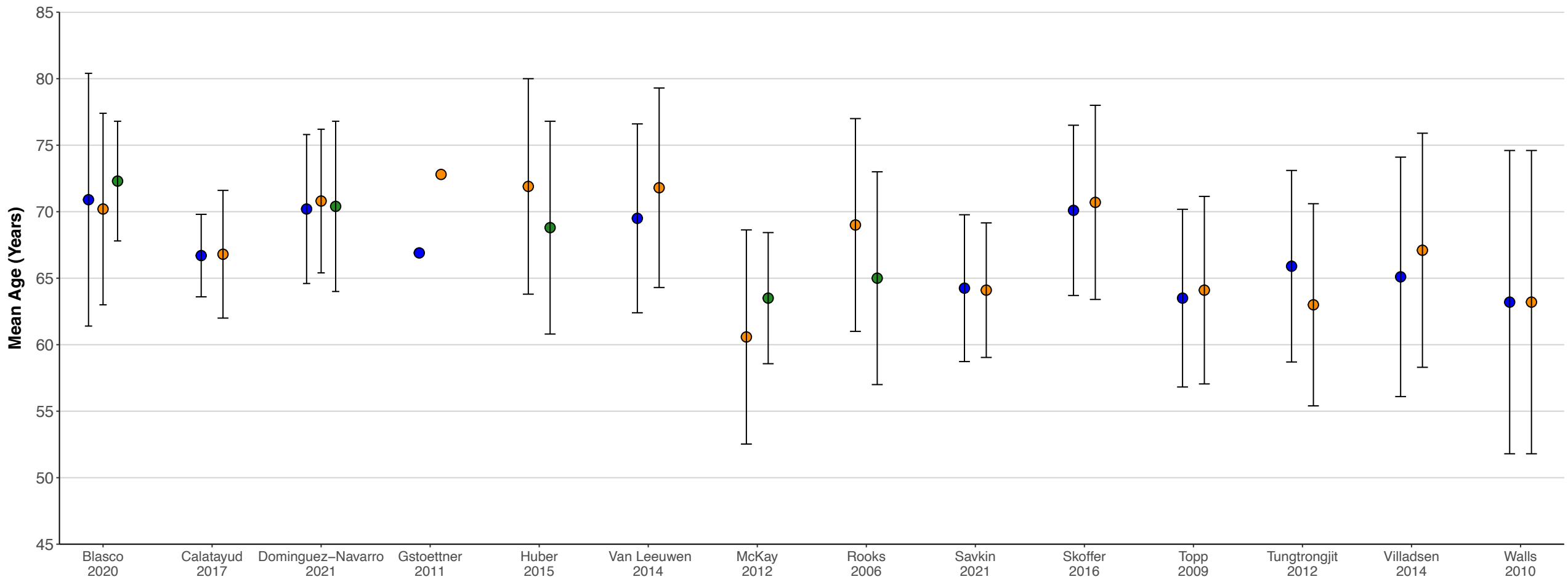

BMI

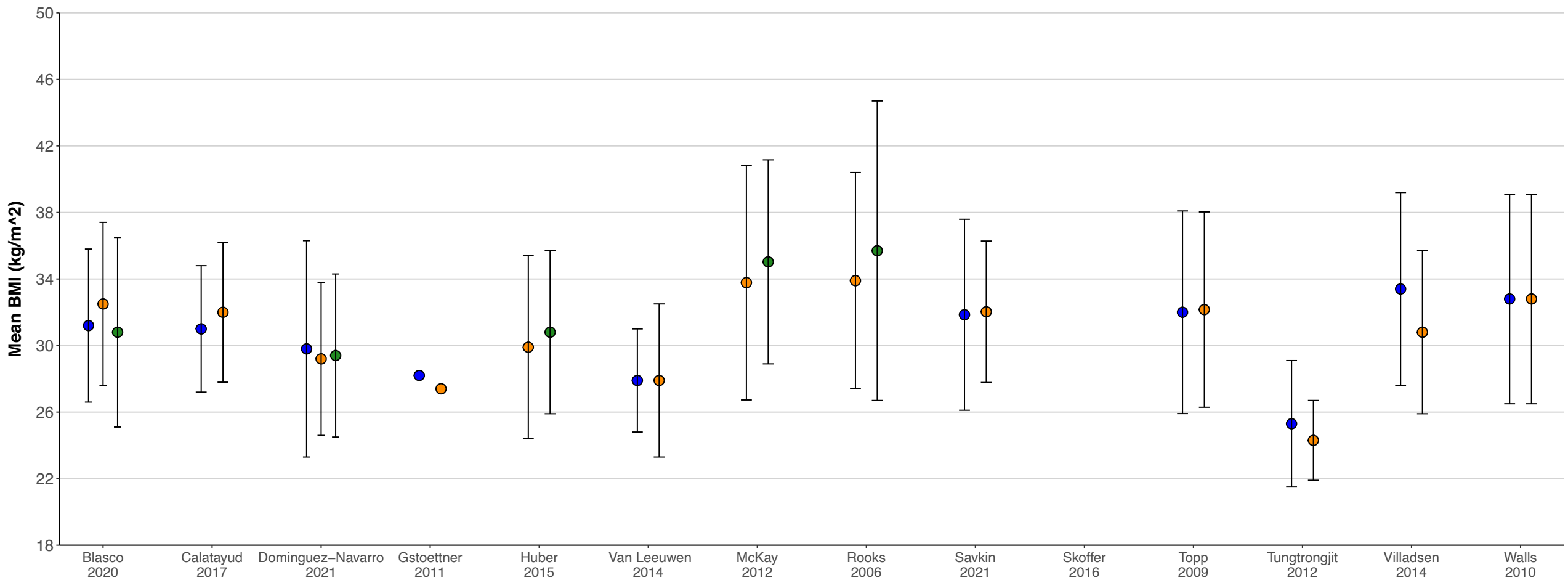

Proportion Females

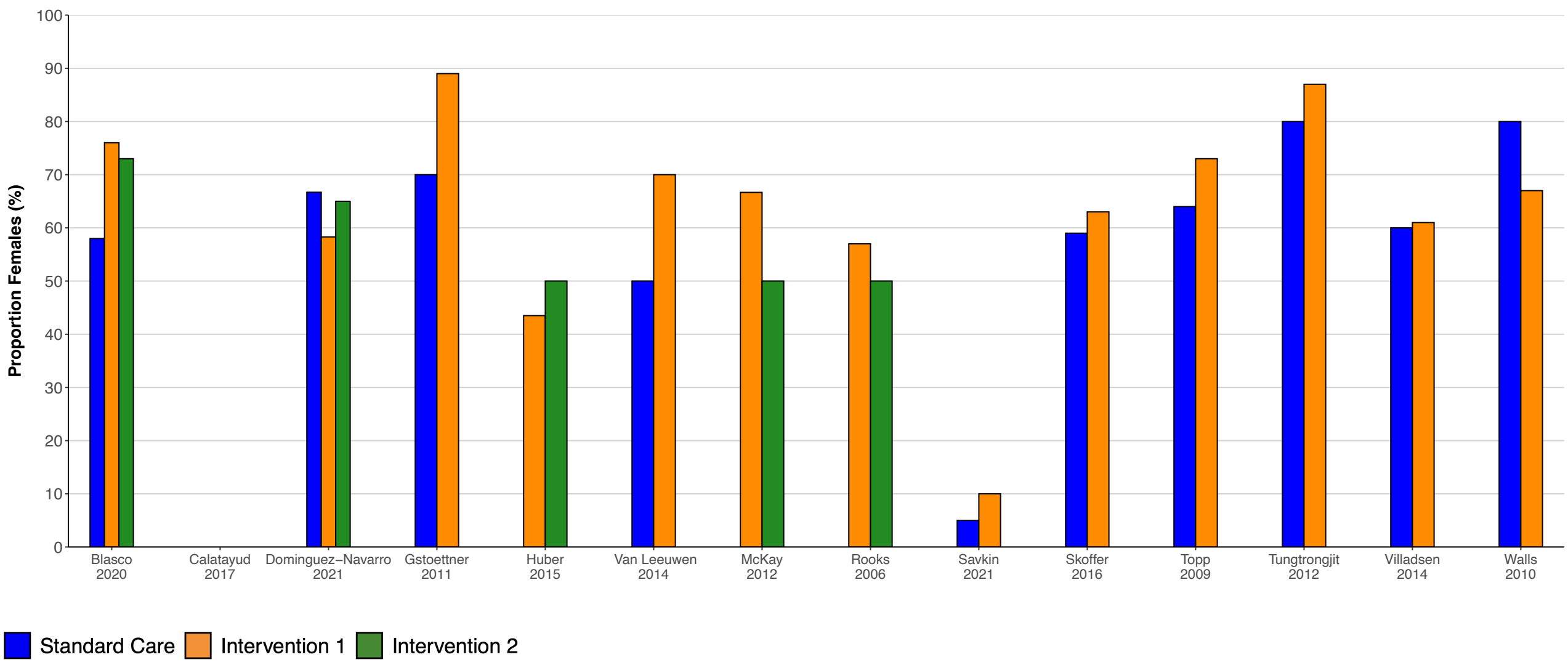

Baseline characteristics across studies included in the 4–8-week network meta-analysis. The distributions of mean age, body mass index (BMI), and the proportion of female participants are presented to evaluate the plausibility of the transitivity assumption.

S19. Network Plot of Prehabilitation Modalities (4–8 Weeks)

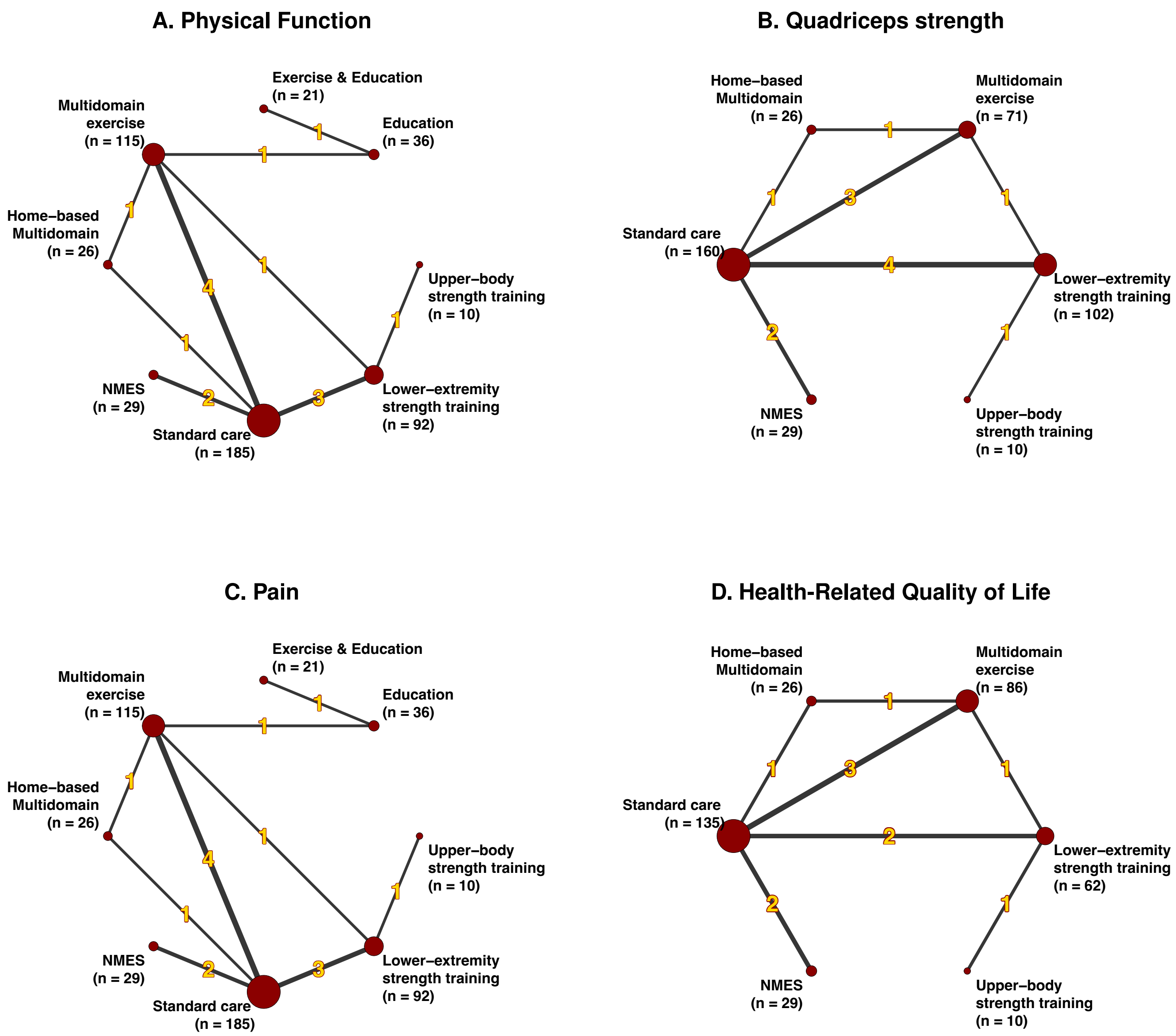

Network plots of prehabilitation modalities 4–8 weeks after TKA. Nodes represent prehabilitation modalities, with node sizes proportional to the total sample size (n). Edges represent direct treatment comparisons, with edge thickness proportional to the number of studies contributing to each comparison. Separate network plots are shown for: (A) Physical function, (B) Quadriceps strength, (C) Pain, and (D) Health-related quality of life. NMES, neuromuscular electrical stimulation.

S20: Network Meta-Analysi Forest Plots (4–8 Weeks)

A. Physical Function

Treatment	SMD	95%−CI	95%−PI	Direct comp.	Prop. direct	Mean path	Min. parallelism
Standard care (n=185)	0.00	[0.00; 0.00]					
Upper−body strength (n=10)	0.24	[−1.02; 1.51]	[−1.58; 2.07]	0	0.00	2.14	1.00
Multidomain exercise (n=115)	0.29	[−0.16; 0.74]	[−0.78; 1.36]	4	0.95	1.09	2.06
NMES (n=29)	0.29	[−0.49; 1.07]	[−1.04; 1.62]	2	1.00	1.00	1.00
Home−based Multidomain (n=26)	0.32	[−0.50; 1.14]	[−1.05; 1.69]	1	0.81	1.42	1.00
Lower−extremity strength (n=92)	0.32	[−0.19; 0.83]	[−0.79; 1.43]	3	0.93	1.14	1.58
Education (n=36)	0.39	[−0.73; 1.52]	[−1.28; 2.06]	0	0.00	2.09	1.00
Exercise & Education (n=21)	0.44	[−1.03; 1.91]	[−1.61; 2.49]	0	0.00	3.09	1.00

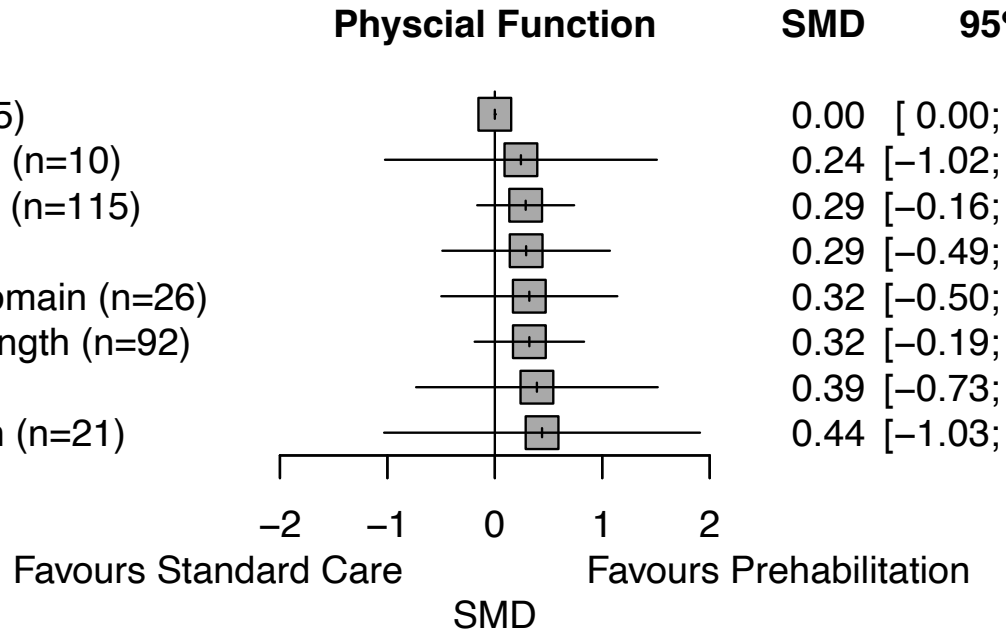

B. Quadriceps Strength

Treatment	SMD	95%−CI	95%−PI	Direct comp.	Prop. direct	Mean path	Min. parallelism
Multidomain exercise (n=71)	−0.19	[−0.54; 0.17]	[−0.77; 0.4]	3	0.94	1.13	2.96
Standard care (n=160)	0.00	[0.00; 0.00]					
Home−based Multidomain (n=26)	0.03	[−0.54; 0.60]	[−0.77; 0.83]	1	0.83	1.39	1.00
NMES (n=29)	0.06	[−0.53; 0.65]	[−0.77; 0.89]	2	1.00	1.00	1.00
Upper−body strength (n=10)	0.41	[−0.60; 1.42]	[−0.9; 1.72]	0	0.00	2.11	1.00
Lower−extremity strength (n=102)	0.50	[0.16; 0.83]	[−0.07; 1.06]	4	0.94	1.11	1.45

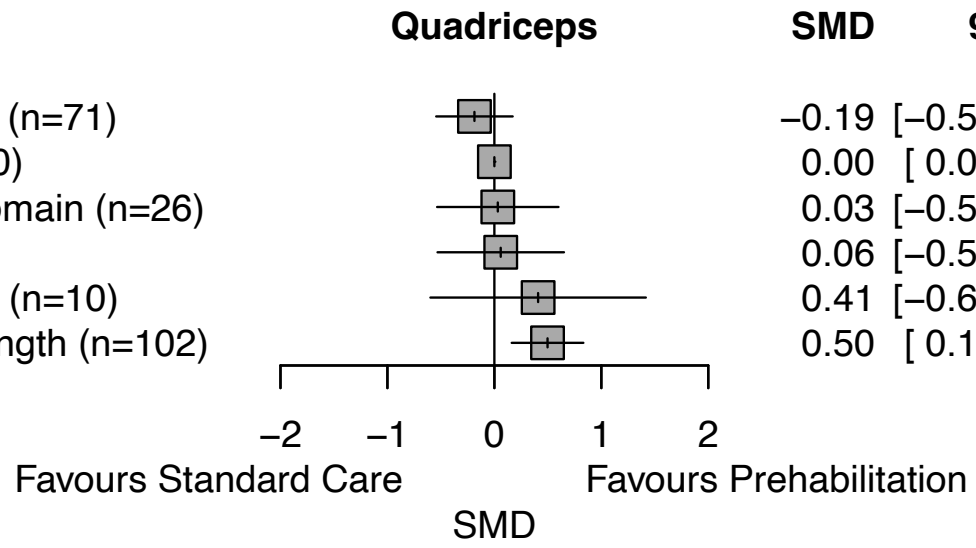

C. Pain

Treatment	SMD	95%−CI	95%−PI	Direct comp.	Prop. direct	Mean path	Min. parallelism
Exercise & Education (n=21)	−0.40	[−1.78; 0.98]	[−2.3; 1.5]	0	0.00	3.09	1.00
Education (n=36)	−0.08	[−1.14; 0.98]	[−1.63; 1.47]	0	0.00	2.09	1.00
Standard care (n=185)	0.00	[0.00; 0.00]					
Multidomain exercise (n=115)	0.02	[−0.39; 0.44]	[−0.94; 0.98]	4	0.95	1.09	2.05
NMES (n=29)	0.09	[−0.64; 0.83]	[−1.13; 1.31]	2	1.00	1.00	1.00
Home−based Multidomain (n=26)	0.17	[−0.59; 0.93]	[−1.07; 1.42]	1	0.81	1.42	1.00
Lower−extremity strength (n=92)	0.48	[0.00; 0.95]	[−0.52; 1.48]	3	0.93	1.14	1.58
Upper−body strength (n=10)	0.66	[−0.55; 1.86]	[−1.05; 2.37]	0	0.00	2.14	1.00

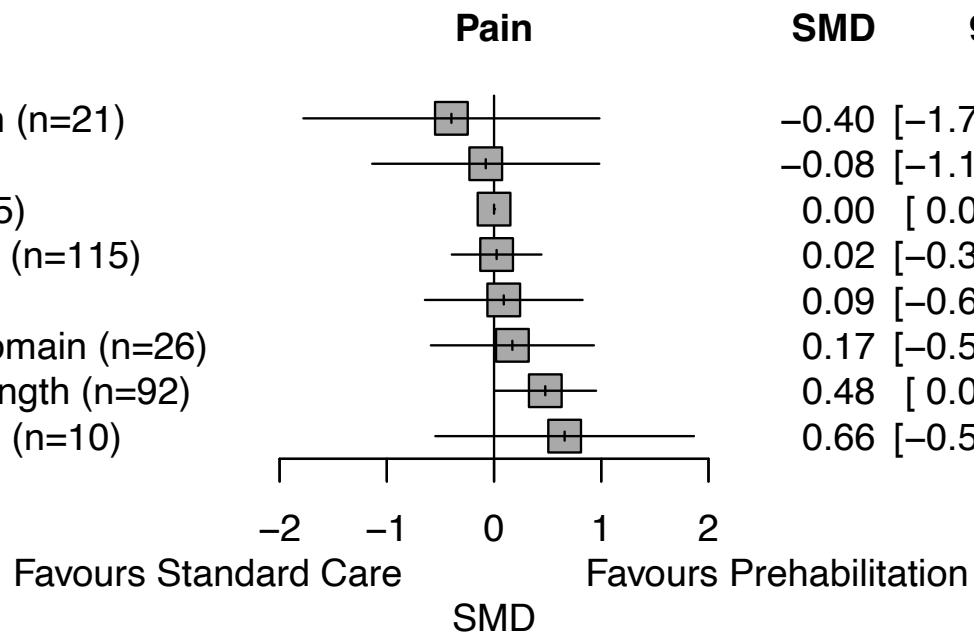

D. Health-Related Quality of Life

Treatment	SMD	95%−CI	95%−PI	Direct comp.	Prop. direct	Mean path	Min. parallelism
Upper−body strength (n=10)	−0.11	[−1.20; 0.97]	[−1.75; 1.52]	0	0.00	2.19	1.00
Standard care (n=135)	0.00	[0.00; 0.00]					
Multidomain exercise (n=86)	0.05	[−0.32; 0.42]	[−0.71; 0.81]	3	0.96	1.08	2.50
Lower−extremity strength (n=62)	0.15	[−0.31; 0.61]	[−0.7; 1.01]	2	0.90	1.19	1.88
NMES (n=29)	0.18	[−0.44; 0.81]	[−0.85; 1.22]	2	1.00	1.00	1.00
Home−based Multidomain (n=26)	0.50	[−0.11; 1.11]	[−0.53; 1.52]	1	0.83	1.38	1.00

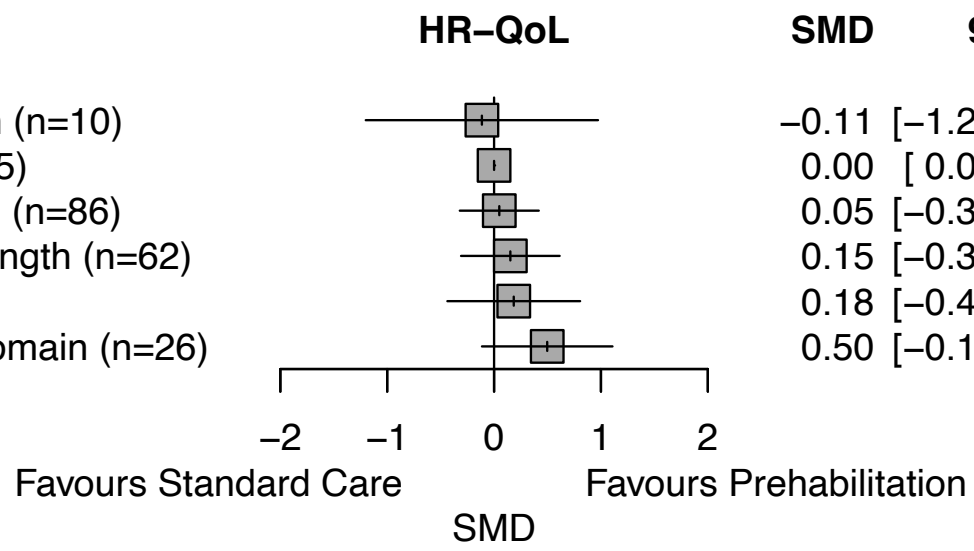

Network meta-analysis forest plots 4-8 weeks after TKA. Standardized mean differences (SMDs) with 95% confidence intervals (CIs) and prediction intervals (PIs) for all modalities versus standard care. Positive SMDs favor the prehabilitation modality. Outcome measures: (A) Physical function, (B) Quadriceps strength, (C) Pain, and (D) Health-related quality of life (HR-QoL). Direct comp, number of direct comparisons; Mean path, mean path length, indicates indirectness (shorter = more direct evidence); Min. parallelism, the minimum number of independent evidence paths; n, number of participants per modality; NMES, neuromuscular electrical stimulation; Prop. direct, proportion of direct evidence.

S21A. Forest Plots of Study-Level Pairwise Comparisons (4–8 Weeks)

Physical Function

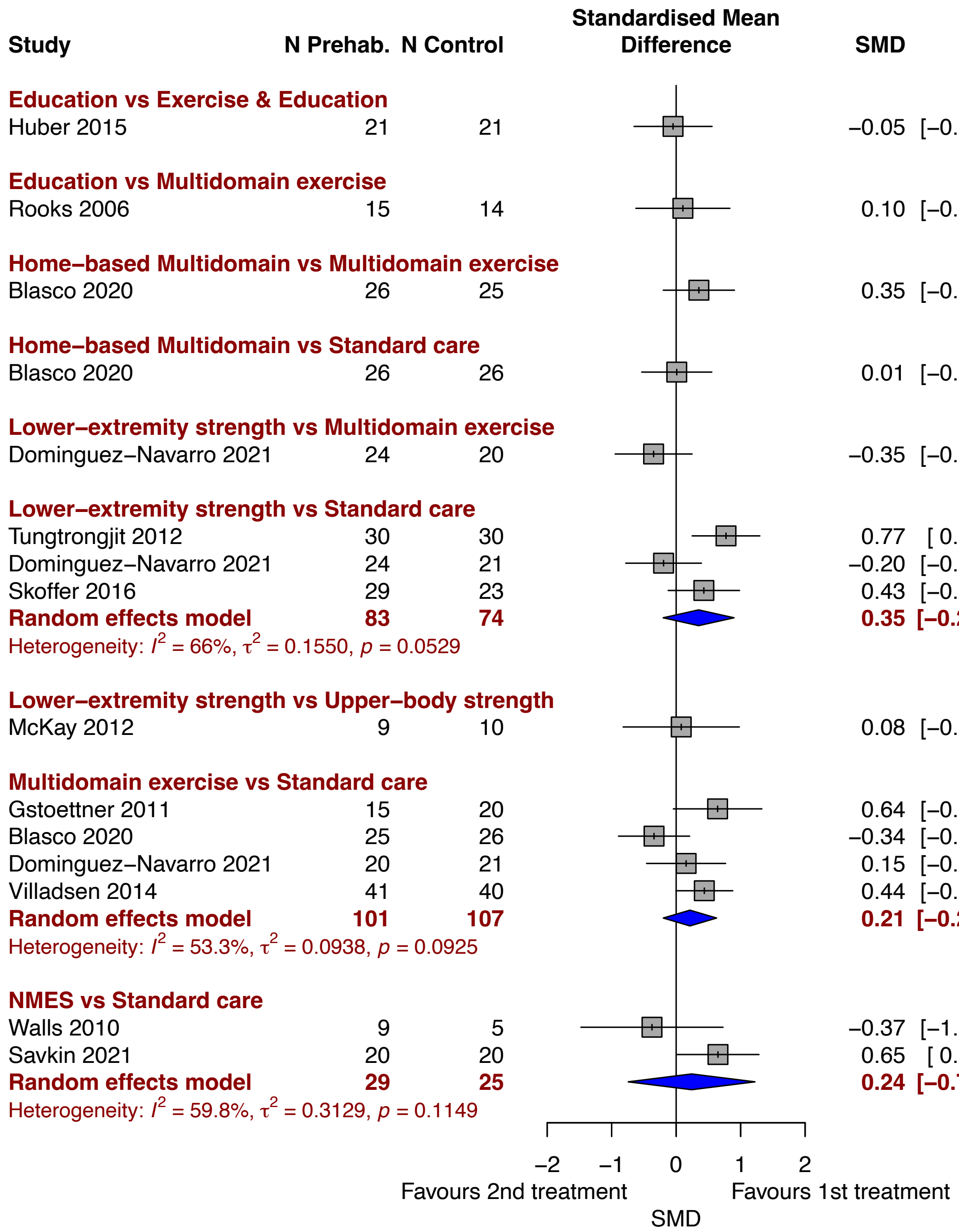

Study-level pairwise treatment comparisons for physical function at 4–8 weeks after TKA. Results are presented as standardized mean differences (SMD) with 95% confidence intervals (CI). Positive SMDs favor the first treatment listed in each comparison; negative SMDs favor the second treatment. NMES, neuromuscular electrical stimulation.

S21B. Forest Plots of Study-Level Pairwise Comparisons (4–8 Weeks)

Quadriceps Strength

Study	N Prehab.	N Control	SMD	95% CI	SE
**Home–based Multidomain vs Multidomain exercise**					
Blasco 2020	26	25	0.27	[−0.28; 0.82]	0.28
**Home–based Multidomain vs Standard care**					
Blasco 2020	26	26	−0.02	[−0.56; 0.53]	0.28
**Lower–extremity strength vs Multidomain exercise**					
Dominguez–Navarro 2021	24	20	0.39	[−0.20; 0.99]	0.30
**Lower–extremity strength vs Standard care**					
Dominguez–Navarro 2021	24	21	0.40	[−0.19; 0.99]	0.30
Leeuwen 2014	10	7	−0.22	[−1.19; 0.75]	0.49
Skoffer 2016	29	23	1.00	[0.42; 1.58]	0.30
Tungtrongjit 2012	30	30	0.54	[0.02; 1.05]	0.26
**Random effects model**	**93**	**81**	**0.52**	**[0.12; 0.93]**	
Heterogeneity: $I^2 = 39.8\%$, $\tau^2 = 0.0676$, $p = 0.1729$					
**Lower–extremity strength vs Upper–body strength**					
McKay 2012	9	10	0.09	[−0.81; 0.99]	0.46
**Multidomain exercise vs Standard care**					
Blasco 2020	25	26	−0.29	[−0.84; 0.26]	0.28
Dominguez–Navarro 2021	20	21	0.00	[−0.61; 0.62]	0.31
Topp 2009	26	28	−0.35	[−0.89; 0.18]	0.27
**Random effects model**	**71**	**75**	**−0.23**	**[−0.56; 0.10]**	
Heterogeneity: $I^2 = 0\%$, $\tau^2 = 0$, $p = 0.6669$					
**NMES vs Standard care**					
Savkin 2021	20	20	−0.11	[−0.73; 0.51]	0.32
Walls 2010	9	5	0.53	[−0.59; 1.64]	0.57
**Random effects model**	**29**	**25**	**0.04**	**[−0.50; 0.58]**	
Heterogeneity: $I^2 = 0\%$, $\tau^2 = 0$, $p = 0.3295$					

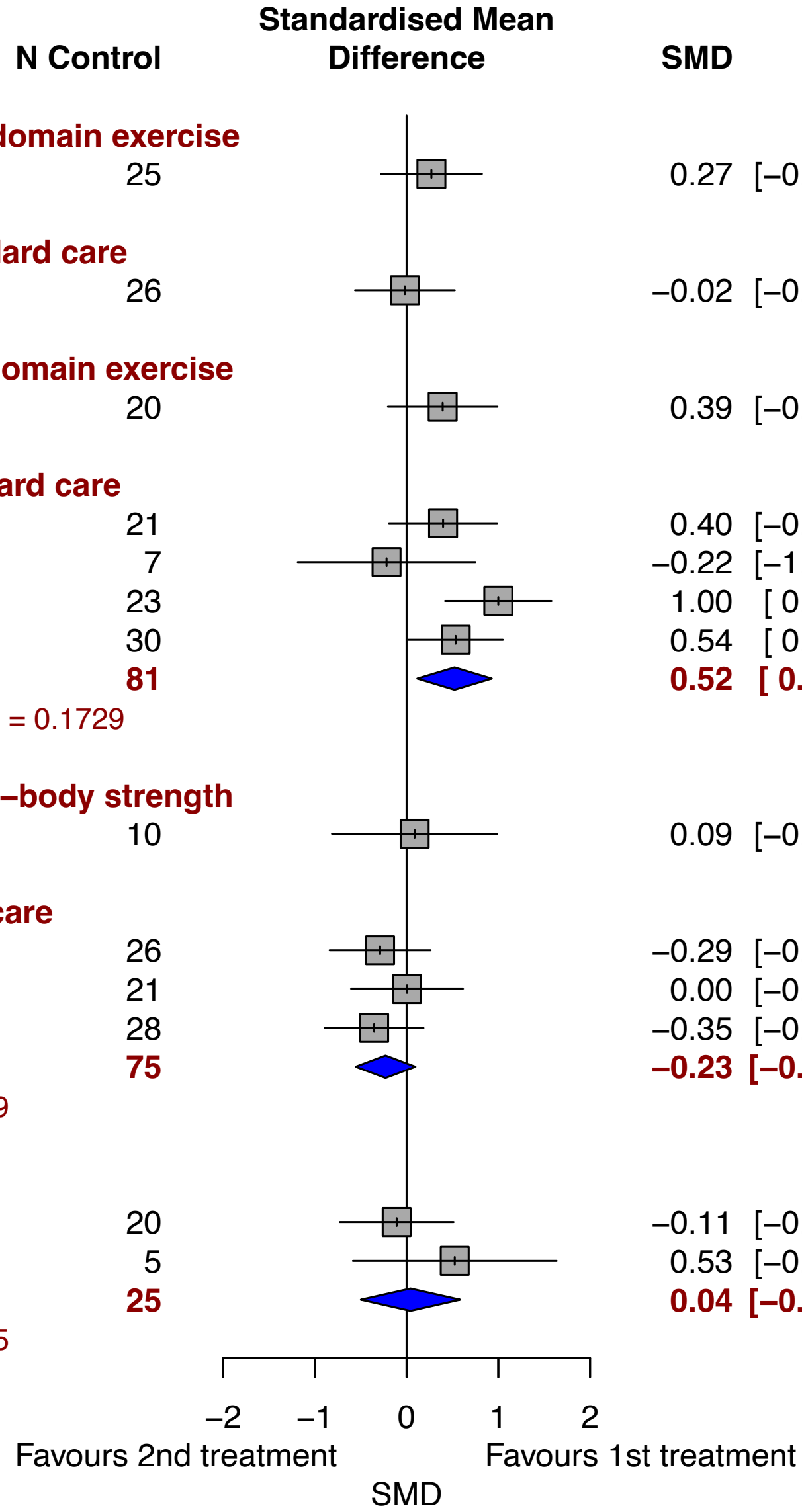

Study-level pairwise treatment comparisons for quadriceps strength outcomes at 4–8 weeks after TKA. Results are presented as standardized mean differences (SMD) with 95% confidence intervals (CI). Positive SMDs favor the first treatment listed in each comparison; negative SMDs favor the second treatment. NMES, neuromuscular electrical stimulation.

S21C. Forest Plots of Study-Level Pairwise Comparisons (4–8 Weeks)

Pain

Study	N Prehab.	N Control	SMD	95% CI	SE
**Education vs Exercise & Education**					
Huber 2015	21	21	0.32	[−0.29; 0.93]	0.31
**Education vs Multidomain exercise**					
Rooks 2006	15	14	−0.10	[−0.83; 0.63]	0.37
**Home−based Multidomain vs Multidomain exercise**					
Blasco 2020	26	25	0.25	[−0.30; 0.80]	0.28
**Home−based Multidomain vs Standard care**					
Blasco 2020	26	26	0.07	[−0.48; 0.61]	0.28
**Lower−extremity strength vs Multidomain exercise**					
Dominguez−Navarro 2021	24	20	0.30	[−0.29; 0.90]	0.30
**Lower−extremity strength vs Standard care**					
Tungtrongjit 2012	30	30	1.04	[0.50; 1.58]	0.28
Dominguez−Navarro 2021	24	21	0.11	[−0.47; 0.70]	0.30
Skoffer 2016	29	23	0.24	[−0.31; 0.79]	0.28
**Random effects model**	**83**	**74**	**0.47**	**[−0.11; 1.05]**	
Heterogeneity: $I^2 = 68.7\%$, $\tau^2 = 0.1780$, $p = 0.0408$					
**Lower−extremity strength vs Upper−body strength**					
McKay 2012	9	10	−0.18	[−1.08; 0.72]	0.46
**Multidomain exercise vs Standard care**					
Gstoettner 2011	15	20	−0.31	[−0.98; 0.37]	0.34
Blasco 2020	25	26	−0.18	[−0.73; 0.37]	0.28
Dominguez−Navarro 2021	20	21	−0.19	[−0.80; 0.42]	0.31
Villadsen 2014	41	40	0.46	[0.02; 0.90]	0.23
**Random effects model**	**101**	**107**	**−0.01**	**[−0.39; 0.38]**	
Heterogeneity: $I^2 = 46.9\%$, $\tau^2 = 0.0717$, $p = 0.1301$					
**NMES vs Standard care**					
Walls 2010	9	5	−0.49	[−1.60; 0.62]	0.57
Savkin 2021	20	20	0.37	[−0.25; 1.00]	0.32
**Random effects model**	**29**	**25**	**0.07**	**[−0.74; 0.88]**	
Heterogeneity: $I^2 = 43.4\%$, $\tau^2 = 0.1616$, $p = 0.1838$					

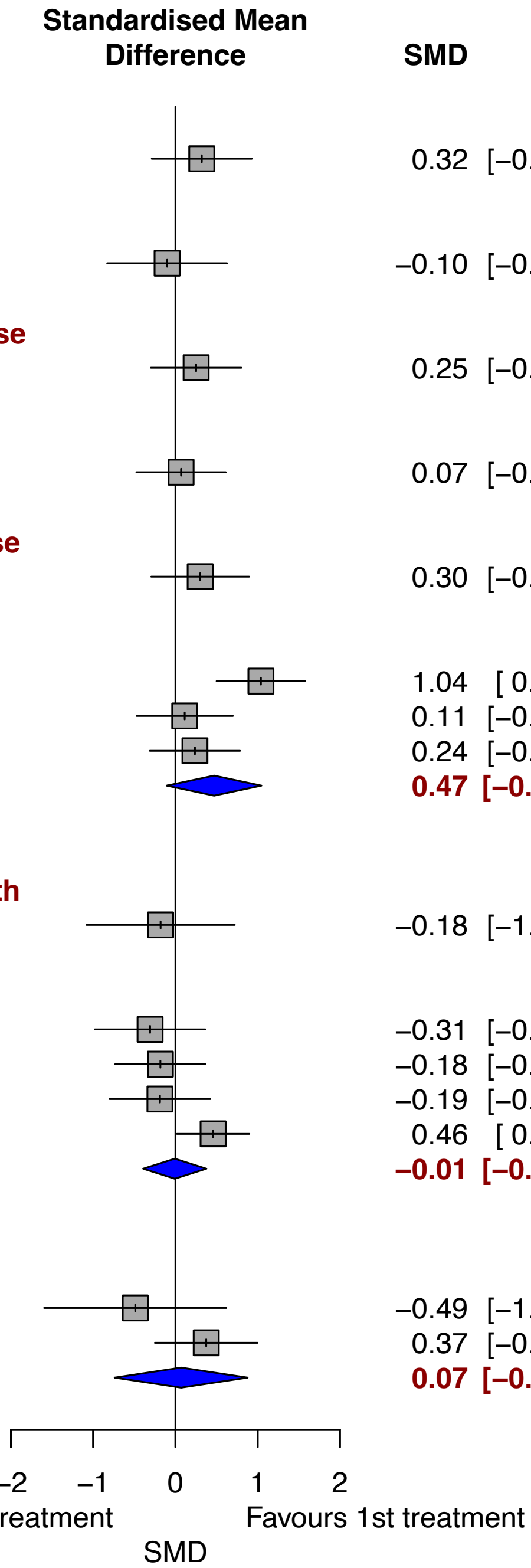

Study-level pairwise treatment comparisons for pain outcomes at 4–8 weeks after TKA. Results are presented as standardized mean differences (SMD) with 95% confidence intervals (CI). Positive SMDs favor the first treatment listed in each comparison; negative SMDs favor the second treatment. NMES, neuromuscular electrical stimulation.

S21D. Forest Plots of Study-Level Pairwise Comparisons (4–8 Weeks)

Health-Related Quality of Life

Study	N Prehab.	N Control	SMD	95% CI	SE
**Home–based Multidomain vs Multidomain exercise**					
Blasco 2020	26	25	0.70	[0.14; 1.26]	0.29
**Home–based Multidomain vs Standard care**					
Blasco 2020	26	26	0.26	[−0.28; 0.81]	0.28
**Lower–extremity strength vs Multidomain exercise**					
Dominguez–Navarro 2021	24	20	−0.12	[−0.71; 0.48]	0.30
**Lower–extremity strength vs Standard care**					
Dominguez–Navarro 2021	24	21	0.04	[−0.54; 0.63]	0.30
Skoffer 2016	29	23	0.35	[−0.20; 0.91]	0.28
**Random effects model**	**53**	**44**	**0.21**	**[−0.19; 0.61]**	
Heterogeneity: $I^2 = 0\%$, $\tau^2 = 0$, $p = 0.4488$					
**Lower–extremity strength vs Upper–body strength**					
McKay 2012	9	10	0.27	[−0.64; 1.17]	0.46
**Multidomain exercise vs Standard care**					
Blasco 2020	25	26	−0.44	[−0.99; 0.12]	0.28
Dominguez–Navarro 2021	20	21	0.16	[−0.45; 0.77]	0.31
Villadsen 2014	41	40	0.27	[−0.17; 0.70]	0.22
**Random effects model**	**86**	**87**	**0.01**	**[−0.42; 0.45]**	
Heterogeneity: $I^2 = 50.4\%$, $\tau^2 = 0.0745$, $p = 0.1330$					
**NMES vs Standard care**					
Savkin 2021	20	20	0.35	[−0.28; 0.97]	0.32
Walls 2010	9	5	−0.22	[−1.32; 0.87]	0.56
**Random effects model**	**29**	**25**	**0.21**	**[−0.34; 0.75]**	
Heterogeneity: $I^2 = 0\%$, $\tau^2 = 0$, $p = 0.3779$					

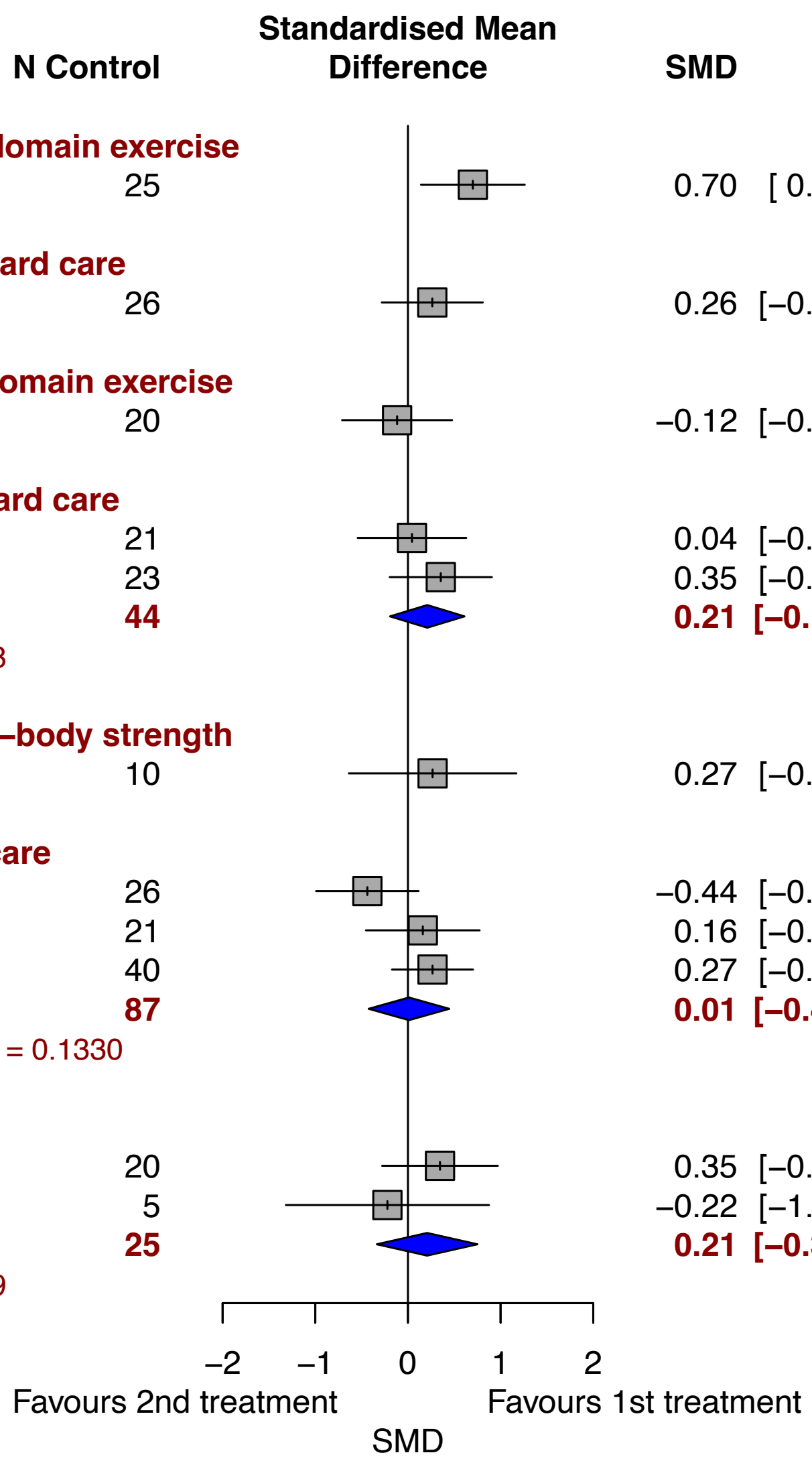

Study-level pairwise treatment comparisons for health-related quality of life outcomes at 4–8 weeks after TKA. Results are presented as standardized mean differences (SMD) with 95% confidence intervals (CI). Positive SMDs favor the first treatment listed in each comparison; negative SMDs favor the second treatment. NMES, neuromuscular electrical stimulation.

S22. Probability Ranking Heatmap of Prehabilitation Modalities (4–8 Weeks)

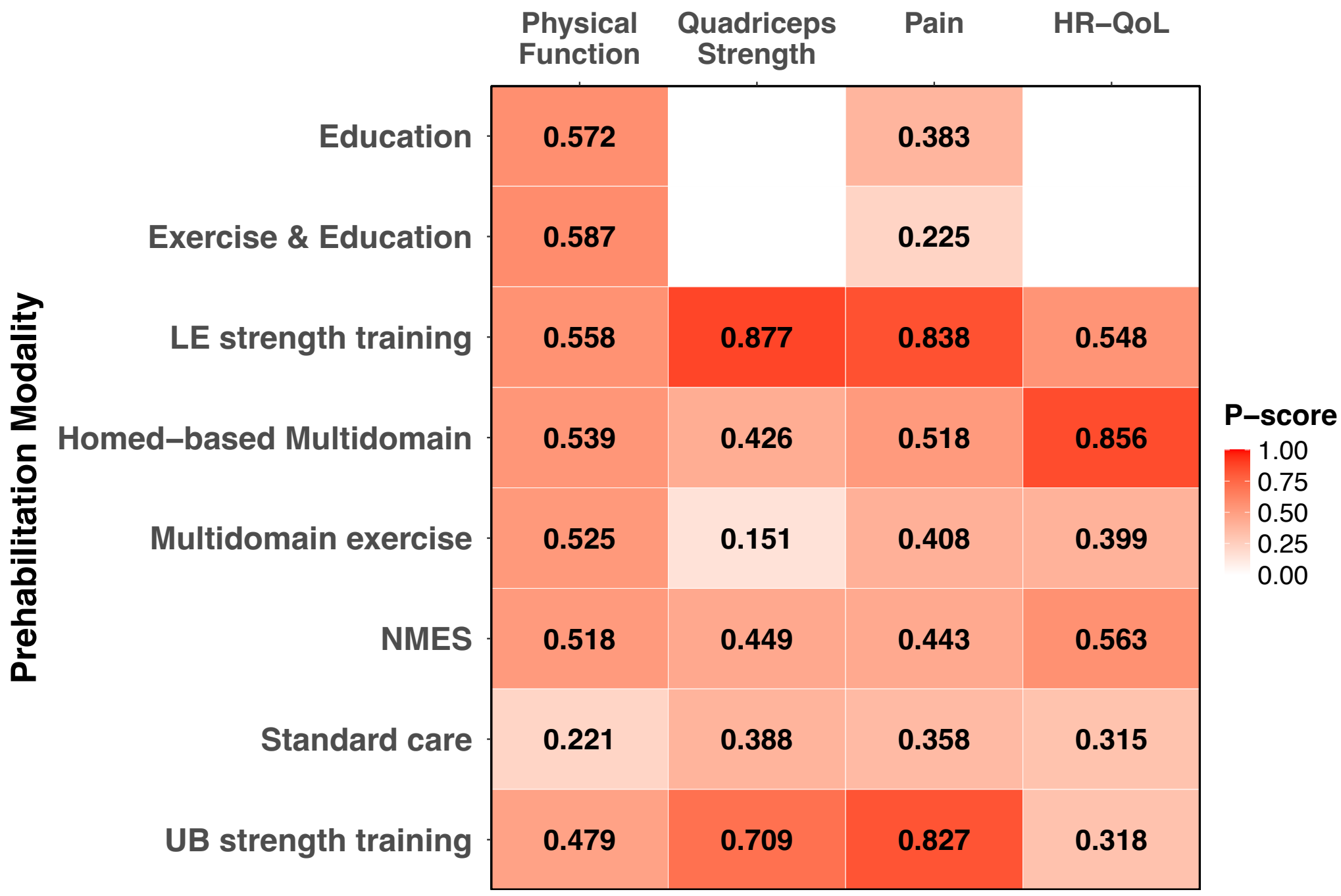

Probability ranking heatmap of prehabilitation modalities at 4–8 weeks after TKA. Heatmap showing the relative ranking of prehabilitation modalities across physical function, quadriceps strength, pain, and health-related quality of life (HR-QoL). Cells represent P-scores ranging from 0 to 1, with higher values indicating a more favorable relative ranking for the corresponding outcome. Empty white boxes indicate that the respective modality was not included in the network for the corresponding outcome. LE, lower-extremity; NMES, neuromuscular electrical stimulation; UB, upper body.

S23A CINeMA Assessment of Network Confidence (4–8 Weeks)

Physical Function

TREATMENT COMPARISON	NUMBER of STUDIES	WITHIN-STUDY BIAS	REPORTING BIAS	INDIRECTNESS	IMPRECISION	HETEROGENEITY	INCOHERENCE	CONFIDENCE RATING
MIXED ESTIMATES								
Education:Exercise & Education	1	Some concerns	Low risk	Some concerns	Major concerns	No concerns	Some concerns	**Low**
Education:Multidomain exercise	1	Some concerns	Low risk	Some concerns	Major concerns	No concerns	Some concerns	**Low**
Home-based Multidomain:Multidomain exercise	1	Major concerns	Low risk	Some concerns	Major concerns	No concerns	No concerns	**Very Low**
Home-based Multidomain:Standard care	1	Major concerns	Low risk	Some concerns	Some concerns	Some concerns	No concerns	**Low**
Lower-extremity strength:Multidomain exercise	1	Major concerns	Low risk	Some concerns	Major concerns	No concerns	No concerns	**Very Low**
Lower-extremity strength:Standard care	3	Major concerns	Low risk	Some concerns	Some concerns	Some concerns	No concerns	**Low**
Lower-extremity strength:Upper-body strength	1	Some concerns	Low risk	Some concerns	Major concerns	No concerns	Some concerns	**Low**
Multidomain exercise:Standard care	4	Major concerns	Low risk	Some concerns	Some concerns	Some concerns	No concerns	**Low**
NMES:Standard care	2	Some concerns	Low risk	Some concerns	Some concerns	Some concerns	Some concerns	**Low**
INDIRECT ESTIMATES								
Education:Home-based Multidomain	0	Major concerns	Low risk	Some concerns	Major concerns	No concerns	Some concerns	**Very Low**
Education:Lower-extremity strength	0	Some concerns	Low risk	Some concerns	Major concerns	No concerns	Some concerns	**Very Low**
Education:NMES	0	Some concerns	Low risk	Some concerns	Major concerns	No concerns	Some concerns	**Very Low**
Education:Standard care	0	Some concerns	Low risk	Some concerns	Major concerns	No concerns	Some concerns	**Very Low**
Education:Upper-body strength	0	Some concerns	Low risk	Some concerns	Major concerns	No concerns	Some concerns	**Very Low**
Exercise & Education:Home-based Multidomain	0	Some concerns	Low risk	Some concerns	Major concerns	No concerns	Some concerns	**Very Low**
Exercise & Education:Lower-extremity strength	0	Some concerns	Low risk	Some concerns	Major concerns	No concerns	Some concerns	**Very Low**
Exercise & Education:Multidomain exercise	0	Some concerns	Low risk	Some concerns	Major concerns	No concerns	Some concerns	**Very Low**
Exercise & Education:NMES	0	Some concerns	Low risk	Some concerns	Major concerns	No concerns	Some concerns	**Very Low**
Exercise & Education:Standard care	0	Some concerns	Low risk	Some concerns	Major concerns	No concerns	Some concerns	**Very Low**
Exercise & Education:Upper-body strength	0	Some concerns	Low risk	Some concerns	Major concerns	No concerns	Some concerns	**Very Low**
Home-based Multidomain:Lower-extremity strength	0	Major concerns	Low risk	Some concerns	Major concerns	No concerns	Some concerns	**Very Low**
Home-based Multidomain:NMES	0	Major concerns	Low risk	Some concerns	Major concerns	No concerns	Some concerns	**Very Low**
Home-based Multidomain:Upper-body strength	0	Major concerns	Low risk	Some concerns	Major concerns	No concerns	Some concerns	**Very Low**
Lower-extremity strength:NMES	0	Some concerns	Low risk	Some concerns	Major concerns	No concerns	Some concerns	**Very Low**
Multidomain exercise:NMES	0	Some concerns	Low risk	Some concerns	Major concerns	No concerns	Some concerns	**Very Low**
Multidomain exercise:Upper-body strength	0	Some concerns	Low risk	Some concerns	Major concerns	No concerns	Some concerns	**Very Low**
NMES:Upper-body strength	0	Some concerns	Low risk	Some concerns	Major concerns	No concerns	Some concerns	**Very Low**
Standard care:Upper-body strength	0	Some concerns	Low risk	Some concerns	Major concerns	No concerns	Some concerns	**Very Low**

CINeMA: Confidence in Network Meta-Analysis for physical function at 4–8 weeks after TKA. Six domains influencing confidence in network meta-analysis results are assessed: (a) within-study bias, (b) reporting bias, (c) indirectness, (d) imprecision, (e) heterogeneity, and (f) incoherence. Confidence levels are categorized as high, moderate, low, or very low. Imprecision, heterogeneity, and incoherence were assessed using a clinically important SMD threshold of ±0.5, considering the position of confidence intervals, prediction intervals, and direct–indirect disagreement relative to this threshold. NMES, neuromuscular electrical stimulation.

S23B CINeMA Assessment of Network Confidence (4–8 Weeks)

Quadriceps Strength

TREATMENT COMPARISON	NUMBER of STUDIES	WITHIN-STUDY BIAS	REPORTING BIAS	INDIRECTNESS	IMPRECISION	HETEROGENEITY	INCOHERENCE	CONFIDENCE RATING
MIXED ESTIMATES								
Home-based Multidomain:Multidomain exercise	1	Major concerns	Low risk	Some concerns	Some concerns	Some concerns	No concerns	**Low**
Home-based Multidomain:Standard care	1	Major concerns	Low risk	Some concerns	Major concerns	No concerns	No concerns	**Very Low**
Lower-extremity strength:Multidomain exercise	1	Major concerns	Low risk	Some concerns	No concerns	No concerns	No concerns	**Low**
Lower-extremity strength:Standard care	4	Major concerns	Low risk	Some concerns	No concerns	Some concerns	No concerns	**Low**
Lower-extremity strength:Upper-body strength	1	Some concerns	Low risk	Some concerns	Major concerns	No concerns	No concerns	**Low**
Multidomain exercise:Standard care	3	Major concerns	Low risk	Some concerns	Some concerns	No concerns	No concerns	**Low**
NMES:Standard care	2	Some concerns	Low risk	Some concerns	Major concerns	No concerns	No concerns	**Low**
INDIRECT ESTIMATES								
Home-based Multidomain:Lower-extremity strength	0	Major concerns	Low risk	Some concerns	Some concerns	No concerns	No concerns	**Very Low**
Home-based Multidomain:NMES	0	Major concerns	Low risk	Some concerns	Major concerns	No concerns	No concerns	**Very Low**
Home-based Multidomain:Upper-body strength	0	Major concerns	Low risk	Some concerns	Major concerns	No concerns	No concerns	**Very Low**
Lower-extremity strength:NMES	0	Some concerns	Low risk	Some concerns	Some concerns	No concerns	No concerns	**Low**
Multidomain exercise:NMES	0	Some concerns	Low risk	Some concerns	Some concerns	Some concerns	No concerns	**Low**
Multidomain exercise:Upper-body strength	0	Some concerns	Low risk	Some concerns	Some concerns	Some concerns	No concerns	**Low**
NMES:Upper-body strength	0	Some concerns	Low risk	Some concerns	Major concerns	No concerns	No concerns	**Very Low**
Standard care:Upper-body strength	0	Some concerns	Low risk	Some concerns	Major concerns	No concerns	No concerns	**Very Low**

CINeMA: Confidence in Network Meta-Analysis for quadriceps strength at 4–8 weeks after TKA. Six domains influencing confidence in network meta-analysis results are assessed: (a) within-study bias, (b) reporting bias, (c) indirectness, (d) imprecision, (e) heterogeneity, and (f) incoherence. Confidence levels are categorized as high, moderate, low, or very low. Imprecision, heterogeneity, and incoherence were assessed using a clinically important SMD threshold of ±0.5, considering the position of confidence intervals, prediction intervals, and direct–indirect disagreement relative to this threshold. NMES, neuromuscular electrical stimulation.

S23C CINeMA Assessment of Network Confidence (4–8 Weeks)

Pain

TREATMENT COMPARISON	NUMBER of STUDIES	WITHIN-STUDY BIAS	REPORTING BIAS	INDIRECTNESS	IMPRECISION	HETEROGENEITY	INCOHERENCE	CONFIDENCE RATING
MIXED ESTIMATES								
Education:Exercise & Education	1	Some concerns	Low risk	Some concerns	Major concerns	No concerns	No concerns	**Low**
Education:Multidomain exercise	1	Some concerns	Low risk	Some concerns	Major concerns	No concerns	No concerns	**Low**
Home-based Multidomain:Multidomain exercise	1	Major concerns	Low risk	Some concerns	Major concerns	No concerns	No concerns	**Very Low**
Home-based Multidomain:Standard care	1	Major concerns	Low risk	Some concerns	Major concerns	No concerns	No concerns	**Very Low**
Lower-extremity strength:Multidomain exercise	1	Major concerns	Low risk	Some concerns	Some concerns	Some concerns	No concerns	**Low**
Lower-extremity strength:Standard care	3	Major concerns	Low risk	Some concerns	No concerns	Major concerns	No concerns	**Very Low**
Lower-extremity strength:Upper-body strength	1	Some concerns	Low risk	Some concerns	Major concerns	No concerns	No concerns	**Low**
Multidomain exercise:Standard care	4	Major concerns	Low risk	Some concerns	No concerns	Major concerns	No concerns	**Very Low**
NMES:Standard care	2	Some concerns	Low risk	Some concerns	Major concerns	No concerns	No concerns	**Low**
INDIRECT ESTIMATES								
Education:Home-based Multidomain	0	Major concerns	Low risk	Some concerns	Major concerns	No concerns	No concerns	**Very Low**
Education:Lower-extremity strength	0	Some concerns	Low risk	Some concerns	Major concerns	No concerns	No concerns	**Very Low**
Education:NMES	0	Some concerns	Low risk	Some concerns	Major concerns	No concerns	No concerns	**Very Low**
Education:Standard care	0	Some concerns	Low risk	Some concerns	Major concerns	No concerns	No concerns	**Very Low**
Education:Upper-body strength	0	Some concerns	Low risk	Some concerns	Major concerns	No concerns	No concerns	**Very Low**
Exercise & Education:Home-based Multidomain	0	Some concerns	Low risk	Some concerns	Major concerns	No concerns	No concerns	**Very Low**
Exercise & Education:Lower-extremity strength	0	Some concerns	Low risk	Some concerns	Major concerns	No concerns	No concerns	**Very Low**
Exercise & Education:Multidomain exercise	0	Some concerns	Low risk	Some concerns	Major concerns	No concerns	No concerns	**Very Low**
Exercise & Education:NMES	0	Some concerns	Low risk	Some concerns	Major concerns	No concerns	No concerns	**Very Low**
Exercise & Education:Standard care	0	Some concerns	Low risk	Some concerns	Major concerns	No concerns	No concerns	**Very Low**
Exercise & Education:Upper-body strength	0	Some concerns	Low risk	Some concerns	Major concerns	No concerns	No concerns	**Very Low**
Home-based Multidomain:Lower-extremity strength	0	Major concerns	Low risk	Some concerns	Major concerns	No concerns	No concerns	**Very Low**
Home-based Multidomain:NMES	0	Major concerns	Low risk	Some concerns	Major concerns	No concerns	No concerns	**Very Low**
Home-based Multidomain:Upper-body strength	0	Major concerns	Low risk	Some concerns	Major concerns	No concerns	No concerns	**Very Low**
Lower-extremity strength:NMES	0	Some concerns	Low risk	Some concerns	Some concerns	Some concerns	No concerns	**Low**
Multidomain exercise:NMES	0	Some concerns	Low risk	Some concerns	Major concerns	No concerns	No concerns	**Very Low**
Multidomain exercise:Upper-body strength	0	Some concerns	Low risk	Some concerns	Major concerns	No concerns	No concerns	**Very Low**
NMES:Upper-body strength	0	Some concerns	Low risk	Some concerns	Major concerns	No concerns	No concerns	**Very Low**
Standard care:Upper-body strength	0	Some concerns	Low risk	Some concerns	Major concerns	No concerns	No concerns	**Very Low**

CINeMA: Confidence in Network Meta-Analysis for pain at 4–8 weeks after TKA. Six domains influencing confidence in network meta-analysis results are assessed: (a) within-study bias, (b) reporting bias, (c) indirectness, (d) imprecision, (e) heterogeneity, and (f) incoherence. Confidence levels are categorized as high, moderate, low, or very low. Imprecision, heterogeneity, and incoherence were assessed using a clinically important SMD threshold of ±0.5, considering the position of confidence intervals, prediction intervals, and direct–indirect disagreement relative to this threshold. NMES, neuromuscular electrical stimulation.

S23D CINeMA Assessment of Network Confidence (4–8 Weeks)

Health-Related Quality of Life

TREATMENT COMPARISON	NUMBER of STUDIES	WITHIN-STUDY BIAS	REPORTING BIAS	INDIRECTNESS	IMPRECISION	HETEROGENEITY	INCOHERENCE	CONFIDENCE RATING
MIXED ESTIMATES								
Home-based Multidomain:Multidomain exercise	1	Major concerns	Low risk	Some concerns	Some concerns	Some concerns	Some concerns	**Low**
Home-based Multidomain:Standard care	1	Major concerns	Low risk	Some concerns	Some concerns	Some concerns	No concerns	**Low**
Lower-extremity strength:Multidomain exercise	1	Major concerns	Low risk	Some concerns	Some concerns	Some concerns	No concerns	**Low**
Lower-extremity strength:Standard care	2	Some concerns	Low risk	Some concerns	Some concerns	Some concerns	No concerns	**Low**
Lower-extremity strength:Upper-body strength	1	Some concerns	Low risk	Some concerns	Major concerns	No concerns	No concerns	**Low**
Multidomain exercise:Standard care	3	Major concerns	Low risk	Some concerns	No concerns	Major concerns	No concerns	**Very Low**
NMES:Standard care	2	Some concerns	Low risk	Some concerns	Some concerns	Some concerns	No concerns	**Low**
INDIRECT ESTIMATES								
Home-based Multidomain:Lower-extremity strength	0	Major concerns	Low risk	Some concerns	Some concerns	Some concerns	No concerns	**Very Low**
Home-based Multidomain:NMES	0	Major concerns	Low risk	Some concerns	Major concerns	No concerns	No concerns	**Very Low**
Home-based Multidomain:Upper-body strength	0	Major concerns	Low risk	Some concerns	Major concerns	No concerns	No concerns	**Very Low**
Lower-extremity strength:NMES	0	Some concerns	Low risk	Some concerns	Major concerns	No concerns	No concerns	**Very Low**
Multidomain exercise:NMES	0	Some concerns	Low risk	Some concerns	Major concerns	No concerns	No concerns	**Very Low**
Multidomain exercise:Upper-body strength	0	Some concerns	Low risk	Some concerns	Major concerns	No concerns	No concerns	**Very Low**
NMES:Upper-body strength	0	Some concerns	Low risk	Some concerns	Major concerns	No concerns	No concerns	**Very Low**
Standard care:Upper-body strength	0	Some concerns	Low risk	Some concerns	Major concerns	No concerns	No concerns	**Very Low**

CINeMA: Confidence in Network Meta-Analysis for health-related quality of life at 4–8 weeks after TKA. Six domains influencing confidence in network meta-analysis results are assessed: (a) within-study bias, (b) reporting bias, (c) indirectness, (d) imprecision, (e) heterogeneity, and (f) incoherence. Confidence levels are categorized as high, moderate, low, or very low. Imprecision, heterogeneity, and incoherence were assessed using a clinically important SMD threshold of ±0.5, considering the position of confidence intervals, prediction intervals, and direct–indirect disagreement relative to this threshold. NMES, neuromuscular electrical stimulation.

S24. Comparison-Adjusted Funnel Plots (4–8 Weeks)

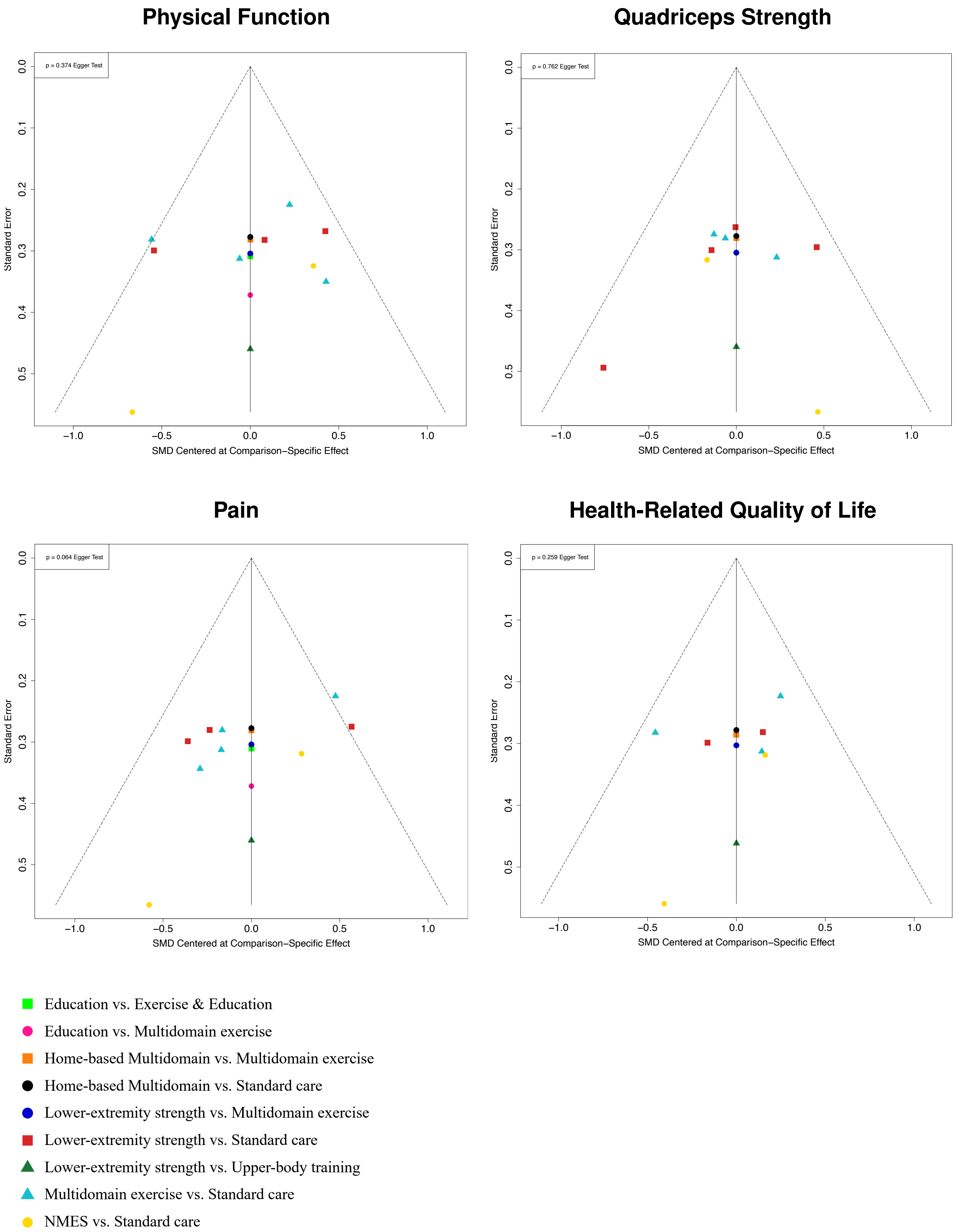

Comparison-adjusted funnel plots of standardized mean differences (SMDs) centered at the comparison-specific effect. Coloured symbols indicate treatment comparisons. P-values from Egger's regression test are presented. NMES, neuromuscular electrical stimulation.

S25. League Tables Comparing Prehabilitation Modalities (4–8 Weeks)

Physical Function

**Education**	.	-0.05 (-0.99; 0.90)	.	.	0.10 (-0.93; 1.13)	.	.
0.10 (-1.27; 1.47)	**NMES**	.	.	.	.	0.29 (-0.49; 1.07)	.
-0.05 (-0.99; 0.90)	-0.15 (-1.81; 1.52)	**Exercise & Education**	.	.	.	.	.
0.07 (-1.25; 1.39)	-0.03 (-1.16; 1.10)	0.12 (-1.50; 1.74)	**Home-based Multidomain**	.	0.35 (-0.56; 1.27)	0.01 (-0.90; 0.92)	.
0.07 (-1.13; 1.27)	-0.03 (-0.96; 0.90)	0.12 (-1.41; 1.65)	-0.00 (-0.94; 0.94)	**LE-strength**	-0.35 (-1.29; 0.59)	0.35 (-0.18; 0.88)	0.08 (-1.08; 1.24)
0.10 (-0.93; 1.13)	0.00 (-0.90; 0.91)	0.15 (-1.25; 1.55)	0.03 (-0.79; 0.85)	0.03 (-0.59; 0.66)	**Multidomain exercise**	0.21 (-0.25; 0.68)	.
0.39 (-0.73; 1.52)	0.29 (-0.49; 1.07)	0.44 (-1.03; 1.91)	0.32 (-0.50; 1.14)	0.32 (-0.19; 0.83)	0.29 (-0.16; 0.74)	**Standard care**	.
0.15 (-1.52; 1.82)	0.05 (-1.44; 1.53)	0.20 (-1.72; 2.12)	0.08 (-1.42; 1.57)	0.08 (-1.08; 1.24)	0.04 (-1.27; 1.36)	-0.24 (-1.51; 1.02)	**Upper body strength**

Quadriceps Strength

**NMES**	.	.	.	0.06 (-0.53; 0.65)	.
0.03 (-0.79; 0.85)	**Home-based Multidomain**	.	0.27 (-0.36; 0.90)	-0.02 (-0.64; 0.60)	.
-0.44 (-1.12; 0.24)	-0.46 (-1.11; 0.18)	**LE-strength**	0.39 (-0.28; 1.06)	0.54 (0.20; 0.89)	0.09 (-0.86; 1.04)
0.25 (-0.45; 0.94)	0.22 (-0.35; 0.79)	0.68 (0.24; 1.13)	**Multidomain exercise**	-0.23 (-0.60; 0.14)	.
0.06 (-0.53; 0.65)	0.03 (-0.54; 0.60)	0.50 (0.16; 0.83)	-0.19 (-0.54; 0.17)	**Standard care**	.
-0.35 (-1.52; 0.82)	-0.38 (-1.52; 0.77)	0.09 (-0.86; 1.04)	-0.60 (-1.64; 0.45)	-0.41 (-1.42; 0.60)	**Upper body strength**

Pain

**Education**	.	0.32 (-0.57; 1.21)	.	.	-0.10 (-1.07; 0.87)	.	.
-0.17 (-1.46; 1.12)	**NMES**	.	.	.	.	0.09 (-0.64; 0.83)	.
0.32 (-0.57; 1.21)	0.49 (-1.08; 2.05)	**Exercise & Education**	.	.	.	.	.
-0.25 (-1.48; 0.99)	-0.08 (-1.14; 0.98)	-0.57 (-2.09; 0.95)	**Home-based Multidomain**	.	0.25 (-0.60; 1.10)	0.07 (-0.77; 0.91)	.
-0.56 (-1.69; 0.58)	-0.39 (-1.26; 0.49)	-0.88 (-2.31; 0.56)	-0.31 (-1.18; 0.57)	**LE-strength**	0.30 (-0.58; 1.18)	0.47 (-0.02; 0.96)	-0.18 (-1.29; 0.93)
-0.10 (-1.07; 0.87)	0.07 (-0.78; 0.91)	-0.42 (-1.74; 0.89)	0.15 (-0.61; 0.91)	0.45 (-0.12; 1.03)	**Multidomain exercise**	-0.02 (-0.45; 0.41)	.
-0.08 (-1.14; 0.98)	0.09 (-0.64; 0.83)	-0.40 (-1.78; 0.98)	0.17 (-0.59; 0.93)	0.48 (0.00; 0.95)	0.02 (-0.39; 0.44)	**Standard care**	.
-0.74 (-2.32; 0.85)	-0.57 (-1.98; 0.84)	-1.06 (-2.87; 0.76)	-0.49 (-1.90; 0.93)	-0.18 (-1.29; 0.93)	-0.63 (-1.88; 0.62)	-0.66 (-1.86; 0.55)	**Upper body strength**

Health-Related Quality of Life

**NMES**	.	.	.	0.18 (-0.44; 0.81)	.
-0.31 (-1.18; 0.56)	**Home-based Multidomain**	.	0.70 (0.02; 1.38)	0.26 (-0.41; 0.93)	.
0.03 (-0.74; 0.81)	0.35 (-0.40; 1.09)	**LE-strength**	-0.12 (-0.83; 0.59)	0.21 (-0.28; 0.69)	0.27 (-0.72; 1.25)
0.14 (-0.59; 0.86)	0.45 (-0.17; 1.06)	0.10 (-0.42; 0.63)	**Multidomain exercise**	0.02 (-0.36; 0.39)	.
0.18 (-0.44; 0.81)	0.50 (-0.11; 1.11)	0.15 (-0.31; 0.61)	0.05 (-0.32; 0.42)	**Standard care**	.
0.30 (-0.95; 1.55)	0.61 (-0.62; 1.84)	0.27 (-0.72; 1.25)	0.16 (-0.95; 1.28)	0.11 (-0.97; 1.20)	**Upper body strength**

League tables presenting standardized mean differences (SMDs) with 95% confidence intervals (CIs) for Physical function, Quadriceps strength, Pain, and Health-related quality of life across all prehabilitation modality comparisons. The upper triangle displays direct treatment effects, whereas the lower triangle presents indirect network estimates. Interpretation differs between triangles: in the upper triangle, positive SMDs favor the row-defined treatment and negative SMDs favor the column-defined treatment; in the lower triangle, positive SMDs favor the column-defined treatment and negative SMDs favor the row-defined treatment. Statistically significant comparisons ($p < 0.05$) are highlighted in red. LE, lower extremity; NMES, neuromuscular electrical stimulation.

S26A. Local Inconsistency by Treatment Comparison (4–8 Weeks)

Physical Function

Comparison	Number of Studies	Direct Evidence	I2	SMD	95% CI
**Upper−body strength vs Standard care**					
Direct estimate	0	0			
Indirect estimate				0.24	[−1.02; 1.51]
Network estimate				0.24	[−1.02; 1.51]
95% prediction interval				0.24	[−1.58; 2.07]
**Multidomain exercise vs Standard care**					
Direct estimate	4	0.95	53.3%	0.21	[−0.25; 0.68]
Indirect estimate				1.77	[−0.31; 3.86]
Network estimate				0.29	[−0.16; 0.74]
95% prediction interval				0.29	[−0.78; 1.36]
**NMES vs Standard care**					
Direct estimate	2	1.00	59.8%	0.29	[−0.49; 1.07]
Indirect estimate					
Network estimate				0.29	[−0.49; 1.07]
95% prediction interval				0.29	[−1.04; 1.62]
**Home−based Multidomain vs Standard care**					
Direct estimate	1	0.81		0.01	[−0.90; 0.92]
Indirect estimate				1.68	[−0.22; 3.57]
Network estimate				0.32	[−0.50; 1.14]
95% prediction interval				0.32	[−1.05; 1.69]
**Lower−extremity strength vs Standard care**					
Direct estimate	3	0.93	66.0%	0.35	[−0.18; 0.88]
Indirect estimate				−0.04	[−1.95; 1.87]
Network estimate				0.32	[−0.19; 0.83]
95% prediction interval				0.32	[−0.79; 1.43]
**Education vs Standard care**					
Direct estimate	0	0			
Indirect estimate				0.39	[−0.73; 1.52]
Network estimate				0.39	[−0.73; 1.52]
95% prediction interval				0.39	[−1.28; 2.06]
**Exercise & Education vs Standard care**					
Direct estimate	0	0			
Indirect estimate				0.44	[−1.03; 1.91]
Network estimate				0.44	[−1.03; 1.91]
95% prediction interval				0.44	[−1.61; 2.49]

Random Effects Model

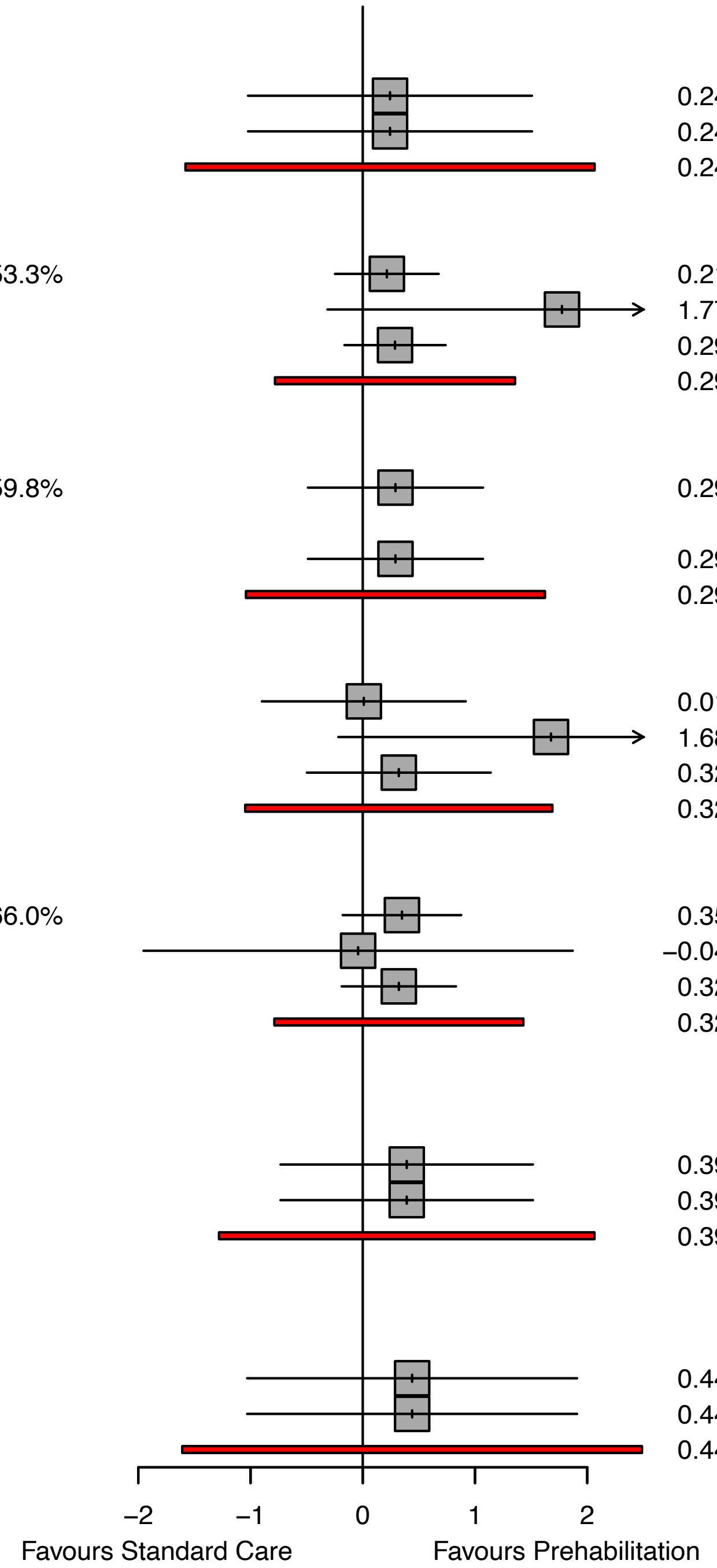

Local inconsistency assessment for physical function at 4–8 weeks after TKA. Direct, indirect, and network estimates versus standard care are presented as standardized mean differences (SMDs) with 95% confidence intervals (CIs). Agreement between the direct and indirect estimates indicates little evidence of local inconsistency. Red horizontal lines indicate the 95% prediction intervals. Positive SMDs favor the prehabilitation modality, whereas negative SMDs favor standard care. NMES, neuromuscular electrical stimulation.

S26B. Local Inconsistency by Treatment Comparison (4–8 Weeks)

Quadriceps Strength

Comparison	Number of Studies	Direct Evidence	I2	SMD	95% CI
**Multidomain exercise vs Standard care**					
Direct estimate	3	0.94	0.0%	−0.23	[−0.60; 0.14]
Indirect estimate				0.40	[−1.02; 1.83]
Network estimate				−0.19	[−0.54; 0.17]
95% prediction interval				−0.19	[−0.77; 0.40]
**Home−based Multidomain vs Standard care**					
Direct estimate	1	0.83		−0.02	[−0.64; 0.60]
Indirect estimate				0.28	[−1.11; 1.68]
Network estimate				0.03	[−0.54; 0.60]
95% prediction interval				0.03	[−0.77; 0.83]
**NMES vs Standard care**					
Direct estimate	2	1.00	0.0%	0.06	[−0.53; 0.65]
Indirect estimate					
Network estimate				0.06	[−0.53; 0.65]
95% prediction interval				0.06	[−0.77; 0.89]
**Upper−body strength vs Standard care**					
Direct estimate	0	0			
Indirect estimate				0.41	[−0.60; 1.42]
Network estimate				0.41	[−0.60; 1.42]
95% prediction interval				0.41	[−0.90; 1.72]
**Lower−extremity strength vs Standard care**					
Direct estimate	4	0.94	39.8%	0.54	[0.20; 0.89]
Indirect estimate				−0.23	[−1.64; 1.18]
Network estimate				0.50	[0.16; 0.83]
95% prediction interval				0.50	[−0.07; 1.06]

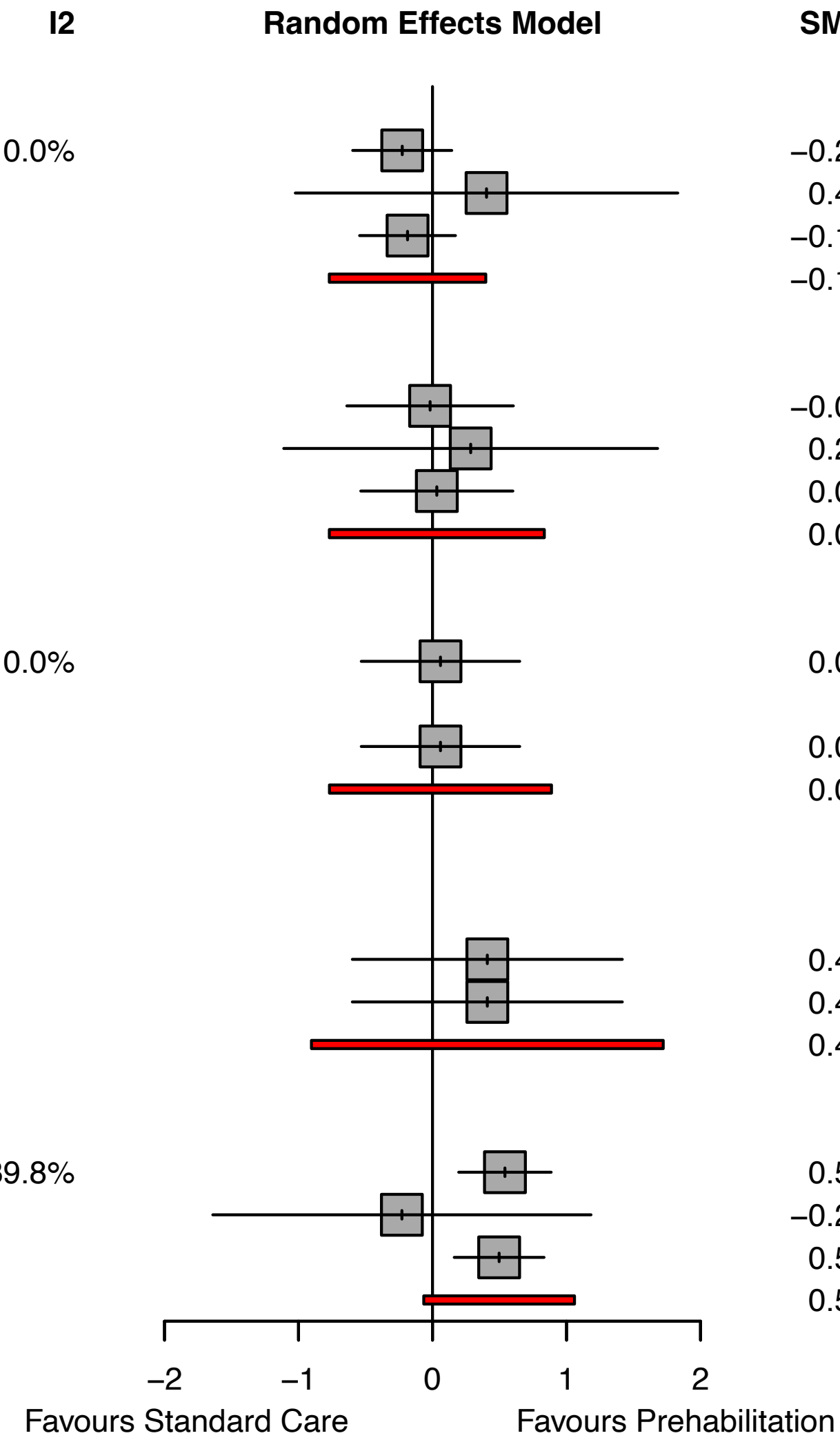

Local inconsistency assessment for quadriceps strength at 4–8 weeks after TKA. Direct, indirect, and network estimates versus standard care are presented as standardized mean differences (SMDs) with 95% confidence intervals (CIs). Agreement between the direct and indirect estimates indicates little evidence of local inconsistency. Red horizontal lines indicate the 95% prediction intervals. Positive SMDs favor the prehabilitation modality, whereas negative SMDs favor standard care. NMES, neuromuscular electrical stimulation.

S26C. Local Inconsistency by Treatment Comparison (4–8 Weeks)

Pain

Comparison	Number of Studies	Direct Evidence	I2	SMD	95% CI
**Exercise & Education vs Standard care**					
Direct estimate	0	0			
Indirect estimate				−0.40	[−1.78; 0.98]
Network estimate				−0.40	[−1.78; 0.98]
95% prediction interval				−0.40	[−2.30; 1.50]
**Education vs Standard care**					
Direct estimate	0	0			
Indirect estimate				−0.08	[−1.14; 0.98]
Network estimate				−0.08	[−1.14; 0.98]
95% prediction interval				−0.08	[−1.63; 1.47]
**Multidomain exercise vs Standard care**					
Direct estimate	4	0.95	46.9%	−0.02	[−0.45; 0.41]
Indirect estimate				0.88	[−1.06; 2.83]
Network estimate				0.02	[−0.39; 0.44]
95% prediction interval				0.02	[−0.94; 0.98]
**NMES vs Standard care**					
Direct estimate	2	1.00	43.4%	0.09	[−0.64; 0.83]
Indirect estimate					
Network estimate				0.09	[−0.64; 0.83]
95% prediction interval				0.09	[−1.13; 1.31]
**Home−based Multidomain vs Standard care**					
Direct estimate	1	0.81		0.07	[−0.77; 0.91]
Indirect estimate				0.62	[−1.14; 2.38]
Network estimate				0.17	[−0.59; 0.93]
95% prediction interval				0.17	[−1.07; 1.42]
**Lower−extremity strength vs Standard care**					
Direct estimate	3	0.93	68.7%	0.47	[−0.02; 0.96]
Indirect estimate				0.55	[−1.23; 2.32]
Network estimate				0.48	[0.00; 0.95]
95% prediction interval				0.48	[−0.52; 1.48]
**Upper−body strength vs Standard care**					
Direct estimate	0	0			
Indirect estimate				0.66	[−0.55; 1.86]
Network estimate				0.66	[−0.55; 1.86]
95% prediction interval				0.66	[−1.05; 2.37]

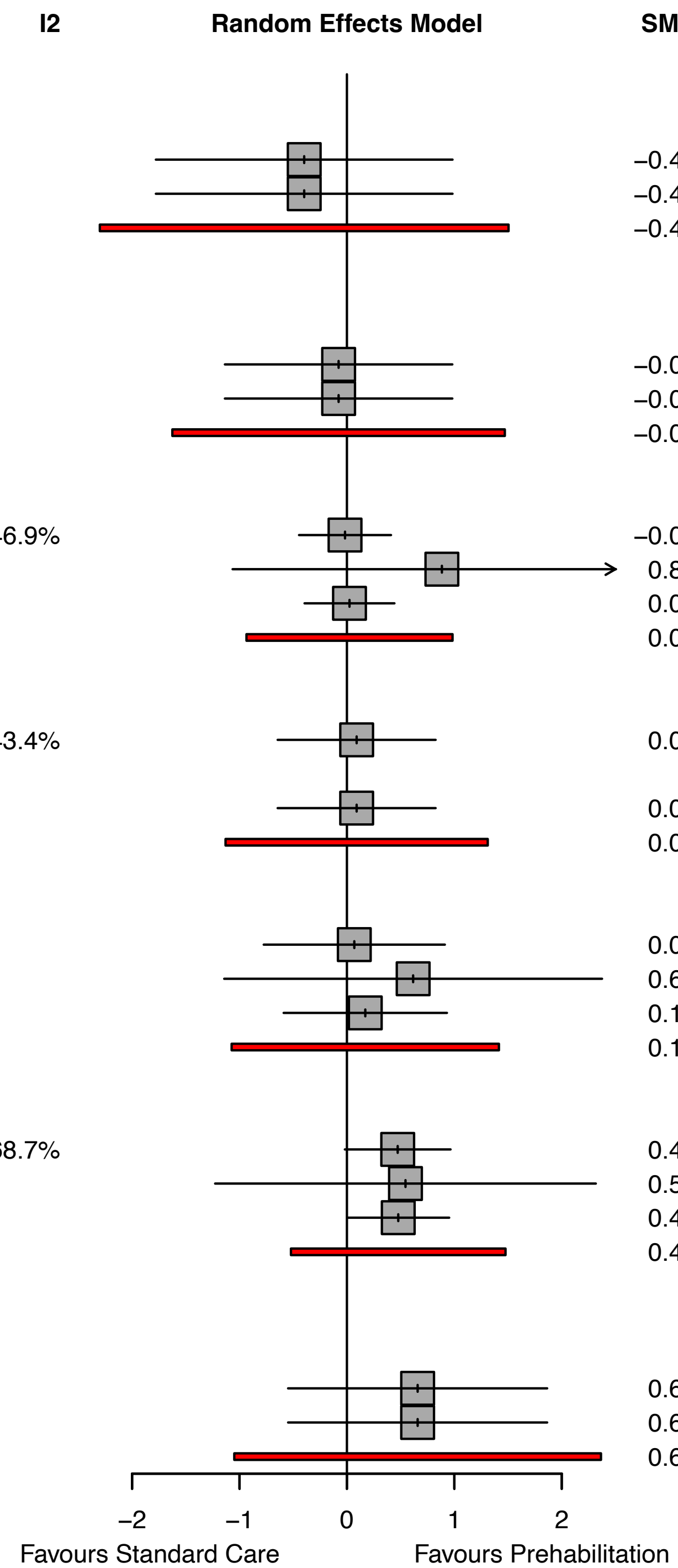

Local inconsistency assessment for pain at 4–8 weeks after TKA. Direct, indirect, and network estimates versus standard care are presented as standardized mean differences (SMDs) with 95% confidence intervals (CIs). Agreement between the direct and indirect estimates indicates little evidence of local inconsistency. Red horizontal lines indicate the 95% prediction intervals. Positive SMDs favor the prehabilitation modality, whereas negative SMDs favor standard care. NMES, neuromuscular electrical stimulation.

S26D. Local Inconsistency by Treatment Comparison (4–8 Weeks)

Health-Related Quality of Life

Comparison	Number of Studies	Direct Evidence	I2	SMD	95% CI
**Upper−body strength vs Standard care**					
Direct estimate	0	0			
Indirect estimate				−0.11	[−1.20; 0.97]
Network estimate				−0.11	[−1.20; 0.97]
95% prediction interval				−0.11	[−1.75; 1.52]
**Multidomain exercise vs Standard care**					
Direct estimate	3	0.96	50.4%	0.02	[−0.36; 0.39]
Indirect estimate				0.79	[−1.05; 2.64]
Network estimate				0.05	[−0.32; 0.42]
95% prediction interval				0.05	[−0.71; 0.81]
**Lower−extremity strength vs Standard care**					
Direct estimate	2	0.90	0.0%	0.21	[−0.28; 0.69]
Indirect estimate				−0.33	[−1.79; 1.13]
Network estimate				0.15	[−0.31; 0.61]
95% prediction interval				0.15	[−0.70; 1.01]
**NMES vs Standard care**					
Direct estimate	2	1.00	0.0%	0.18	[−0.44; 0.81]
Indirect estimate					
Network estimate				0.18	[−0.44; 0.81]
95% prediction interval				0.18	[−0.85; 1.22]
**Home−based Multidomain vs Standard care**					
Direct estimate	1	0.83		0.26	[−0.41; 0.93]
Indirect estimate				1.68	[0.18; 3.18]
Network estimate				0.50	[−0.11; 1.11]
95% prediction interval				0.50	[−0.53; 1.52]

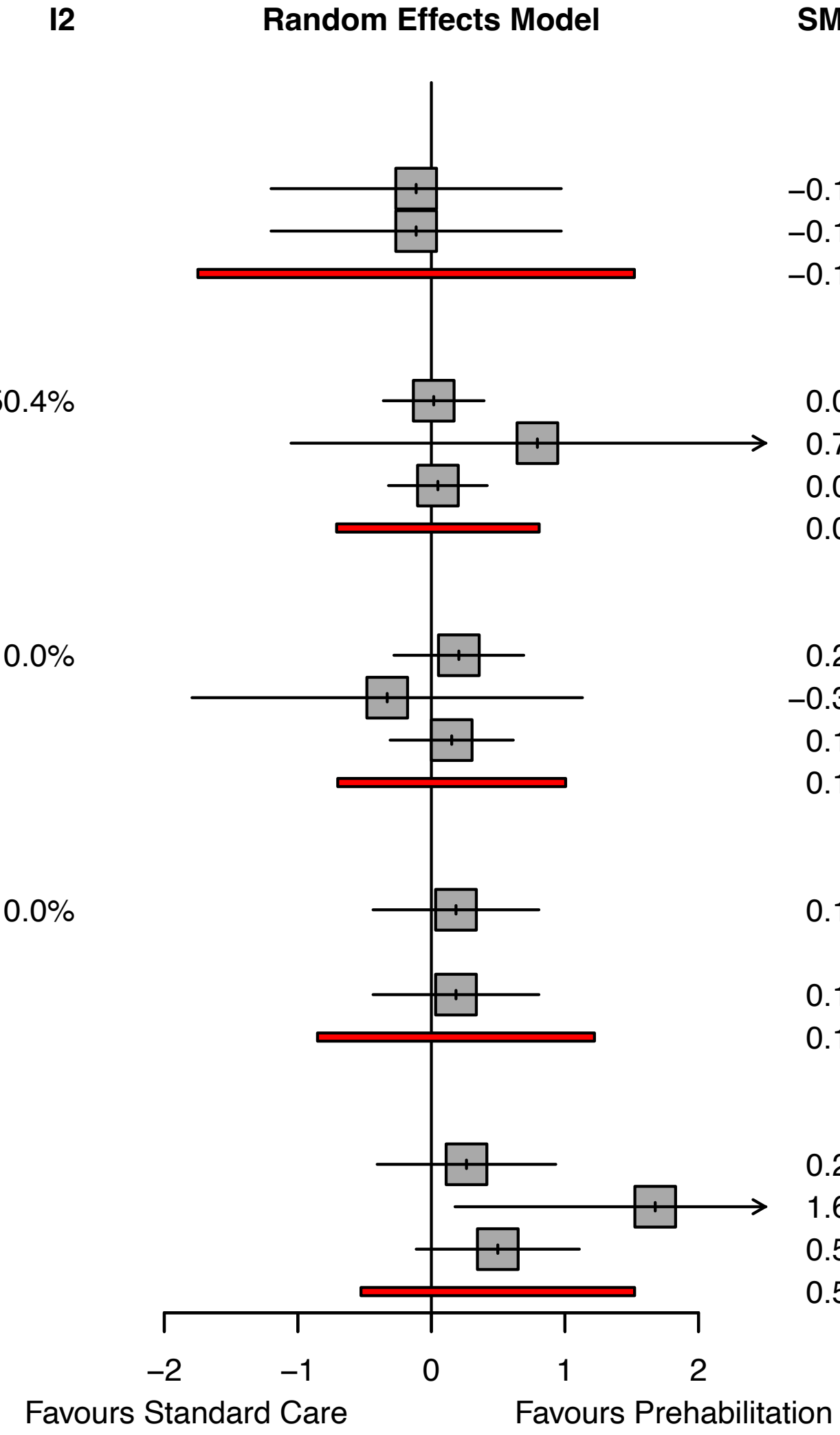

Local inconsistency assessment for health-related quality of life at 4–8 weeks after TKA. Direct, indirect, and network estimates versus standard care are presented as standardized mean differences (SMDs) with 95% confidence intervals (CIs). Agreement between the direct and indirect estimates indicates little evidence of local inconsistency. Red horizontal lines indicate the 95% prediction intervals. Positive SMDs favor the prehabilitation modality, whereas negative SMDs favor standard care. NMES, neuromuscular electrical stimulation.

S27A. Sensitivity Analysis: Excluding High Risk of Bias Studies (4–8 Weeks)

Physical Function

Treatment	High–RoB–trials excluded	SMD	95% CI
**Education**			
High–RoB–studies excluded		0.62	[−0.35; 1.58]
All studies		0.39	[−0.73; 1.52]
**Exercise & Education**			
High–RoB–studies excluded		0.66	[−0.55; 1.88]
All studies		0.44	[−1.03; 1.91]
**Lower–extremity strength**			
High–RoB–studies excluded		0.43	[−0.26; 1.12]
All studies		0.32	[−0.19; 0.83]
**Multidomain exercise**			
High–RoB–studies excluded		0.51	[0.03; 0.99]
All studies		0.29	[−0.16; 0.74]
**NMES**			
High–RoB–studies excluded		0.35	[−0.29; 0.99]
All studies		0.29	[−0.49; 1.07]
**Upper–body strength**			
High–RoB–studies excluded		0.35	[−0.86; 1.56]
All studies		0.24	[−1.02; 1.51]
**Home–based Multidomain**			
All studies		0.32	[−0.50; 1.14]

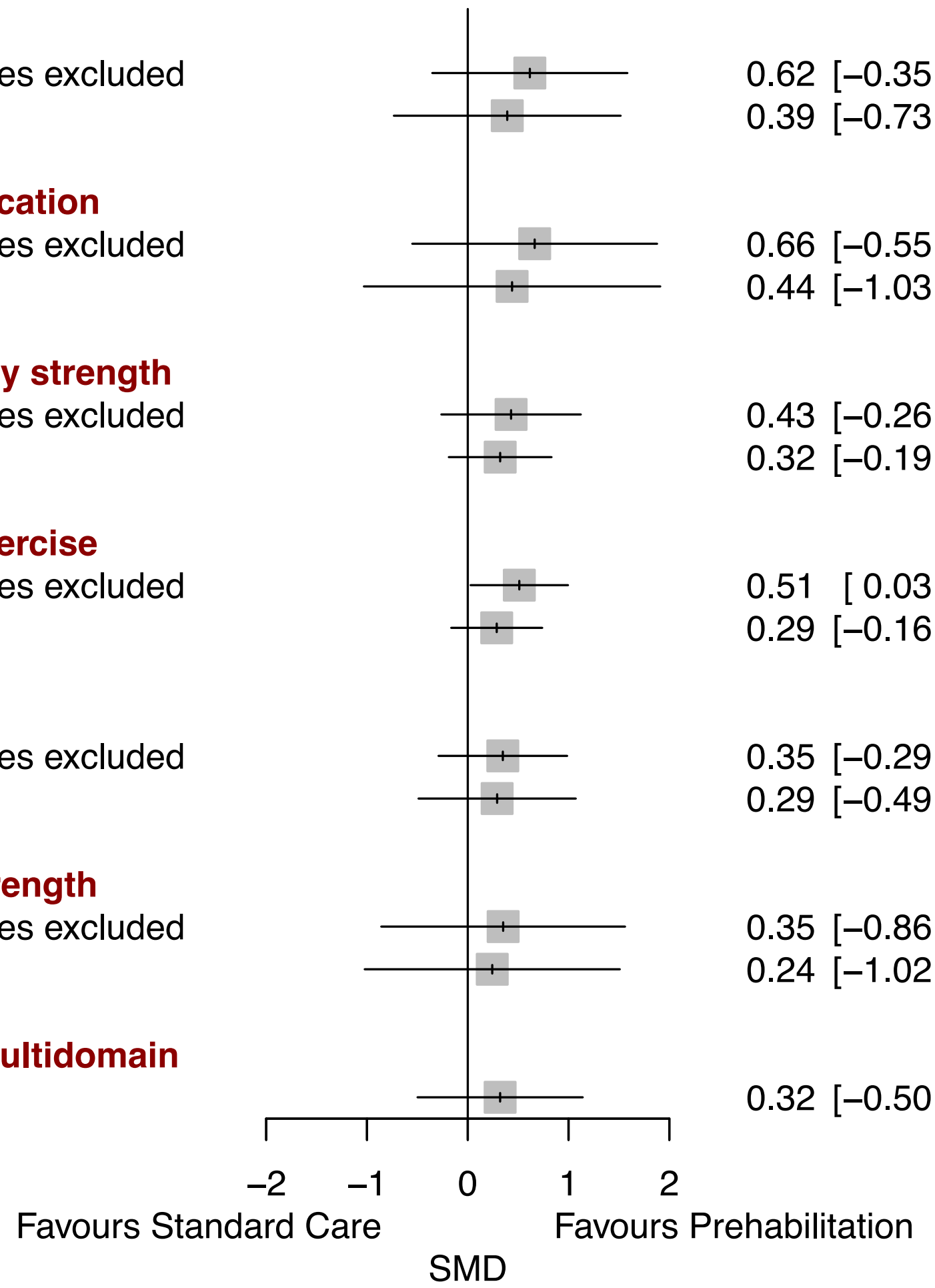

Sensitivity analysis for physical function after excluding studies at high risk of bias (Blasco et al. 2020; Dominguez-Navarro et al. 2021; Tungtrongjit et al. 2012). Results are presented as standardized mean differences (SMDs) with 95% confidence intervals (CIs). Positive SMDs favor the prehabilitation modality, whereas negative SMDs favor standard care. NMES, neuromuscular electrical stimulation.

S27B. Sensitivity Analysis: Excluding High Risk of Bias Studies (4–8 Weeks)

Quadriceps Strength

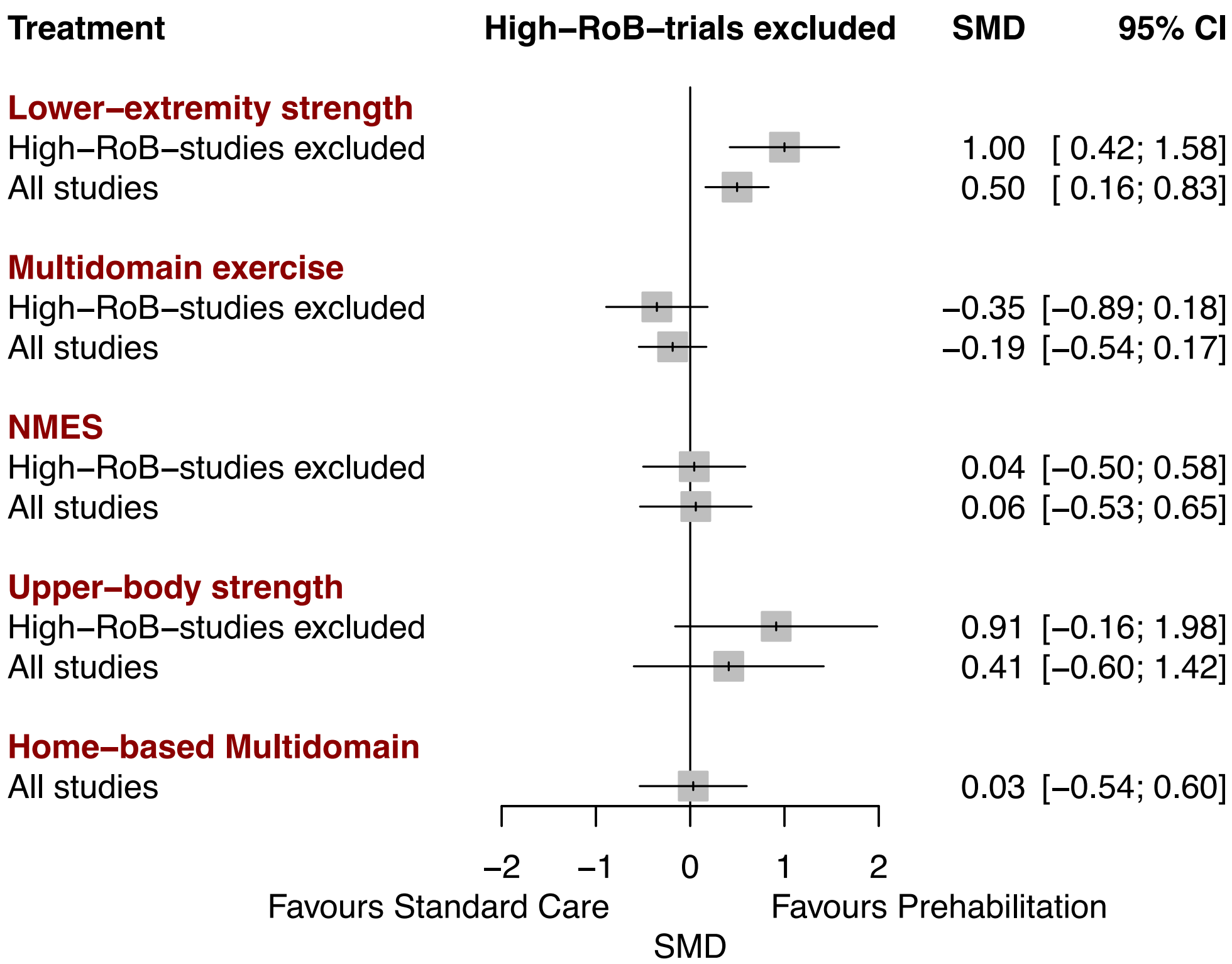

Sensitivity analysis for quadriceps strength after excluding studies at high risk of bias (Blasco et al. 2020; Dominguez-Navarro et al. 2021; Tungtrongjit et al. 2012; Van Leeuwen et al. 2014). Results are presented as standardized mean differences (SMDs) with 95% confidence intervals (CIs). Positive SMDs favor the prehabilitation modality, whereas negative SMDs favor standard care. BFR, blood flow restriction; NMES, neuromuscular electrical stimulation.

S27C. Sensitivity Analysis: Excluding High Risk of Bias Studies (4–8 Weeks)

Pain

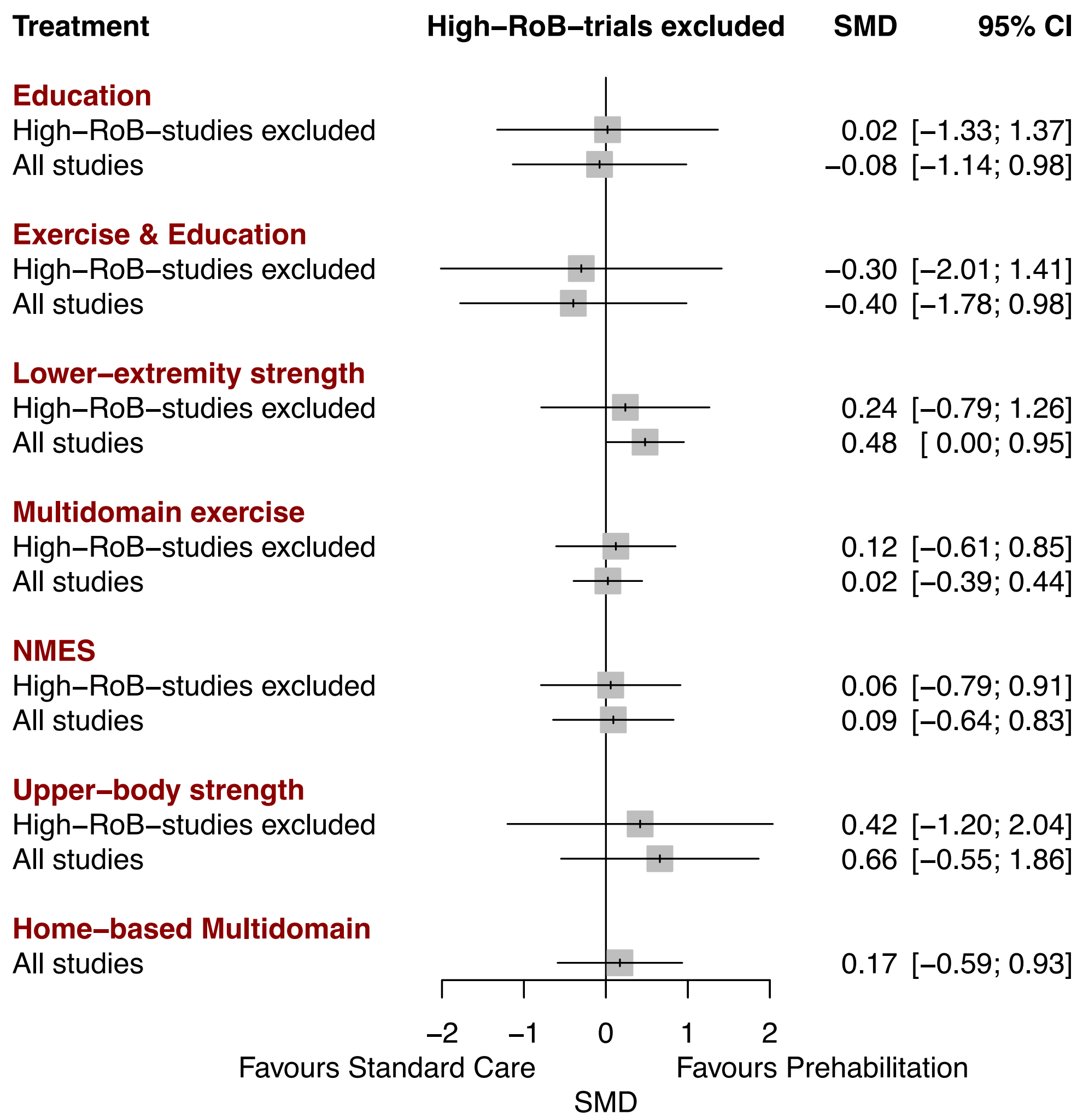

Sensitivity analysis for pain after excluding studies at high risk of bias (Blasco et al. 2020; Dominguez-Navarro et al. 2021; Tungtrongjit et al. 2012). Results are presented as standardized mean differences (SMDs) with 95% confidence intervals (CIs). Positive SMDs favor the prehabilitation modality, whereas negative SMDs favor standard care. NMES, neuromuscular electrical stimulation.

S27D. Sensitivity Analysis: Excluding High Risk of Bias Studies (4–8 Weeks)

Health-related quality of life

Treatment	SMD	95% CI
**Lower–extremity strength**		
High–RoB–studies excluded	0.35	[−0.20; 0.91]
All studies	0.15	[−0.31; 0.61]
**Multidomain exercise**		
High–RoB–studies excluded	0.27	[−0.17; 0.70]
All studies	0.05	[−0.32; 0.42]
**NMES**		
High–RoB–studies excluded	0.21	[−0.34; 0.75]
All studies	0.18	[−0.44; 0.81]
**Upper–body strength**		
High–RoB–studies excluded	0.09	[−0.97; 1.15]
All studies	−0.11	[−1.20; 0.97]
**Home–based Multidomain**		
All studies	0.50	[−0.11; 1.11]

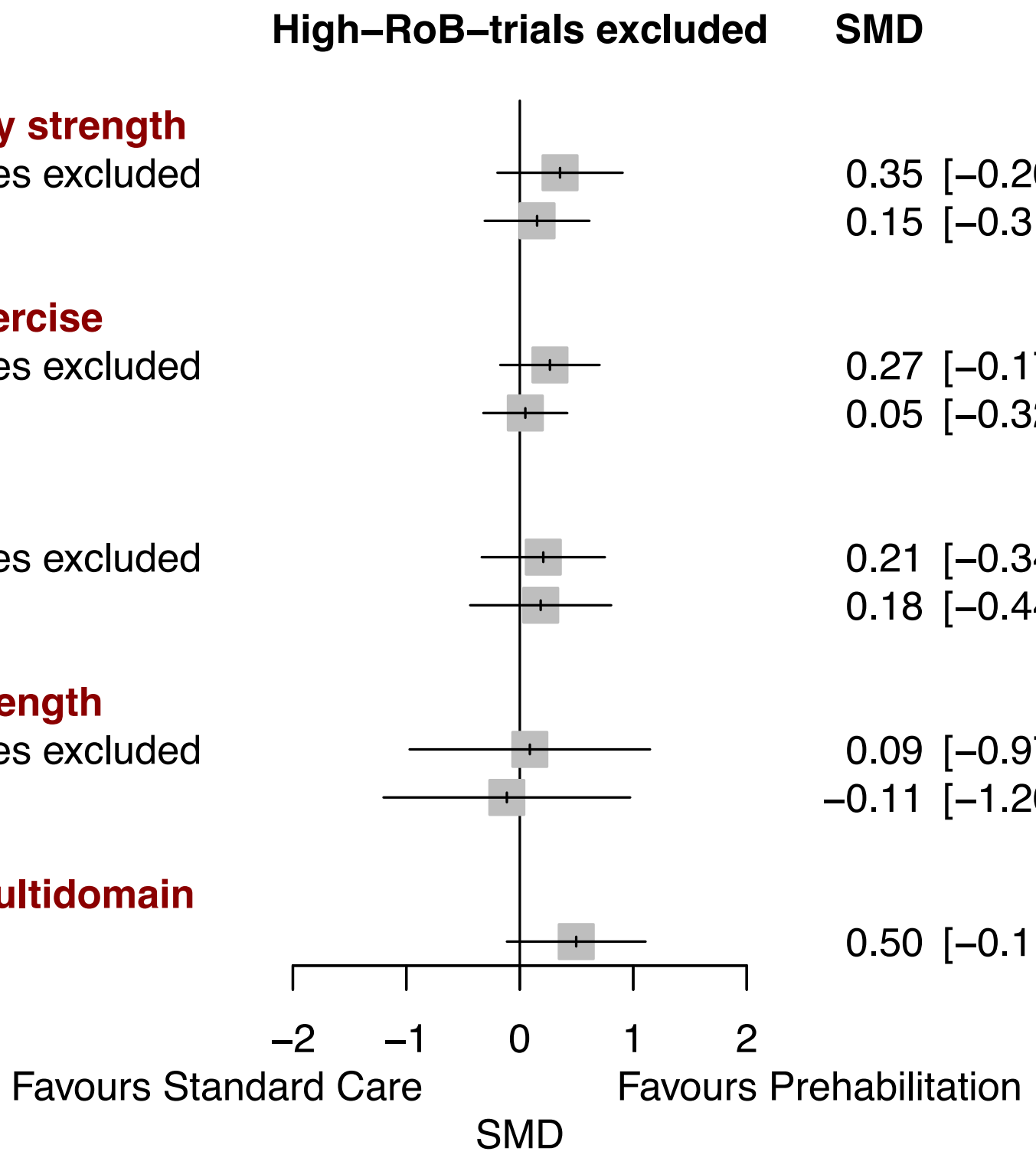

Sensitivity analysis for health-related quality of life after excluding studies at high risk of bias (Blasco et al. 2020; Dominguez-Navarro et al. 2021). Results are presented as standardized mean differences (SMDs) with 95% confidence intervals (CIs). Positive SMDs favor the prehabilitation modality, whereas negative SMDs favor standard care. NMES, neuro-muscular electrical stimulation.

S28A. Sensitivity Analysis: Excluding Change Score Studies (4–8 Weeks)

Physical Function

Treatment	Change Score Trials Excluded	SMD	95% CI
**Education**			
Change Score Studies Excluded		0.34	[−0.93; 1.60]
All studies		0.39	[−0.73; 1.52]
**Exercise & Education**			
Change Score Studies Excluded		0.39	[−1.26; 2.03]
All studies		0.44	[−1.03; 1.91]
**Home−based Multidomain**			
Change Score Studies Excluded		0.29	[−0.63; 1.22]
All studies		0.32	[−0.50; 1.14]
**Lower−extremity strength**			
Change Score Studies Excluded		0.31	[−0.26; 0.88]
All studies		0.32	[−0.19; 0.83]
**Multidomain exercise**			
Change Score Studies Excluded		0.23	[−0.35; 0.82]
All studies		0.29	[−0.16; 0.74]
**NMES**			
Change Score Studies Excluded		0.27	[−0.57; 1.12]
All studies		0.29	[−0.49; 1.07]
**Upper−body strength**			
Change Score Studies Excluded		0.23	[−1.13; 1.59]
All studies		0.24	[−1.02; 1.51]

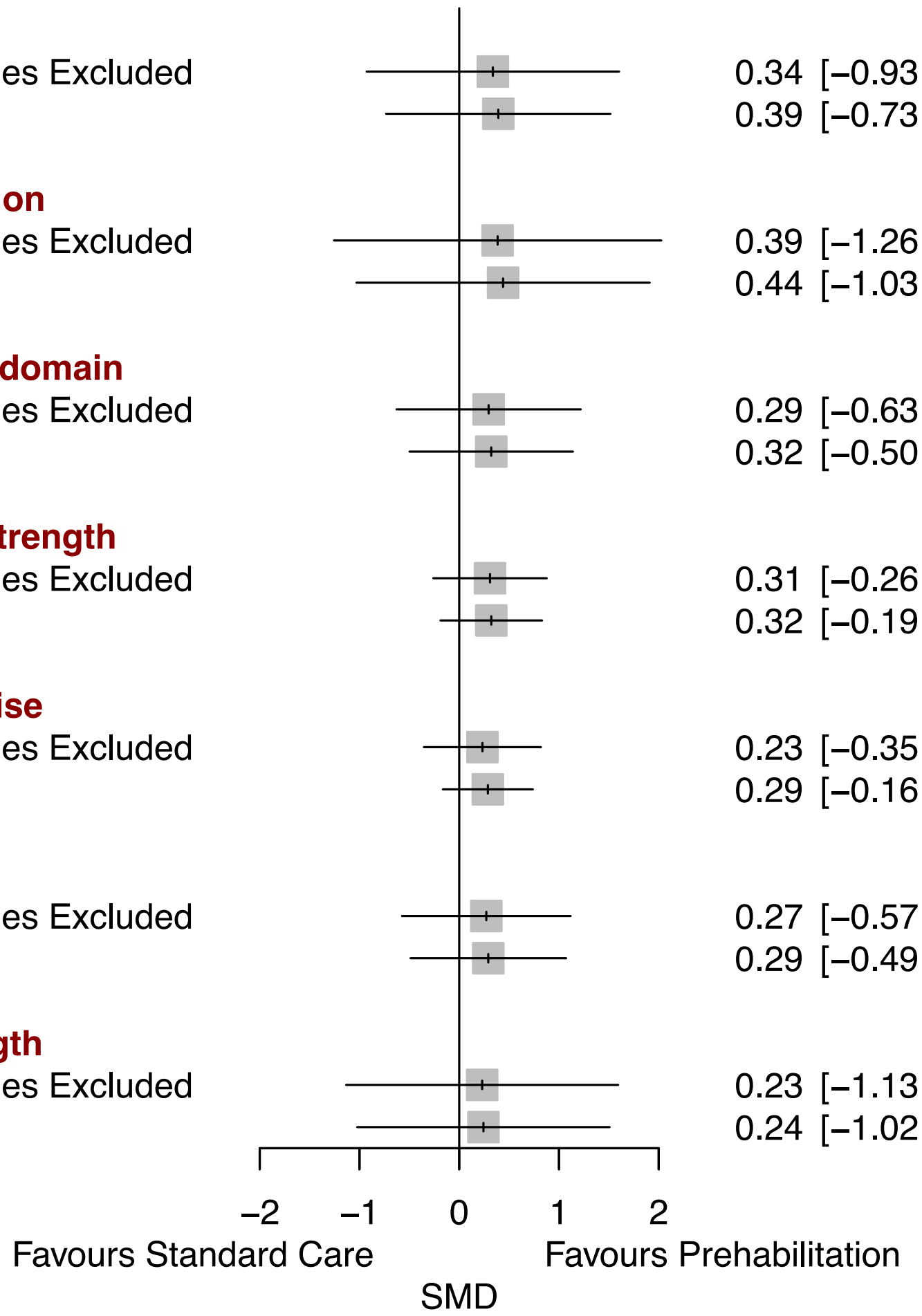

Sensitivity analysis for physical function excluding studies reporting change scores from baseline to 4–8 weeks after TKA (Villadsen et al. 2014). Results are presented as standardized mean differences (SMDs) with 95% confidence intervals (CIs). Positive SMDs favor the prehabilitation modality, whereas negative SMDs favor standard care. NMES, neuromuscular electrical stimulation.

S28B. Sensitivity Analysis: Excluding Change Score Studies (4–8 Weeks)

Quadriceps Strength

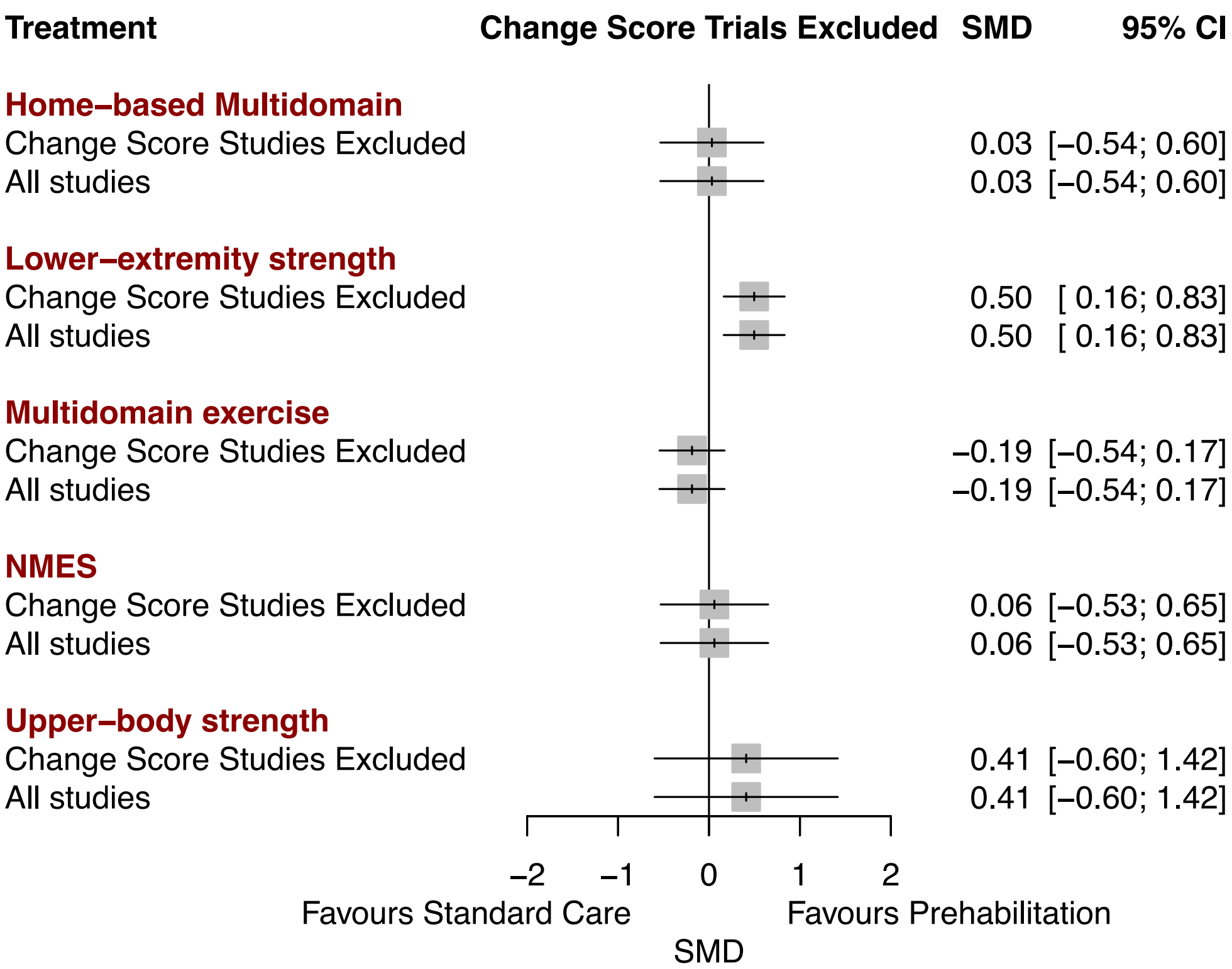

Sensitivity analysis for quadriceps strength excluding studies reporting change scores from baseline to 4–8 weeks after TKA (Huber et al. 2015; Villadsen et al. 2014). Results are presented as standardized mean differences (SMDs) with 95% confidence intervals (CIs). Positive SMDs favor the prehabilitation modality, whereas negative SMDs favor standard care. NMES, neuromuscular electrical stimulation.

S28C. Sensitivity Analysis: Excluding Change Score Studies (4–8 Weeks)

Pain

Treatment	Change Score Trials Excluded	SMD	95% CI
**Education**			
Change Score Studies Excluded		−0.25	[−1.26; 0.76]
All studies		−0.08	[−1.14; 0.98]
**Exercise & Education**			
Change Score Studies Excluded		−0.57	[−1.86; 0.72]
All studies		−0.40	[−1.78; 0.98]
**Home−based Multidomain**			
Change Score Studies Excluded		0.09	[−0.61; 0.78]
All studies		0.17	[−0.59; 0.93]
**Lower−extremity strength**			
Change Score Studies Excluded		0.45	[0.02; 0.88]
All studies		0.48	[0.00; 0.95]
**Multidomain exercise**			
Change Score Studies Excluded		−0.15	[−0.60; 0.30]
All studies		0.02	[−0.39; 0.44]
**NMES**			
Change Score Studies Excluded		0.11	[−0.57; 0.79]
All studies		0.09	[−0.64; 0.83]
**Upper−body strength**			
Change Score Studies Excluded		0.63	[−0.51; 1.76]
All studies		0.66	[−0.55; 1.86]

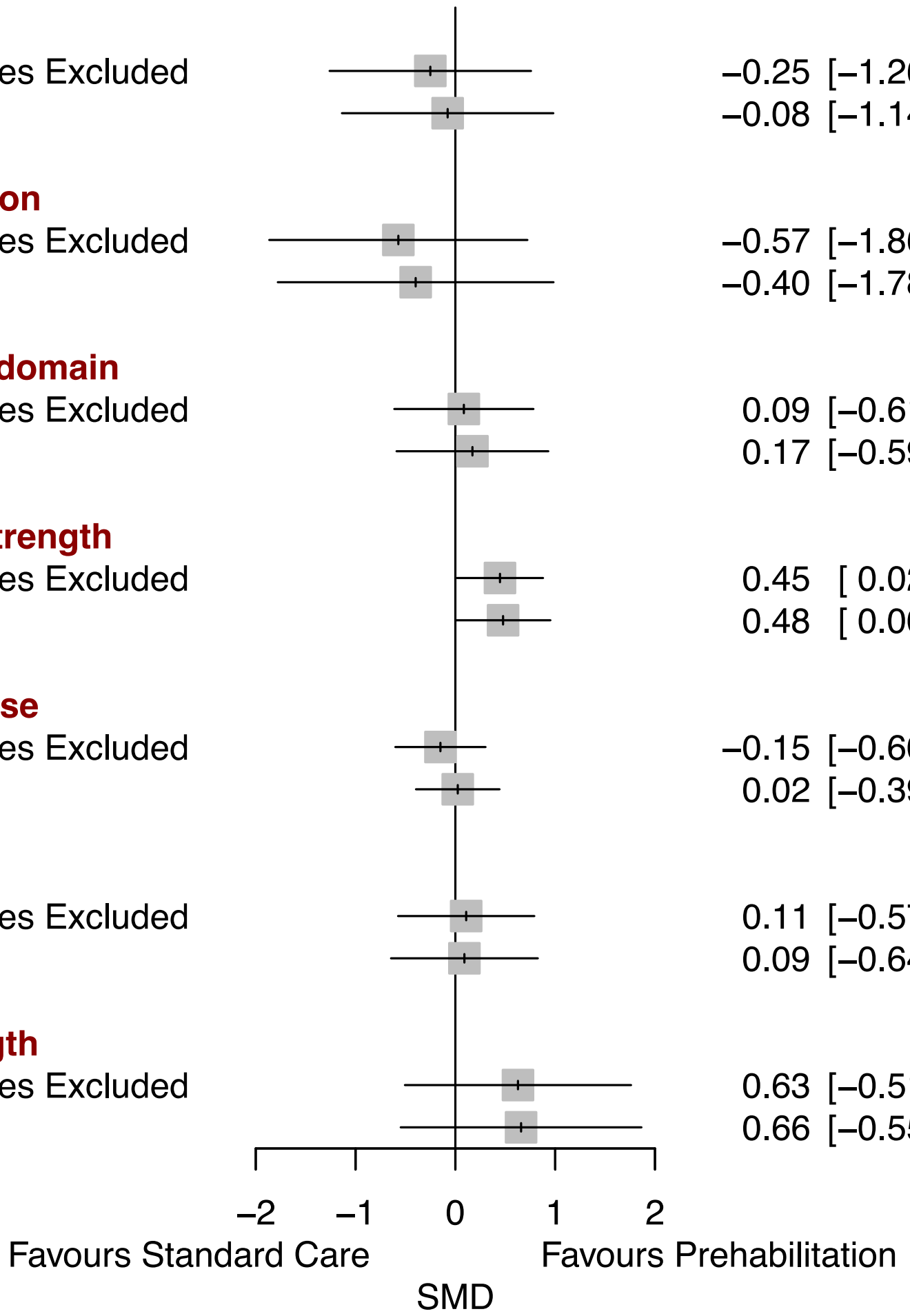

Sensitivity analysis for pain excluding studies reporting change scores from baseline to 4–8 weeks after TKA (Villadsen et al. 2014). Results are presented as standardized mean differences (SMDs) with 95% confidence intervals (CIs). Positive SMDs favor the prehabilitation modality, whereas negative SMDs favor standard care. NMES, neuromuscular electrical stimulation.

S28D. Sensitivity Analysis: Excluding Change Score Studies (4–8 Weeks)

Health-Related Quality of Life

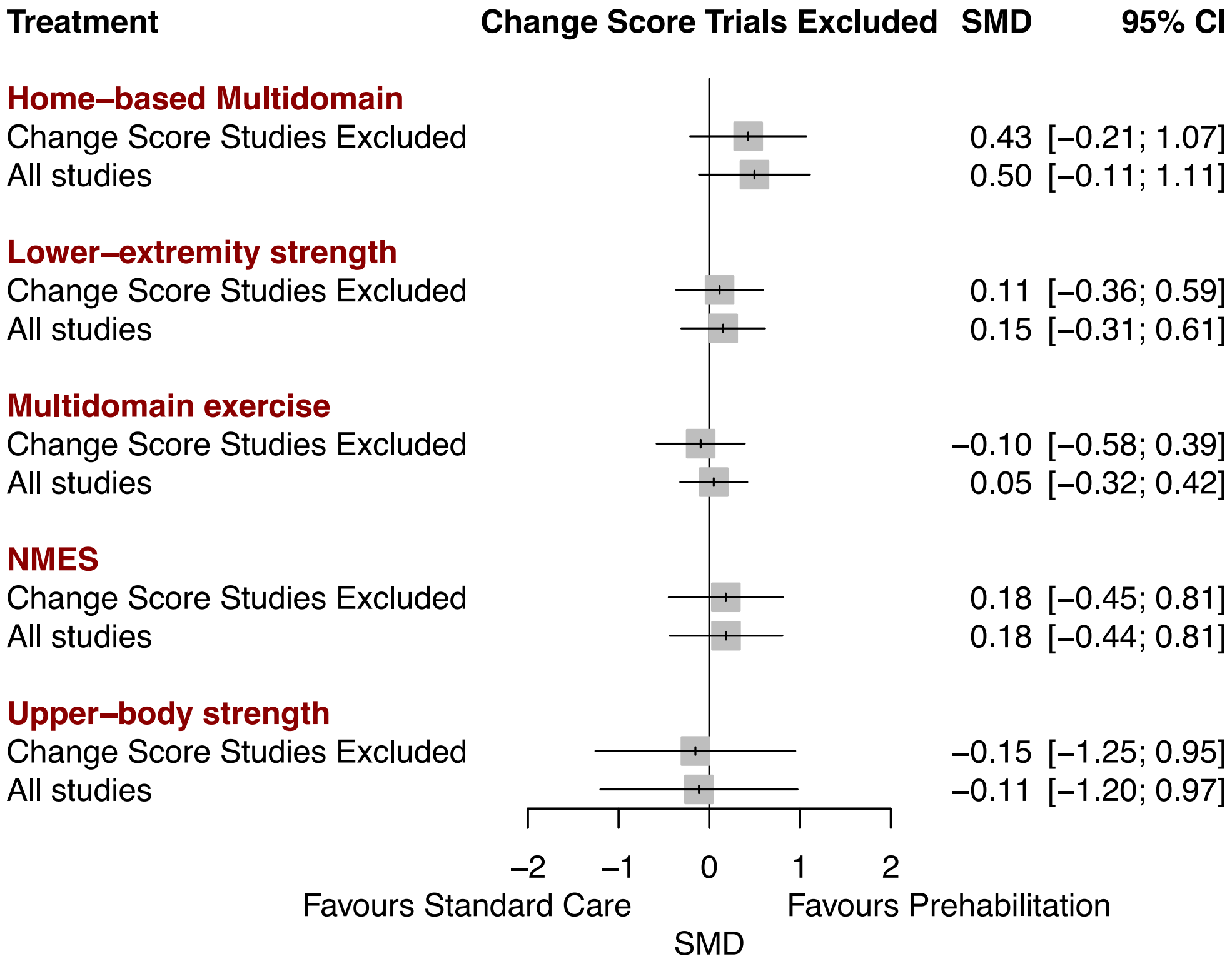

Sensitivity analysis for health-related quality of life excluding studies reporting change scores from baseline to 4–8 weeks after TKA (Huber et al. 2015; Villadsen et al. 2014). Results are presented as standardized mean differences (SMDs) with 95% confidence intervals (CIs). Positive SMDs favor the prehabilitation modality, whereas negative SMDs favor standard care. NMES, neuromuscular electrical stimulation.

S29A. Sensitivity Analysis: Including Model-Based CIs (4–8 Weeks)

Physical Function

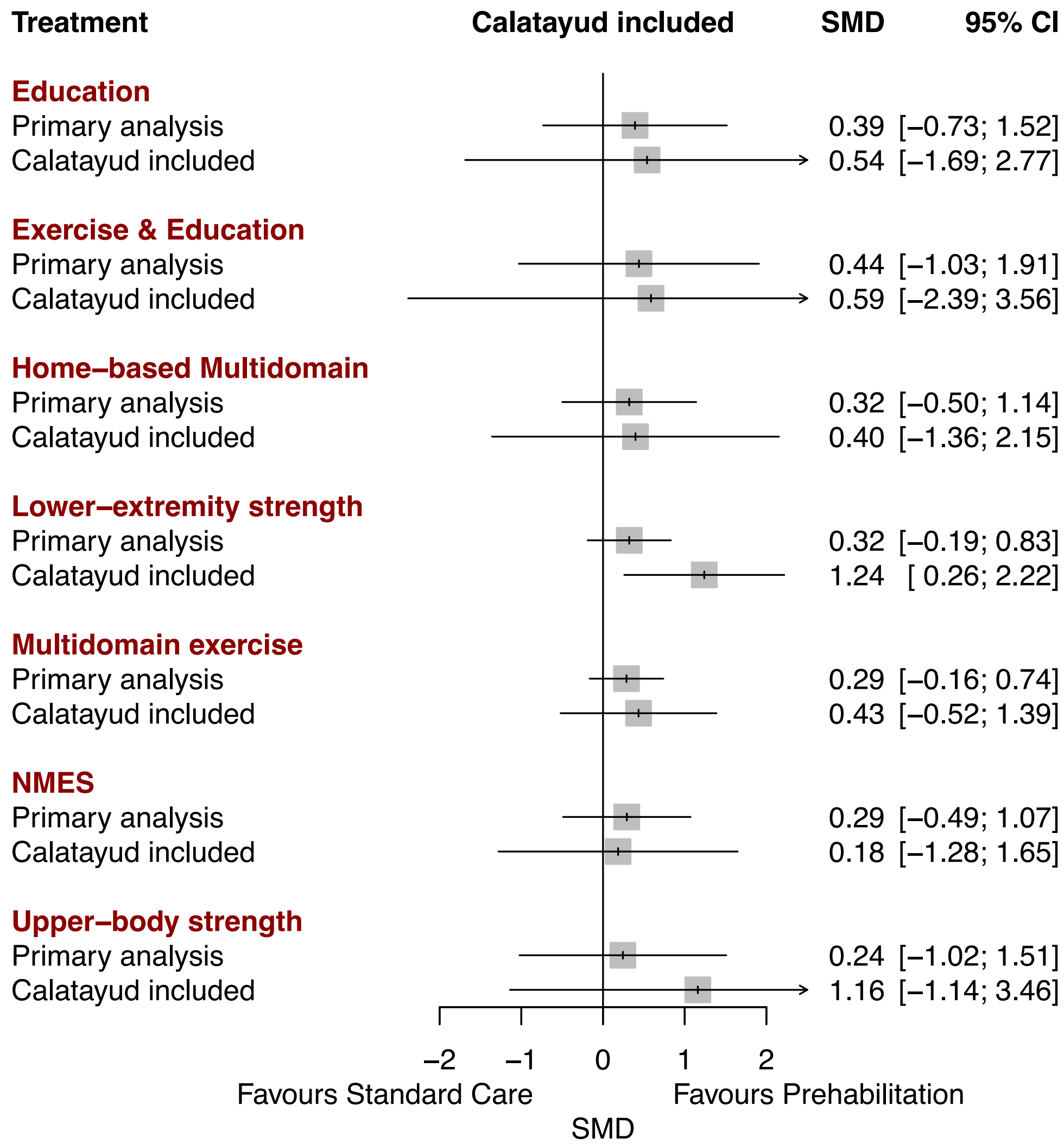

Sensitivity analysis for physical function including the previously excluded study by Calatayud et al. (2017), using SDs derived from model-based confidence intervals (CIs). Results are presented as standardized mean differences (SMDs) with 95% CIs. Positive SMDs favor the prehabilitation modality, whereas negative SMDs favor standard care. NMES, neuromuscular electrical stimulation.

S29B. Sensitivity Analysis: Including Model-Based CIs (4–8 Weeks)

Quadriceps Strength

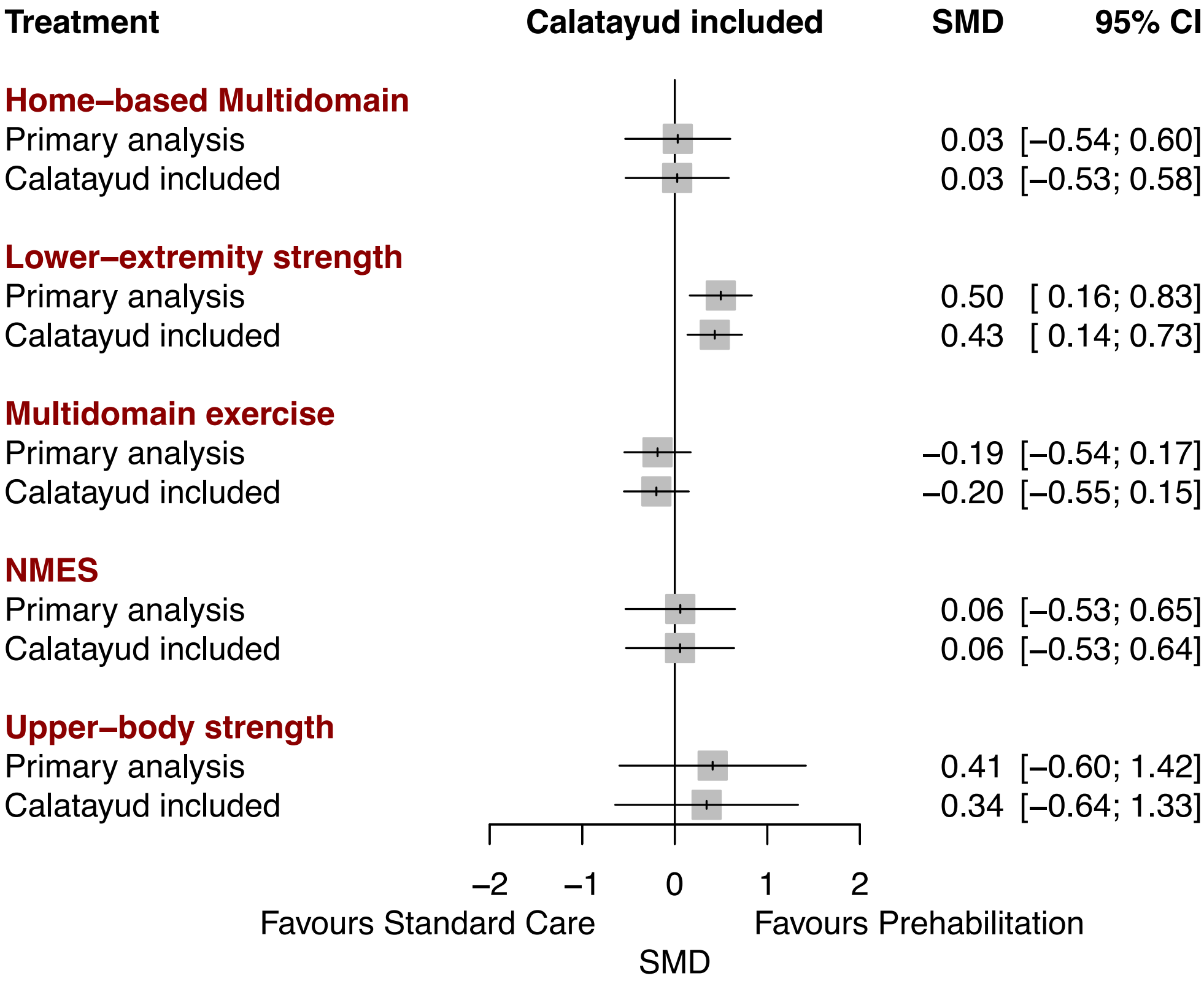

Sensitivity analysis for quadriceps strength including the previously excluded study by Calatayud et al. (2017), using SDs derived from model-based confidence intervals (CIs). Results are presented as standardized mean differences (SMDs) with 95% CIs. Positive SMDs favor the prehabilitation modality, whereas negative SMDs favor standard care. NMES, neuromuscular electrical stimulation.

S29C. Sensitivity Analysis: Including Model-Based CIs (4–8 Weeks)

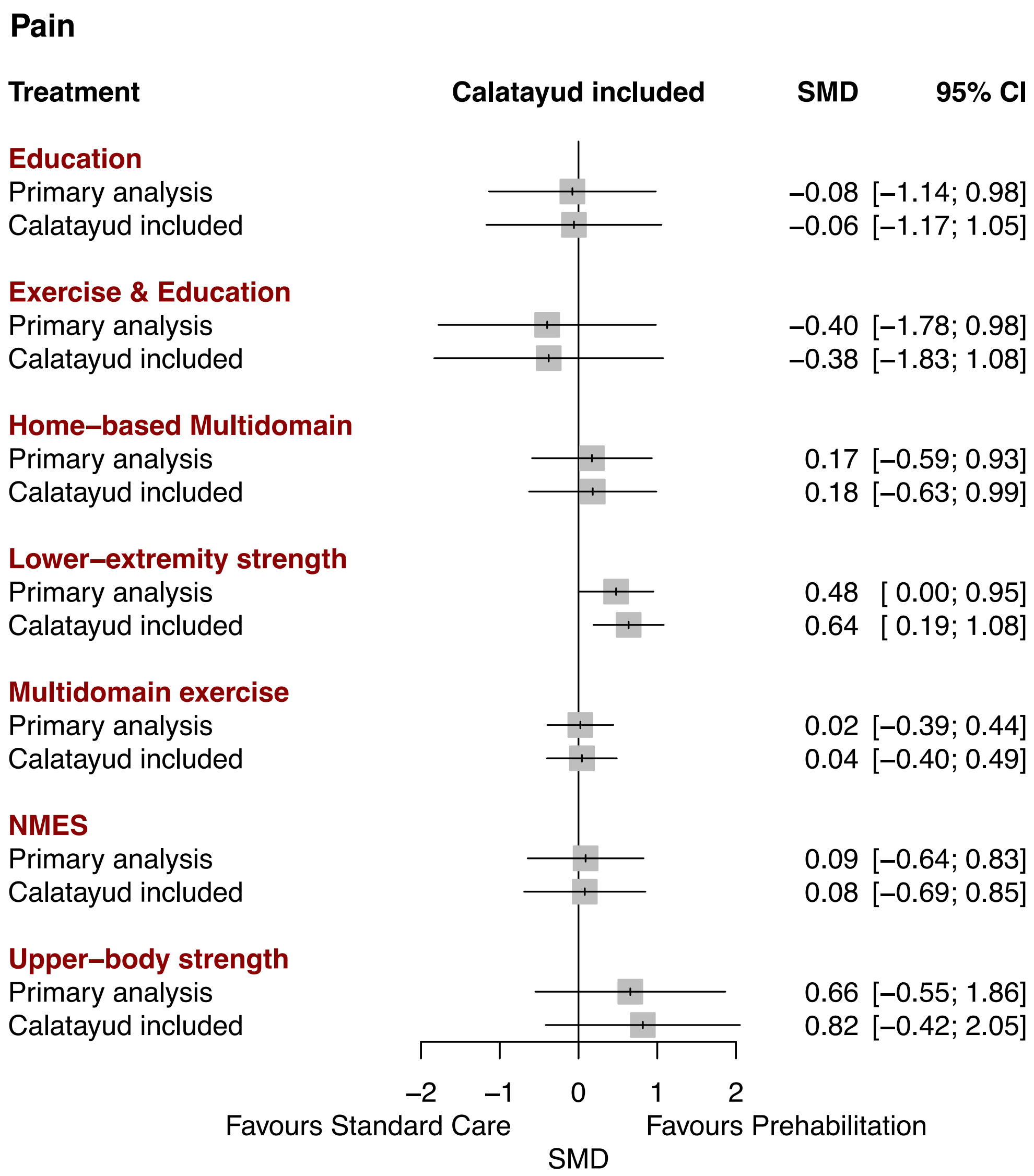

Sensitivity analysis for pain including the previously excluded study by Calatayud et al. (2017), using SDs derived from model-based confidence intervals (CIs). Results are presented as standardized mean differences (SMDs) with 95% CIs. Positive SMDs favor the prehabilitation modality, whereas negative SMDs favor standard care. NMES, neuromuscular electrical stimulation.

S30A. Sensitivity Analysis: Excluding Small Modalities (n<20) (4–8 Weeks)

Physical Function

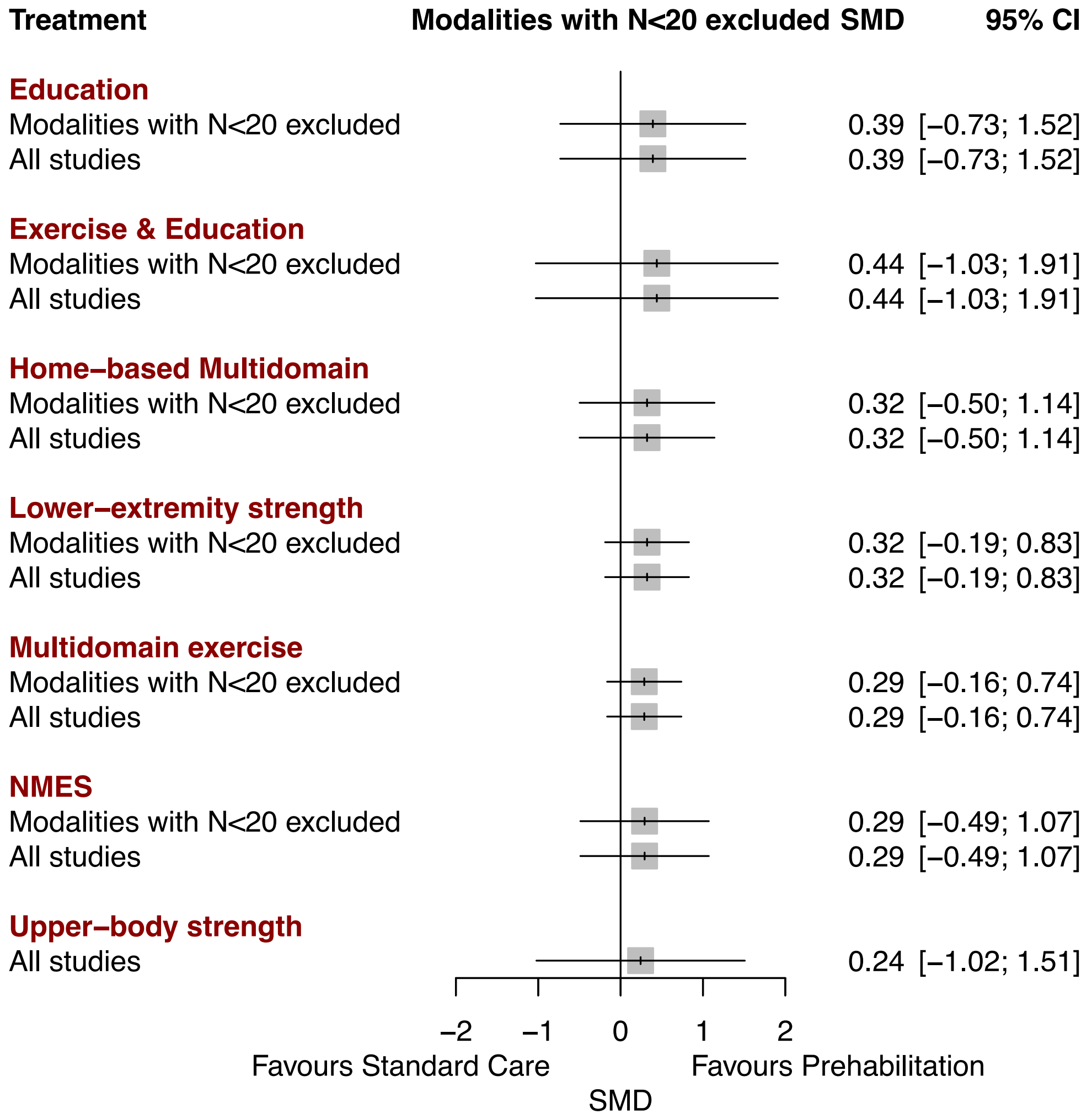

Sensitivity analysis for physical function after excluding modalities with a sample size of n<20 (aerobic exercise and upper-body strength; n = 10 each). Results are presented as standardized mean differences (SMDs) with 95% confidence intervals (CIs). Positive SMDs favor the prehabilitation modality, whereas negative SMDs favor standard care. NMES, neuromuscular electrical stimulation.

S30B. Sensitivity Analysis: Excluding Small Modalities (n<20) (4–8 Weeks)

Quadriceps Strength

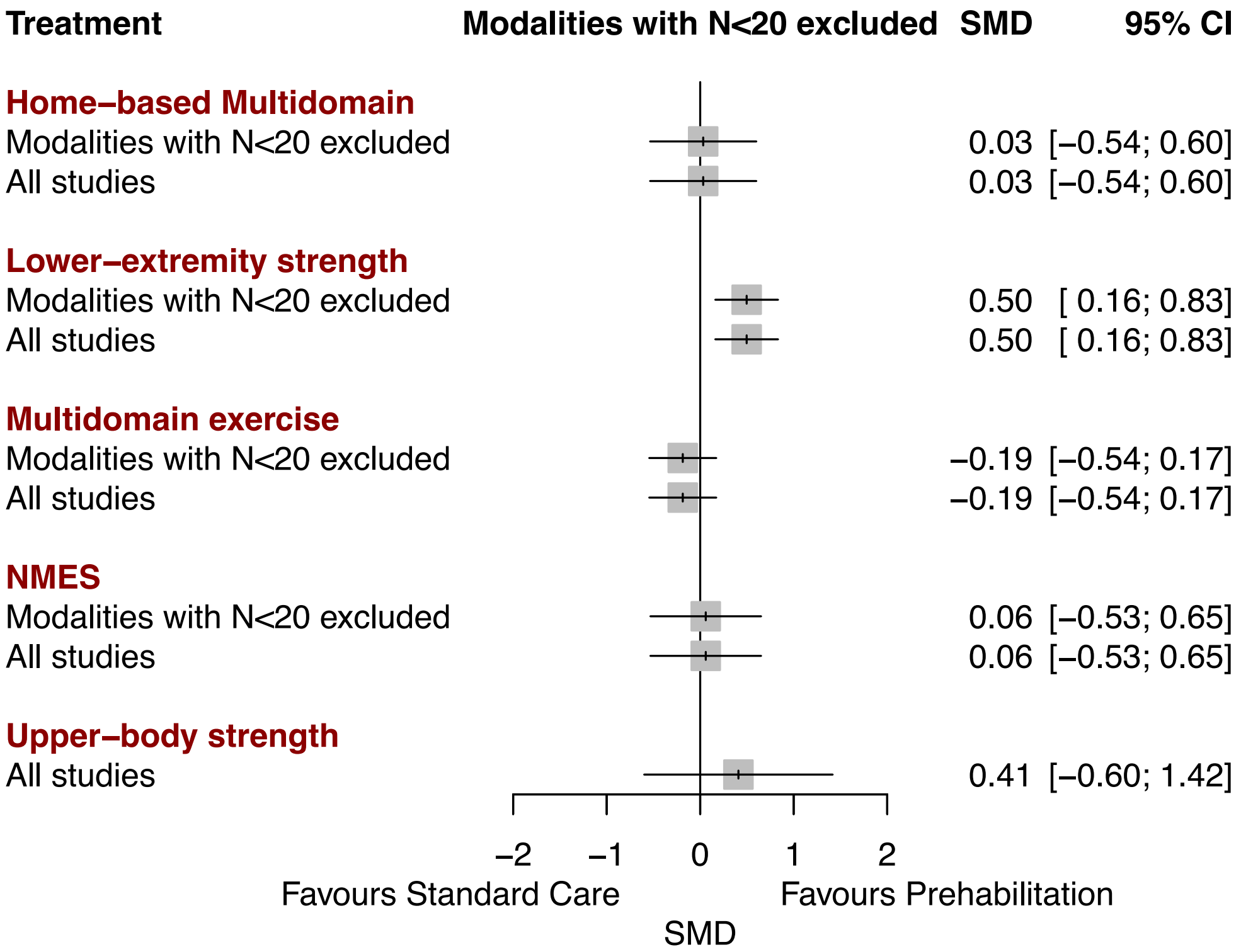

Sensitivity analysis for quadriceps strength after excluding modalities with a sample size of n<20 (aerobic exercise and upper-body strength; n = 10 each). Results are presented as standardized mean differences (SMDs) with 95% confidence intervals (CIs). Positive SMDs favor the prehabilitation modality, whereas negative SMDs favor standard care. NMES, neuromuscular electrical stimulation.

S30C. Sensitivity Analysis: Excluding Small Modalities (n<20) (4–8 Weeks)

Pain

Treatment	Modalities with N<20 excluded	SMD	95% CI
**Education**			
Modalities with N<20 excluded		−0.08	[−1.14; 0.98]
All studies		−0.08	[−1.14; 0.98]
**Exercise & Education**			
Modalities with N<20 excluded		−0.40	[−1.78; 0.98]
All studies		−0.40	[−1.78; 0.98]
**Home−based Multidomain**			
Modalities with N<20 excluded		0.17	[−0.59; 0.93]
All studies		0.17	[−0.59; 0.93]
**Lower−extremity strength**			
Modalities with N<20 excluded		0.48	[0.00; 0.95]
All studies		0.48	[0.00; 0.95]
**Multidomain exercise**			
Modalities with N<20 excluded		0.02	[−0.39; 0.44]
All studies		0.02	[−0.39; 0.44]
**NMES**			
Modalities with N<20 excluded		0.09	[−0.64; 0.83]
All studies		0.09	[−0.64; 0.83]
**Upper−body strength**			
All studies		0.66	[−0.55; 1.86]

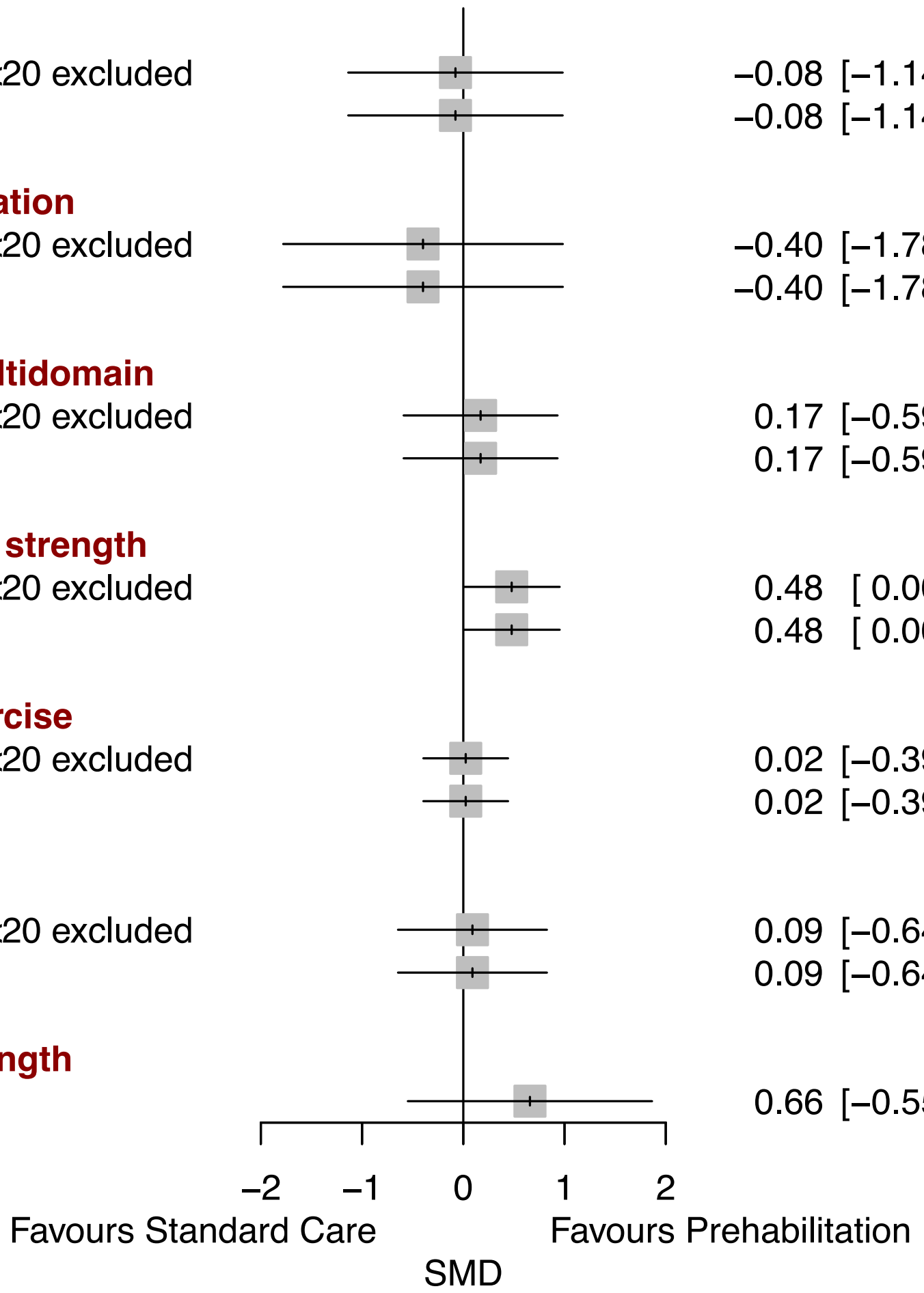

Sensitivity analysis for pain after excluding modalities with a sample size of n<20 (aerobic exercise and upper-body strength; n = 10 each). Results are presented as standardized mean differences (SMDs) with 95% confidence intervals (CIs). Positive SMDs favor the prehabilitation modality, whereas negative SMDs favor standard care. NMES, neuromuscular electrical stimulation.

S30D. Sensitivity Analysis: Excluding Small Modalities (n<20) (4–8 Weeks)

Health-related quality of life

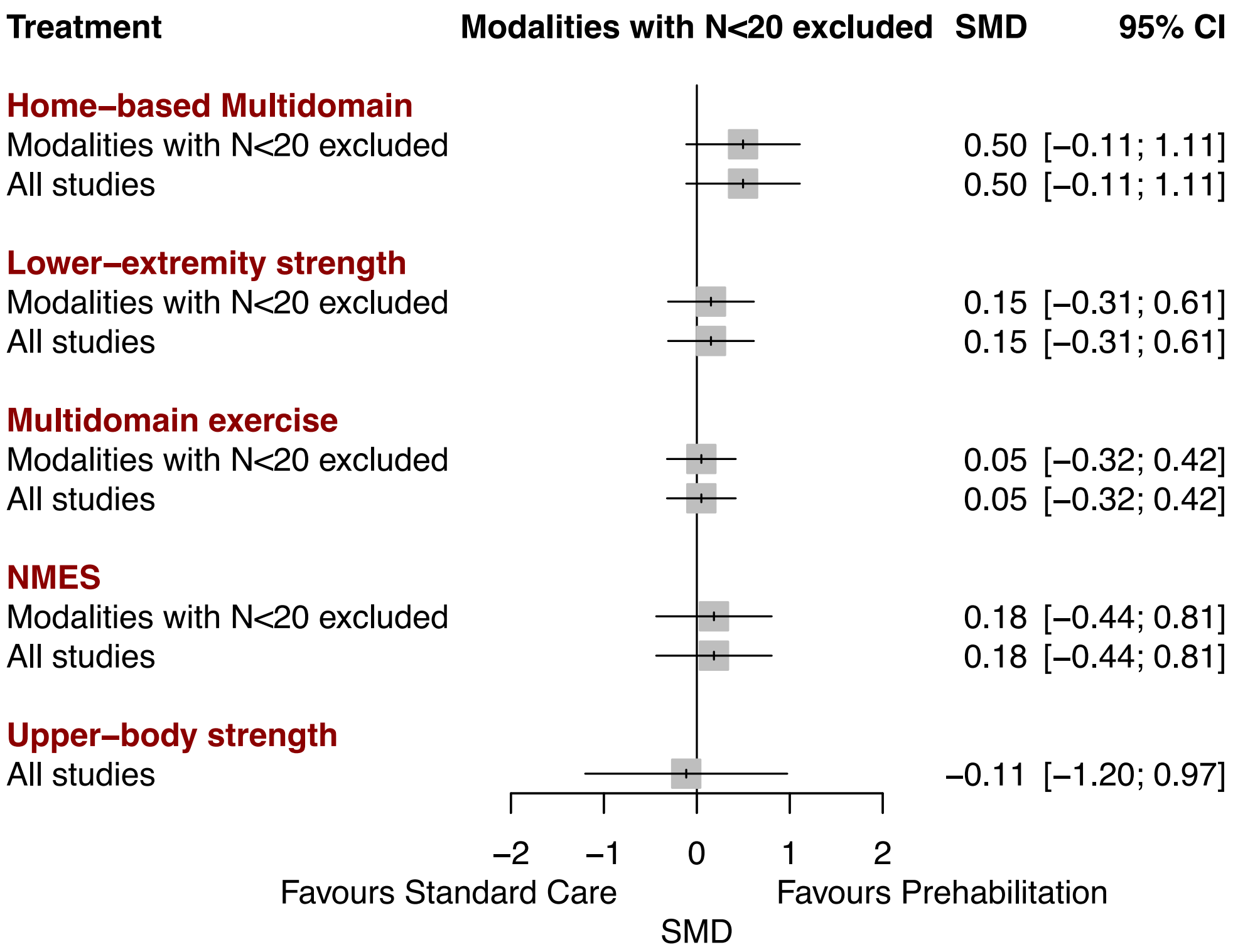

Sensitivity analysis for health-related quality of life after excluding modalities with a sample size of n<20 (aerobic exercise and upper-body strength; n = 10 each). Results are presented as standardized mean differences (SMDs) with 95% confidence intervals (CIs). Positive SMDs favor the prehabilitation modality, whereas negative SMDs favor standard care. NMES, neuromuscular electrical stimulation.
